# Evaluating the quality of evidence for gaming disorder: A summary of systematic reviews of associations between gaming disorder and depression or anxiety

**DOI:** 10.1371/journal.pone.0240032

**Published:** 2020-10-26

**Authors:** Michelle Colder Carras, Jing Shi, Gregory Hard, Ian J. Saldanha

**Affiliations:** 1 Behavioral Sciences Institute, Radboud University, Nijmegen, Netherlands; 2 Institute for Mental Health Policy Research, Centre for Addiction and Mental Health, Toronto, Ontario, Canada; 3 School of Rehabilitation Science, Faculty of Health Sciences, McMaster University, Hamilton, Ontario, Canada; 4 MGH Institute of Health Professions, Mass General Brigham, Boston, Massachusetts, United States of America; 5 Center for Evidence Synthesis in Health, Department of Health Services, Policy, and Practice, and Department of Epidemiology, Brown University School of Public Health, Providence, Rhode Island, United States of America; University of Rennes 1, FRANCE

## Abstract

Gaming disorder has been described as an urgent public health problem and has garnered many systematic reviews of its associations with other health conditions. However, review methodology can contribute to bias in the conclusions, leading to research, policy, and patient care that are not truly evidence-based. This study followed a pre-registered protocol (PROSPERO 2018 CRD42018090651) with the objective of identifying reliable and methodologically-rigorous systematic reviews that examine the associations between gaming disorder and depression or anxiety in any population. We searched PubMed and PsycInfo for published systematic reviews and the gray literature for unpublished systematic reviews as of June 24, 2020. Reviews were classified as reliable according to several quality criteria, such as whether they conducted a risk of bias assessment of studies and whether they clearly described how outcomes from each study were selected. We assessed possible selective outcome reporting among the reviews. Seven reviews that included a total of 196 studies met inclusion criteria. The overall number of participants was not calculable because not all reviews reported these data. All reviews specified eligibility criteria for studies, but not for outcomes within studies. Only one review assessed risk of bias. Evidence of selective outcome reporting was found in all reviews—only one review incorporated any of the null findings from studies it included. Thus, none were classified as reliable according to prespecified quality criteria. Systematic reviews related to gaming disorder do not meet methodological standards. As clinical and policy decisions are heavily reliant on reliable, accurate, and unbiased evidence synthesis; researchers, clinicians, and policymakers should consider the implications of selective outcome reporting. Limitations of the current summary include using counts of associations and restricting to systematic reviews published in English. Systematic reviewers should follow established guidelines for review conduct and transparent reporting to ensure evidence about technology use disorders is reliable.

## Introduction

Gaming disorder or Internet gaming disorder (IGD) is a disorder related to excessive video, computer, or online game play that results in psychological distress and/or functional impairment [1,2]. Internet gaming disorder was included as a condition for further research in the 5^th^ edition of the Diagnostic and Statistical Manual (DSM-5) and the diagnosis of gaming disorder has been added to the 11^th^ edition of the World Health Organization (WHO) International Classification of Diseases (ICD-11) [1,2]. Gaming disorder includes symptoms related to substance use disorder, such as loss of control (that continues despite negative consequences), functional impairment, distress, and/or interference with daily activities. The disorder is distinguished from other related disorders, such as technology overuse, Internet addiction, and social networking addiction [3]. Recent commentaries have described gaming disorder (which we will define here broadly to include the diagnoses of IGD or gaming disorder, problematic/pathological video gaming, and other concepts related to excessive video game play) as a clinical and public health problem in urgent need of advancements in treatment development [4,5].

Delineation and measurement of a clear construct with no overlap with other related conditions, such as gambling, Internet use, and technology use, are crucial to this field. Many recent commentaries on the need for a diagnosis of gaming disorder use terms like “Internet addiction or Gaming disorder” [6], “Internet-related disorders including gaming disorder” [4], and “Internet addiction including gaming addiction” [5], pointing to the persistent overlap in measurement of these problematic behaviors. From a public health perspective, many forms of Internet use—not just gaming—continue to be recognized as potentially problematic, as evidenced by a recently-funded international research collaborative on problematic Internet use [7].

Systematic reviews are research activities that follow established, rigorous methods to summarize all relevant evidence on specific research questions that are vital for decision-making by clinicians, patients, policy-makers, and other stakeholders. The methods include framing the research question, searching for the evidence, screening studies for eligibility, assessing risk of bias and extracting data from included studies, conducting qualitative and, where merited, quantitative syntheses, and reporting the findings. Recent decades have witnessed a surge in the number of systematic reviews conducted [8]. Multiple standards have been developed for the conduct and reporting of systematic reviews [9]. However, research has shown that reviews in some fields provide low-quality evidence, are unreliable, and can be sources of bias themselves [8,10,11]. Bias can sometimes be introduced due to methods used in the systematic review (‘meta-bias’) [12].

**Fig 1:**
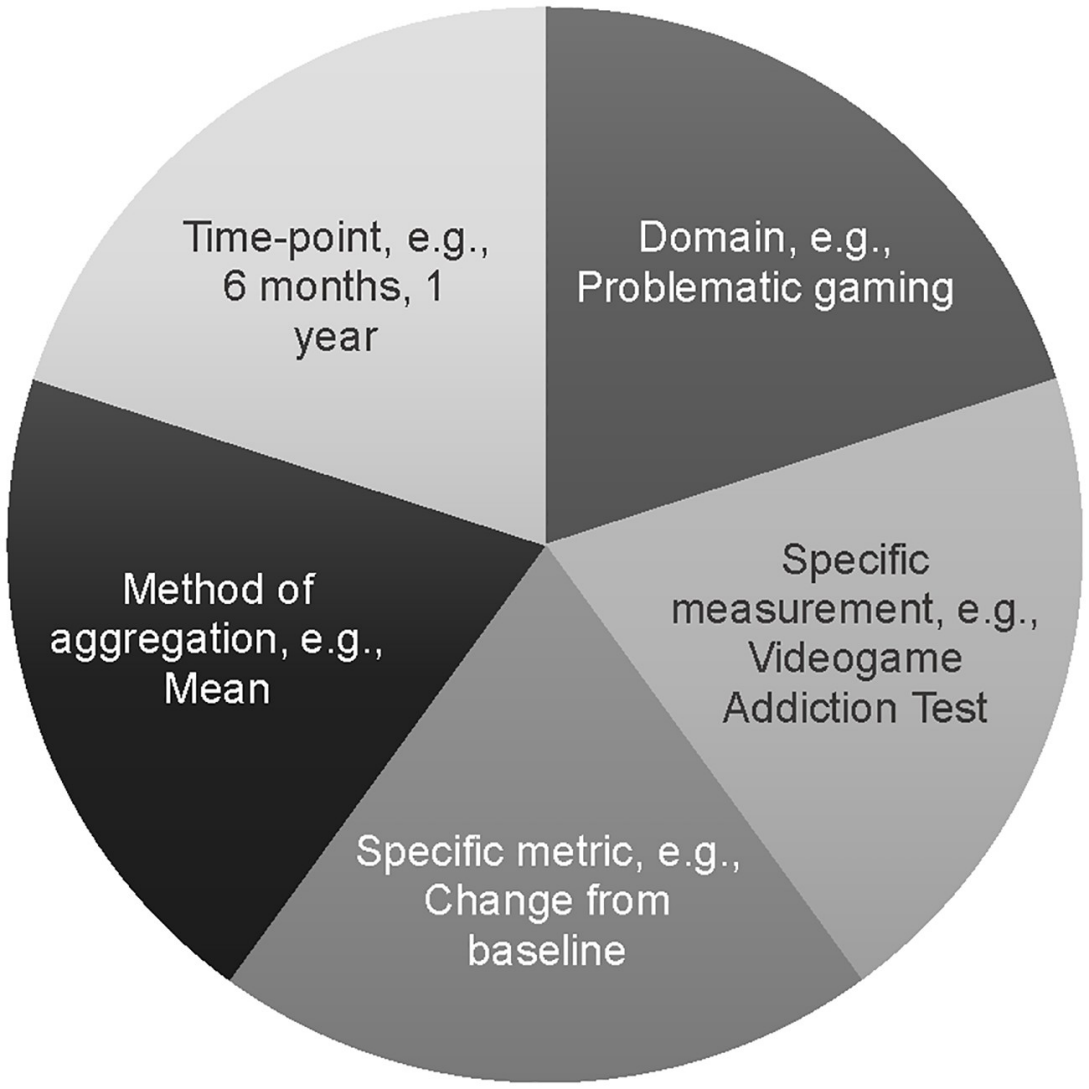
Defining outcomes for a systematic review or meta-analysis. Elements of outcome domains required for complete outcome specification in health research. Figure adapted from [15]; see also the PRISMA-P [16] statement or description of PICOS [15].

One source of meta-bias can potentially occur when a given study included in a review reports results for a given relevant outcome in multiple ways, and the reviewer must make a choice among these to determine which result(s) to extract for the review [13,14]. In this situation, choice of the result based on the largest (or smallest) magnitude of treatment effect, on statistical significance, and/or on the result that supports the reviewer’s conscious or subconscious preconceptions can be problematic and lead to bias. Such bias can be preempted by completely prespecifying the five elements of an outcome (Fig 1) [10,15]. However, complete prespecification is not always possible and/or may be considered too restrictive. Moreover, choosing specific results from multiple reported analyses from multiple data sources for a given study is a multi-dimensional problem. In one study of meta-analytic methods, an examination of outcomes reported in 14 clinical trials revealed that, depending on which outcomes from the trials were chosen by the reviewers, over 34 trillion meta-analyses were possible [13].

Now that gaming disorder has been recognized as a disorder by the WHO, ensuring systematic and accurate measurement of gaming disorder in studies *and* accurate reporting of exposures, outcomes, and conclusions in reviews are vital for ongoing decision-making regarding diagnosis, treatment, and public health interventions. Given the established association between gaming disorder and two common mental health outcomes—depression and anxiety—we limited the scope of our study to systematic reviews that included data about these outcomes. This allowed us to explore the issue of selective outcome reporting in reviews.

In this summary of systematic reviews, we assess the reliability of current reviews that have examined the association between gaming disorder and depression or between gaming disorder and anxiety in any population. We aimed to answer the following research questions to inform directions for future research and policymaking:

1. Do systematic reviews of the associations between gaming disorder and depression and between gaming disorder and anxiety meet reliability standards for systematic reviews?
2. Do systematic reviews of the associations between gaming disorder and depression and between gaming disorder and anxiety distinguish between gaming disorder and other constructs, such as Internet addiction?
3. Do systematic reviews of the associations between gaming disorder and depression and between gaming disorder and anxiety report outcomes selectively?
4. What are the associations between gaming disorder and depression and between gaming disorder and anxiety reported in reliable systematic reviews?

## Methods

This study is a summary of systematic reviews of the associations between gaming disorder and depression and between gaming disorder and anxiety in any population. The review methods, including the research question, search strategy, inclusion/exclusion criteria, and risk of bias assessment, were developed *a priori* and described in the registered protocol (PROSPERO 2018 CRD42018090651); these are also available in S1 Protocol. All data, the protocol, a list of articles excluded at the full-text screening stage with reasons for exclusion, and other supporting documentation are available on our Open Science Framework website (*see Project on OSF website*) and in Supporting Information files. In this paper, we discuss two groups of research studies: the systematic reviews (henceforth called ‘reviews’) and the primary studies included in those reviews (henceforth called ‘studies’).

We examined reviews that included studies of the associations between the exposure of gaming disorder (as defined by the review authors) and the outcomes of depression or anxiety. We restricted to reviews published in English by June 24, 2020. We excluded reviews that:

- Were narrative reviews, overviews of reviews, commentaries, and other non-systematic reviews of studies;
- Only examined Internet addiction or other technological addiction; or
- Did not report results for the associations between gaming disorder and depression or anxiety separately (e.g., we excluded reviews that only reported pooled outcomes for "mental health").

**Fig 2:**
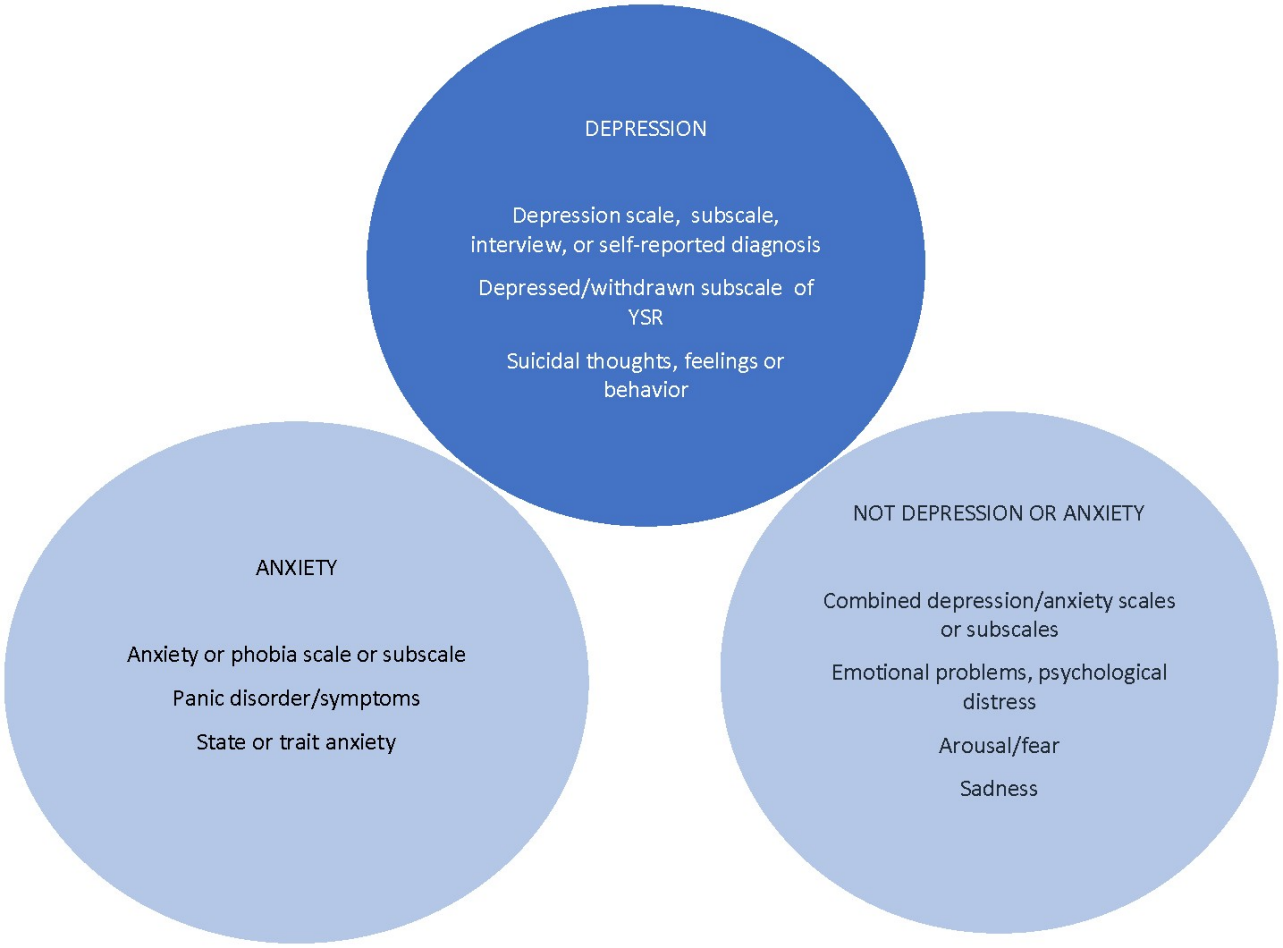
Domains used to define depression and anxiety as constructs for analysis. YSR = Youth Self Report scale.

Fig 2 illustrates how we defined the domains of depression and anxiety in our study. For the outcome of depression, we restricted to scales, subscales, diagnosis, or clinical interviews for depression or more severe single symptoms related to depression, such as suicidal ideation, but excluded measurements of nonspecific symptoms, such as low energy, sleep problems, sadness, or withdrawal from social activities. For the outcome of anxiety, we included scales, subscales, diagnosis, or clinical interviews for anxiety, social anxiety, and social phobia, but excluded measurements that combined anxiety with other constructs (e.g., anxiety/depression).

### Search strategy and screening process

We conducted electronic searches of PubMed and PsycInfo for published reviews and meta-analyses (searches were current as of June 24, 2020). Searches combined terms related to gaming disorder and terms related to depression or anxiety (S1 Search Strategies). In addition, we reviewed all years of the *Journal of Behavioral Addictions*, including its supplements, and all proceedings of the International Conference on Behavioral Addictions.

### Assessment of reliability of reviews

We adapted the definition of “reliability” of systematic reviews developed by Cochrane Eyes and Vision [17–21]. This definition, in turn, was informed by items identified from the Critical Appraisal Skills Programme (CASP), A Measurement Tool to Assess systematic Reviews (AMSTAR), and the Preferred Reporting Items for Systematic Reviews and Meta-Analyses (PRISMA) tools [9,22,23]. According to this definition, a review is reliable when its authors did each of the following:

(1) Defined eligibility criteria for including studies;
(2) Conducted a comprehensive literature search for studies (i.e., searched at least one relevant electronic database, such as PubMed and PsycInfo; used at least one other method of searching, such as searching the grey literature, searching for unpublished studies, and searching the reference lists of included articles; and were not limited to English language citations);
(3) Assessed risk of bias in individual included studies;
(4) Used appropriate methods for meta-analysis, when conducted (e.g., adequately accounting for any heterogeneity); and
(5) Presented conclusions that were supported by the evidence reported in the review.

Because we also examined each study included in the reviews, we added an additional criterion that review authors should have:

(6) Specified in the methods or protocol which outcomes from their eligible studies were included in the synthesis or synthesized *all* reported outcomes from each included study.

We classified a review as reliable only if *all six* of the criteria were met. Finally, we conducted a full assessment of the quality of the included reviews using A Measurement Tool to Assess systematic Reviews—version 2 (AMSTAR 2) [24]; the full results of this assessment are found in S1 Data Extraction.

### Assessment of other outcomes

Other outcomes included the proportion of all studies within a review that measured gaming disorder with a gaming disorder-specific instrument; the proportion of reviews that specified all elements of an outcome; and the specific review- and study-level associations between gaming disorder and depression and anxiety. All reported associations within the studies were extracted from the original study reports and characterized as present and positive, present and negative, present and null, unclear, or absent. The count and type (positive, null, negative, unclear, or absent) of results for each study were compared with the results reported for each study in the reviews. We also made several comparisons regarding overall conclusions about the associations between gaming disorder and depression and between gaming disorder and anxiety by comparing bivariable versus multivariable analyses, cross-sectional versus longitudinal analyses, and results from reviews classified as reliable versus results from all reviews.

### Data extraction

We developed and pilot tested a data extraction form using Microsoft Excel^®^, based on the form developed by Mayo-Wilson et al. [17]. We added questions relevant to reviews of epidemiological studies [25]. During the initial data extraction, we noticed discrepancies in how specific studies were reported in the reviews, resulting in potential selective outcome reporting at the review level. To ensure that we evaluated this potential source of bias, we expanded the scope of our preregistered protocol to include examining study-level outcomes and how they were reported in reviews.

Two investigators from among MCC, JS, and GH extracted data from each review, consulting the third investigator for resolution of discrepancies where needed. If a review did not have a summary of findings table that included the total number of studies mentioned in the results or in supplementary material, we extracted data for all studies mentioned in text or tables of the Results section. Data on depression and anxiety outcomes within each study of each review were extracted by one investigator. Extracted data for a 10% random sample of studies were validated by the second and third investigator.

Data extracted from the reviews included information on methods for specifying eligibility criteria and outcomes, specific measurements (e.g., scales) of depression and anxiety in included studies, analyses conducted, whether and how review authors assessed risk of bias in included studies, specific measurement (e.g., scales) of gaming disorder in included studies, and all items from the AMSTAR 2 tool.

**Fig 3:**
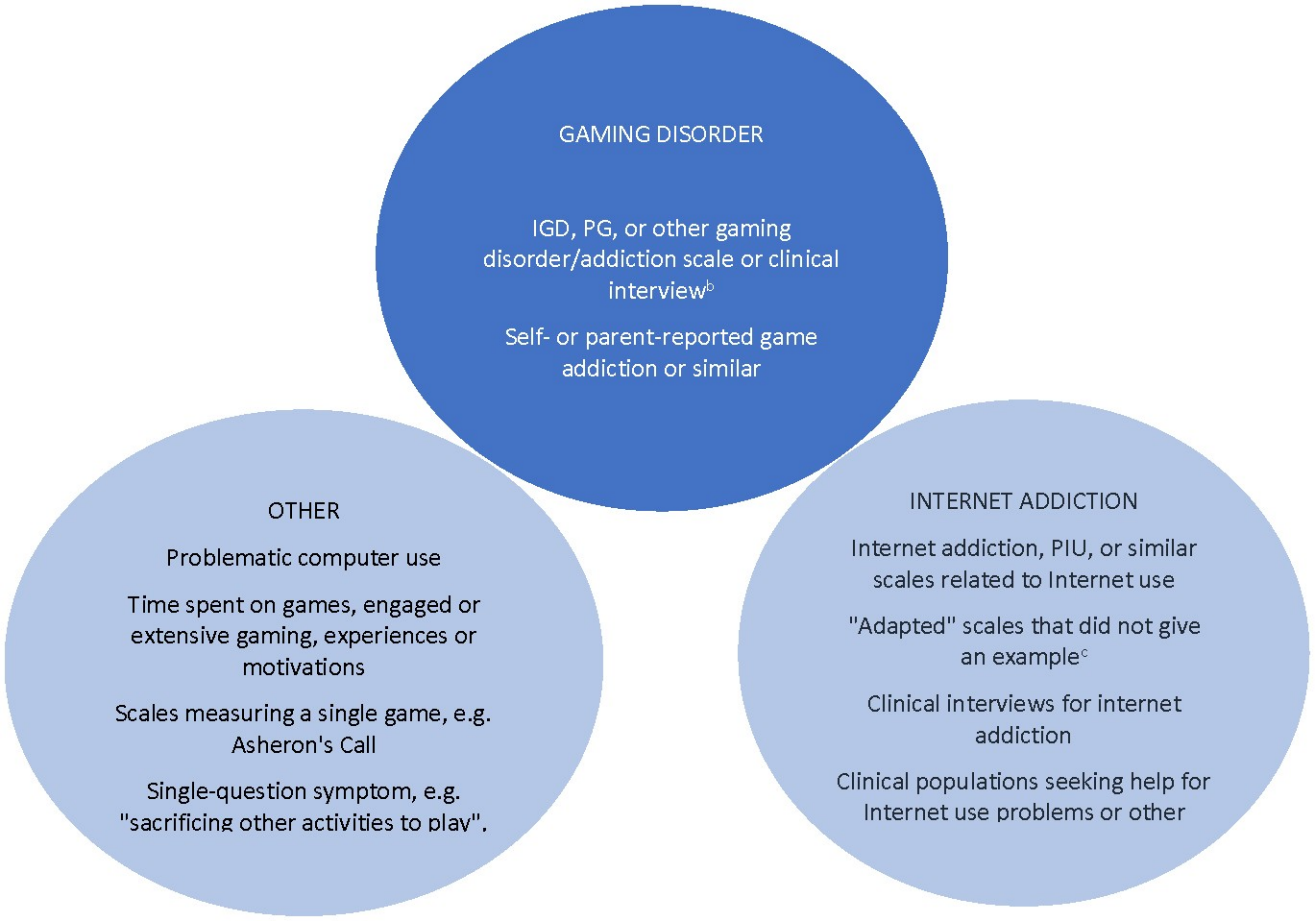
Domains used to define gaming disorder as a construct for analysis. IGD = Internet gaming disorder; PG = problematic gaming; PIU = problematic Internet use. (a) Sensitivity analysis: Clinical population of those seeking help for gaming-related problems but an Internet addiction scale was used. (b) Including those adapted from Internet addiction scales where an example question is given. (c) Where scales referenced appendices or other papers, these were also searched for example questions.

We summarize below the three conditions that had to be met for a specific measurement or scale to be classified as asssesing gaming disorder (Fig 3):

- The specific measurement or scale asked questions about computer, video, online, or digital game use in general, rather than just a single game (e.g., World of Warcraft^®^).
- The specific measurement or scale asked questions about gaming or online gaming rather than Internet or computer use in general (e.g., did not use only an Internet addiction measure, such as the Young Internet Addiction Test or the Compulsive Internet Use Test). If a study mentioned adapting a scale for video games and gave an example of an adapted question, we classified that scale as measuring gaming disorder. Otherwise, we classified the measurement according to the original scale from which it was adapted. We also conducted a sensitivity analysis to examine how our findings differed when other measurements (e.g., the Young Internet Addiction Test) were used with a clinical population diagnosed with gaming disorder. When the clinical population was unclear or was not diagnosed with gaming disorder and Internet addiction scales or other specific measurements/scales/interviews were used, we did not characterize this as gaming disorder (e.g., Young Internet Addiction Test in a clinical population of patients with gambling disorder).
- The specific measurement or scale asked questions about specific symptoms of gaming disorder rather than only experiences related to video game use in general, such as time spent playing games or the experience of time loss.

Data on depression and anxiety consisted of study scale, type of analysis, direction of association (positive, negative, or null), and how each review reported the outcome of the study (positive, negative, null, unclear, or absent).

### Quality assessment

See section above entitled ‘Assessment of reliability of reviews’.

### Strategy for data synthesis and reporting

We narratively describe the characteristics of included reviews and their reliability. Because measurements of exposures and outcomes were heterogeneous, we present counts of positive or null/negative outcomes from studies and how they were reported in reviews [26]. Because consistency is one factor that supports strength of evidence, we compared tallies of qualitative associations from the multiple outcomes reported in studies. We described associations to be ‘positive and consistent’ at the study level if the count of statistically-significant positive associations was greater than the total number of negative or null associations. We described an association as ‘null’ if there were more null findings or negative associations than positive. We conducted a sensitivity analysis to examine the impact of measuring gaming disorder with a scale for Internet addiction in a clinical population of individuals with gaming disorder. All extracted data and derived variables are available in S1 Dataset.

The PRISMA checklist [9] for the current study is available in S1 PRISMA Checklist. This study was conducted using publicly-available information and therefore did not require Institutional Board (IRB) approval.

## Results

**Fig 4:**
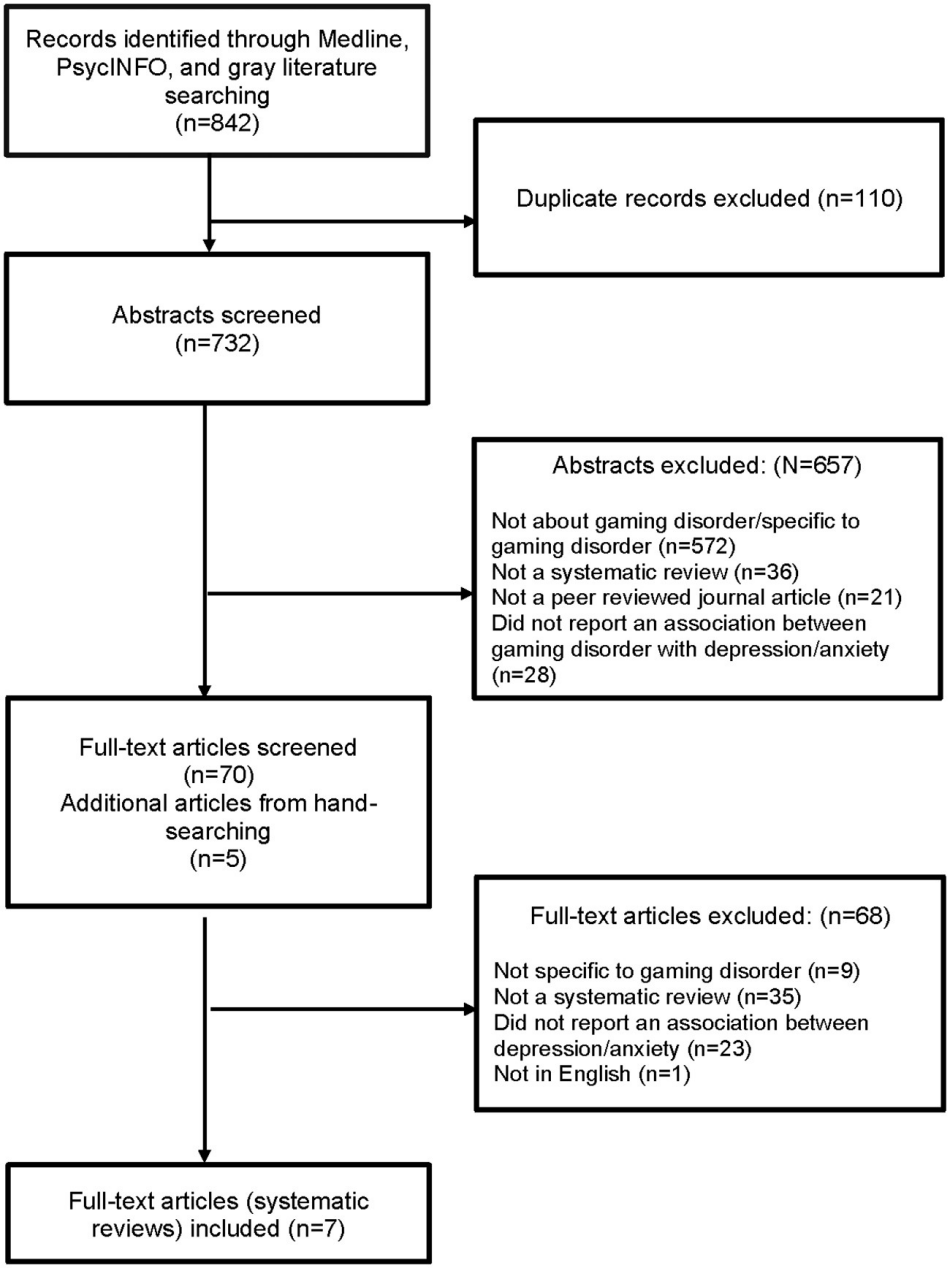
PRISMA flow diagram.

The searches yielded 842 records, of which, seven reviews were eligible for inclusion in this overview (Fig 4). The most frequent reasons for excluding articles (at the full-text screening stage) were that they were not a systematic review or did not specify methods (n = 35), did not report associations between gaming disorder and anxiety or depression (n = 23), and were not specific to gaming disorder (e.g., being about behavioral addictions in general) (n = 9).

### Review characteristics

The characteristics of the seven included reviews are reported in Table 1. They included a total of 196 unique studies. The number of included studies per review ranged from 24 to 63, with a mean of 46. Most studies (61.7%) were included in only one review each.

**Table 1:** Review characteristics and reliability criteria.

						Reliability criteria					
First author, year	Number of included studies^a^	Total number of participants across all included studies^b^	Participant populations	Years of publication of included studies^c^	Number (%) studies measuring problematic gaming^d^	(1) Defined eligibility criteria?	(2) Conducted a comprehensive search?	(3) Assessed of risk of bias?	(4) Used appropriate methods to combine results? ^e^	(5) Conclusions about depression and anxiety supported by evidence?	(6) Specified which outcomes would be included in the synthesis?
Sugaya 2019 [27]	51	Unclear	Age 0–28	Until 2018	25(56.8)	Yes	Yes	No	Yes	No	No
González-Bueso 2018 [28]	24	53,889	Any	2011–2017	17 (70.8)	Yes	Yes	No	Unclear	No	No
Mihara 2017 [29]	47	127,749	Any	Until 2016	44 (93.6)	Yes	No	No	Yes	No	No
Männikkö 2017 [30]	50	129,430	>12.5 years	2005–2016	41 (82.0)	Yes	Yes	Yes	No	No	No
King 2013 [31]	63	58,415	Any	2000–2012	39 (61.9)	Yes	Yes	No	Yes	No	No
Kuss 2012 [32]	58	Unclear	Any	2000–2010	33 (56.9)	Yes	No	No	Yes	No	No
Kuss 2012 [33]	30	72,825	Children	2000–2011	20 (66.7)	Yes	Yes	No	Yes	No	No

Notes:

^(a)^ The number of included studies for a review is taken from the PRISMA flow diagram (where possible) or from reports in the text or tables of each review.

^(b)^The number of participants was taken directly from the text where possible or calculated from other information that was reported in the review.

^(c)^ If no years were given, the end year was listed as one year prior to the year of publication.

^(d)^ Proportion of studies measuring problematic gaming was assessed out of all studies mentioned in the review. This did not always match the number of studies that were said to be included in the review in the abstract, methods, or results.

^(e)^ most reviews did not combine results quantitatively.

### Research question 1: Assessment of review reliability

We found that none of the seven included reviews fulfilled all six criteria for reliability. All reviews defined eligibility criteria and most reviews (six of seven) conducted comprehensive database searches (Table 1). No review defined outcomes using all five elements of completely-specified outcomes (i.e., domain, specific measurement, specific metric, method of aggregation, and time points). No reviews specified which outcomes of a study would be used in synthesis. One review specified that it would consider only study effect sizes from multivariable analyses, classifying full associations as “…a correlation was found for both genders after multivariable analyses” or partial associations as “…correlation was identified for only one gender” [28]. Other reviews did not specify how outcomes would be included, although some mentioned that "factors", "disorders", "comorbidity", "health-related outcomes", or "psycho-social features" "associated with" problematic gaming were "identified" [28], "ascertained" [29], or "extracted" [30,31].

Although all reviews acknowledged heterogeneity in measurement of problematic gaming, only one review assessed risk of bias systematically [30]. In this context, because five studies chose to conduct qualitative syntheses instead of quantitative syntheses (i.e., meta-analyses), we considered their results to have been combined appropriately. In one review, results were combined quantitatively despite a very high amount of statistical heterogeneity among studies (suggested by an I^2^ value of 98%) [30]. Another review classified effect sizes as small, medium, or large and presented a table of counts of effect sizes for four mental health outcomes as a way to address heterogeneity in measurement [28]. Most reviews discussed limitations at the study, outcome, and review level, but two reviews did not discuss limitations systematically [27,29].

Assessment of AMSTAR 2 criteria showed that no study met all criteria, and some criteria were lacking in all studies. Full results can be found in S1 Data Extraction.

**Fig 5:**
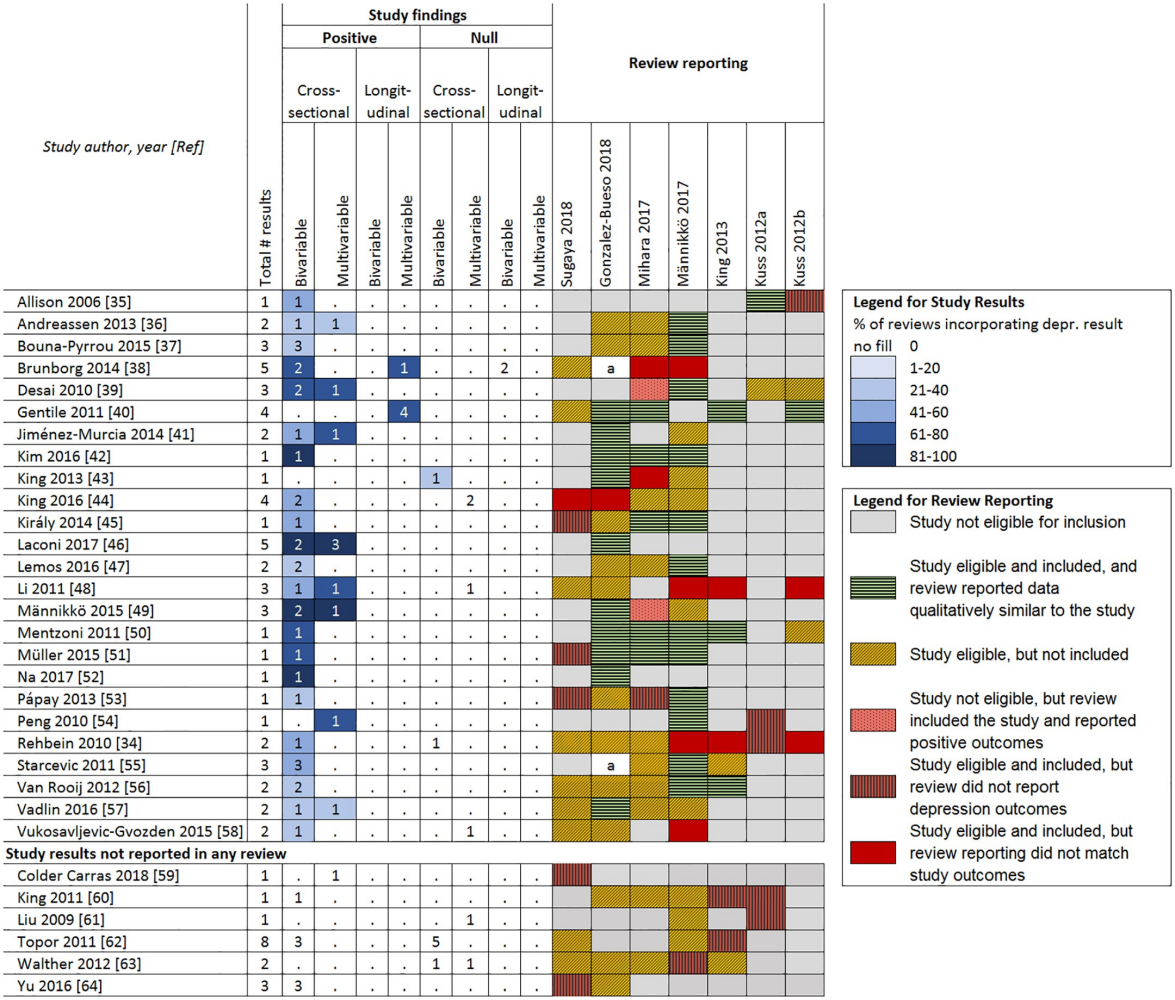
Associations between problematic gaming and depression. a = Composite reporting of outcomes in review made comparisons difficult.

**Fig 6:**
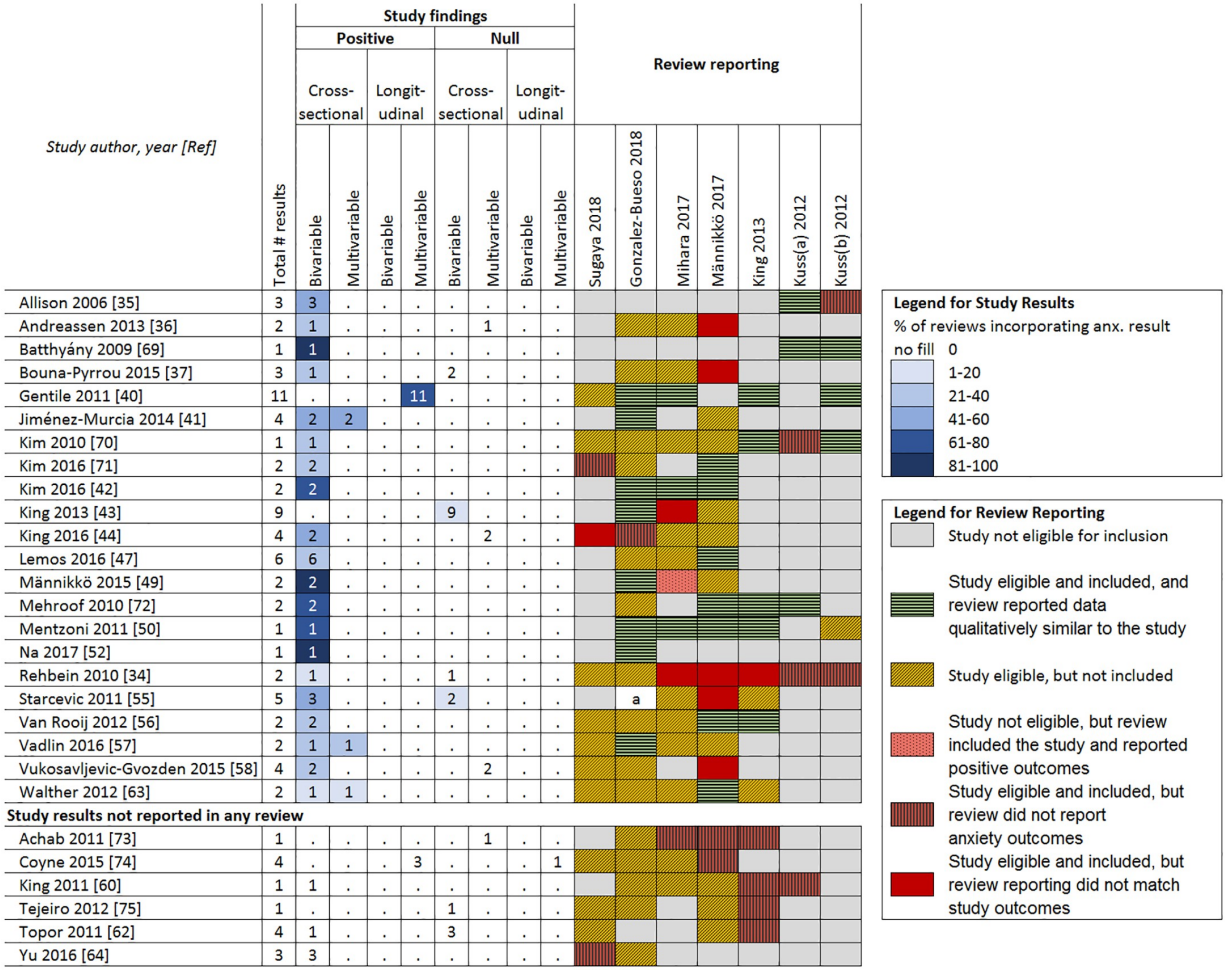
Study reporting of associations between problematic gaming and anxiety. a = Composite reporting of outcomes in review made comparisons difficult.

Because of the lack of clarity around how study outcomes were selected, the reporting of outcomes that was inconsistent with study findings (see Figs 5 and 6), the inclusion of studies that did not measure gaming disorder, and the lack of systematic assessment of bias (except for one review [30]), we determined that review conclusions were not supported by the evidence from included studies. This is further explored in the following sections.

### Research question 2: Distinguishing between gaming disorder and other concepts

Based on our definition for measurement of gaming disorder (Fig 3), no review focused *only* on studies that measured gaming disorder. The percentage of studies within a review that measured gaming disorder ranged from 56.8% to 93.6%. On sensitivity analysis, where measurement of gaming disorder also included using an Internet addiction scale in a gaming disorder clinical population, the percent remained similar, ranging from 58.6% to 93.6%.

### Research question 3: Reporting of associations between gaming disorder and depression or anxiety

Figs 5 and 6 report the positive and null associations for the depression and anxiety outcomes according to analysis type (bivariable/multivariable, cross-sectional, and longitudinal), their frequency of being incorporated into reviews, and how they are represented/reported in reviews (e.g., not reported, not eligible, report conflicts with outcomes). The shades of blue highlighting pertain to different percentages of reviews that incorporated a given relevant result from a given study (darker highlighting indicates higher percentages). Note that only two negative (inverse) associations were found (between gaming disorder and anxiety) and because these represent findings that were not positive and significant, they were included in the count of null findings. Overall, only the review by González-Bueso and colleagues [28] reported any null results about depression or anxiety from any study.

#### Associations between gaming disorder and depression

For the depression outcome (Fig 5, including citations [34–64]), of the 31 studies reporting associations between gaming disorder and depression, results from 25 were included in at least one review. We found frequent under-incorporation of null results for the depression outcome by the reviews, as suggested by the paucity of blue cell highlights in the null columns. For example, the 2010 study by Rehbein and colleagues [34] reported two findings related to depression—a positive association between gaming disorder and suicidal thoughts in one subsample, but a null association between gaming disorder and self-reported depression in the full sample. However, the three reviews that included this study and reported results for depression all reported them as positive [30,31,33].

Ten of the 31 studies reporting associations between gaming disorder and depression reported both bivariable and multivariable analyses. In five of these 10 studies, results from both analyses were robust and positive, while five studies reported inconsistent results. Whether consistent or inconsistent in the studies, positive results were incorporated into five of the six reviews that included depression findings from the study.

Only one study reporting an association between gaming disorder and depression examined both cross-sectional and longitudinal associations, and the results were inconsistent [38]. However, the results were incorporated into two of three reviews as showing a positive association. The final review used a composite definition when reporting associations, which made comparisons difficult [28].

Six studies reported additional cross-sectional depression results that were not incorporated into any review. Three of these studies reported null findings and in one of those cases, results were null in both bivariable and multivariable analyses. An additional 15 studies were mentioned by reviews as reporting associations between gaming disorder and depression, but using domain definitions in Figs 2 and 3, these were not found (S1 Output contains full results). All but one of the six reviews that included these studies reported these as positive associations. Some reasons for this were: studies used a measure of Internet addiction or other exposure (e.g., "excessive" gaming), studies reported a composite measure (depression/anxiety/stress) as depression, and possible mistake in citation or data extraction (e.g., reporting data for a problematic Internet use subgroup rather than problematic gaming subgroup).

In a sensitivity analysis that included studies where a broad Internet addiction scale (rather than a gaming disorder scale) was used to measure gaming disorder in a clinical population identified as having gaming disorder, one additional study [65] was found to have positive associations and was reported in the single review that included it as positive, while another three studies [66–68] had null findings which were not reported by the three reviews that included them.

#### Associations between gaming disorder and anxiety

Of the 28 studies that reported associations between anxiety and gaming disorder, results from only 22 of these studies were incorporated into reviews (Fig 6, including citations [34–75]).

Six studies reported both bivariable and multivariable associations; half of these showed inconsistent results. Whether consistent or inconsistent, reviews incorporated only positive findings. Six studies reported results that were not incorporated into any review; four of these had inconsistent or null findings. An additional nine studies were mentioned by reviews as reporting associations between an gaming disorder and anxiety, but using domain definitions in Fig 3, these were not found. All but one of the three reviews that incorporated these studies reported these associations as positive.

In the sensitivity analysis, one additional study [65] reported inconsistent associations in bivariable and multivariable analysis and was reported as positive in the one review that contained it.

### Research question 4: Association between gaming disorder and depression or anxiety in reliable reviews

Overall, no review satisfied all the criteria we used to identify reliable reviews, so we could not address this research question.

## Discussion

This summary of systematic reviews found methodological problems in all seven systematic reviews that reported on associations between gaming disorder and depression or anxiety; no reviews could be classified as reliable based on established criteria. Although most systematic reviews studied herein defined their criteria for selecting studies and conducted a comprehensive search, each review was rated as unreliable because of one or more of the other criteria. Because of the poor pre-specification of how outcomes would be included, it is difficult to draw conclusions from these reviews regarding associations between gaming disorder and depression or anxiety that are supported by evidence. These findings suggest that the way systematic reviews of gaming disorder have been reporting results and drawing conclusions may have introduced bias into the gaming disorder literature, possibly misleading future research, policy-making, and patient care.

Various concerns identified during this summary of systematic reviews are worthy of further discussion. We present these in the hope that the current work drives important progress in research on gaming disorder and other types of behavioral addictions in the coming years.

First, the existing reviews seldom incorporated null findings (i.e., lack of associations) or negative findings (i.e., inverse associations) from included studies even when the studies reported such findings. This is a major concern because it seems to represent selective outcome reporting at the review level. It is vital to conduct systematic reviews and meta-analyses in ways that are replicable and consistent with best practices to ensure that all evidence is reported and that relevant studies and findings are not overlooked. Selecting which outcomes of studies to include in a review without specifying the process, which has been labelled “cherry-picking” in the clinical epidemiology literature, can lead to biased conclusions at the review level [10,13]. Completely specifying all elements of outcomes (i.e., domain, specific measurements, specific metrics, methods of aggregation, and time-points of interest) or explicitly noting whether all variations of a given outcome element will be extracted is the current standard for evidence synthesis [15,26,76]. As incomplete outcome specification may lead to trillions of potential combinations of meta-analytic results [13], it is inappropriate to draw meaningful and reliable conclusions about associations between gaming disorder and the common mental health problems of depression and anxiety from the reviews summarized in this paper. Selective reporting of outcomes can be hard to detect, and further research into the impact of selective inclusion of results in reviews is needed to advance the understanding of this form of bias on evidence synthesis [77,78].

A second major concern is that reviews did not limit evidence synthesis and conclusions to studies that measured the construct of gaming disorder and at times used overly-broad definitions of depression and anxiety (e.g., combined depression, anxiety, and stress), which might have led to reports of associations between gaming disorder and depression or anxiety when none might exist.

Although more recent reviews had higher proportions of gaming-only measures, even recent reviews included studies that used Internet addiction questions to measure gaming disorder. Distinguishing between problematic behaviors is vital in ongoing research of problematic technology use and will continue to be relevant to shaping the future of health policy and government regulation of the Internet, video games, and other forms of media and technology. Ensuring that systematic and accurate measurement of gaming disorder in studies *and* accurate measurement and reporting of exposures, outcomes, and conclusions in reviews are vital to inform ongoing decision making regarding diagnosis, treatment, and public health interventions.

A third major concern is that only one review [30] reported a systematic assessment of risk of bias using multiple domains, which has long been a best practice in conducting systematic reviews [79–83]. When the risk of bias is not systematically assessed and reported, conclusions from studies included in reviews may be seen as valid and reliable when they may actually reflect biases, such as selection bias, information bias, and/or confounding [84]. When evidence of questionable methodologic quality is used to inform public health or policy decisions, such decisions may be misguided.

To our knowledge, the current analysis is the first comprehensive examination of selective outcome reporting in systematic reviews of gaming disorder, a relatively new clinical entity. Due to this selective outcome reporting, incomplete outcome specification, and lack of systematic assessment of risk of bias, we found no reviews that could be considered reliable. These findings suggest that the evidence base of systematic reviews of associations between gaming disorder and the most common mental health problems must be improved.

### Limitations

The current overview is subject to certain limitations. First, at the level of the studies we found significant inconsistencies in measurement and analysis, which were dealt with by describing counts of associations by type. While this is a somewhat reductionist approach to summarizing results, it helps paint a picture. Relatedly, no reviews defined outcomes completely. Second, we limited our analysis to systematic reviews published in English. It is possible that our findings may have been different had we included reviews in other languages. Third, we focused on the outcomes of depression or anxiety. This narrow scope made a detailed analysis possible, but findings regarding associations between gaming disorder and other outcomes (e.g., attention-deficit hyperactivity disorder) may have been different. However, due to the ubiquitous nature of selective outcome reporting, in particular, in the reviews herein, we consider this to be unlikely. Fourth, we defined the constructs of gaming disorder, depression, and anxiety very specifically; had we used broader definitions, our findings would likely be different. However, using a narrow definition was our aim. We do not attempt to draw conclusions at the study level (the 196 studies) due to the inconsistency within studies and the uncertain nature of the examined evidence. Finally, in our search of PubMed we used the PubMed publication type filters of “systematic review”, “review,” or “meta-analysis”, while we broadened our search of PsycInfo to include these terms as text-words in all fields. For this reason, it is possible that we missed some systematic reviews that were only available in PubMed and were not indexed using these terms or did not contain these terms in the title, abstract, publication type, or keywords.

### Conclusions

To advance the field of addictive behaviors and ensure that research measures and reports constructs rigorously and with clarity, existing standards for systematic review conduct and reporting should be followed. Increasing transparency of reviews and minimizing the risk of bias requires the effort of multiple agents. Authors must prospectively register protocols (including adequately specifying outcomes); use reporting guidelines, such as those from the EQUATOR Network; and share data, analysis code, and other study materials. Journals and editors must verify authors’ adherence to reporting guidelines [77]. Although public health decision-making should always proceed on the best available evidence [85], the data provided in this paper suggest that limiting technology-related diagnoses to video game play is not likely to accurately reflect the findings of years of research surrounding problematic technology use. A highly rigorous systematic review that fully specifies outcome domains is needed to clarify the potential mental health problems associated with problematic technology behaviors, including video gaming and Internet use.

## Supporting information

S1 PRISMA Checklist PRISMA checklist for reporting of our systematic review. (PDF)

S1 Protocol PROSPERO registration for our systematic review protocol. (PDF)

S1 Search Strategy Search strategies. (PDF)

S1 Data Extraction Data extraction at the review level, including AMSTAR 2. (XLSX)

S1 Dataset Complete analysis dataset containing extracted and derived variables. (DTA)

S1 Output Output of analysis. (DOCX)

## References

[1] World Health Organization. International classification of diseases for mortality and morbidity statistics (11th Revision). World Health Organization; 2018. https://icd.who.int/browse11/l-m/en.

[2] American Psychiatric Association. Diagnostic and Statistical Manual of Mental Disorders, Fifth Edition. Arlington, VA: American Psychiatric Association; 2013. dsm.psychiatryonline.org.

[3] Starcevic V, Billieux J. Does the construct of Internet addiction reflect a single entity or a spectrum of disorders? Clin Neuropsychiatry J Treat Eval. 2017;14: 5–10. Available: http://search.ebscohost.com/login.aspx?direct=true&db=psyh&AN=2017-10293-002&site=ehost-live&scope=site.

[4] Rumpf H-J, Achab S, Billieux J, Bowden-Jones H, Carragher N, Demetrovics Z, et al Including gaming disorder in the ICD-11: The need to do so from a clinical and public health perspective. J Behav Addict. 2018; 1–6. 10.1556/2006.7.2018.59 30010410 PMC6426367

[5] Saunders JB, Hao W, Long J, King DL, Mann K, Fauth-Bühler M, et al Gaming disorder: Its delineation as an important condition for diagnosis, management, and prevention. J Behav Addict. 2017;6: 271–279. 10.1556/2006.6.2017.039 28816494 PMC5700714

[6] Higuchi S, Nakayama H, Mihara S, Maezono M, Kitayuguchi T, Hashimoto T. Inclusion of gaming disorder criteria in ICD-11: A clinical perspective in favor. J Behav Addict. 2017;6: 293–295. 10.1556/2006.6.2017.049 28816497 PMC5700724

[7] Fineberg NA, Demetrovics Z, Stein DJ, Ioannidis K, Potenza MN, Grünblatt E, et al Manifesto for a European research network into Problematic Usage of the Internet. Eur Neuropsychopharmacol J Eur Coll Neuropsychopharmacol. 2018;28: 1232–1246. 10.1016/j.euroneuro.2018.08.004 30509450 PMC6276981

[8] Page MJ, Shamseer L, Altman DG, Tetzlaff J, Sampson M, Tricco AC, et al Epidemiology and Reporting Characteristics of Systematic Reviews of Biomedical Research: A Cross-Sectional Study. PLoS Med. 2016;13: e1002028 10.1371/journal.pmed.1002028 27218655 PMC4878797

[9] Moher D, Liberati A, Tetzlaff J, Altman DG, PRISMA Group. The PRISMA Group. Preferred reporting items for systematic reviews and meta-analyses: the PRISMA statement. PLoS Med. 2009;6: e1000097 10.1371/journal.pmed.1000097 19621072 PMC2707599

[10] Mayo-Wilson E, Fusco N, Li T, Hong H, Canner JK, Dickersin K, et al Multiple outcomes and analyses in clinical trials create challenges for interpretation and research synthesis. J Clin Epidemiol. 2017;86: 39–50. 10.1016/j.jclinepi.2017.05.007 28529187

[11] Pussegoda K, Turner L, Garritty C, Mayhew A, Skidmore B, Stevens A, et al Systematic review adherence to methodological or reporting quality. Syst Rev. 2017;6: 131 10.1186/s13643-017-0527-2 28720117 PMC5516390

[12] Goodman S, Dickersin K. Metabias: a challenge for comparative effectiveness research. Ann Intern Med. 2011;155: 61–62. 10.7326/0003-4819-155-1-201107050-00010 21727295

[13] Mayo-Wilson E, Li T, Fusco N, Bertizzolo L, Canner JK, Cowley T, et al Cherry-picking by trialists and meta-analysts can drive conclusions about intervention efficacy. J Clin Epidemiol. 2017 10.1016/j.jclinepi.2017.07.014 28842290

[14] Saldanha IJ, Lindsley KB, Money S, Kimmel HJ, Smith BT, Dickersin K. Outcome choice and definition in systematic reviews leads to few eligible studies included in meta-analyses: a case study. BMC Med Res Methodol. 2020;20: 30 10.1186/s12874-020-0898-2 32046643 PMC7014938

[15] Saldanha IJ, Dickersin K, Wang X, Li T. Outcomes in Cochrane systematic reviews addressing four common eye conditions: an evaluation of completeness and comparability. PloS One. 2014;9: e109400 10.1371/journal.pone.0109400 25329377 PMC4199623

[16] Moher D, Shamseer L, Clarke M, Ghersi D, Liberati A, Petticrew M, et al Preferred reporting items for systematic review and meta-analysis protocols (PRISMA-P) 2015 statement. Syst Rev. 2015;4: 1 10.1186/2046-4053-4-1 25554246 PMC4320440

[17] Mayo-Wilson E, Ng SM, Chuck RS, Li T. The quality of systematic reviews about interventions for refractive error can be improved: a review of systematic reviews. BMC Ophthalmol. 2017;17: 164 10.1186/s12886-017-0561-9 28870179 PMC5584039

[18] Saldanha IJ, Lindsley KB, Lum F, Dickersin K, Li T. Reliability of the Evidence Addressing Treatment of Corneal Diseases: A Summary of Systematic Reviews. JAMA Ophthalmol. 2019;137: 775–785. 10.1001/jamaophthalmol.2019.1063 31070698 PMC6512297

[19] Le JT, Qureshi R, Twose C, Rosman L, Han G, Fapohunda K, et al Evaluation of Systematic Reviews of Interventions for Retina and Vitreous Conditions. JAMA Ophthalmol. 2019 10.1001/jamaophthalmol.2019.4016 31600387 PMC6802257

[20] Golozar A, Chen Y, Lindsley K, Rouse B, Musch DC, Lum F, et al Identification and Description of Reliable Evidence for 2016 American Academy of Ophthalmology Preferred Practice Pattern Guidelines for Cataract in the Adult Eye. JAMA Ophthalmol. 2018;136: 514–523. 10.1001/jamaophthalmol.2018.0786 29800249 PMC6145658

[21] Lindsley K, Li T, Ssemanda E, Virgili G, Dickersin K. Interventions for Age-Related Macular Degeneration: Are Practice Guidelines Based on Systematic Reviews? Ophthalmology. 2016;123: 884–897. 10.1016/j.ophtha.2015.12.004 26804762 PMC4808456

[22] Shea BJ, Bouter LM, Peterson J, Boers M, Andersson N, Ortiz Z, et al External Validation of a Measurement Tool to Assess Systematic Reviews (AMSTAR). PLOS ONE. 2007;2: e1350 10.1371/journal.pone.0001350 18159233 PMC2131785

[23] Critical Appraisal Skills Programme. CASP systematic review checklist. 2018. https://casp-uk.net/wp-content/uploads/2018/01/CASP-Systematic-Review-Checklist_2018.pdf.

[24] Shea BJ, Reeves BC, Wells G, Thuku M, Hamel C, Moran J, et al AMSTAR 2: a critical appraisal tool for systematic reviews that include randomised or non-randomised studies of healthcare interventions, or both. BMJ. 2017;358 10.1136/bmj.j4008 28935701 PMC5833365

[25] Stroup DF, Berlin JA, Morton SC, Olkin I, Williamson GD, Rennie D, et al Meta-analysis of observational studies in epidemiology: a proposal for reporting. Meta-analysis Of Observational Studies in Epidemiology (MOOSE) group. JAMA J Am Med Assoc. 2000;283: 2008–2012. 10.1001/jama.283.15.2008 10789670

[26] Chandler J, Cumpston M, Li T, Page MJ, Welch VA. Cochrane Handbook for Systematic Reviews of Interventions. 2nd edition Higgins JPT, Thomas J, editors. Chichester (UK): John Wiley & Sons; 2019.

[27] Sugaya N, Shirasaka T, Takahashi K, Kanda H. Bio-psychosocial factors of children and adolescents with internet gaming disorder: a systematic review. Biopsychosoc Med. 2019;13: 3 10.1186/s13030-019-0144-5 30809270 PMC6374886

[28] González-Bueso V, Santamaría JJ, Fernández D, Merino L, Montero E, Ribas J. Association between Internet Gaming Disorder or Pathological Video-Game Use and Comorbid Psychopathology: A Comprehensive Review. Int J Environ Res Public Health. 2018;15 10.3390/ijerph15040668 29614059 PMC5923710

[29] Mihara S, Higuchi S. Cross‐sectional and longitudinal epidemiological studies of internet gaming disorder: A systematic review of the literature. Psychiatry Clin Neurosci. 2017;71: 425–444. 10.1111/pcn.12532 28436212

[30] Männikkö N, Ruotsalainen H, Miettunen J, Pontes HM, Kääriäinen M. Problematic gaming behaviour and health-related outcomes: A systematic review and meta-analysis. J Health Psychol. 2017; 1359105317740414. 10.1177/1359105317740414 29192524

[31] King DL, Haagsma MC, Delfabbro PH, Gradisar M, Griffiths MD. Toward a consensus definition of pathological video-gaming: A systematic review of psychometric assessment tools. Clin Psychol Rev. 2013;33: 331–342. 10.1016/j.cpr.2013.01.002 23396015

[32] Kuss DJ, Griffiths MD. Internet gaming addiction: A systematic review of empirical research. Int J Ment Health Addict. 2012;10: 278–296. 10.1007/s11469-011-9318-5

[33] Kuss DJ, Griffiths MD. Online gaming addiction in children and adolescents: A review of empirical research. J Behav Addict. 2012;1: 3–22. 10.1556/JBA.1.2012.1.1 26166826

[34] Rehbein F, Kleimann M, Mößle T. Prevalence and risk factors of video game dependency in adolescence: results of a German nationwide survey. Cyberpsychology Behav Soc Netw. 2010;13: 269–277. 10.1089/cyber.2009.0227 20557246

[35] Allison SE, von Wahlde L, Shockley T, Gabbard GO. The development of the self in the era of the internet and role-playing fantasy games. Am J Psychiatry. 2006;163: 381–385. 10.1176/appi.ajp.163.3.381 16513856

[36] Andreassen CS, Griffiths MD, Gjertsen SR, Krossbakken E, Kvam S, Pallesen S. The relationships between behavioral addictions and the five-factor model of personality. J Behav Addict. 2013;2: 90–99. Available: http://search.ebscohost.com/login.aspx?direct=true&db=psyh&AN=2013-30916-004&site=ehost-live&scope=site. 26165928 10.1556/JBA.2.2013.003

[37] Bouna-Pyrrou P, Mühle C, Kornhuber J, Lenz B. Internet gaming disorder, social network disorder and laterality: Handedness relates to pathological use of social networks. J Neural Transm. 2015;122: 1187–1196. 10.1007/s00702-014-1361-5 25576300 PMC4513228

[38] Brunborg GS, Mentzoni RA, Froyland LR. Is video gaming, or video game addiction, associated with depression, academic achievement, heavy episodic drinking, or conduct problems? J Behav Addict. 2014;3 10.1556/JBA.3.2014.002 25215212 PMC4117274

[39] Desai RA, Krishnan-Sarin S, Cavallo D, Potenza MN. Video-gaming among high school students: health correlates, gender differences, and problematic gaming. Pediatrics. 2010;126: e1414–1424. 10.1542/peds.2009-2706 21078729 PMC3678538

[40] Gentile DA, Choo H, Liau A, Sim T, Li D, Fung D, et al Pathological video game use among youths: a two-year longitudinal study. Pediatrics. 2011;127: e319–329. 10.1542/peds.2010-1353 21242221

[41] Jiménez-Murcia S, Fernández-Aranda F, Granero R, Chóliz M, La Verde M, Aguglia E, et al Video Game Addiction in Gambling Disorder: Clinical, Psychopathological, and Personality Correlates. BioMed Res Int. 2014;2014: 11 10.1155/2014/315062. PMC4122146 25126551

[42] Kim NR, Hwang SS-H, Choi J-S, Kim D-J, Demetrovics Z, Király O, et al Characteristics and Psychiatric Symptoms of Internet Gaming Disorder among Adults Using Self-Reported DSM-5 Criteria. Psychiatry Investig. 2015;13: 58–66. 10.4306/pi.2016.13.1.58 26766947 PMC4701686

[43] King DL, Delfabbro PH, Zwaans T, Kaptsis D. Clinical features and axis I comorbidity of Australian adolescent pathological Internet and video game users. Aust N Z J Psychiatry. 2013;47: 1058–1067. 10.1177/0004867413491159 23719181

[44] King DL, Delfabbro PH. The Cognitive Psychopathology of Internet Gaming Disorder in Adolescence. J Abnorm Child Psychol. 2016;44: 1635–1645. 10.1007/s10802-016-0135-y 26875565

[45] Király O, Griffiths MD, Urban R, Farkas J, Kokonyei G, Elekes Z, et al Problematic internet use and problematic online gaming are not the same: findings from a large nationally representative adolescent sample. Cyberpsychology Behav Soc Netw. 2014;17: 749–754. 10.1089/cyber.2014.0475 25415659 PMC4267705

[46] Laconi S, Pirès S, Chabrol H. Internet gaming disorder, motives, game genres and psychopathology. Comput Hum Behav. 2017;75: 652–659. 10.1016/j.chb.2017.06.012

[47] Lemos IL, Cardoso A, Sougey EB. Validity and reliability assessment of the Brazilian version of the game addiction scale (GAS). Compr Psychiatry. 2016;67: 19–25. 10.1016/j.comppsych.2016.01.014 27095330

[48] Li D, Liau A, Khoo A. Examining the influence of actual-ideal self-discrepancies, depression, and escapism, on pathological gaming among massively multiplayer online adolescent gamers. Cyberpsychology Behav Soc Netw. 2011;14: 535–539. 10.1089/cyber.2010.0463 21332374

[49] Männikkö N, Billieux J, Kaariainen M. Problematic digital gaming behavior and its relation to the psychological, social and physical health of Finnish adolescents and young adults. J Behav Addict. 2015;4: 281–288. 10.1556/2006.4.2015.040 26690623 PMC4712762

[50] Mentzoni RA, Brunborg GS, Molde H, Myrseth H, Mår Skouverøe KJ, Hetland J, et al Problematic video game use: Estimated prevalence and associations with mental and physical health. Cyberpsychology Behav Soc Netw. 2011;14: 591–596. Available: http://search.ebscohost.com/login.aspx?direct=true&db=psyh&AN=2011-24261-006&site=ehost-live&scope=site. 10.1089/cyber.2010.0260 21342010

[51] Müller KW, Janikian M, Dreier M, Wölfling K, Beutel ME, Tzavara C, et al Regular gaming behavior and internet gaming disorder in European adolescents: results from a cross-national representative survey of prevalence, predictors, and psychopathological correlates. Eur Child Adolesc Psychiatry. 2015;24: 565–574. 10.1007/s00787-014-0611-2 25189795

[52] Na E, Lee H, Choi I, Kim D-J. Comorbidity of Internet gaming disorder and alcohol use disorder: A focus on clinical characteristics and gaming patterns. Am J Addict. 2017;26: 326–334. 10.1111/ajad.12528 28328110

[53] Pápay O, Urbán R, Griffiths MD, Nagygyörgy K, Farkas J, Kökönyei G, et al Psychometric properties of the Problematic Online Gaming Questionnaire Short-Form and prevalence of problematic online gaming in a national sample of adolescents. Cyberpsychology Behav Soc Netw. 2013;16: 340–348. 10.1089/cyber.2012.0484 23621688 PMC3678653

[54] Peng W, Liu M. Online gaming dependency: a preliminary study in China. Cyberpsychology Behav Soc Netw. 2010;13: 329–333. 10.1089/cyber.2009.0082 20557254

[55] Starcevic V, Berle D, Porter G, Fenech P. Problem video game use and dimensions of psychopathology. Int J Ment Health Addict. 2011;9: 248–256. Available: http://search.ebscohost.com/login.aspx?direct=true&db=psyh&AN=2011-11090-003&site=ehost-live&scope=site.

[56] Van Rooij AJ, Schoenmakers TM, Van den Eijnden RJJM, Vermulst AA, Van de Mheen D. Video game addiction test: validity and psychometric characteristics. Cyberpsychology Behav Soc Netw. 2012;15: 507–511. 10.1089/cyber.2012.0007 22900926

[57] Vadlin S, Aslund C, Hellstrom C, Nilsson KW. Associations between problematic gaming and psychiatric symptoms among adolescents in two samples. Addict Behav. 2016;61: 8–15. 10.1016/j.addbeh.2016.05.001 27203825

[58] Vukosavljevic-Gvozden T, Filipovic S, Opacic G. The mediating role of symptoms of psychopathology between irrational beliefs and Internet gaming addiction. J Ration-Emotive Cogn-Behav Ther. 2015;33: 387–405. 10.1007/s10942-015-0218-7

[59] Colder Carras M, Kalbarczyk A, Wells K, Banks J, Kowert R, Gillespie C, et al Connection, meaning, and distraction: A qualitative study of video game play and mental health recovery in veterans treated for mental and/or behavioral health problems. Soc Sci Med 1982. 2018;216: 124–132. 10.1016/j.socscimed.2018.08.044 30257787 PMC6193255

[60] King DL, Delfabbro PH, Zajac IT. Preliminary validation of a new clinical tool for identifying problem video game playing. Int J Ment Health Addict. 2011;9: 72–87. Available: http://search.ebscohost.com/login.aspx?direct=true&db=psyh&AN=2011-02644-006&site=ehost-live&scope=site.

[61] Liu M, Peng W. Cognitive and Psychological Predictors of the Negative Outcomes Associated with Playing MMOGs (Massively Multiplayer Online Games). Comput Hum Behav. 2009;25: 1306–1311. 10.1016/j.chb.2009.06.002

[62] Topor DR, Swenson LP, Liguori GM, Spirito A, Lowenhaupt EA, Hunt JI. Problematic video game use scale: initial psychometric properties with psychiatrically hospitalized adolescents. J Clin Psychiatry. 2011;72: 1611–1615. 10.4088/JCP.11m07040 22244022

[63] Walther B, Morgenstern M, Hanewinkel R. Co-occurrence of addictive behaviours: Personality factors related to substance use, gambling and computer gaming. Eur Addict Res. 2012;18: 167–174. 10.1159/000335662 22398819

[64] Yu H, Cho J. Prevalence of Internet Gaming Disorder among Korean Adolescents and Associations with Non-psychotic Psychological Symptoms, and Physical Aggression. Am J Health Behav. 2016;40: 705–716. 10.5993/AJHB.40.6.3 27779939

[65] Hyun GJ, Han DH, Lee YS, Kang KD, Yoo SK, Chung U-S, et al Risk factors associated with online game addiction: A hierarchical model. Comput Hum Behav. 2015;48: 706–713. 10.1016/j.chb.2015.02.008

[66] Han DH, Lyoo IK, Renshaw PF. Differential regional gray matter volumes in patients with on-line game addiction and professional gamers. J Psychiatr Res. 2012;46: 507–515. 10.1016/j.jpsychires.2012.01.004 22277302 PMC4632992

[67] Kim SM, Han DH, Lee YS, Kim JE, Renshaw PF. Changes in brain activity in response to problem solving during the abstinence from online game play. J Behav Addict. 2012;1: 41–49. 10.1556/JBA.1.2012.2.1 26165305

[68] Han DH, Hwang JW, Renshaw PF. Bupropion sustained release treatment decreases craving for video games and cue-induced brain activity in patients with Internet video game addiction. Exp Clin Psychopharmacol. 2010;18: 297–304. 10.1037/a0020023 20695685

[69] Batthyány D, Muller KW, Benker F, Wolfling K. [Computer game playing: clinical characteristics of dependence and abuse among adolescents]. Wien Klin Wochenschr. 2009;121: 502–509. 10.1007/s00508-009-1198-3 19787320

[70] Kim MG, Kim J. Cross-validation of reliability, convergent and discriminant validity for the problematic online game use scale. Comput Hum Behav. 2010;26: 389–398. Available: http://search.ebscohost.com/login.aspx?direct=true&db=psyh&AN=2010-04134-016&site=ehost-live&scope=site.

[71] Kim N, Hughes TL, Park CG, Quinn L, Kong ID. Resting-State Peripheral Catecholamine and Anxiety Levels in Korean Male Adolescents with Internet Game Addiction. Cyberpsychology Behav Soc Netw. 2016;19: 202–208. 10.1089/cyber.2015.0411 26849530 PMC4799709

[72] Mehroof M, Griffiths MD. Online gaming addiction: The role of sensation seeking, self-control, neuroticism, aggression, state anxiety, and trait anxiety. Cyberpsychology Behav Soc Netw. 2010;13: 313–316. Available: http://search.ebscohost.com/login.aspx?direct=true&db=psyh&AN=2010-13062-011&site=ehost-live&scope=site. 10.1089/cyber.2009.0229 20557251

[73] Achab S, Nicolier M, Mauny F, Monnin J, Trojak B, Vandel P, et al Massively multiplayer online role-playing games: comparing characteristics of addict vs non-addict online recruited gamers in a French adult population. BMC Psychiatry. 2011;11 10.1186/1471-244X-11-144 21871089 PMC3176476

[74] Coyne SM, Dyer WJ, Densley R, Money NM, Day RD, Harper JM. Physiological indicators of pathologic video game use in adolescence. J Adolesc Health. 2015 10.1016/j.jadohealth.2014.10.271 25586229

[75] Tejeiro RA, Gómez-Vallecillo JL, Pelegrina M, Wallace A, Emberley E. Risk factors associated with the abuse of video games in adolescents. Psychology. 2012;3: 310–314. 10.4236/psych.2012.34044

[76] Zarin D, Tse T, Williams R, Califf R, Ide N. The ClinicalTrials.gov Results Database—Update and Key Issues. NEJM. 2011;364: 852–860. 10.1056/NEJMsa1012065 21366476 PMC3066456

[77] McLeroy KR, Garney W, Mayo-Wilson E, Grant S. Scientific Reporting: Raising the Standards. Health Educ Behav Off Publ Soc Public Health Educ. 2016;43: 501–508. 10.1177/1090198116668522 27624441

[78] Page MJ, McKenzie JE, Kirkham J, Dwan K, Kramer S, Green S, et al Bias due to selective inclusion and reporting of outcomes and analyses in systematic reviews of randomised trials of healthcare interventions. Cochrane Database Syst Rev. 2014; MR000035. 10.1002/14651858.MR000035.pub2 25271098 PMC8191366

[79] Higgins J, Green S, editors. Cochrane Handbook for Systematic Reviews of Interventions Version 5.1.0 [updated March 2011]. The Cochrane Collaboration; 2011. www.cochrane-handbook.org.

[80] Moher D, Jadad AR, Nichol G, Penman M, Tugwell P, Walsh S. Assessing the quality of randomized controlled trials: an annotated bibliography of scales and checklists. Control Clin Trials. 1995;16: 62–73. 10.1016/0197-2456(94)00031-w 7743790

[81] Huwiler-Müntener K, Jüni P, Junker C, Egger M. Quality of reporting of randomized trials as a measure of methodologic quality. JAMA. 2002;287: 2801–2804. 10.1001/jama.287.21.2801 12038917

[82] Higgins JPT, Altman DG, Gøtzsche PC, Jüni P, Moher D, Oxman AD, et al The Cochrane Collaboration’s tool for assessing risk of bias in randomised trials. BMJ. 2011;343: d5928 10.1136/bmj.d5928 22008217 PMC3196245

[83] Sterne JAC, Savović J, Page MJ, Elbers RG, Blencowe NS, Boutron I, et al RoB 2: a revised tool for assessing risk of bias in randomised trials. BMJ. 2019;366: l4898 10.1136/bmj.l4898 31462531

[84] Institute of Medicine (US) Committee on Standards for Systematic Reviews of Comparative Effectiveness Research. Finding What Works in Health Care: Standards for Systematic Reviews. Eden J, Levit L, Berg A, Morton S, editors. Washington (DC): National Academies Press (US); 2011 http://www.ncbi.nlm.nih.gov/books/NBK209518/. 24983062

[85] Brownson RC, Chriqui JF, Stamatakis KA. Understanding Evidence-Based Public Health Policy. Am J Public Health. 2009;99: 1576–1583. 10.2105/AJPH.2008.156224 19608941 PMC2724448

