# Supplementary material for: Evaluating the quality of evidence for gaming disorder: A summary of systematic reviews of associations between gaming disorder and depression or anxiety
Source: PLoS One. 2020 Oct 26;15(10):e0240032. doi: 10.1371/journal.pone.0240032 (PMC7588081; doi:10.1371/journal.pone.0240032)
Supplement: S1 Protocol — (PDF) [file pone.0240032.s002.pdf]

Evaluating the quality of evidence for internet gaming disorder and associations with depression or anxiety: a review of systematic reviews

Michelle Colder Carras, Gregory Hard, Jing Shi

Citation

Michelle Colder Carras, Gregory Hard, Jing Shi. Evaluating the quality of evidence for internet gaming disorder and associations with depression or anxiety: a review of systematic reviews. PROSPERO 2018 CRD42018090651 Available from: https://www.crd.york.ac.uk/prospero/display_record.php?ID=CRD42018090651

Review question

(1) Do systematic reviews of problematic gaming/Internet gaming disorder (PG/IGD) and its relationship with depression or anxiety meet quality standards for systematic reviews?

(2) Do systematic reviews of PG/IGD and relationships with depression or anxiety distinguish between problematic gaming/Internet gaming disorder and Internet addiction/problematic Internet use?

(3) Do systematic reviews of PG/IGD and relationships with depression or anxiety report outcomes selectively?

(4) What are the associations between PG/IGD and depression or anxiety?

Searches

We will search PubMed and PsycINFO for systematic reviews and meta-analyses that focus on Internet gaming disorder or problematic gaming (using broad search terms) and are published in English. See full search strategy in PubMed below.

Search strategy

https://www.crd.york.ac.uk/PROSPEROFILES/90651_STRATEGY_20180319.pdf

Types of study to be included

Systematic reviews and meta-analyses will be included if they examine the exposure (PG/IGD) and outcomes associated with depression or anxiety. If a review did not specify whether it was systematic, we defined a systematic review as a review that "aim[s] to identify, evaluate and summarise the findings of all relevant individual studies [and] ...adhere[s] to a strict scientific design based on explicit, pre-specified and reproducible methods."

Centre for Reviews and Dissemination. (2009). Systematic Reviews: CRD’s Guidance for Undertaking Reviews in Healthcare. York: Centre for Reviews & Dissemination.

Condition or domain being studied

Internet gaming disorder/gaming disorder (also known as problematic, pathological or compulsive gaming), has been described as a behavioral addiction or addictive disorder related to excessive video, computer, or online game play. The disorder includes symptoms related to addiction such as loss of control over gaming behavior, continuing despite negative consequences, and functional impairment, distress, and or interference with daily activities. The disorder is distinguished from disorders related to Internet use such as Internet addiction and social networking addiction. The disorder was included as an Emerging Measure in the Diagnostic and Statistical Manual 5 and has been proposed for inclusion in the World Health Organization's International Classification for Disease-11.

Participants/population

Any

Intervention(s), exposure(s)
The exposure in this review is Internet gaming disorder/problematic/pathological gaming.

Comparator(s)/control
N/A

Main outcome(s)
Evidence quality of systematic reviews was the primary outcome. Reviews were classified as “reliable” per Mayo-Wilson et al. (2017) when systematic review authors had (1) defined criteria for selecting studies, (2) conducted comprehensive literature searches for studies, (3) assessed methodological quality (risk of bias) of included studies, (4) used appropriate methods for meta-analysis (when meta-analytic results were reported), and (5) presented conclusions that were supported by the evidence provided in the review. In addition to the criteria given in Mayo-Wilson et al., we required that reviews define which outcomes from their eligible studies were included in the synthesis (e.g., outcomes from analyses that controlled for confounding vs. all analyses, etc) or report/synthesize all reported outcomes from each included study. Reviews were classified as “unreliable” when one or more of these criteria were not met.

Mayo-Wilson, E., Ng, S. M., Chuck, R. S., & Li, T. (2017). The quality of systematic reviews about interventions for refractive error can be improved: a review of systematic reviews. BMC Ophthalmology, 17(1), 164. https://doi.org/10.1186/s12886-017-0561-9

Additional outcome(s)
(1) Proportion of included studies within reviews that use scales, interviews or other assessments that are specific to PG/IGD (as opposed to including studies that also use scales/measurements that assess Internet addiction).

(2) Proportion of systematic reviews that specify how outcomes from included studies are included in the review.

(3) Description of how outcomes are reported for depression and anxiety within all studies of our included systematic reviews. All associations within a review will be reported and characterized as present and positive, present and negative, unclear, or absent and compared to the full list of outcomes reported in the included test cases.

(3) Associations between depression or anxiety and PG/IGD. For each included review, we will list included studies, measures of depression/anxiety within included studies, and conclusions about association between PG/IGD and depression/anxiety at the level of each review and across all reviews. We will analyze subgroups of outcomes to compare outcomes from univariate vs. multivariate analyses and from studies classified as reliable vs. all studies.

Data extraction (selection and coding)
We conducted an initial search in PubMed and PsycINFO in 2018 to establish search criteria. From an initial retrieval of titles and abstracts, two individuals identified systematic reviews related to PG/IGD and depression or anxiety. Disagreements at this stage were resolved through discussion and consensus. Two individuals retrieved full text of remaining articles and reviewed for eligibility. Again, disagreements at this stage were resolved through discussion and consensus.

A data abstraction form was developed using Microsoft Excel based on the form used in Mayo-Wilson et al. (2017). The form was supplemented with additional questions related to review of evidence quality for systematic reviews of epidemiological studies (Stroop et al., 2000) as well as questions specific to selective outcomes reporting and measurement of IGD/PG within studies. Except for outcomes related to anxiety and depression from individual empirical studies within systematic reviews, all data will be entered electronically onto the form separately by two authors. Extracted data will be compared and differences will be discussed and resolved through consensus. Data about depression and anxiety outcomes within each empirical study of our systematic reviews will be entered by MCC.

Data to be extracted includes objectives and conclusions from each review, whether authors pre-specified eligibility criteria; whether authors limited reviews to studies using scales of problematic gaming (rather than

internet addiction, e.g.); information about participants, sampling, analyses, controlling for confounding or conducting multivariate analyses; specific measures of depression and/or anxiety used; whether authors reported plans for deciding which outcomes to include; types and number of databases searched; whether and how authors assessed the risk of bias in included studies; whether authors reported how decisions were made about which studies to include and how to abstract data from studies; numbers and types of studies in reviews; subgroup comparisons within reviews (e.g., individuals classified as PG/IGD vs. those not having PG/IGD); associations between PG/IGD and depression/anxiety; number of associations/comparisons per review; how results were synthesized/combined; whether authors discussed limitations at the study, outcome and review level; and any financial or other interests associated with the review. The data abstraction form is currently posted on the OSF website and will be made public when a manuscript is submitted for publication.

Stroup, D. F., Berlin, J. A., Morton, S. C., Olkin, I., Williamson, G. D., Rennie, D., … Thacker, S. B. (2000). Meta-analysis of observational studies in epidemiology: a proposal for reporting. Meta-analysis Of Observational Studies in Epidemiology (MOOSE) group. JAMA: The Journal of the American Medical Association, 283(15), 2008–2012.

Risk of bias (quality) assessment

Risk of bias (quality assessment) will be determined by whether systematic review authors (1) defined criteria for selecting studies, (2) conducted comprehensive literature searches for studies, (3) defined which outcomes from eligible studies were included in the synthesis (e.g., those from full samples vs. subsamples; those in multivariate vs. univariate analyses, etc.) or reported all outcomes in the study, (4) assessed methodological quality (risk of bias) of included studies, (5) used appropriate methods for meta-analysis (when meta-analytic results were reported), and (6) presented conclusions that were supported by the evidence provided in the review. Reviews will be classified as “unreliable” when one or more of these criteria were not met.

Although the review will include the proportion of included empirical studies that assess PG/IGD with a scale/interview that is specific to video game play within each eligible review, the quality of individual studies within our eligible systematic reviews will not be assessed in this review.

Data about risk of bias will be entered electronically onto the form separately by two authors. Differences will be discussed and resolved through consensus.

Strategy for data synthesis

We will provide a narrative synthesis that describes the characteristics of included reviews and evaluation of their quality based on our pre-specified risk of bias assessment. We will include the number of included studies in the reviews (range, mean), the number of participants in the included reviews (range, mean), the types of populations included in the reviews, years of publication, assessment for risk of bias, and reasons for being classified as unreliable.

To assess secondary outcomes, we will (1) describe the number and proportion of instrument(s) used to measure IGD/PG vs. Internet addiction/problematic Internet use within each review, (2) the numbers and proportion of systematic reviews that specify how outcomes will be reported from individual studies within the review, (3) provide the number and proportion of outcomes for depression and anxiety found within each study of each systematic review , and (4) use a vote-counting approach to count the number of positive, negative and null associations between IGD/PG and depression or anxiety within each systematic review and across all reviews, with additional reports by (a) subgroups of univariate vs. multivariate associations and (b) reviews classified as reliable vs. all studies.

Analysis of subgroups or subsets

We will report results for the full sample of included reviews and compare to results in those classified as reliable. We will also report results for univariate analyses and compare to those for multivariate (controlled) analyses.

Contact details for further information
Michelle Colder Carras
michelle.carras@gmail.com

Organisational affiliation of the review
None

Review team members and their organisational affiliations
Dr Michelle Colder Carras. None
Mr Gregory Hard. Johns Hopkins Bloomberg School of Public Health
Ms Jing Shi. University of Toronto

Type and method of review
Review of reviews

Anticipated or actual start date
08 March 2018

Anticipated completion date
30 April 2018

Funding sources/sponsors
None

Conflicts of interest

Language
(there is not an English language summary)

Country
Canada, United States of America

Stage of review
Review Ongoing

Subject index terms status
Subject indexing assigned by CRD

Subject index terms
Anxiety; Anxiety Disorders; Depression; Depressive Disorder; Humans; Internet

Date of registration in PROSPERO
05 April 2018

Date of first submission
20 March 2018

Stage of review at time of this submission

Stage	Started	Completed
Preliminary searches	No	Yes
Piloting of the study selection process	No	Yes
Formal screening of search results against eligibility criteria	No	Yes
Data extraction	No	No
Risk of bias (quality) assessment	No	No
Data analysis	No	No

The record owner confirms that the information they have supplied for this submission is accurate and complete and they understand that deliberate provision of inaccurate information or omission of data may be construed as scientific misconduct.

The record owner confirms that they will update the status of the review when it is completed and will add publication details in due course.

Versions

05 April 2018
20 June 2018

PROSPERO
This information has been provided by the named contact for this review. CRD has accepted this information in good faith and registered the review in PROSPERO. The registrant confirms that the information supplied for this submission is accurate and complete. CRD bears no responsibility or liability for the content of this registration record, any associated files or external websites.
