# Supplementary material for: Evaluating the quality of evidence for gaming disorder: A summary of systematic reviews of associations between gaming disorder and depression or anxiety
Source: PLoS One. 2020 Oct 26;15(10):e0240032. doi: 10.1371/journal.pone.0240032 (PMC7588081; doi:10.1371/journal.pone.0240032)
Supplement: S1 Search Strategy — (PDF) [file pone.0240032.s003.pdf]

S2 Strategy

PubMed

Search	Query
#12	Search ("video games"[MeSH Terms]) OR #1
	[note that this does not change the number of citations retrieved from query #1]
#10	Search (#9 AND #8)
#9	Search english[Language]
#8	Search (#7 AND #3)
#7	Search (#6 OR #5 OR #4)
#6	Search systematic review[Publication Type]
#5	Search meta-analysis[Publication Type]
#4	Search review[Publication Type]
#3	Search (#1 AND #2)
#2	Search (Pathological OR problematic OR compulsive OR addiction OR dependence OR excessive OR disorder* OR "problem play")
#1	Search ("Internet gaming" OR videogaming OR videogames OR video-games OR "video games" OR "video game" OR "online game" OR "online games" OR "online gaming" OR "computer game" OR "computer games" OR "computer gaming" OR "electronic game")

PsycInfo

"meta analysis" OR "systematic review" OR review OR meta-analysis
AND

Gaming OR "Internet gaming" OR videogaming OR videogames OR video-games OR "video games" OR "video game" OR "online game" OR "online games" OR "online gaming" OR "computer game" OR "computer games" OR "computer gaming" OR "electronic game"
AND

Pathological OR problematic OR compulsive OR addiction OR dependence OR excessive OR disorder* OR "problem play"
