# Supplementary material for: Evaluating the quality of evidence for gaming disorder: A summary of systematic reviews of associations between gaming disorder and depression or anxiety
Source: PLoS One. 2020 Oct 26;15(10):e0240032. doi: 10.1371/journal.pone.0240032 (PMC7588081; doi:10.1371/journal.pone.0240032)
Supplement: S1 Output — (DOCX) [file pone.0240032.s006.docx]

------------------------------------------------------------------------------------------------------------------------------------

name: <unnamed>

log: /Users/michellecarras/Dropbox/Sys rev of sys rev Revisions/PLOS ONE submission/New submission July 2020/OSF additions/O

> utput.log

log type: text

opened on: 16 Jul 2020, 10:48:13

. import excel "/Users/michellecarras/Dropbox/Sys rev of sys rev Revisions/PLOS ONE submission/Revisions/New analysis/Cleaned databa

> se V4.xlsx", sheet("DataV4") firstrow clear

.

. ***generate unique identifier for study

. cap drop id

. gen id=_n

. list id study

+------------------------------+

| id study |

|------------------------------|

1. | 1 Achab_2011 |

2. | 2 Allison_2006 |

3. | 3 Andreass_Sch |

4. | 4 Baer _2012 |

5. | 5 Baer_2011 |

|------------------------------|

6. | 6 Batthya_2009 |

7. | 7 Bayrakt_2007 |

8. | 8 Beranuy_2010 |

9. | 9 Billieux_2011 |

10. | 10 Bioulac_2008 |

|------------------------------|

11. | 11 Bouna-P |

12. | 12 Brunborg_2014 |

13. | 13 Brunborg_2015 |

14. | 14 Caplan_2009 |

15. | 15 Chan&Rab_2006 |

|------------------------------|

16. | 16 Chang_2008 |

17. | 17 Chappell_2006 |

18. | 18 Charlt_2002 |

19. | 19 Charlt_2007 |

20. | 20 Charlt_2010 |

|------------------------------|

21. | 21 Chiou_2008 |

22. | 22 Chiu_2004 |

23. | 23 Choo_2010 |

24. | 24 Chou_2003 |

25. | 25 Chuang_2006 |

|------------------------------|

26. | 26 Chumbley_2006 |

27. | 27 Coeffec_2015 |

28. | 28 Collins_2012 |

29. | 29 Coyne_2015 |

30. | 30 Cultura_2002 |

|------------------------------|

31. | 31 Demetrovics_2012 |

32. | 32 Desai_2010 |

33. | 33 Dong_2011 |

34. | 34 Dreier_2016 |

35. | 35 Dworak_2007 |

|------------------------------|

36. | 36 Festl_2012 |

37. | 37 Gentile_2009 |

38. | 38 Gentile_2011 |

39. | 39 Griffiths&H |

40. | 40 Griffiths_2004 |

|------------------------------|

41. | 41 Griffiths_2010a_OG_add |

42. | 42 Griffiths_2010b_role |

43. | 43 Grusser_2005 |

44. | 44 Grusser_2007 |

45. | 45 Haagsma_2012 prev |

|------------------------------|

46. | 46 Haagsma_2013 TPB |

47. | 47 Han_Hwang_2010 |

48. | 48 Han_Lee_2007 |

49. | 49 Han_Lee_2009 |

50. | 50 Han_Lyoo_2012 |

|------------------------------|

51. | 51 Hart_2009 |

52. | 52 Hawi_2012 |

53. | 53 Henchoz_2016 |

54. | 54 Hoeft_2008 |

55. | 55 Hsue_2009 |

|------------------------------|

56. | 56 Hull_2013 |

57. | 57 Hussain_2009a_att |

58. | 58 Hussain_2009b_exc |

59. | 59 Hussain_2012 |

60. | 60 Jeong_2011 |

|------------------------------|

61. | 61 Johansson_2004 |

62. | 62 Khazaal_2008 |

63. | 63 Kim_2008 |

64. | 64 Kim_2010 |

65. | 65 Kim_K_2015 |

|------------------------------|

66. | 66 Kim_NH_2016 |

67. | 67 Kim_NR_2016 |

68. | 68 King_2009a_motiv |

69. | 69 King_2009b_under |

70. | 70 King_2010 |

|------------------------------|

71. | 71 King_2011 |

72. | 72 King_2013_Axis |

73. | 73 King_2013 traj |

74. | 74 Kiraly_2014 |

75. | 75 Kiraly_2015 |

|------------------------------|

76. | 76 Ko_2005a |

77. | 77 Ko_2009 |

78. | 78 Konkoly_2015 |

79. | 79 Kwon_2009 |

80. | 80 Kwon_2011 |

|------------------------------|

81. | 81 Lafreniere_2009 |

82. | 82 Lee_2007 |

83. | 83 Lee_2015 |

84. | 84 Lehenbau_2015 |

85. | 85 Lemmens_2006 |

|------------------------------|

86. | 86 Lemmens_2009 |

87. | 87 Lemmens_2011_agg |

88. | 88 Lemmens_2011_psyc |

89. | 89 Lemmens_2015 |

90. | 90 Lemos_2016 |

|------------------------------|

91. | 91 Leung_2004 |

92. | 92 Li_2011 |

93. | 93 Liu_2009 |

94. | 94 Lopez-F_2013 |

95. | 95 Lu_2008 |

|------------------------------|

96. | 96 Manniko_2015 |

97. | 97 Meerkerk_2006 |

98. | 98 Meerkerk_2010 |

99. | 99 Mehroof_2010 |

100. | 100 Mentzoni_2011 |

|------------------------------|

101. | 101 Metcalf_2011 |

102. | 102 Montag_2011 |

103. | 103 Mottram_2009 |

104. | 104 Muller_2015 |

105. | 105 Ng_2005 |

|------------------------------|

106. | 106 Papay_2013 |

107. | 107 Parker_2008 |

108. | 108 Pawlikowski_2011 |

109. | 109 Peng_2010 |

110. | 110 Peters_2008 |

|------------------------------|

111. | 111 Pontes_2014 |

112. | 112 Porter_2010 |

113. | 113 Rasmussen_2015 |

114. | 114 Rau_2006 |

115. | 115 Ream_2011 |

|------------------------------|

116. | 116 Rehbein_2010 |

117. | 117 Rehbein_2013 |

118. | 118 Rehbein_2015 |

119. | 119 Rikkers_2016 |

120. | 120 Salguero_2002 |

|------------------------------|

121. | 121 Scharkow_2014 |

122. | 122 Schmitt_2015 |

123. | 123 Seah_2007 |

124. | 124 Seok_2012 |

125. | 125 Skoric_2009 |

|------------------------------|

126. | 126 Smahel_2008 |

127. | 127 Son_2013 |

128. | 128 Starcevic_2011 |

129. | 129 Stetina_2011 |

130. | 130 Strittmat_2015 |

|------------------------------|

131. | 131 Sun_2008 |

132. | 132 Tejeiro_2012 |

133. | 133 Thalemann_2004 |

134. | 134 Thalemann_2007 |

135. | 135 Thomas_2010 |

|------------------------------|

136. | 136 Tolchinsky_2011 |

137. | 137 Topor_2011 |

138. | 138 Tsitsika_2011 |

139. | 139 Turner_2012 |

140. | 140 Vadlin_2015 |

|------------------------------|

141. | 141 VRooij_2010_R |

142. | 142 VRooij_2011 |

143. | 143 VRooij_2012 |

144. | 144 Vukosc-G_2015 |

145. | 145 Walther_2012 |

|------------------------------|

146. | 146 Wan_2006a psych |

147. | 147 Wan_2006b why |

148. | 148 Wan_Chiou_2007 |

149. | 149 Wang_2014 |

150. | 150 Wang_2015 |

|------------------------------|

151. | 151 Wittek_2016 |

152. | 152 Wood_2007 |

153. | 153 Wu_2013 |

154. | 154 Yee_2006a_dem |

155. | 155 Yee_2006b_psych |

|------------------------------|

156. | 156 Yu_2015 |

157. | 157 Zhou_2009 |

158. | 158 Cole_2013 |

159. | 159 Jimenez_2014 |

160. | 160 King_2016 |

|------------------------------|

161. | 161 Laconi_2017 |

162. | 162 Na_2017 |

163. | 163 Panagiot_2017 |

164. | 164 Hyun_2015 |

165. | 165 Yen_2016 |

|------------------------------|

166. | 166 Vadlin_5/2016 |

167. | 167 Wang_2018 |

168. | 168 Wartbg_2017_IGD |

169. | 169 Wei_2012 |

170. | 170 Bae_2016 |

|------------------------------|

171. | 171 Bonnaire_2017 |

172. | 172 Cai_2016 |

173. | 173 Chun_2015 |

174. | 174 Colder_carr_2018 |

175. | 175 Ding_2013 |

|------------------------------|

176. | 176 Ding_2014 |

177. | 177 Du_2016 |

178. | 178 Feng_2013 |

179. | 179 Han_2017 |

180. | 180 Han_dh_2012 |

|------------------------------|

181. | 181 Hong_2015 |

182. | 182 Hu_2017 |

183. | 183 Jin_2016 |

184. | 184 Kim_N_2016 |

185. | 185 Kim_SM_2012 |

|------------------------------|

186. | 186 Park_2015 |

187. | 187 Park_2016 |

188. | 188 Park_2017 |

189. | 189 Pontes_2016 |

190. | 190 Qi_2015 |

|------------------------------|

191. | 191 Sakuma_2016 |

192. | 192 Wang_2015_al |

193. | 193 Wang_2015_dec |

194. | 194 Wartbg_2017 Assoc |

195. | 195 Wong_2016 |

|------------------------------|

196. | 196 Xing |

197. | 197 Yu_2016 |

+------------------------------+

.

.

. save "/Users/michellecarras/Dropbox/Sys rev of sys rev Revisions/PLOS ONE submission/New submission July 2020/OSF additions/Databa

> se.dta", replace

(note: file /Users/michellecarras/Dropbox/Sys rev of sys rev Revisions/PLOS ONE submission/New submission July 2020/OSF additions/Da

> tabase.dta not found)

file /Users/michellecarras/Dropbox/Sys rev of sys rev Revisions/PLOS ONE submission/New submission July 2020/OSF additions/Database.

> dta saved

.

.

. *****************************************************************************

. *

> *

. * SECTION 2

> *

. * DATA VALIDATION AND CLEANING

> *

. *

> *

. *****************************************************************************

. summ

Variable | Obs Mean Std. Dev. Min Max

-------------+--------------------------------------------------------

study | 0

notes | 0

GAclinpop | 196 .2653061 .7239637 0 3

pgscale | 0

ages | 0

-------------+--------------------------------------------------------

sampsi | 0

lang | 0

case | 0

exper | 0

neuro | 0

-------------+--------------------------------------------------------

long6 | 0

longit | 0

inmihar | 0

elmiha | 43 .5116279 .5057805 0 1

txtexcmih | 0

-------------+--------------------------------------------------------

inking | 0

elkig | 43 .3488372 .4822428 0 1

txtexck | 0

inmann | 0

elmann | 43 .744186 .4414814 0 1

-------------+--------------------------------------------------------

txtexclman | 0

inkuon | 0

elkuon | 43 .2093023 .4116251 0 1

txtsxckuon | 0

inkuin | 0

-------------+--------------------------------------------------------

elkuin | 43 .2325581 .4274626 0 1

txtexclkuin | 0

ingonz | 0

elgonz | 42 .8095238 .3974366 0 1

txtexcgon | 0

-------------+--------------------------------------------------------

insug | 0

elsug | 43 .4883721 .5057805 0 1

txtexcsug | 0

totEL | 43 3.27907 1.485254 1 7

wasit_PG | 194 .6752577 .5507472 0 2

-------------+--------------------------------------------------------

PGscaleYN | 0

year | 197 2011.299 3.894875 1998 2018

PGdepyesno | 195 .4769231 .8870377 0 4

PGanxyesno | 194 .4278351 .862247 0 3

depmu1txt | 0

-------------+--------------------------------------------------------

depmu1vote | 18 .6666667 .4850713 0 1

depmu2txt | 0

depmu2vote | 6 .8333333 .4082483 0 1

depmu3txt | 0

depmu3vote | 2 1 0 1 1

-------------+--------------------------------------------------------

depmu4txt | 0

depmu4vote | 1 1 . 1 1

anxmu1txt | 0

anxmu1vote | 13 .3846154 .6504436 -1 1

anxmu2txt | 0

-------------+--------------------------------------------------------

anxmu2vote | 8 .25 .7071068 -1 1

anxmu3txt | 0

anxmu3vote | 3 .6666667 .5773503 0 1

anxmu4txt | 0

anxmu4vote | 2 .5 .7071068 0 1

-------------+--------------------------------------------------------

anxmu5txt | 0

anxmu5vote | 1 1 . 1 1

anxmu6txt | 0

anxmu6vote | 1 1 . 1 1

anxmu7txt | 0

-------------+--------------------------------------------------------

anxmu7vote | 1 1 . 1 1

anxmu8txt | 0

anxmu8vote | 1 1 . 1 1

anxmu9txt | 0

anxmu9vote | 1 1 . 1 1

-------------+--------------------------------------------------------

depbi1txt | 0

depbi1vote | 42 .7142857 .45723 0 1

depbi2txt | 0

depbi2vote | 16 .875 .341565 0 1

depbi3txt | 0

-------------+--------------------------------------------------------

depbi3vote | 4 1 0 1 1

depbi4txt | 0

depbi4vote | 1 0 . 0 0

depbi5txt | 0

depbi5vote | 1 0 . 0 0

-------------+--------------------------------------------------------

depbi6txt | 0

depbi6vote | 1 0 . 0 0

depbi7txt | 0

depbi7vote | 1 1 . 1 1

depbi8txt | 0

-------------+--------------------------------------------------------

depbi8vote | 1 0 . 0 0

depbi9txt | 0

depbi9vote | 0

anxbi1txt | 0

anxbi1vote | 34 .7647059 .4305615 0 1

-------------+--------------------------------------------------------

anxbi2txt | 0

anxbi2vote | 19 .6842105 .4775669 0 1

anxbi3txt | 0

anxbi3vote | 9 .6666667 .5 0 1

anxbi4txt | 0

-------------+--------------------------------------------------------

anxbi4vote | 5 .4 .5477226 0 1

anxbi5txt | 0

anxbi5vote | 3 .3333333 .5773503 0 1

anxbi6txt | 0

anxbi6vote | 2 .5 .7071068 0 1

-------------+--------------------------------------------------------

anxbi7txt | 0

anxbi7vote | 1 0 . 0 0

anxbi8txt | 0

anxbi8vote | 1 0 . 0 0

anxbi9txt | 0

-------------+--------------------------------------------------------

anxbi9vote | 1 0 . 0 0

Ldepbi1txt | 0

Ldepbi1vote | 1 0 . 0 0

Ldepbi2txt | 0

Ldepbi2vote | 1 0 . 0 0

-------------+--------------------------------------------------------

Ldepbi3txt | 0

Ldepbi3vote | 0

Ldepbi4txt | 0

Ldepbi4vote | 0

Ldepbi5txt | 0

-------------+--------------------------------------------------------

Ldepbi5vote | 0

Ldepbi6txt | 0

Ldepbi6vote | 0

Lanxbi1txt | 0

Lanxbi1vote | 0

-------------+--------------------------------------------------------

Lanxbi2txt | 0

Lanxbi2vote | 0

Lanxbi3txt | 0

Lanxbi3vote | 0

Lanxbi4txt | 0

-------------+--------------------------------------------------------

Lanxbi4vote | 0

Lanxbi5txt | 0

Lanxbi5vote | 0

Lanxbi6txt | 0

Lanxbi6vote | 0

-------------+--------------------------------------------------------

Ldepmu1txt | 0

Ldepmu1vote | 2 1 0 1 1

Ldepmu2txt | 0

Ldepmu2vote | 1 1 . 1 1

Ldepmu3txt | 0

-------------+--------------------------------------------------------

Ldepmu3vote | 1 1 . 1 1

Ldepmu4txt | 0

Ldepmu4vote | 1 1 . 1 1

Ldepmu5txt | 0

Ldepmu5vote | 0

-------------+--------------------------------------------------------

Lanxmu1txt | 0

Lanxmu1vote | 1 1 . 1 1

Lanxmu2txt | 0

Lanxmu2vote | 1 1 . 1 1

Lanxmu3txt | 0

-------------+--------------------------------------------------------

Lanxmu3vote | 1 1 . 1 1

Lanxmu4txt | 0

Lanxmu4vote | 1 1 . 1 1

Lanxmu5txt | 0

Lanxmu5vote | 1 1 . 1 1

-------------+--------------------------------------------------------

Lanxmu6txt | 0

Lanxmu6vote | 1 1 . 1 1

Lanxmu7txt | 0

Lanxmu7vote | 1 1 . 1 1

Lanxmu8txt | 0

-------------+--------------------------------------------------------

Lanxmu8vote | 1 1 . 1 1

Lanxmu9txt | 0

Lanxmu9vote | 1 1 . 1 1

Lanxmu10txt | 0

Lanxmu10vote | 1 1 . 1 1

-------------+--------------------------------------------------------

Lanxmu11txt | 0

Lanxmu11vote | 1 1 . 1 1

id | 197 99 57.01316 1 197

.

. codebook, compact

Variable Obs Unique Mean Min Max Label

------------------------------------------------------------------------------------------------------------------------------------

study 197 197 . . . study

notes 57 52 . . . notes

GAclinpop 196 4 .2653061 0 3 GA clin pop

pgscale 197 128 . . . pgscale

ages 43 42 . . . ages

sampsi 43 42 . . . sampsi

lang 43 2 . . . lang

case 43 3 . . . case

exper 43 2 . . . exper

neuro 43 4 . . . neuro

long6 194 4 . . . long6+

longit 194 2 . . . longit

inmihar 196 2 . . . inmihar

elmiha 43 2 .5116279 0 1 elmiha

txtexcmih 20 5 . . . txtexcmih

inking 196 2 . . . inking

elkig 43 2 .3488372 0 1 elkig

txtexck 28 5 . . . txtexck

inmann 196 2 . . . inmann

elmann 43 2 .744186 0 1 elmann

txtexclman 11 6 . . . txtexclman

inkuon 196 2 . . . inkuon

elkuon 43 2 .2093023 0 1 elkuon

txtsxckuon 34 2 . . . txtsxckuon

inkuin 197 2 . . . inkuin

elkuin 43 2 .2325581 0 1 elkuin

txtexclkuin 33 1 . . . txtexclkuin

ingonz 197 2 . . . ingonz

elgonz 42 2 .8095238 0 1 elgonz

txtexcgon 8 4 . . . txtexcgon

insug 197 2 . . . insug

elsug 43 2 .4883721 0 1 elsug

txtexcsug 22 4 . . . txtexcsug

totEL 43 6 3.27907 1 7 totEL

wasit_PG 194 3 .6752577 0 2 wasit_PG

PGscaleYN 194 3 . . . PGscaleYN

year 197 18 2011.299 1998 2018 year

PGdepyesno 195 5 .4769231 0 4 PGdepyesno

PGanxyesno 194 4 .4278351 0 3 PGanxyesno

depmu1txt 18 18 . . . depmu1txt

depmu1vote 18 2 .6666667 0 1 depmu1vote

depmu2txt 6 6 . . . depmu2txt

depmu2vote 6 2 .8333333 0 1 depmu2vote

depmu3txt 2 2 . . . depmu3txt

depmu3vote 2 1 1 1 1 depmu3vote

depmu4txt 1 1 . . . depmu4txt

depmu4vote 1 1 1 1 1 depmu4vote

anxmu1txt 13 13 . . . anxmu1txt

anxmu1vote 13 3 .3846154 -1 1 anxmu1vote

anxmu2txt 8 8 . . . anxmu2txt

anxmu2vote 8 3 .25 -1 1 anxmu2vote

anxmu3txt 3 3 . . . anxmu3txt

anxmu3vote 3 2 .6666667 0 1 anxmu3vote

anxmu4txt 2 2 . . . anxmu4txt

anxmu4vote 2 2 .5 0 1 anxmu4vote

anxmu5txt 1 1 . . . anxmu5txt

anxmu5vote 1 1 1 1 1 anxmu5vote

anxmu6txt 1 1 . . . anxmu6txt

anxmu6vote 1 1 1 1 1 anxmu6vote

anxmu7txt 1 1 . . . anxmu7txt

anxmu7vote 1 1 1 1 1 anxmu7vote

anxmu8txt 1 1 . . . anxmu8txt

anxmu8vote 1 1 1 1 1 anxmu8vote

anxmu9txt 1 1 . . . anxmu9txt

anxmu9vote 1 1 1 1 1 anxmu9vote

depbi1txt 43 36 . . . depbi1txt

depbi1vote 42 2 .7142857 0 1 depbi1vote

depbi2txt 16 15 . . . depbi2txt

depbi2vote 16 2 .875 0 1 depbi2vote

depbi3txt 4 4 . . . depbi3txt

depbi3vote 4 1 1 1 1 depbi3vote

depbi4txt 1 1 . . . depbi4txt

depbi4vote 1 1 0 0 0 depbi4vote

depbi5txt 1 1 . . . depbi5txt

depbi5vote 1 1 0 0 0 depbi5vote

depbi6txt 1 1 . . . depbi6txt

depbi6vote 1 1 0 0 0 depbi6vote

depbi7txt 1 1 . . . depbi7txt

depbi7vote 1 1 1 1 1 depbi7vote

depbi8txt 1 1 . . . depbi8txt

depbi8vote 1 1 0 0 0 depbi8vote

depbi9txt 0 0 . . . depbi9txt

depbi9vote 0 0 . . . depbi9vote

anxbi1txt 35 31 . . . anxbi1txt

anxbi1vote 34 2 .7647059 0 1 anxbi1vote

anxbi2txt 19 19 . . . anxbi2txt

anxbi2vote 19 2 .6842105 0 1 anxbi2vote

anxbi3txt 9 9 . . . anxbi3txt

anxbi3vote 9 2 .6666667 0 1 anxbi3vote

anxbi4txt 5 5 . . . anxbi4txt

anxbi4vote 5 2 .4 0 1 anxbi4vote

anxbi5txt 3 3 . . . anxbi5txt

anxbi5vote 3 2 .3333333 0 1 anxbi5vote

anxbi6txt 2 2 . . . anxbi6txt

anxbi6vote 2 2 .5 0 1 anxbi6vote

anxbi7txt 1 1 . . . anxbi7txt

anxbi7vote 1 1 0 0 0 anxbi7vote

anxbi8txt 1 1 . . . anxbi8txt

anxbi8vote 1 1 0 0 0 anxbi8vote

anxbi9txt 1 1 . . . anxbi9txt

anxbi9vote 1 1 0 0 0 anxbi9vote

Ldepbi1txt 1 1 . . . Ldepbi1txt

Ldepbi1vote 1 1 0 0 0 Ldepbi1vote

Ldepbi2txt 1 1 . . . Ldepbi2txt

Ldepbi2vote 1 1 0 0 0 Ldepbi2vote

Ldepbi3txt 0 0 . . . Ldepbi3txt

Ldepbi3vote 0 0 . . . Ldepbi3vote

Ldepbi4txt 0 0 . . . Ldepbi4txt

Ldepbi4vote 0 0 . . . Ldepbi4vote

Ldepbi5txt 0 0 . . . Ldepbi5txt

Ldepbi5vote 0 0 . . . Ldepbi5vote

Ldepbi6txt 0 0 . . . Ldepbi6txt

Ldepbi6vote 0 0 . . . Ldepbi6vote

Lanxbi1txt 0 0 . . . Lanxbi1txt

Lanxbi1vote 0 0 . . . Lanxbi1vote

Lanxbi2txt 0 0 . . . Lanxbi2txt

Lanxbi2vote 0 0 . . . Lanxbi2vote

Lanxbi3txt 0 0 . . . Lanxbi3txt

Lanxbi3vote 0 0 . . . Lanxbi3vote

Lanxbi4txt 0 0 . . . Lanxbi4txt

Lanxbi4vote 0 0 . . . Lanxbi4vote

Lanxbi5txt 0 0 . . . Lanxbi5txt

Lanxbi5vote 0 0 . . . Lanxbi5vote

Lanxbi6txt 0 0 . . . Lanxbi6txt

Lanxbi6vote 0 0 . . . Lanxbi6vote

Ldepmu1txt 2 2 . . . Ldepmu1txt

Ldepmu1vote 2 1 1 1 1 Ldepmu1vote

Ldepmu2txt 1 1 . . . Ldepmu2txt

Ldepmu2vote 1 1 1 1 1 Ldepmu2vote

Ldepmu3txt 1 1 . . . Ldepmu3txt

Ldepmu3vote 1 1 1 1 1 Ldepmu3vote

Ldepmu4txt 1 1 . . . Ldepmu4txt

Ldepmu4vote 1 1 1 1 1 Ldepmu4vote

Ldepmu5txt 0 0 . . . Ldepmu5txt

Ldepmu5vote 0 0 . . . Ldepmu5vote

Lanxmu1txt 1 1 . . . Lanxmu1txt

Lanxmu1vote 1 1 1 1 1 Lanxmu1vote

Lanxmu2txt 1 1 . . . Lanxmu2txt

Lanxmu2vote 1 1 1 1 1 Lanxmu2vote

Lanxmu3txt 1 1 . . . Lanxmu3txt

Lanxmu3vote 1 1 1 1 1 Lanxmu3vote

Lanxmu4txt 1 1 . . . Lanxmu4txt

Lanxmu4vote 1 1 1 1 1 Lanxmu4vote

Lanxmu5txt 1 1 . . . Lanxmu5txt

Lanxmu5vote 1 1 1 1 1 Lanxmu5vote

Lanxmu6txt 1 1 . . . Lanxmu6txt

Lanxmu6vote 1 1 1 1 1 Lanxmu6vote

Lanxmu7txt 1 1 . . . Lanxmu7txt

Lanxmu7vote 1 1 1 1 1 Lanxmu7vote

Lanxmu8txt 1 1 . . . Lanxmu8txt

Lanxmu8vote 1 1 1 1 1 Lanxmu8vote

Lanxmu9txt 1 1 . . . Lanxmu9txt

Lanxmu9vote 1 1 1 1 1 Lanxmu9vote

Lanxmu10txt 1 1 . . . Lanxmu10txt

Lanxmu10vote 1 1 1 1 1 Lanxmu10vote

Lanxmu11txt 1 1 . . . Lanxmu11txt

Lanxmu11vote 1 1 1 1 1 Lanxmu11vote

id 197 197 99 1 197

------------------------------------------------------------------------------------------------------------------------------------

. lab var GAclinpop "Study recruited clinical population-IGD or otherwise"

. lab def clinpop 0 "0_No" 1 "1_IGD pop or mixed" 2 "2_clin, not IGD" 3 "3_unclear"

. lab values GAclinpop clinpop

. codebook GAclinpop

------------------------------------------------------------------------------------------------------------------------------------

GAclinpop Study recruited clinical population-IGD or otherwise

------------------------------------------------------------------------------------------------------------------------------------

type: numeric (byte)

label: clinpop

range: [0,3] units: 1

unique values: 4 missing .: 1/197

tabulation: Freq. Numeric Label

168 0 0_No

12 1 1_IGD pop or mixed

8 2 2_clin, not IGD

8 3 3_unclear

1 .

.

. foreach var in elgonz elkig elsug elmann elmiha elkuin elkuon {

2. lab var `var' "eligible for review and dep/anx findings"

3. }

.

.

. codebook PGdepyesno

------------------------------------------------------------------------------------------------------------------------------------

PGdepyesno PGdepyesno

------------------------------------------------------------------------------------------------------------------------------------

type: numeric (byte)

range: [0,4] units: 1

unique values: 5 missing .: 2/197

tabulation: Freq. Value

137 0

39 1

4 2

14 3

1 4

2 .

. codebook PGanxyesno

------------------------------------------------------------------------------------------------------------------------------------

PGanxyesno PGanxyesno

------------------------------------------------------------------------------------------------------------------------------------

type: numeric (byte)

range: [0,3] units: 1

unique values: 4 missing .: 3/197

tabulation: Freq. Value

144 0

32 1

3 2

15 3

3 .

. list study id if PGdepyesno==4

+--------------------+

| study id |

|--------------------|

129. | Stetina_2011 129 |

+--------------------+

.

. ***Stettina has the same problem as the "nonspecifics" - doesn't measure depression

. *****and PG but rather depression and MMORPGs

. replace PGdepyesno=3 if id==129

(1 real change made)

. lab def yesnoPG 0 "0_No" 1 "1_Yes" 2 "2_both PG & PIU" 3 "3_nonspecific" 4 "4_psych but not PG", replace

. note PGdepyesno: Nonspecific (value 3) means dep measure or 'PG' meas was nonspecific"

. lab values PGanxyesno yesnoPG

. lab values PGdepyesno yesnoPG

. codebook PG*

------------------------------------------------------------------------------------------------------------------------------------

PGscaleYN PGscaleYN

------------------------------------------------------------------------------------------------------------------------------------

type: string (str10)

unique values: 3 missing "": 3/197

tabulation: Freq. Value

3 ""

71 "0_No"

114 "1_Yes"

9 "2_PG+other"

------------------------------------------------------------------------------------------------------------------------------------

PGdepyesno PGdepyesno

------------------------------------------------------------------------------------------------------------------------------------

type: numeric (byte)

label: yesnoPG

range: [0,3] units: 1

unique values: 4 missing .: 2/197

tabulation: Freq. Numeric Label

137 0 0_No

39 1 1_Yes

4 2 2_both PG & PIU

15 3 3_nonspecific

2 .

------------------------------------------------------------------------------------------------------------------------------------

PGanxyesno PGanxyesno

------------------------------------------------------------------------------------------------------------------------------------

type: numeric (byte)

label: yesnoPG

range: [0,3] units: 1

unique values: 4 missing .: 3/197

tabulation: Freq. Numeric Label

144 0 0_No

32 1 1_Yes

3 2 2_both PG & PIU

15 3 3_nonspecific

3 .

. lab var PGdepyesno "Did the study assoc. PG/IA & depr?"

. lab var PGanxyesno "Did the study assoc. PG/IA & anx?"

. ***

. *****June 9-the PGdep/anxyesno value of 3 is confusing. Make a new var that is the "old"

. ***var and change the orig var to have the nonspecific "3" now be 0

. ****JUNE 11 snd the value 2 both PG ad PIU =1

. ****

. cap drop old_PGdepyesno

. clonevar old_PGdepyesno=PGdepyesno

(2 missing values generated)

. lab var PGdepyesno "New PGdep without 3 or2 "

. recode PGdepyesno 3=0

(PGdepyesno: 15 changes made)

. recode PGdepyesno 2=1

(PGdepyesno: 4 changes made)

. codebook old_PGdepyesno PGdepyesno

------------------------------------------------------------------------------------------------------------------------------------

old_PGdepyesno Did the study assoc. PG/IA & depr?

------------------------------------------------------------------------------------------------------------------------------------

type: numeric (byte)

label: yesnoPG

range: [0,3] units: 1

unique values: 4 missing .: 2/197

tabulation: Freq. Numeric Label

137 0 0_No

39 1 1_Yes

4 2 2_both PG & PIU

15 3 3_nonspecific

2 .

------------------------------------------------------------------------------------------------------------------------------------

PGdepyesno New PGdep without 3 or2

------------------------------------------------------------------------------------------------------------------------------------

type: numeric (byte)

label: yesnoPG

range: [0,1] units: 1

unique values: 2 missing .: 2/197

tabulation: Freq. Numeric Label

152 0 0_No

43 1 1_Yes

2 .

. tab old_PGdepyesno PGdepyesno, mi

Did the study |

assoc. PG/IA & | New PGdep without 3 or2

depr? | 0_No 1_Yes . | Total

-------------------+---------------------------------+----------

0_No | 137 0 0 | 137

1_Yes | 0 39 0 | 39

2_both PG & PIU | 0 4 0 | 4

3_nonspecific | 15 0 0 | 15

. | 0 0 2 | 2

-------------------+---------------------------------+----------

Total | 152 43 2 | 197

.

. cap drop old_PGanxyesno

. clonevar old_PGanxyesno=PGanxyesno

(3 missing values generated)

. lab var PGanxyesno "New PGanx without 3 or 2"

. recode PGanxyesno 3=0

(PGanxyesno: 15 changes made)

. recode PGanxyesno 2=1

(PGanxyesno: 3 changes made)

. codebook old_PGanxyesno PGanxyesno

------------------------------------------------------------------------------------------------------------------------------------

old_PGanxyesno Did the study assoc. PG/IA & anx?

------------------------------------------------------------------------------------------------------------------------------------

type: numeric (byte)

label: yesnoPG

range: [0,3] units: 1

unique values: 4 missing .: 3/197

tabulation: Freq. Numeric Label

144 0 0_No

32 1 1_Yes

3 2 2_both PG & PIU

15 3 3_nonspecific

3 .

------------------------------------------------------------------------------------------------------------------------------------

PGanxyesno New PGanx without 3 or 2

------------------------------------------------------------------------------------------------------------------------------------

type: numeric (byte)

label: yesnoPG

range: [0,1] units: 1

unique values: 2 missing .: 3/197

tabulation: Freq. Numeric Label

159 0 0_No

35 1 1_Yes

3 .

. tab old_PGanxyesno PGanxyesno, mi

Did the study |

assoc. PG/IA & | New PGanx without 3 or 2

anx? | 0_No 1_Yes . | Total

-------------------+---------------------------------+----------

0_No | 144 0 0 | 144

1_Yes | 0 32 0 | 32

2_both PG & PIU | 0 3 0 | 3

3_nonspecific | 15 0 0 | 15

. | 0 0 3 | 3

-------------------+---------------------------------+----------

Total | 159 35 3 | 197

.

.

. *change the text variables to string

. tostring *1txt *2txt *3txt *4txt *5txt *6txt *txt, replace

depmu1txt already string; no replace

anxmu1txt already string; no replace

depbi1txt already string; no replace

anxbi1txt already string; no replace

Ldepbi1txt already string; no replace

Lanxbi1txt was byte now str1

Ldepmu1txt already string; no replace

Lanxmu1txt already string; no replace

Lanxmu11txt already string; no replace

depmu2txt already string; no replace

anxmu2txt already string; no replace

depbi2txt already string; no replace

anxbi2txt already string; no replace

Ldepbi2txt already string; no replace

Lanxbi2txt was byte now str1

Ldepmu2txt already string; no replace

Lanxmu2txt already string; no replace

depmu3txt already string; no replace

anxmu3txt already string; no replace

depbi3txt already string; no replace

anxbi3txt already string; no replace

Ldepbi3txt was byte now str1

Lanxbi3txt was byte now str1

Ldepmu3txt already string; no replace

Lanxmu3txt already string; no replace

depmu4txt already string; no replace

anxmu4txt already string; no replace

depbi4txt already string; no replace

anxbi4txt already string; no replace

Ldepbi4txt was byte now str1

Lanxbi4txt was byte now str1

Ldepmu4txt already string; no replace

Lanxmu4txt already string; no replace

anxmu5txt already string; no replace

depbi5txt already string; no replace

anxbi5txt already string; no replace

Ldepbi5txt was byte now str1

Lanxbi5txt was byte now str1

Ldepmu5txt was byte now str1

Lanxmu5txt already string; no replace

anxmu6txt already string; no replace

depbi6txt already string; no replace

anxbi6txt already string; no replace

Ldepbi6txt was byte now str1

Lanxbi6txt was byte now str1

Lanxmu6txt already string; no replace

depmu1txt already string; no replace

depmu2txt already string; no replace

depmu3txt already string; no replace

depmu4txt already string; no replace

anxmu1txt already string; no replace

anxmu2txt already string; no replace

anxmu3txt already string; no replace

anxmu4txt already string; no replace

anxmu5txt already string; no replace

anxmu6txt already string; no replace

anxmu7txt already string; no replace

anxmu8txt already string; no replace

anxmu9txt already string; no replace

depbi1txt already string; no replace

depbi2txt already string; no replace

depbi3txt already string; no replace

depbi4txt already string; no replace

depbi5txt already string; no replace

depbi6txt already string; no replace

depbi7txt already string; no replace

depbi8txt already string; no replace

depbi9txt was byte now str1

anxbi1txt already string; no replace

anxbi2txt already string; no replace

anxbi3txt already string; no replace

anxbi4txt already string; no replace

anxbi5txt already string; no replace

anxbi6txt already string; no replace

anxbi7txt already string; no replace

anxbi8txt already string; no replace

anxbi9txt already string; no replace

Ldepbi1txt already string; no replace

Ldepbi2txt already string; no replace

Ldepbi3txt already string; no replace

Ldepbi4txt already string; no replace

Ldepbi5txt already string; no replace

Ldepbi6txt already string; no replace

Lanxbi1txt already string; no replace

Lanxbi2txt already string; no replace

Lanxbi3txt already string; no replace

Lanxbi4txt already string; no replace

Lanxbi5txt already string; no replace

Lanxbi6txt already string; no replace

Ldepmu1txt already string; no replace

Ldepmu2txt already string; no replace

Ldepmu3txt already string; no replace

Ldepmu4txt already string; no replace

Ldepmu5txt already string; no replace

Lanxmu1txt already string; no replace

Lanxmu2txt already string; no replace

Lanxmu3txt already string; no replace

Lanxmu4txt already string; no replace

Lanxmu5txt already string; no replace

Lanxmu6txt already string; no replace

Lanxmu7txt already string; no replace

Lanxmu8txt already string; no replace

Lanxmu9txt already string; no replace

Lanxmu10txt already string; no replace

Lanxmu11txt already string; no replace

.

.

. ***Create variable for study meas. PIU

. cap drop wasitPIU

. gen wasitPIU=.

(197 missing values generated)

. lab var wasitPIU "Did the study meas. PIU?"

.

. cap drop iascale

. generate str iascale= ""

(197 missing values generated)

. lab var iascale "Internet addic. scale for studies meas. PG and IA"

.

. ****Create variable for study meas. PIU & anx or dep

. cap drop IAdepyesno

. gen IAdepyesno=.

(197 missing values generated)

. lab var IAdepyesno "The study meas.d IA & dep"

. cap drop IAanxyesno

. gen IAanxyesno=.

(197 missing values generated)

. lab var IAanxyesno "The study meas.d IA & anx"

.

. ***replace values with No.s

.

. foreach var in ///

> PGscaleYN inkuon inkuin inking inmihar inmann ingonz insug longit {

2. cap drop `var'_2

3. clonevar `var'_2=`var'

4. replace `var'_2="0" if `var'_2=="0_no" | `var'_2=="0_No"

5. replace `var'_2="1" if `var'_2=="1_yes" | `var'_2=="1_Yes"

6. replace `var'_2="2" if `var'_2=="2_PG+other"

7. replace `var'_2="." if `var'_2=="*"

8. codebook `var' `var'_2

9. tab `var' `var'_2

10. destring `var'_2, replace force

11. codebook `var' `var'_2

12. tab `var' `var'_2, mi

13. drop `var'

14. rename `var'_2 `var'

15. }

(3 missing values generated)

(71 real changes made)

(114 real changes made)

(9 real changes made)

(0 real changes made)

------------------------------------------------------------------------------------------------------------------------------------

PGscaleYN PGscaleYN

------------------------------------------------------------------------------------------------------------------------------------

type: string (str10)

unique values: 3 missing "": 3/197

tabulation: Freq. Value

3 ""

71 "0_No"

114 "1_Yes"

9 "2_PG+other"

------------------------------------------------------------------------------------------------------------------------------------

PGscaleYN_2 PGscaleYN

------------------------------------------------------------------------------------------------------------------------------------

type: string (str10), but longest is str1

unique values: 3 missing "": 3/197

tabulation: Freq. Value

3 ""

71 "0"

114 "1"

9 "2"

| PGscaleYN

PGscaleYN | 0 1 2 | Total

-----------+---------------------------------+----------

0_No | 71 0 0 | 71

1_Yes | 0 114 0 | 114

2_PG+other | 0 0 9 | 9

-----------+---------------------------------+----------

Total | 71 114 9 | 194

PGscaleYN_2 contains nonnumeric characters; replaced as byte

(3 missing values generated)

------------------------------------------------------------------------------------------------------------------------------------

PGscaleYN PGscaleYN

------------------------------------------------------------------------------------------------------------------------------------

type: string (str10)

unique values: 3 missing "": 3/197

tabulation: Freq. Value

3 ""

71 "0_No"

114 "1_Yes"

9 "2_PG+other"

------------------------------------------------------------------------------------------------------------------------------------

PGscaleYN_2 PGscaleYN

------------------------------------------------------------------------------------------------------------------------------------

type: numeric (byte)

range: [0,2] units: 1

unique values: 3 missing .: 3/197

tabulation: Freq. Value

71 0

114 1

9 2

3 .

| PGscaleYN

PGscaleYN | 0 1 2 . | Total

-----------+--------------------------------------------+----------

| 0 0 0 3 | 3

0_No | 71 0 0 0 | 71

1_Yes | 0 114 0 0 | 114

2_PG+other | 0 0 9 0 | 9

-----------+--------------------------------------------+----------

Total | 71 114 9 3 | 197

(1 missing value generated)

(166 real changes made)

(30 real changes made)

(0 real changes made)

(0 real changes made)

------------------------------------------------------------------------------------------------------------------------------------

inkuon inkuon

------------------------------------------------------------------------------------------------------------------------------------

type: string (str5)

unique values: 2 missing "": 1/197

tabulation: Freq. Value

1 ""

166 "0_no"

30 "1_yes"

------------------------------------------------------------------------------------------------------------------------------------

inkuon_2 inkuon

------------------------------------------------------------------------------------------------------------------------------------

type: string (str5), but longest is str1

unique values: 2 missing "": 1/197

tabulation: Freq. Value

1 ""

166 "0"

30 "1"

| inkuon

inkuon | 0 1 | Total

-----------+----------------------+----------

0_no | 166 0 | 166

1_yes | 0 30 | 30

-----------+----------------------+----------

Total | 166 30 | 196

inkuon_2 contains nonnumeric characters; replaced as byte

(1 missing value generated)

------------------------------------------------------------------------------------------------------------------------------------

inkuon inkuon

------------------------------------------------------------------------------------------------------------------------------------

type: string (str5)

unique values: 2 missing "": 1/197

tabulation: Freq. Value

1 ""

166 "0_no"

30 "1_yes"

------------------------------------------------------------------------------------------------------------------------------------

inkuon_2 inkuon

------------------------------------------------------------------------------------------------------------------------------------

type: numeric (byte)

range: [0,1] units: 1

unique values: 2 missing .: 1/197

tabulation: Freq. Value

166 0

30 1

1 .

| inkuon

inkuon | 0 1 . | Total

-----------+---------------------------------+----------

| 0 0 1 | 1

0_no | 166 0 0 | 166

1_yes | 0 30 0 | 30

-----------+---------------------------------+----------

Total | 166 30 1 | 197

(139 real changes made)

(58 real changes made)

(0 real changes made)

(0 real changes made)

------------------------------------------------------------------------------------------------------------------------------------

inkuin inkuin

------------------------------------------------------------------------------------------------------------------------------------

type: string (str5)

unique values: 2 missing "": 0/197

tabulation: Freq. Value

139 "0_no"

58 "1_yes"

------------------------------------------------------------------------------------------------------------------------------------

inkuin_2 inkuin

------------------------------------------------------------------------------------------------------------------------------------

type: string (str5), but longest is str1

unique values: 2 missing "": 0/197

tabulation: Freq. Value

139 "0"

58 "1"

| inkuin

inkuin | 0 1 | Total

-----------+----------------------+----------

0_no | 139 0 | 139

1_yes | 0 58 | 58

-----------+----------------------+----------

Total | 139 58 | 197

inkuin_2 contains nonnumeric characters; replaced as byte

------------------------------------------------------------------------------------------------------------------------------------

inkuin inkuin

------------------------------------------------------------------------------------------------------------------------------------

type: string (str5)

unique values: 2 missing "": 0/197

tabulation: Freq. Value

139 "0_no"

58 "1_yes"

------------------------------------------------------------------------------------------------------------------------------------

inkuin_2 inkuin

------------------------------------------------------------------------------------------------------------------------------------

type: numeric (byte)

range: [0,1] units: 1

unique values: 2 missing .: 0/197

tabulation: Freq. Value

139 0

58 1

| inkuin

inkuin | 0 1 | Total

-----------+----------------------+----------

0_no | 139 0 | 139

1_yes | 0 58 | 58

-----------+----------------------+----------

Total | 139 58 | 197

(1 missing value generated)

(132 real changes made)

(64 real changes made)

(0 real changes made)

(0 real changes made)

------------------------------------------------------------------------------------------------------------------------------------

inking inking

------------------------------------------------------------------------------------------------------------------------------------

type: string (str5)

unique values: 2 missing "": 1/197

tabulation: Freq. Value

1 ""

132 "0_no"

64 "1_yes"

------------------------------------------------------------------------------------------------------------------------------------

inking_2 inking

------------------------------------------------------------------------------------------------------------------------------------

type: string (str5), but longest is str1

unique values: 2 missing "": 1/197

tabulation: Freq. Value

1 ""

132 "0"

64 "1"

| inking

inking | 0 1 | Total

-----------+----------------------+----------

0_no | 132 0 | 132

1_yes | 0 64 | 64

-----------+----------------------+----------

Total | 132 64 | 196

inking_2 contains nonnumeric characters; replaced as byte

(1 missing value generated)

------------------------------------------------------------------------------------------------------------------------------------

inking inking

------------------------------------------------------------------------------------------------------------------------------------

type: string (str5)

unique values: 2 missing "": 1/197

tabulation: Freq. Value

1 ""

132 "0_no"

64 "1_yes"

------------------------------------------------------------------------------------------------------------------------------------

inking_2 inking

------------------------------------------------------------------------------------------------------------------------------------

type: numeric (byte)

range: [0,1] units: 1

unique values: 2 missing .: 1/197

tabulation: Freq. Value

132 0

64 1

1 .

| inking

inking | 0 1 . | Total

-----------+---------------------------------+----------

| 0 0 1 | 1

0_no | 132 0 0 | 132

1_yes | 0 64 0 | 64

-----------+---------------------------------+----------

Total | 132 64 1 | 197

(1 missing value generated)

(149 real changes made)

(47 real changes made)

(0 real changes made)

(0 real changes made)

------------------------------------------------------------------------------------------------------------------------------------

inmihar inmihar

------------------------------------------------------------------------------------------------------------------------------------

type: string (str5)

unique values: 2 missing "": 1/197

tabulation: Freq. Value

1 ""

149 "0_no"

47 "1_yes"

------------------------------------------------------------------------------------------------------------------------------------

inmihar_2 inmihar

------------------------------------------------------------------------------------------------------------------------------------

type: string (str5), but longest is str1

unique values: 2 missing "": 1/197

tabulation: Freq. Value

1 ""

149 "0"

47 "1"

| inmihar

inmihar | 0 1 | Total

-----------+----------------------+----------

0_no | 149 0 | 149

1_yes | 0 47 | 47

-----------+----------------------+----------

Total | 149 47 | 196

inmihar_2 contains nonnumeric characters; replaced as byte

(1 missing value generated)

------------------------------------------------------------------------------------------------------------------------------------

inmihar inmihar

------------------------------------------------------------------------------------------------------------------------------------

type: string (str5)

unique values: 2 missing "": 1/197

tabulation: Freq. Value

1 ""

149 "0_no"

47 "1_yes"

------------------------------------------------------------------------------------------------------------------------------------

inmihar_2 inmihar

------------------------------------------------------------------------------------------------------------------------------------

type: numeric (byte)

range: [0,1] units: 1

unique values: 2 missing .: 1/197

tabulation: Freq. Value

149 0

47 1

1 .

| inmihar

inmihar | 0 1 . | Total

-----------+---------------------------------+----------

| 0 0 1 | 1

0_no | 149 0 0 | 149

1_yes | 0 47 0 | 47

-----------+---------------------------------+----------

Total | 149 47 1 | 197

(1 missing value generated)

(146 real changes made)

(50 real changes made)

(0 real changes made)

(0 real changes made)

------------------------------------------------------------------------------------------------------------------------------------

inmann inmann

------------------------------------------------------------------------------------------------------------------------------------

type: string (str5)

unique values: 2 missing "": 1/197

tabulation: Freq. Value

1 ""

146 "0_no"

50 "1_yes"

------------------------------------------------------------------------------------------------------------------------------------

inmann_2 inmann

------------------------------------------------------------------------------------------------------------------------------------

type: string (str5), but longest is str1

unique values: 2 missing "": 1/197

tabulation: Freq. Value

1 ""

146 "0"

50 "1"

| inmann

inmann | 0 1 | Total

-----------+----------------------+----------

0_no | 146 0 | 146

1_yes | 0 50 | 50

-----------+----------------------+----------

Total | 146 50 | 196

inmann_2 contains nonnumeric characters; replaced as byte

(1 missing value generated)

------------------------------------------------------------------------------------------------------------------------------------

inmann inmann

------------------------------------------------------------------------------------------------------------------------------------

type: string (str5)

unique values: 2 missing "": 1/197

tabulation: Freq. Value

1 ""

146 "0_no"

50 "1_yes"

------------------------------------------------------------------------------------------------------------------------------------

inmann_2 inmann

------------------------------------------------------------------------------------------------------------------------------------

type: numeric (byte)

range: [0,1] units: 1

unique values: 2 missing .: 1/197

tabulation: Freq. Value

146 0

50 1

1 .

| inmann

inmann | 0 1 . | Total

-----------+---------------------------------+----------

| 0 0 1 | 1

0_no | 146 0 0 | 146

1_yes | 0 50 0 | 50

-----------+---------------------------------+----------

Total | 146 50 1 | 197

(173 real changes made)

(24 real changes made)

(0 real changes made)

(0 real changes made)

------------------------------------------------------------------------------------------------------------------------------------

ingonz ingonz

------------------------------------------------------------------------------------------------------------------------------------

type: string (str5)

unique values: 2 missing "": 0/197

tabulation: Freq. Value

173 "0_no"

24 "1_yes"

------------------------------------------------------------------------------------------------------------------------------------

ingonz_2 ingonz

------------------------------------------------------------------------------------------------------------------------------------

type: string (str5), but longest is str1

unique values: 2 missing "": 0/197

tabulation: Freq. Value

173 "0"

24 "1"

| ingonz

ingonz | 0 1 | Total

-----------+----------------------+----------

0_no | 173 0 | 173

1_yes | 0 24 | 24

-----------+----------------------+----------

Total | 173 24 | 197

ingonz_2 contains nonnumeric characters; replaced as byte

------------------------------------------------------------------------------------------------------------------------------------

ingonz ingonz

------------------------------------------------------------------------------------------------------------------------------------

type: string (str5)

unique values: 2 missing "": 0/197

tabulation: Freq. Value

173 "0_no"

24 "1_yes"

------------------------------------------------------------------------------------------------------------------------------------

ingonz_2 ingonz

------------------------------------------------------------------------------------------------------------------------------------

type: numeric (byte)

range: [0,1] units: 1

unique values: 2 missing .: 0/197

tabulation: Freq. Value

173 0

24 1

| ingonz

ingonz | 0 1 | Total

-----------+----------------------+----------

0_no | 173 0 | 173

1_yes | 0 24 | 24

-----------+----------------------+----------

Total | 173 24 | 197

(153 real changes made)

(44 real changes made)

(0 real changes made)

(0 real changes made)

------------------------------------------------------------------------------------------------------------------------------------

insug insug

------------------------------------------------------------------------------------------------------------------------------------

type: string (str5)

unique values: 2 missing "": 0/197

tabulation: Freq. Value

153 "0_no"

44 "1_yes"

------------------------------------------------------------------------------------------------------------------------------------

insug_2 insug

------------------------------------------------------------------------------------------------------------------------------------

type: string (str5), but longest is str1

unique values: 2 missing "": 0/197

tabulation: Freq. Value

153 "0"

44 "1"

| insug

insug | 0 1 | Total

-----------+----------------------+----------

0_no | 153 0 | 153

1_yes | 0 44 | 44

-----------+----------------------+----------

Total | 153 44 | 197

insug_2 contains nonnumeric characters; replaced as byte

------------------------------------------------------------------------------------------------------------------------------------

insug insug

------------------------------------------------------------------------------------------------------------------------------------

type: string (str5)

unique values: 2 missing "": 0/197

tabulation: Freq. Value

153 "0_no"

44 "1_yes"

------------------------------------------------------------------------------------------------------------------------------------

insug_2 insug

------------------------------------------------------------------------------------------------------------------------------------

type: numeric (byte)

range: [0,1] units: 1

unique values: 2 missing .: 0/197

tabulation: Freq. Value

153 0

44 1

| insug

insug | 0 1 | Total

-----------+----------------------+----------

0_no | 153 0 | 153

1_yes | 0 44 | 44

-----------+----------------------+----------

Total | 153 44 | 197

(3 missing values generated)

(178 real changes made)

(16 real changes made)

(0 real changes made)

(0 real changes made)

------------------------------------------------------------------------------------------------------------------------------------

longit longit

------------------------------------------------------------------------------------------------------------------------------------

type: string (str5)

unique values: 2 missing "": 3/197

tabulation: Freq. Value

3 ""

178 "0_no"

16 "1_yes"

------------------------------------------------------------------------------------------------------------------------------------

longit_2 longit

------------------------------------------------------------------------------------------------------------------------------------

type: string (str5), but longest is str1

unique values: 2 missing "": 3/197

tabulation: Freq. Value

3 ""

178 "0"

16 "1"

| longit

longit | 0 1 | Total

-----------+----------------------+----------

0_no | 178 0 | 178

1_yes | 0 16 | 16

-----------+----------------------+----------

Total | 178 16 | 194

longit_2 contains nonnumeric characters; replaced as byte

(3 missing values generated)

------------------------------------------------------------------------------------------------------------------------------------

longit longit

------------------------------------------------------------------------------------------------------------------------------------

type: string (str5)

unique values: 2 missing "": 3/197

tabulation: Freq. Value

3 ""

178 "0_no"

16 "1_yes"

------------------------------------------------------------------------------------------------------------------------------------

longit_2 longit

------------------------------------------------------------------------------------------------------------------------------------

type: numeric (byte)

range: [0,1] units: 1

unique values: 2 missing .: 3/197

tabulation: Freq. Value

178 0

16 1

3 .

| longit

longit | 0 1 . | Total

-----------+---------------------------------+----------

| 0 0 3 | 3

0_no | 178 0 0 | 178

1_yes | 0 16 0 | 16

-----------+---------------------------------+----------

Total | 178 16 3 | 197

. tab PGscaleYN wasit_PG, mi

| wasit_PG

PGscaleYN | 0 1 2 . | Total

-----------+--------------------------------------------+----------

0 | 71 0 0 0 | 71

1 | 0 114 0 0 | 114

2 | 0 1 8 0 | 9

. | 0 0 0 3 | 3

-----------+--------------------------------------------+----------

Total | 71 115 8 3 | 197

.

. lab var ingonz "Study was included in Gonzalez-Bueso"

. lab var insug "Study was included in Sugaya"

. lab var inmiha "Study was included in Mihara"

. lab var inmann "Study was included in Mannikko"

. lab var inking "Study was included in King"

. lab var inkuin "Study was included in Kuss Internet Gaming Addiction"

. lab var inkuon "Study was included in Kuss Online Gaming Addiction"

. lab var lang "Language"

. lab var neuro "Neuroimaging study"

. lab var case "Case study/series"

. lab var long6 "Longitudinal study with 6mos or greater follow up"

. lab var pgscale "Scale used to measure PG"

. lab var sampsi "Sample size"

. lab var totEL "total reviews for which a study is eligible for inclusion"

. foreach var in txtexcgon txtexck txtexclkuin txtexclman ///

> txtexcmih txtexcsug txtsxckuon {

2. lab var `var' "Reasons why study determined not eligible for review"

3. }

. lab var longit "Study had longitudinal results"

. *****CHECK RESULTS OF IMPORTING

. **Kwon 2009 is actually Kwon 2011, so same in King and KUIN

. cap drop depanxnote

. gen depanxnote=""

(197 missing values generated)

. lab var depanxnote "reasons why scale is not dep/anx"

. codebook depanxnote

------------------------------------------------------------------------------------------------------------------------------------

depanxnote reasons why scale is not dep/anx

------------------------------------------------------------------------------------------------------------------------------------

type: string (str1), but longest is str0

unique values: 0 missing "": 197/197

tabulation: Freq. Value

197 ""

. drop if id==79

(1 observation deleted)

.

. *Change indicator for being in King to 1 for Kwon 2011

. replace inking=1 if id==80

(0 real changes made)

. list in 78/80

+-----------------------------------------------------------------------------------------------------------------------------+

78. | study | notes | GAclin~p | pgscale | ages | sampsi | lang | case | exper | neuro | long6 |

| Konkoly_2015 | | 0_No | gaming or "int chat" as BAD | | | | | | | 1_yes |

|------------------------------------------------------------------+----------------------------------------------------------|

| elmiha | txtexc~h | elkig | txtexck | elmann | txtex~an | elkuon | txtsxc~n | elkuin | txtex~in | elgonz | txtex~on | elsug |

| . | | . | | . | | . | | . | | . | | . |

|-----------------------------------------------------------------------------------------------------------------------------|

| txtexc~g | totEL | wasit_PG | year | PGdepy~o | PGanxy~o | depmu1~t | depmu1~e | depmu2~t | depmu2~e | depmu3~t | depmu3~e |

| | . | 2 | 2015 | 0_No | 0_No | | . | | . | | . |

|----------+------------------------------------------------------------------------------------------------------+-----------|

| depmu4~t | depmu4~e | anxmu1~t | anxmu1~e | anxmu2~t | anxmu2~e | anxmu3~t | anxmu3~e | anxmu4~t | anxmu4~e | anxmu5~t |

| | . | | . | | . | | . | | . | |

|----------+----------+----------+----------+----------+----------+-----------+-----------+-----------+-----------+-----------|

| anxmu5~e | anxmu6~t | anxmu6~e | anxmu7~t | anxmu7~e | anxmu8~t | anxmu8~e | anxmu9~t | anxmu9~e | depbi1~t | depbi1~e |

| . | | . | | . | | . | | . | | . |

|----------+----------+----------+----------+----------+----------+-----------+-----------+-----------+-----------+-----------|

| depbi2~t | depbi2~e | depbi3~t | depbi3~e | depbi4~t | depbi4~e | depbi5~t | depbi5~e | depbi6~t | depbi6~e | depbi7~t |

| | . | | . | | . | | . | | . | |

|----------+----------+----------+----------+----------+----------+-----------+-----------+-----------+-----------+-----------|

| depbi7~e | depbi8~t | depbi8~e | depbi9~t | depbi9~e | anxbi1~t | anxbi1~e | anxbi2~t | anxbi2~e | anxbi3~t | anxbi3~e |

| . | | . | . | . | | . | | . | | . |

|----------+----------+----------+----------+----------+----------+-----------+-----------+-----------+-----------+-----------|

| anxbi4~t | anxbi4~e | anxbi5~t | anxbi5~e | anxbi6~t | anxbi6~e | anxbi7~t | anxbi7~e | anxbi8~t | anxbi8~e | anxbi9~t |

| | . | | . | | . | | . | | . | |

|----------+----------+----------+----------+----------+----------+-----------+-----------+-----------+-----------+-----------|

| anxbi9~e | Ld~i1txt | Ldepbi.. | Ld~i2txt | Ldepbi.. | Ld~i3txt | Ldepbi.. | Ld~i4txt | Ldepbi.. | Ld~i5txt | Ldepbi.. |

| . | | . | | . | . | . | . | . | . | . |

|----------+----------+----------+----------+----------+----------+-----------+-----------+-----------+-----------+-----------|

| Lde~6txt | Ld~6vote | La~i1txt | Lanxbi.. | La~i2txt | Lanxbi.. | La~i3txt | Lanxbi.. | La~i4txt | Lanxbi.. | La~i5txt |

| . | . | . | . | . | . | . | . | . | . | . |

|----------+----------+----------+----------+----------+----------+-----------+-----------+-----------+-----------+-----------|

| Lanxbi.. | La~i6txt | Lanxbi.. | Ld~u1txt | Ldepmu.. | Ld~u2txt | Ldepmu.. | Ld~u3txt | Ldepmu.. | Ld~u4txt | Ldepmu.. |

| . | . | . | | . | | . | | . | | . |

|----------+----------+----------+----------+----------+----------+-----------+-----------+-----------+-----------+-----------|

| Ld~u5txt | Ldepmu.. | La~u1txt | Lanxmu.. | La~u2txt | Lanxmu.. | La~u3txt | Lanxmu.. | La~u4txt | Lanxmu.. | La~u5txt |

| . | . | | . | | . | | . | | . | |

|----------+----------+----------+----------+----------+----------+-----------+-----------+-----------+-----------+-----------|

| Lanxmu.. | La~u6txt | L~u6vote | Lan~7txt | La~7vote | Lan~8txt | La~8vote | Lan~9txt | La~9vote | Lan~0txt | La~0vote |

| . | | . | | . | | . | | . | | . |

|----------+----------+-------------------------------------------------------------------------------------------------------|

| La~11txt | L~11vote | id | old_PGdepye~o | old_PGanxye~o | wasitPIU | iascale | IAdepy~o | IAanxy~o | PGscal~N | inkuon |

| | . | 78 | 0_No | 0_No | . | | . | . | 2 | 0 |

|-------------------------------------------------------------------------------------------+---------------------------------|

| inkuin | inking | inmihar | inmann | ingonz | insug | longit | depanx~e |

| 0 | 0 | 1 | 0 | 0 | 0 | 1 | |

+-----------------------------------------------------------------------------------------------------------------------------+

+-----------------------------------------------------------------------------------------------------------------------------+

79. | study | notes | GAclin~p | pgscale | ages | sampsi | lang | case | exper | neuro | long6 |

| Kwon_2011 | | 0_No | IAS (adapted to games) | | | | | | | 0_no |

|------------------------------------------------------------------+----------------------------------------------------------|

| elmiha | txtexc~h | elkig | txtexck | elmann | txtex~an | elkuon | txtsxc~n | elkuin | txtex~in | elgonz | txtex~on | elsug |

| . | | . | | . | | . | | . | | . | | . |

|-----------------------------------------------------------------------------------------------------------------------------|

| txtexc~g | totEL | wasit_PG | year | PGdepy~o | PGanxy~o | depmu1~t | depmu1~e | depmu2~t | depmu2~e | depmu3~t | depmu3~e |

| | . | 1 | 2011 | 0_No | 0_No | | . | | . | | . |

|----------+------------------------------------------------------------------------------------------------------+-----------|

| depmu4~t | depmu4~e | anxmu1~t | anxmu1~e | anxmu2~t | anxmu2~e | anxmu3~t | anxmu3~e | anxmu4~t | anxmu4~e | anxmu5~t |

| | . | | . | | . | | . | | . | |

|----------+----------+----------+----------+----------+----------+-----------+-----------+-----------+-----------+-----------|

| anxmu5~e | anxmu6~t | anxmu6~e | anxmu7~t | anxmu7~e | anxmu8~t | anxmu8~e | anxmu9~t | anxmu9~e | depbi1~t | depbi1~e |

| . | | . | | . | | . | | . | | . |

|----------+----------+----------+----------+----------+----------+-----------+-----------+-----------+-----------+-----------|

| depbi2~t | depbi2~e | depbi3~t | depbi3~e | depbi4~t | depbi4~e | depbi5~t | depbi5~e | depbi6~t | depbi6~e | depbi7~t |

| | . | | . | | . | | . | | . | |

|----------+----------+----------+----------+----------+----------+-----------+-----------+-----------+-----------+-----------|

| depbi7~e | depbi8~t | depbi8~e | depbi9~t | depbi9~e | anxbi1~t | anxbi1~e | anxbi2~t | anxbi2~e | anxbi3~t | anxbi3~e |

| . | | . | . | . | | . | | . | | . |

|----------+----------+----------+----------+----------+----------+-----------+-----------+-----------+-----------+-----------|

| anxbi4~t | anxbi4~e | anxbi5~t | anxbi5~e | anxbi6~t | anxbi6~e | anxbi7~t | anxbi7~e | anxbi8~t | anxbi8~e | anxbi9~t |

| | . | | . | | . | | . | | . | |

|----------+----------+----------+----------+----------+----------+-----------+-----------+-----------+-----------+-----------|

| anxbi9~e | Ld~i1txt | Ldepbi.. | Ld~i2txt | Ldepbi.. | Ld~i3txt | Ldepbi.. | Ld~i4txt | Ldepbi.. | Ld~i5txt | Ldepbi.. |

| . | | . | | . | . | . | . | . | . | . |

|----------+----------+----------+----------+----------+----------+-----------+-----------+-----------+-----------+-----------|

| Lde~6txt | Ld~6vote | La~i1txt | Lanxbi.. | La~i2txt | Lanxbi.. | La~i3txt | Lanxbi.. | La~i4txt | Lanxbi.. | La~i5txt |

| . | . | . | . | . | . | . | . | . | . | . |

|----------+----------+----------+----------+----------+----------+-----------+-----------+-----------+-----------+-----------|

| Lanxbi.. | La~i6txt | Lanxbi.. | Ld~u1txt | Ldepmu.. | Ld~u2txt | Ldepmu.. | Ld~u3txt | Ldepmu.. | Ld~u4txt | Ldepmu.. |

| . | . | . | | . | | . | | . | | . |

|----------+----------+----------+----------+----------+----------+-----------+-----------+-----------+-----------+-----------|

| Ld~u5txt | Ldepmu.. | La~u1txt | Lanxmu.. | La~u2txt | Lanxmu.. | La~u3txt | Lanxmu.. | La~u4txt | Lanxmu.. | La~u5txt |

| . | . | | . | | . | | . | | . | |

|----------+----------+----------+----------+----------+----------+-----------+-----------+-----------+-----------+-----------|

| Lanxmu.. | La~u6txt | L~u6vote | Lan~7txt | La~7vote | Lan~8txt | La~8vote | Lan~9txt | La~9vote | Lan~0txt | La~0vote |

| . | | . | | . | | . | | . | | . |

|----------+----------+-------------------------------------------------------------------------------------------------------|

| La~11txt | L~11vote | id | old_PGdepye~o | old_PGanxye~o | wasitPIU | iascale | IAdepy~o | IAanxy~o | PGscal~N | inkuon |

| | . | 80 | 3_nonspecific | 3_nonspecific | . | | . | . | 1 | 1 |

|-------------------------------------------------------------------------------------------+---------------------------------|

| inkuin | inking | inmihar | inmann | ingonz | insug | longit | depanx~e |

| 0 | 1 | 0 | 1 | 0 | 0 | 0 | |

+-----------------------------------------------------------------------------------------------------------------------------+

+-----------------------------------------------------------------------------------------------------------------------------+

80. | study | notes | GAclin~p | pgscale | ages | sampsi | lang | case | exper | neuro | long6 |

| Lafreniere_2009 | | 0_No | PVP | | | | | | | 0_no |

|------------------------------------------------------------------+----------------------------------------------------------|

| elmiha | txtexc~h | elkig | txtexck | elmann | txtex~an | elkuon | txtsxc~n | elkuin | txtex~in | elgonz | txtex~on | elsug |

| . | | . | | . | | . | | . | | . | | . |

|-----------------------------------------------------------------------------------------------------------------------------|

| txtexc~g | totEL | wasit_PG | year | PGdepy~o | PGanxy~o | depmu1~t | depmu1~e | depmu2~t | depmu2~e | depmu3~t | depmu3~e |

| | . | 1 | 2009 | 0_No | 0_No | | . | | . | | . |

|----------+------------------------------------------------------------------------------------------------------+-----------|

| depmu4~t | depmu4~e | anxmu1~t | anxmu1~e | anxmu2~t | anxmu2~e | anxmu3~t | anxmu3~e | anxmu4~t | anxmu4~e | anxmu5~t |

| | . | | . | | . | | . | | . | |

|----------+----------+----------+----------+----------+----------+-----------+-----------+-----------+-----------+-----------|

| anxmu5~e | anxmu6~t | anxmu6~e | anxmu7~t | anxmu7~e | anxmu8~t | anxmu8~e | anxmu9~t | anxmu9~e | depbi1~t | depbi1~e |

| . | | . | | . | | . | | . | | . |

|----------+----------+----------+----------+----------+----------+-----------+-----------+-----------+-----------+-----------|

| depbi2~t | depbi2~e | depbi3~t | depbi3~e | depbi4~t | depbi4~e | depbi5~t | depbi5~e | depbi6~t | depbi6~e | depbi7~t |

| | . | | . | | . | | . | | . | |

|----------+----------+----------+----------+----------+----------+-----------+-----------+-----------+-----------+-----------|

| depbi7~e | depbi8~t | depbi8~e | depbi9~t | depbi9~e | anxbi1~t | anxbi1~e | anxbi2~t | anxbi2~e | anxbi3~t | anxbi3~e |

| . | | . | . | . | | . | | . | | . |

|----------+----------+----------+----------+----------+----------+-----------+-----------+-----------+-----------+-----------|

| anxbi4~t | anxbi4~e | anxbi5~t | anxbi5~e | anxbi6~t | anxbi6~e | anxbi7~t | anxbi7~e | anxbi8~t | anxbi8~e | anxbi9~t |

| | . | | . | | . | | . | | . | |

|----------+----------+----------+----------+----------+----------+-----------+-----------+-----------+-----------+-----------|

| anxbi9~e | Ld~i1txt | Ldepbi.. | Ld~i2txt | Ldepbi.. | Ld~i3txt | Ldepbi.. | Ld~i4txt | Ldepbi.. | Ld~i5txt | Ldepbi.. |

| . | | . | | . | . | . | . | . | . | . |

|----------+----------+----------+----------+----------+----------+-----------+-----------+-----------+-----------+-----------|

| Lde~6txt | Ld~6vote | La~i1txt | Lanxbi.. | La~i2txt | Lanxbi.. | La~i3txt | Lanxbi.. | La~i4txt | Lanxbi.. | La~i5txt |

| . | . | . | . | . | . | . | . | . | . | . |

|----------+----------+----------+----------+----------+----------+-----------+-----------+-----------+-----------+-----------|

| Lanxbi.. | La~i6txt | Lanxbi.. | Ld~u1txt | Ldepmu.. | Ld~u2txt | Ldepmu.. | Ld~u3txt | Ldepmu.. | Ld~u4txt | Ldepmu.. |

| . | . | . | | . | | . | | . | | . |

|----------+----------+----------+----------+----------+----------+-----------+-----------+-----------+-----------+-----------|

| Ld~u5txt | Ldepmu.. | La~u1txt | Lanxmu.. | La~u2txt | Lanxmu.. | La~u3txt | Lanxmu.. | La~u4txt | Lanxmu.. | La~u5txt |

| . | . | | . | | . | | . | | . | |

|----------+----------+----------+----------+----------+----------+-----------+-----------+-----------+-----------+-----------|

| Lanxmu.. | La~u6txt | L~u6vote | Lan~7txt | La~7vote | Lan~8txt | La~8vote | Lan~9txt | La~9vote | Lan~0txt | La~0vote |

| . | | . | | . | | . | | . | | . |

|----------+----------+-------------------------------------------------------------------------------------------------------|

| La~11txt | L~11vote | id | old_PGdepye~o | old_PGanxye~o | wasitPIU | iascale | IAdepy~o | IAanxy~o | PGscal~N | inkuon |

| | . | 81 | 0_No | 0_No | . | | . | . | 1 | 0 |

|-------------------------------------------------------------------------------------------+---------------------------------|

| inkuin | inking | inmihar | inmann | ingonz | insug | longit | depanx~e |

| 0 | 1 | 0 | 1 | 0 | 0 | 0 | |

+-----------------------------------------------------------------------------------------------------------------------------+

.

.

. list study id longit PG* if longit==1

+-------------------------------------------------------------------+

| study id longit PGdepy~o PGanxy~o PGscal~N |

|-------------------------------------------------------------------|

12. | Brunborg_2014 12 1 1_Yes 0_No 1 |

29. | Coyne_2015 29 1 0_No 1_Yes 1 |

38. | Gentile_2011 38 1 1_Yes 1_Yes 1 |

46. | Haagsma_2013 TPB 46 1 0_No 0_No 1 |

53. | Henchoz_2016 53 1 0_No 0_No 1 |

|-------------------------------------------------------------------|

56. | Hull_2013 56 1 0_No 0_No 1 |

73. | King_2013 traj 73 1 0_No 0_No 1 |

78. | Konkoly_2015 78 1 0_No 0_No 2 |

86. | Lemmens_2011_agg 87 1 0_No 0_No 1 |

87. | Lemmens_2011_psyc 88 1 0_No 0_No 1 |

|-------------------------------------------------------------------|

96. | Meerkerk_2006 97 1 0_No 0_No 0 |

116. | Rehbein_2013 117 1 0_No 0_No 1 |

120. | Scharkow_2014 121 1 0_No 0_No 1 |

121. | Schmitt_2015 122 1 0_No 0_No 1 |

141. | VRooij_2011 142 1 1_Yes 1_Yes 0 |

|-------------------------------------------------------------------|

155. | Yu_2015 156 1 0_No 0_No 1 |

+-------------------------------------------------------------------+

. ***brunborg 2014, Coyne, Gentile, Han, Van Rooij 2011 should all have longit anx/dep results

. list if id==12

+-----------------------------------------------------------------------------------------------------------------------------+

12. | study | notes | GAclin~p | pgscale | ages | sampsi | lang | case | exper | neuro | long6 | elmiha | txtexc~h |

| Brunborg_2014 | | 0_No | GAS | 13-17 | 1928 | eng | no | no | no | 1_yes | 1 | |

|-----------------------------------------------------------------------------------------------------------------------------|

| elkig | txtexck | elmann | txtex~an | elkuon | txtsxc~n | elkuin | txtex~in | elgonz | txtex~on | elsug | txtexc~g | totEL |

| 0 | year | 1 | | 0 | year | 0 | year | 1 | | 1 | | 4 |

|-----------------+-----------------------------------------------------------+-----------------------------------------------|

| wasit_PG | year | PGdepy~o | PGanxy~o | depmu1~t | depmu1~e | depmu2~t | depmu2~e | depmu3~t | depmu3~e | depmu4~t |

| 1 | 2014 | 1_Yes | 0_No | | . | | . | | . | |

|----------+------------------------------------------------------+-----------+-----------+-----------+-----------+-----------|

| depmu4~e | anxmu1~t | anxmu1~e | anxmu2~t | anxmu2~e | anxmu3~t | anxmu3~e | anxmu4~t | anxmu4~e | anxmu5~t | anxmu5~e |

| . | | . | | . | | . | | . | | . |

|-----------------------------------------------------------------------------+-----------------------------------------------|

| anxmu6~t | anxmu6~e | anxmu7~t | anxmu7~e | anxmu8~t | anxmu8~e | anxmu9~t | anxmu9~e |

| | . | | . | | . | | . |

|-----------------------------------------------------------------------------------------------------------------------------|

| depbi1txt | depbi1~e | depbi2txt | depbi2~e | depbi3~t | depbi3~e |

| Time1 depression Time1 PG correlation | 1 | Time2 depression Time2 PG correlation | 1 | | . |

|-----------------------------------------------------------------------------------------------------+-----------+-----------|

| depbi4~t | depbi4~e | depbi5~t | depbi5~e | depbi6~t | depbi6~e | depbi7~t | depbi7~e | depbi8~t | depbi8~e | depbi9~t |

| | . | | . | | . | | . | | . | . |

|----------+----------+----------+----------+----------+----------+-----------+-----------+-----------+-----------+-----------|

| depbi9~e | anxbi1~t | anxbi1~e | anxbi2~t | anxbi2~e | anxbi3~t | anxbi3~e | anxbi4~t | anxbi4~e | anxbi5~t | anxbi5~e |

| . | | . | | . | | . | | . | | . |

|----------+----------+----------+----------+----------+----------+-----------------------------------------------------------|

| anxbi6~t | anxbi6~e | anxbi7~t | anxbi7~e | anxbi8~t | anxbi8~e | anxbi9~t | anxbi9~e | Ldepbi1txt | Ldepbi.. |

| | . | | . | | . | | . | Time1 depression~Time2PG | 0 |

|------------------------------------------------------------------------------------------------------------------+----------|

| Ldepbi2txt | Ldepbi.. | Ld~i3txt | Ldepbi.. | Ld~i4txt | Ldepbi.. | Ld~i5txt | Ldepbi.. | Lde~6txt | Ld~6vote |

| Time1PG~Time2 depression | 0 | . | . | . | . | . | . | . | . |

|-----------------------------------------------------------------------------------------------------------------------------|

| La~i1txt | Lanxbi.. | La~i2txt | Lanxbi.. | La~i3txt | Lanxbi.. | La~i4txt | Lanxbi.. | La~i5txt | Lanxbi.. | La~i6txt |

| . | . | . | . | . | . | . | . | . | . | . |

|----------+------------------------------------------------------------------------------------------------------+-----------|

| Lanxbi.. | Ldepmu1txt | Ldepmu.. | Ld~u2txt | Ldepmu.. | Ld~u3txt | Ldepmu.. | Ld~u4txt | Ldepmu.. | Ld~u5txt |

| . | FD regression, weighted | 1 | | . | | . | | . | . |

|----------+------------------------------------------------------------------------------------------------------+-----------|

| Ldepmu.. | La~u1txt | Lanxmu.. | La~u2txt | Lanxmu.. | La~u3txt | Lanxmu.. | La~u4txt | Lanxmu.. | La~u5txt | Lanxmu.. |

| . | | . | | . | | . | | . | | . |

|----------+----------+----------+----------+----------+----------+-----------+-----------+-----------+-----------+-----------|

| La~u6txt | L~u6vote | Lan~7txt | La~7vote | Lan~8txt | La~8vote | Lan~9txt | La~9vote | Lan~0txt | La~0vote | La~11txt |

| | . | | . | | . | | . | | . | |

|----------+------------------------------------------------------------------------------------------------------------------|

| L~11vote | id | o~pyesno | o~xyesno | wasitPIU | iascale | IAdepy~o | IAanxy~o | PGscal~N | inkuon | inkuin | inking |

| . | 12 | 1_Yes | 0_No | . | | . | . | 1 | 0 | 0 | 0 |

|-----------------------------------------------------------------------------------------------------------------------------|

| inmihar | inmann | ingonz | insug | longit | depanx~e |

| 1 | 1 | 1 | 0 | 1 | |

+-----------------------------------------------------------------------------------------------------------------------------+

. list if id==29

+-----------------------------------------------------------------------------------------------------------------------------+

29. | study | notes | GAclin~p | pgscale | ages | sampsi | lang | case | exper | neuro | long6 |

| Coyne_2015 | | 0_No | Adapted DSM IV TR gaming | 13-18 (M15.3) | 374 | eng | no | no | maybe | 1_yes |

|-----------------------------------------------------------------------------------------------------------------------------|

| elmiha | txtexc~h | elkig | txtexck | elmann | txtex~an | elkuon | txtsxc~n | elkuin | txtex~in | elgonz | txtex~on | elsug |

| 1 | | 0 | year | 1 | | 0 | year | 0 | year | 1 | | 1 |

|-----------------------------------------------------------------------------------------------------------------------------|

| txtexc~g | totEL | wasit_PG | year | PGdepy~o | PGanxy~o | depmu1~t | depmu1~e | depmu2~t | depmu2~e | depmu3~t | depmu3~e |

| | 4 | 1 | 2015 | 0_No | 1_Yes | | . | | . | | . |

|-----------------------------------------------------------------------------------------------------------------------------|

| depmu4~t | depmu4~e | anxmu1txt | anxmu1~e |

| | . | anxiety on number of symptoms/Rubik's condition | 1 |

|-----------------------------------------------------------------------------------------------------------------------------|

| anxmu2txt | anxmu2~e | anxmu3txt | anxmu3~e |

| anxiety on presence of symptoms/problem solving condition | -1 | anxiety/SC/number of symptoms | 1 |

|-----------------------------------------------------------------------------------------------------------------------------|

| anxmu4~t | anxmu4~e | anxmu5~t | anxmu5~e | anxmu6~t | anxmu6~e | anxmu7~t | anxmu7~e | anxmu8~t | anxmu8~e | anxmu9~t |

| | . | | . | | . | | . | | . | |

|----------+----------+----------+----------+----------+----------+-----------+-----------+-----------+-----------+-----------|

| anxmu9~e | depbi1~t | depbi1~e | depbi2~t | depbi2~e | depbi3~t | depbi3~e | depbi4~t | depbi4~e | depbi5~t | depbi5~e |

| . | | . | | . | | . | | . | | . |

|----------+----------+----------+----------+----------+----------+-----------+-----------+-----------+-----------+-----------|

| depbi6~t | depbi6~e | depbi7~t | depbi7~e | depbi8~t | depbi8~e | depbi9~t | depbi9~e | anxbi1~t | anxbi1~e | anxbi2~t |

| | . | | . | | . | . | . | | . | |

|----------+----------+----------+----------+----------+----------+-----------+-----------+-----------+-----------+-----------|

| anxbi2~e | anxbi3~t | anxbi3~e | anxbi4~t | anxbi4~e | anxbi5~t | anxbi5~e | anxbi6~t | anxbi6~e | anxbi7~t | anxbi7~e |

| . | | . | | . | | . | | . | | . |

|----------+----------+----------+----------+----------+----------+-----------+-----------+-----------+-----------+-----------|

| anxbi8~t | anxbi8~e | anxbi9~t | anxbi9~e | Ld~i1txt | Ldepbi.. | Ld~i2txt | Ldepbi.. | Ld~i3txt | Ldepbi.. | Ld~i4txt |

| | . | | . | | . | | . | . | . | . |

|----------+----------+----------+----------+----------+----------+-----------+-----------+-----------+-----------+-----------|

| Ldepbi.. | Ld~i5txt | Ldepbi.. | Lde~6txt | Ld~6vote | La~i1txt | Lanxbi.. | La~i2txt | Lanxbi.. | La~i3txt | Lanxbi.. |

| . | . | . | . | . | . | . | . | . | . | . |

|----------+----------+----------+----------+----------+----------+-----------+-----------+-----------+-----------+-----------|

| La~i4txt | Lanxbi.. | La~i5txt | Lanxbi.. | La~i6txt | Lanxbi.. | Ld~u1txt | Ldepmu.. | Ld~u2txt | Ldepmu.. | Ld~u3txt |

| . | . | . | . | . | . | | . | | . | |

|----------+----------+----------+----------+----------+----------+-----------+-----------+-----------+-----------+-----------|

| Ldepmu.. | Ld~u4txt | Ldepmu.. | Ld~u5txt | Ldepmu.. | La~u1txt | Lanxmu.. | La~u2txt | Lanxmu.. | La~u3txt | Lanxmu.. |

| . | | . | . | . | | . | | . | | . |

|----------+----------+----------+----------+----------+----------+-----------+-----------+-----------+-----------+-----------|

| La~u4txt | Lanxmu.. | La~u5txt | Lanxmu.. | La~u6txt | L~u6vote | Lan~7txt | La~7vote | Lan~8txt | La~8vote | Lan~9txt |

| | . | | . | | . | | . | | . | |

|----------+----------+----------+----------+----------+----------------------------------------------------------+-----------|

| La~9vote | Lan~0txt | La~0vote | La~11txt | L~11vote | id | o~pyesno | o~xyesno | wasitPIU | iascale | IAdepy~o | IAanxy~o |

| . | | . | | . | 29 | 0_No | 1_Yes | . | | . | . |

|-----------------------------------------------------------------------------------------------------------------------------|

| PGscal~N | inkuon | inkuin | inking | inmihar | inmann | ingonz | insug | longit | depanx~e |

| 1 | 0 | 0 | 0 | 0 | 1 | 0 | 0 | 1 | |

+-----------------------------------------------------------------------------------------------------------------------------+

. list if id==38

+-----------------------------------------------------------------------------------------------------------------------------+

38. | study | notes | GAclin~p | pgscale | ages | sampsi | lang | case | exper | neuro |

| Gentile_2011 | | 0_No | Adapted DSM-IV-TR.latent classes | avg 11.2 | 3034 | eng | no | no | no |

|-----------------------------------------------------------------------------------------------------------------------------|

| long6 | elmiha | txtexc~h | elkig | txtexck | elmann | txtex~an | elkuon | txtsxc~n | elkuin | txtex~in | elgonz | txtex~on |

| 1_yes | 1 | | 1 | | 0 | age | 1 | | 0 | year | 1 | |

|-------+---------------------------------------------------------------------------------------------------------------------|

| elsug | txtexc~g | totEL | wasit_PG | year | PGdepy~o | PGanxy~o | depmu1~t | depmu1~e | depmu2~t | depmu2~e | depmu3~t |

| 1 | | 5 | 1 | 2011 | 1_Yes | 1_Yes | | . | | . | |

|-----------------------------------------------------------------------------+-----------+-----------+-----------+-----------|

| depmu3~e | depmu4~t | depmu4~e | anxmu1~t | anxmu1~e | anxmu2~t | anxmu2~e | anxmu3~t | anxmu3~e | anxmu4~t | anxmu4~e |

| . | | . | | . | | . | | . | | . |

|----------+----------+----------+----------+----------+----------+-----------+-----------+-----------+-----------+-----------|

| anxmu5~t | anxmu5~e | anxmu6~t | anxmu6~e | anxmu7~t | anxmu7~e | anxmu8~t | anxmu8~e | anxmu9~t | anxmu9~e | depbi1~t |

| | . | | . | | . | | . | | . | |

|----------+----------+----------+----------+----------+----------+-----------+-----------+-----------+-----------+-----------|

| depbi1~e | depbi2~t | depbi2~e | depbi3~t | depbi3~e | depbi4~t | depbi4~e | depbi5~t | depbi5~e | depbi6~t | depbi6~e |

| . | | . | | . | | . | | . | | . |

|----------+----------+----------+----------+----------+----------+-----------+-----------+-----------+-----------+-----------|

| depbi7~t | depbi7~e | depbi8~t | depbi8~e | depbi9~t | depbi9~e | anxbi1~t | anxbi1~e | anxbi2~t | anxbi2~e | anxbi3~t |

| | . | | . | . | . | | . | | . | |

|----------+----------+----------+----------+----------+----------+-----------+-----------+-----------+-----------+-----------|

| anxbi3~e | anxbi4~t | anxbi4~e | anxbi5~t | anxbi5~e | anxbi6~t | anxbi6~e | anxbi7~t | anxbi7~e | anxbi8~t | anxbi8~e |

| . | | . | | . | | . | | . | | . |

|----------+----------+----------+----------+----------+----------+-----------+-----------+-----------+-----------+-----------|

| anxbi9~t | anxbi9~e | Ld~i1txt | Ldepbi.. | Ld~i2txt | Ldepbi.. | Ld~i3txt | Ldepbi.. | Ld~i4txt | Ldepbi.. | Ld~i5txt |

| | . | | . | | . | . | . | . | . | . |

|----------+----------+----------+----------+----------+----------+-----------+-----------+-----------+-----------+-----------|

| Ldepbi.. | Lde~6txt | Ld~6vote | La~i1txt | Lanxbi.. | La~i2txt | Lanxbi.. | La~i3txt | Lanxbi.. | La~i4txt | Lanxbi.. |

| . | . | . | . | . | . | . | . | . | . | . |

|----------+----------+----------+----------+---------------------------------------------------------------------------------|

| La~i5txt | Lanxbi.. | La~i6txt | Lanxbi.. | Ldepmu1txt | Ldepmu.. | Ldepmu2txt | Ldepmu.. | Ldepmu3txt |

| . | . | . | . | StartsvNever T2 | 1 | Stops vs. Stays T3 | 1 | PG intercept to T3 |

|----------+------------------------------------------------------------------------------------------------------------------|

| Ldepmu.. | Ldepmu4txt | Ldepmu.. | Ld~u5txt | Ldepmu.. | Lanxmu1txt | Lanxmu.. | Lanxmu2txt |

| 1 | Change in PG to T3 | 1 | . | . | anx Starts v Never T2 | 1 | anx Starts vs Never T3 |

|----------+------------------------------------------------------------------------------------------------------------------|

| Lanxmu.. | Lanxmu3txt | Lanxmu.. | Lanxmu4txt | Lanxmu.. | Lanxmu5txt | Lanxmu.. |

| 1 | SocPhob Starts v Never T2 | 1 | SocPhob Starts vs Never T3 | 1 | anx StopsvStays T3 | 1 |

|-----------------------------------------------------------------------------------------------------------------------------|

| Lanxmu6txt | L~u6vote | Lanxmu7txt | La~7vote | Lanxmu8txt | La~8vote |

| SocPhob stopsvStays T2 | 1 | SocPhob Stopsvs.Stays T3 | 1 | Change in PG to T3 anx | 1 |

|-----------------------------------------------------------------------------------------------------------------------------|

| Lanxmu9txt | La~9vote | Lanxmu10txt | La~0vote | Lanxmu11txt | L~11vote | id |

| Change in PG to T3 SocPhob | 1 | PG intercept to T3anx | 1 | PG intercept to T3 SocPhob | 1 | 38 |

|-----------------------------------------------------------------------------------------------------------------------------|

| o~pyesno | o~xyesno | wasitPIU | iascale | IAdepy~o | IAanxy~o | PGscal~N | inkuon | inkuin | inking | inmihar | inmann |

| 1_Yes | 1_Yes | . | | . | . | 1 | 1 | 0 | 1 | 1 | 0 |

|-----------------------------------------------------------------------------------------------------------------------------|

| ingonz | insug | longit | depanx~e |

| 1 | 0 | 1 | |

+-----------------------------------------------------------------------------------------------------------------------------+

. list if id==47

+-----------------------------------------------------------------------------------------------------------------------------+

47. | study | notes |

| Han_Hwang_2010 | other depression meas. Assoc with tx, not comparing with control, and no longit dep/anx results |

|-----------------------------------------------------------------------------------------------------------------------------|

| GAclinpop | pgscale | ages | sampsi | lang | case | exper | neuro | long6 | elmiha | txtexc~h | elkig | txtexck |

| 1_IGD pop or mixed | YIAT | 20-21 | 19 | eng | no | no | yes | 0 | 0 | sampsi | 1 | |

|-----------------------------------------------------------------------------------------------------------------------------|

| elmann | txtex~an | elkuon | txtsxc~n | elkuin | txtex~in | elgonz | txtex~on | elsug | txtexc~g | totEL | wasit_PG | year |

| 0 | neuro | 1 | | 1 | | 0 | year | 0 | ages | 3 | 0 | 2010 |

|-----------------------------------------------------------------------------------------------------------------------------|

| PGdepy~o | PGanxy~o | depmu1~t | depmu1~e | depmu2~t | depmu2~e | depmu3~t | depmu3~e | depmu4~t | depmu4~e | anxmu1~t |

| 1_Yes | 0_No | | . | | . | | . | | . | |

|----------+----------+----------+----------+----------+----------+-----------+-----------+-----------+-----------+-----------|

| anxmu1~e | anxmu2~t | anxmu2~e | anxmu3~t | anxmu3~e | anxmu4~t | anxmu4~e | anxmu5~t | anxmu5~e | anxmu6~t | anxmu6~e |

| . | | . | | . | | . | | . | | . |

|-----------------------------------------------------------------------------------------+-----------+-----------+-----------|

| anxmu7~t | anxmu7~e | anxmu8~t | anxmu8~e | anxmu9~t | anxmu9~e | depbi1txt | depbi1~e | depbi2~t | depbi2~e |

| | . | | . | | . | BDI vs control | 0 | | . |

|-----------------------------------------------------------------------------------------+-----------+-----------+-----------|

| depbi3~t | depbi3~e | depbi4~t | depbi4~e | depbi5~t | depbi5~e | depbi6~t | depbi6~e | depbi7~t | depbi7~e | depbi8~t |

| | . | | . | | . | | . | | . | |

|----------+----------+----------+----------+----------+----------+-----------+-----------+-----------+-----------+-----------|

| depbi8~e | depbi9~t | depbi9~e | anxbi1~t | anxbi1~e | anxbi2~t | anxbi2~e | anxbi3~t | anxbi3~e | anxbi4~t | anxbi4~e |

| . | . | . | | . | | . | | . | | . |

|----------+----------+----------+----------+----------+----------+-----------+-----------+-----------+-----------+-----------|

| anxbi5~t | anxbi5~e | anxbi6~t | anxbi6~e | anxbi7~t | anxbi7~e | anxbi8~t | anxbi8~e | anxbi9~t | anxbi9~e | Ld~i1txt |

| | . | | . | | . | | . | | . | |

|----------+----------+----------+----------+----------+----------+-----------+-----------+-----------+-----------+-----------|

| Ldepbi.. | Ld~i2txt | Ldepbi.. | Ld~i3txt | Ldepbi.. | Ld~i4txt | Ldepbi.. | Ld~i5txt | Ldepbi.. | Lde~6txt | Ld~6vote |

| . | | . | . | . | . | . | . | . | . | . |

|----------+----------+----------+----------+----------+----------+-----------+-----------+-----------+-----------+-----------|

| La~i1txt | Lanxbi.. | La~i2txt | Lanxbi.. | La~i3txt | Lanxbi.. | La~i4txt | Lanxbi.. | La~i5txt | Lanxbi.. | La~i6txt |

| . | . | . | . | . | . | . | . | . | . | . |

|----------+----------+----------+----------+----------+----------+-----------+-----------+-----------+-----------+-----------|

| Lanxbi.. | Ld~u1txt | Ldepmu.. | Ld~u2txt | Ldepmu.. | Ld~u3txt | Ldepmu.. | Ld~u4txt | Ldepmu.. | Ld~u5txt | Ldepmu.. |

| . | | . | | . | | . | | . | . | . |

|----------+----------+----------+----------+----------+----------+-----------+-----------+-----------+-----------+-----------|

| La~u1txt | Lanxmu.. | La~u2txt | Lanxmu.. | La~u3txt | Lanxmu.. | La~u4txt | Lanxmu.. | La~u5txt | Lanxmu.. | La~u6txt |

| | . | | . | | . | | . | | . | |

|----------+----------+----------+----------+----------+----------+-----------------------------------------------------------|

| L~u6vote | Lan~7txt | La~7vote | Lan~8txt | La~8vote | Lan~9txt | La~9vote | Lan~0txt | La~0vote | La~11txt | L~11vote | id |

| . | | . | | . | | . | | . | | . | 47 |

|----------+----------+----------+--------------------------------------------------------------------------------------------|

| o~pyesno | o~xyesno | wasitPIU | iascale | IAdepy~o | IAanxy~o | PGscal~N | inkuon | inkuin | inking | inmihar | inmann |

| 1_Yes | 0_No | . | | . | . | 0 | 0 | 1 | 1 | 0 | 0 |

|-----------------------------------------------------------------------------------------------------------------------------|

| ingonz | insug | longit | depanx~e |

| 0 | 0 | 0 | |

+-----------------------------------------------------------------------------------------------------------------------------+

.

. *Han only report cross-sectional depression results although study is longit

. replace notes = "longit study but dep results are C-S" if id==47

(1 real change made)

. replace depbi1txt="BDI vs. controls" if id==47

(1 real change made)

. replace depbi1vote=0 if id==47

(0 real changes made)

. replace longit=0 if id==47

(0 real changes made)

.

. **some of these from the Kuss articles are just playing games or time on games

. tab PGscaleYN wasitPIU, mi

| Did the

| study

| meas. PIU?

PGscaleYN | . | Total

-----------+-----------+----------

0 | 70 | 70

1 | 114 | 114

2 | 9 | 9

. | 3 | 3

-----------+-----------+----------

Total | 196 | 196

.

. ***Lee uses the GPIUS and social anxiety

. replace PGanxyesno=1 if id==83

(0 real changes made)

.

. *Parker, Rasmussen and Thomas=2 (both)

. *Griffiths 2010b role= 3 - no scale; self-report

. *Griffiths 2010a Onling gaming add-review article, poss incorrect cite?

. *Smahel, - unclear ---emailed and they use a multiple-game scale, so PGscaleYN=1

. ***Muller is actually YSR subscales, which do not separate depression from other things

. ****HOWEVER we will count withdrawn/depressed subscale as "depressed" but not anxious/depressed subscale

. replace depanxnote="Withdrawn/depr scale of YSR counted as depr, but not anx/depr scale" if id==104

depanxnote was str1 now str67

(1 real change made)

. replace PGanxyesno=0 if id==104

(0 real changes made)

. replace PGdepyesno=1 if id==104

(1 real change made)

. replace notes="Review chapter, possibly incorrect cite on Kuss's part?" if id==41

(1 real change made)

. replace notes="scale wording unclear as to whether gaming" if id==134

(1 real change made)

. replace pgscale="none-wrong article?" if id==41

(1 real change made)

. list study id PGscaleYN if PGscaleYN==3 | PGscaleYN==4

. list study id notes pgscale PGscaleYN if PGscaleYN==3 | PGscaleYN==4

. list study notes pgscale if id==126 | id==133 | id==134 | id==41

+--------------------------------------------------------------------------------------------------------+

| study notes pgscale |

|--------------------------------------------------------------------------------------------------------|

41. | Griffiths_2010a_OG_add Review chapter, possibly incorrect cite on Kuss's part? none-wrong article? |

125. | Smahel_2008 Mult. Game scale used per email unclear, msgd |

132. | Thalemann_2004 Unclear |

133. | Thalemann_2007 scale wording unclear as to whether gaming Unclear |

+--------------------------------------------------------------------------------------------------------+

.

. *check discrepancies in PGscale

. codebook wasit_PG

------------------------------------------------------------------------------------------------------------------------------------

wasit_PG wasit_PG

------------------------------------------------------------------------------------------------------------------------------------

type: numeric (byte)

range: [0,2] units: 1

unique values: 3 missing .: 3/196

tabulation: Freq. Value

70 0

115 1

8 2

3 .

. codebook PGscaleYN

------------------------------------------------------------------------------------------------------------------------------------

PGscaleYN PGscaleYN

------------------------------------------------------------------------------------------------------------------------------------

type: numeric (byte)

range: [0,2] units: 1

unique values: 3 missing .: 3/196

tabulation: Freq. Value

70 0

114 1

9 2

3 .

. tab PGscaleYN wasit_PG, mi

| wasit_PG

PGscaleYN | 0 1 2 . | Total

-----------+--------------------------------------------+----------

0 | 70 0 0 0 | 70

1 | 0 114 0 0 | 114

2 | 0 1 8 0 | 9

. | 0 0 0 3 | 3

-----------+--------------------------------------------+----------

Total | 70 115 8 3 | 196

.

. list study id if PGscaleYN==0 & wasit_PG==1

. *Stettina

.

. list study id if PGscaleYN==1 & wasit_PG==0

. *none

.

. list study id pgscale notes wasit_PG PGscaleYN if wasit_PG==1 & PGscaleYN==0

.

. tab PGscaleYN wasit_PG, mi

| wasit_PG

PGscaleYN | 0 1 2 . | Total

-----------+--------------------------------------------+----------

0 | 70 0 0 0 | 70

1 | 0 114 0 0 | 114

2 | 0 1 8 0 | 9

. | 0 0 0 3 | 3

-----------+--------------------------------------------+----------

Total | 70 115 8 3 | 196

. list study id pgscale notes PGscaleYN wasit_PG if PGscaleYN != wasit_PG

+------------------------------------------------------------------+

| study id pgscale notes PGscal~N wasit_PG |

|------------------------------------------------------------------|

142. | VRooij_2012 143 VAT, GAS, CIUS 2 1 |

+------------------------------------------------------------------+

.

.

. ***************************************************************************

. *POST HOC RESULTS - these were only discovered after the data extraction

. * was done

.

.

. ***May 16--got paper showing Thalemann 2004 was actually PG

. *****but still no assoc with dep/anx

. list study id if strpos(study, "Thal")

+----------------------+

| study id |

|----------------------|

132. | Thalemann_2004 133 |

133. | Thalemann_2007 134 |

+----------------------+

. replace pgscale="CSVK comp games" if id==133

(1 real change made)

. replace PGscaleYN=1 if id==133

(1 real change made)

. replace wasit_PG=1 if id==133

(1 real change made)

. replace PGdepyesno=0 if id==133

(1 real change made)

. replace PGanxyesno=0 if id==133

(1 real change made)

. replace longit=0 if id==133

(1 real change made)

.

. ******Thalemann listed as missing for most reviews; replace with 0

. foreach var in inking inkuon inmann inmihar ingonz insug {

2. replace `var'=0 if id==133

3. }

(1 real change made)

(1 real change made)

(1 real change made)

(1 real change made)

(0 real changes made)

(0 real changes made)

.

.

. ****Smahel sent proof that their work was MMMORPGs, which we code as PG

. replace pgscale="mult.games" if id==126

(1 real change made)

. replace PGscaleYN=1 if id==126

(1 real change made)

.

.

. ****5/1 Coyne returned email and explained results

. *" Anxiety, PG symptoms, and stress reactivity were all measured at the initial

. *****time point. Additionally, our outcome variable (PG symptoms) was again

. *****measured at the second time point, but the other variables were not."

. ***6/2/20 replaced all non-longit with longit

. replace anxmu1vote=. if id==29

(1 real change made, 1 to missing)

. replace anxmu2vote=. if id==29

(1 real change made, 1 to missing)

. replace anxmu3vote=. if id==29

(1 real change made, 1 to missing)

. replace anxmu1txt="" if id==29

(1 real change made)

. replace anxmu2txt="" if id==29

(1 real change made)

. replace anxmu3txt="" if id==29

(1 real change made)

.

. *check and add longit results if necc

.

. **Coyne

. list if id==29

+-----------------------------------------------------------------------------------------------------------------------------+

29. | study | notes | GAclin~p | pgscale | ages | sampsi | lang | case | exper | neuro | long6 |

| Coyne_2015 | | 0_No | Adapted DSM IV TR gaming | 13-18 (M15.3) | 374 | eng | no | no | maybe | 1_yes |

|-----------------------------------------------------------------------------------------------------------------------------|

| elmiha | txtexc~h | elkig | txtexck | elmann | txtex~an | elkuon | txtsxc~n | elkuin | txtex~in | elgonz | txtex~on | elsug |

| 1 | | 0 | year | 1 | | 0 | year | 0 | year | 1 | | 1 |

|-----------------------------------------------------------------------------------------------------------------------------|

| txtexc~g | totEL | wasit_PG | year | PGdepy~o | PGanxy~o | depmu1~t | depmu1~e | depmu2~t | depmu2~e | depmu3~t | depmu3~e |

| | 4 | 1 | 2015 | 0_No | 1_Yes | | . | | . | | . |

|----------+------------------------------------------------------------------------------------------------------+-----------|

| depmu4~t | depmu4~e | anxmu1~t | anxmu1~e | anxmu2~t | anxmu2~e | anxmu3~t | anxmu3~e | anxmu4~t | anxmu4~e | anxmu5~t |

| | . | | . | | . | | . | | . | |

|----------+----------+----------+----------+----------+----------+-----------+-----------+-----------+-----------+-----------|

| anxmu5~e | anxmu6~t | anxmu6~e | anxmu7~t | anxmu7~e | anxmu8~t | anxmu8~e | anxmu9~t | anxmu9~e | depbi1~t | depbi1~e |

| . | | . | | . | | . | | . | | . |

|----------+----------+----------+----------+----------+----------+-----------+-----------+-----------+-----------+-----------|

| depbi2~t | depbi2~e | depbi3~t | depbi3~e | depbi4~t | depbi4~e | depbi5~t | depbi5~e | depbi6~t | depbi6~e | depbi7~t |

| | . | | . | | . | | . | | . | |

|----------+----------+----------+----------+----------+----------+-----------+-----------+-----------+-----------+-----------|

| depbi7~e | depbi8~t | depbi8~e | depbi9~t | depbi9~e | anxbi1~t | anxbi1~e | anxbi2~t | anxbi2~e | anxbi3~t | anxbi3~e |

| . | | . | . | . | | . | | . | | . |

|----------+----------+----------+----------+----------+----------+-----------+-----------+-----------+-----------+-----------|

| anxbi4~t | anxbi4~e | anxbi5~t | anxbi5~e | anxbi6~t | anxbi6~e | anxbi7~t | anxbi7~e | anxbi8~t | anxbi8~e | anxbi9~t |

| | . | | . | | . | | . | | . | |

|----------+----------+----------+----------+----------+----------+-----------+-----------+-----------+-----------+-----------|

| anxbi9~e | Ld~i1txt | Ldepbi.. | Ld~i2txt | Ldepbi.. | Ld~i3txt | Ldepbi.. | Ld~i4txt | Ldepbi.. | Ld~i5txt | Ldepbi.. |

| . | | . | | . | . | . | . | . | . | . |

|----------+----------+----------+----------+----------+----------+-----------+-----------+-----------+-----------+-----------|

| Lde~6txt | Ld~6vote | La~i1txt | Lanxbi.. | La~i2txt | Lanxbi.. | La~i3txt | Lanxbi.. | La~i4txt | Lanxbi.. | La~i5txt |

| . | . | . | . | . | . | . | . | . | . | . |

|----------+----------+----------+----------+----------+----------+-----------+-----------+-----------+-----------+-----------|

| Lanxbi.. | La~i6txt | Lanxbi.. | Ld~u1txt | Ldepmu.. | Ld~u2txt | Ldepmu.. | Ld~u3txt | Ldepmu.. | Ld~u4txt | Ldepmu.. |

| . | . | . | | . | | . | | . | | . |

|----------+----------+----------+----------+----------+----------+-----------+-----------+-----------+-----------+-----------|

| Ld~u5txt | Ldepmu.. | La~u1txt | Lanxmu.. | La~u2txt | Lanxmu.. | La~u3txt | Lanxmu.. | La~u4txt | Lanxmu.. | La~u5txt |

| . | . | | . | | . | | . | | . | |

|----------+----------+----------+----------+----------+----------+-----------+-----------+-----------+-----------+-----------|

| Lanxmu.. | La~u6txt | L~u6vote | Lan~7txt | La~7vote | Lan~8txt | La~8vote | Lan~9txt | La~9vote | Lan~0txt | La~0vote |

| . | | . | | . | | . | | . | | . |

|----------+----------+-------------------------------------------------------------------------------------------------------|

| La~11txt | L~11vote | id | o~pyesno | o~xyesno | wasitPIU | iascale | IAdepy~o | IAanxy~o | PGscal~N | inkuon | inkuin |

| | . | 29 | 0_No | 1_Yes | . | | . | . | 1 | 0 | 0 |

|---------------------------------------------------------------------------------------------------------+-------------------|

| inking | inmihar | inmann | ingonz | insug | longit | depanx~e |

| 0 | 0 | 1 | 0 | 0 | 1 | |

+-----------------------------------------------------------------------------------------------------------------------------+

. replace Lanxmu1txt="W5 anx on W6 # of PG sx, RSA model" if id==29

Lanxmu1txt was str21 now str34

(1 real change made)

. replace Lanxmu2txt="W5 anx on W6presence of PGsx, RSA model" if id==29

Lanxmu2txt was str22 now str39

(1 real change made)

. replace Lanxmu3txt="W5 anx on W6 # of PGsx, SC model" if id==29

Lanxmu3txt was str25 now str32

(1 real change made)

. replace Lanxmu4txt="W5 anx on W6 presence of PGsx, SC model" if id==29

Lanxmu4txt was str26 now str39

(1 real change made)

. replace Lanxmu1vote=1 if id==29

(1 real change made)

. replace Lanxmu2vote=-1 if id==29

(1 real change made)

. replace Lanxmu3vote=1 if id==29

(1 real change made)

. replace Lanxmu4vote=1 if id==29

(1 real change made)

.

.

. codebook PGscaleYN

------------------------------------------------------------------------------------------------------------------------------------

PGscaleYN PGscaleYN

------------------------------------------------------------------------------------------------------------------------------------

type: numeric (byte)

range: [0,2] units: 1

unique values: 3 missing .: 1/196

tabulation: Freq. Value

70 0

116 1

9 2

1 .

.

. list study id PGscaleYN wasitPIU pgscale if PGscale !=1 | PGscale==2

+--------------------------------------------------------------------------------------------------+

| study id PGscal~N wasitPIU pgscale |

|--------------------------------------------------------------------------------------------------|

4. | Baer _2012 4 0 . Comp/Gaming station Add scale |

5. | Baer_2011 5 0 . Comp/Gaming station Add scale |

7. | Bayrakt_2007 7 0 . YIAT |

9. | Billieux_2011 9 0 . YIAT |

14. | Caplan_2009 14 0 . GPIUS |

|--------------------------------------------------------------------------------------------------|

15. | Chan&Rab_2006 15 0 . YIAT |

16. | Chang_2008 16 0 . YIAT |

17. | Chappell_2006 17 0 . thematic |

18. | Charlt_2002 18 0 . Computer Comfort anxiety scale |

19. | Charlt_2007 19 0 . game-specific Add–Eng questionnaire |

|--------------------------------------------------------------------------------------------------|

20. | Charlt_2010 20 0 . game-specific Add–Eng questionnaire |

25. | Chuang_2006 25 0 . None |

26. | Chumbley_2006 26 0 . None |

30. | Cultura_2002 30 0 . parent report |

33. | Dong_2011 33 0 . YIAT, CIAT |

|--------------------------------------------------------------------------------------------------|

35. | Dworak_2007 35 0 . single excessive game use |

40. | Griffiths_2004 40 0 . "sacrificing other activities to play [EQ]" |

41. | Griffiths_2010a_OG_add 41 . . none-wrong article? |

42. | Griffiths_2010b_role 42 0 . self-rep Addiction v not |

44. | Grusser_2007 44 0 . unclear |

|--------------------------------------------------------------------------------------------------|

47. | Han_Hwang_2010 47 0 . YIAT |

48. | Han_Lee_2007 48 0 . IAS |

49. | Han_Lee_2009 49 0 . YIAT |

50. | Han_Lyoo_2012 50 0 . YIAS |

52. | Hawi_2012 52 0 . YIAT |

|--------------------------------------------------------------------------------------------------|

54. | Hoeft_2008 54 0 . None |

62. | Khazaal_2008 62 0 . YIAT |

69. | King_2009b_under 69 0 . 1 theme excessive playing w/ consequences |

70. | King_2010 70 0 . none |

72. | King_2013_Axis 72 2 . PTU, specific to games |

|--------------------------------------------------------------------------------------------------|

76. | Ko_2005a 76 0 . CIAS |

77. | Ko_2009 77 0 . DCIA-C |

78. | Konkoly_2015 78 2 . gaming or "int chat" as BAD |

81. | Lee_2007 82 0 . KIAT (not specific) |

82. | Lee_2015 83 0 . GPIUS + game person played |

|--------------------------------------------------------------------------------------------------|

83. | Lehenbau_2015 84 0 . WoW-specific scale based on Charlton |

90. | Leung_2004 91 0 . YIAT |

96. | Meerkerk_2006 97 0 . CIUS |

97. | Meerkerk_2010 98 0 . CIUS |

101. | Montag_2011 102 0 . IAT |

|--------------------------------------------------------------------------------------------------|

102. | Mottram_2009 103 0 . IAT |

104. | Ng_2005 105 0 . quest. that "suggested heavy overuse" |

106. | Parker_2008 107 2 . IADQ, PVGPS |

107. | Pawlikowski_2011 108 0 . IATWoW |

109. | Peters_2008 110 0 . WoW-specific, based on Charlton |

|--------------------------------------------------------------------------------------------------|

112. | Rasmussen_2015 113 2 . 3 Q abt games, 3 Q abt Internet |

113. | Rau_2006 114 0 . YIAT |

118. | Rikkers_2016 119 0 . combined games/internet |

128. | Stetina_2011 129 0 . ISS-20 |

129. | Strittmat_2015 130 0 . YDQ |

|--------------------------------------------------------------------------------------------------|

130. | Sun_2008 131 0 . none-wrong article? |

133. | Thalemann_2007 134 0 . Unclear |

134. | Thomas_2010 135 2 . adapYDQ Int, YDQ comp games |

137. | Tsitsika_2011 138 0 . YIAT |

140. | VRooij_2010_R 141 0 . CIUS |

|--------------------------------------------------------------------------------------------------|

141. | VRooij_2011 142 0 . CIUS |

142. | VRooij_2012 143 2 . VAT, GAS, CIUS |

151. | Wood_2007 152 0 . theme: experience of "time loss" |

157. | Cole_2013 158 0 . GPIUS |

163. | Hyun_2015 164 0 . YIAS |

|--------------------------------------------------------------------------------------------------|

168. | Wei_2012 169 0 . CIAS |

169. | Bae_2016 170 0 . IGD+ADHD |

171. | Cai_2016 172 2 . IGD crit, IAT |

172. | Chun_2015 173 0 . K-scale |

174. | Ding_2013 175 0 . mod YDQ, CIAS |

|--------------------------------------------------------------------------------------------------|

175. | Ding_2014 176 0 . mod YDQ |

176. | Du_2016 177 0 . YDQ+YIAT+time gaming |

177. | Feng_2013 178 0 . adapted YDQ |

178. | Han_2017 179 0 . YIAS+time |

179. | Han_dh_2012 180 0 . YIAS+time |

|--------------------------------------------------------------------------------------------------|

180. | Hong_2015 181 0 . YIAT |

181. | Hu_2017 182 0 . mod. CIAS |

182. | Jin_2016 183 2 . DSM 5 IGD+YIAT >50 |

184. | Kim_SM_2012 185 0 . YIAS |

185. | Park_2015 186 0 . Kscale+game time |

|--------------------------------------------------------------------------------------------------|

186. | Park_2016 187 2 . DSM5, YIAS |

189. | Qi_2015 190 0 . YDQ+YIAT+hpd |

191. | Wang_2015_al 192 0 . "mod" YDQ no ex |

192. | Wang_2015_dec 193 0 . mod YDQ, CIAS |

195. | Xing 196 0 . IAT |

+--------------------------------------------------------------------------------------------------+

.

.

. **based on scale names in pgscale, replace wasitPIU if scale measures IA

. replace wasitPIU=1 if ///

> id==7 | ///

> id==9 | ///

> id==14 | ///

> id==15 | ///

> id==16 | ///

> id==33 | ///

> id==47 | ///

> id==48 | ///

> id==49 | ///

> id==50 | ///

> id==52 | ///

> id==62 | ///

> id==72 | ///

> id==76 | ///

> id==77 | ///

> id==78 | ///

> id==82 | ///

> id==83 | ///

> id==91 | ///

> id==97 | ///

> id==98 | ///

> id==102 | ///

> id==103 | ///

> id==113 | ///

> id==107 | ///

> id==114 | ///

> id==129 | ///

> id==130 | ///

> id==135 | ///

> id==138 | ///

> id==141 | ///

> id==142 | ///

> id==143 | ///

> id==158 | ///

> id==164 | ///

> id==169 | ///

> id==172 | ///

> id==173 | ///

> id==175 | ///

> id==176 | ///

> id==177 | ///

> id==178 | ///

> id==179 | ///

> id==180 | ///

> id==181 | ///

> id==182 | ///

> id==183 | ///

> id==185 | ///

> id==186 | ///

> id==187 | ///

> id==190 | ///

> id==192 | ///

> id==193 | ///

> id==196

(54 real changes made)

.

.

. list study id pgscale wasitPIU

+---------------------------------------------------------------------------------------+

| study id pgscale wasitPIU |

|---------------------------------------------------------------------------------------|

1. | Achab_2011 1 DAS . |

2. | Allison_2006 2 Cinical interview . |

3. | Andreass_Sch 3 GAS, BSNAS . |

4. | Baer _2012 4 Comp/Gaming station Add scale . |

5. | Baer_2011 5 Comp/Gaming station Add scale . |

|---------------------------------------------------------------------------------------|

6. | Batthya_2009 6 CSVK-R (new) . |

7. | Bayrakt_2007 7 YIAT 1 |

8. | Beranuy_2010 8 Clinical interview . |

9. | Billieux_2011 9 YIAT 1 |

10. | Bioulac_2008 10 PVP . |

|---------------------------------------------------------------------------------------|

11. | Bouna-P 11 IGD based on CIUS . |

12. | Brunborg_2014 12 GAS . |

13. | Brunborg_2015 13 GAS . |

14. | Caplan_2009 14 GPIUS 1 |

15. | Chan&Rab_2006 15 YIAT 1 |

|---------------------------------------------------------------------------------------|

16. | Chang_2008 16 YIAT 1 |

17. | Chappell_2006 17 thematic . |

18. | Charlt_2002 18 Computer Comfort anxiety scale . |

19. | Charlt_2007 19 game-specific Add–Eng questionnaire . |

20. | Charlt_2010 20 game-specific Add–Eng questionnaire . |

|---------------------------------------------------------------------------------------|

21. | Chiou_2008 21 OAST . |

22. | Chiu_2004 22 new scale . |

23. | Choo_2010 23 Adapted DSM IV TR gaming . |

24. | Chou_2003 24 New scale . |

25. | Chuang_2006 25 None . |

|---------------------------------------------------------------------------------------|

26. | Chumbley_2006 26 None . |

27. | Coeffec_2015 27 PUVG . |

28. | Collins_2012 28 GAS . |

29. | Coyne_2015 29 Adapted DSM IV TR gaming . |

30. | Cultura_2002 30 parent report . |

|---------------------------------------------------------------------------------------|

31. | Demetrovics_2012 31 POGquestion . |

32. | Desai_2010 32 3 items sp to games . |

33. | Dong_2011 33 YIAT, CIAT 1 |

34. | Dreier_2016 34 AICA-S . |

35. | Dworak_2007 35 single excessive game use . |

|---------------------------------------------------------------------------------------|

36. | Festl_2012 36 GAS . |

37. | Gentile_2009 37 Adapted DSM IV TR . |

38. | Gentile_2011 38 Adapted DSM-IV-TR.latent classes . |

39. | Griffiths&H 39 criteria adapt. w/ "playing" . |

40. | Griffiths_2004 40 "sacrificing other activities to play [EQ]" . |

|---------------------------------------------------------------------------------------|

41. | Griffiths_2010a_OG_add 41 none-wrong article? . |

42. | Griffiths_2010b_role 42 self-rep Addiction v not . |

43. | Grusser_2005 43 CSVK . |

44. | Grusser_2007 44 unclear . |

45. | Haagsma_2012 prev 45 GAS and 3 items . |

|---------------------------------------------------------------------------------------|

46. | Haagsma_2013 TPB 46 GAS . |

47. | Han_Hwang_2010 47 YIAT 1 |

48. | Han_Lee_2007 48 IAS 1 |

49. | Han_Lee_2009 49 YIAT 1 |

50. | Han_Lyoo_2012 50 YIAS 1 |

|---------------------------------------------------------------------------------------|

51. | Hart_2009 51 PVP . |

52. | Hawi_2012 52 YIAT 1 |

53. | Henchoz_2016 53 GAS . |

54. | Hoeft_2008 54 None . |

55. | Hsue_2009 55 Chou scale . |

|---------------------------------------------------------------------------------------|

56. | Hull_2013 56 GAS . |

57. | Hussain_2009a_att 57 adap. from the Exercise Add Inv (EAI) . |

58. | Hussain_2009b_exc 58 thematic analysis-Addiction . |

59. | Hussain_2012 59 GAS . |

60. | Jeong_2011 60 YIAT "gaming" . |

|---------------------------------------------------------------------------------------|

61. | Johansson_2004 61 YIAT "playing" . |

62. | Khazaal_2008 62 YIAT 1 |

63. | Kim_2008 63 Online game Addiction scale . |

64. | Kim_2010 64 POGU . |

65. | Kim_K_2015 65 Int Game Add scale for adol . |

|---------------------------------------------------------------------------------------|

66. | Kim_NH_2016 66 Online game Add scale . |

67. | Kim_NR_2016 67 DSM 5 criteria . |

68. | King_2009a_motiv 68 PVG . |

69. | King_2009b_under 69 1 theme excessive playing w/ consequences . |

70. | King_2010 70 none . |

|---------------------------------------------------------------------------------------|

71. | King_2011 71 PVGT (also adapted . |

72. | King_2013_Axis 72 PTU, specific to games 1 |

73. | King_2013 traj 73 PVGP . |

74. | Kiraly_2014 74 POGquestion . |

75. | Kiraly_2015 75 POGquestion-SF . |

|---------------------------------------------------------------------------------------|

76. | Ko_2005a 76 CIAS 1 |

77. | Ko_2009 77 DCIA-C 1 |

78. | Konkoly_2015 78 gaming or "int chat" as BAD 1 |

79. | Kwon_2011 80 IAS (adapted to games) . |

80. | Lafreniere_2009 81 PVP . |

|---------------------------------------------------------------------------------------|

81. | Lee_2007 82 KIAT (not specific) 1 |

82. | Lee_2015 83 GPIUS + game person played 1 |

83. | Lehenbau_2015 84 WoW-specific scale based on Charlton . |

84. | Lemmens_2006 85 dsm iv adapted . |

85. | Lemmens_2009 86 DSM based criteria "playing" . |

|---------------------------------------------------------------------------------------|

86. | Lemmens_2011_agg 87 DSM based criteria "playing" . |

87. | Lemmens_2011_psyc 88 DSM based criteria "playing" . |

88. | Lemmens_2015 89 IGD scale . |

89. | Lemos_2016 90 VAT, GAS . |

90. | Leung_2004 91 YIAT 1 |

|---------------------------------------------------------------------------------------|

91. | Li_2011 92 Adapted criteria from Choo . |

92. | Liu_2009 93 new scale . |

93. | Lopez-F_2013 94 PVP . |

94. | Lu_2008 95 new scale . |

95. | Manniko_2015 96 GAS . |

|---------------------------------------------------------------------------------------|

96. | Meerkerk_2006 97 CIUS 1 |

97. | Meerkerk_2010 98 CIUS 1 |

98. | Mehroof_2010 99 GAS . |

99. | Mentzoni_2011 100 GAS . |

100. | Metcalf_2011 101 Adap Charlton's for MMORPG . |

|---------------------------------------------------------------------------------------|

101. | Montag_2011 102 IAT 1 |

102. | Mottram_2009 103 IAT 1 |

103. | Muller_2015 104 AICAS gaming . |

104. | Ng_2005 105 quest. that "suggested heavy overuse" . |

105. | Papay_2013 106 POGquestion . |

|---------------------------------------------------------------------------------------|

106. | Parker_2008 107 IADQ, PVGPS 1 |

107. | Pawlikowski_2011 108 IATWoW . |

108. | Peng_2010 109 Adapted from previous, included "gaming" . |

109. | Peters_2008 110 WoW-specific, based on Charlton . |

110. | Pontes_2014 111 IGD-20 . |

|---------------------------------------------------------------------------------------|

111. | Porter_2010 112 PVG . |

112. | Rasmussen_2015 113 3 Q abt games, 3 Q abt Internet 1 |

113. | Rau_2006 114 YIAT 1 |

114. | Ream_2011 115 PVP . |

115. | Rehbein_2010 116 KFN-CSAS-II . |

|---------------------------------------------------------------------------------------|

116. | Rehbein_2013 117 CSAS . |

117. | Rehbein_2015 118 CSAS . |

118. | Rikkers_2016 119 combined games/internet . |

119. | Salguero_2002 120 PVP . |

120. | Scharkow_2014 121 GAS . |

|---------------------------------------------------------------------------------------|

121. | Schmitt_2015 122 Revised Choo/Gentile scale . |

122. | Seah_2007 123 Adap Charlton's for gaming . |

123. | Seok_2012 124 Adap Charlton's for online gaming . |

124. | Skoric_2009 125 novel scale about VG Addiction . |

125. | Smahel_2008 126 mult.games . |

|---------------------------------------------------------------------------------------|

126. | Son_2013 127 scale from Wan w/ ex . |

127. | Starcevic_2011 128 VGU questionnaire . |

128. | Stetina_2011 129 ISS-20 1 |

129. | Strittmat_2015 130 YDQ 1 |

130. | Sun_2008 131 none-wrong article? . |

|---------------------------------------------------------------------------------------|

131. | Tejeiro_2012 132 PVGP . |

132. | Thalemann_2004 133 CSVK comp games . |

133. | Thalemann_2007 134 Unclear . |

134. | Thomas_2010 135 adapYDQ Int, YDQ comp games 1 |

135. | Tolchinsky_2011 136 PVGP . |

|---------------------------------------------------------------------------------------|

136. | Topor_2011 137 novel scale based on DSMIVTR . |

137. | Tsitsika_2011 138 YIAT 1 |

138. | Turner_2012 139 PVP . |

139. | Vadlin_2015 140 GAIT (IGD-based) . |

140. | VRooij_2010_R 141 CIUS 1 |

|---------------------------------------------------------------------------------------|

141. | VRooij_2011 142 CIUS 1 |

142. | VRooij_2012 143 VAT, GAS, CIUS 1 |

143. | Vukosc-G_2015 144 GAS . |

144. | Walther_2012 145 KFN-CSAS-II . |

145. | Wan_2006a psych 146 OAST . |

|---------------------------------------------------------------------------------------|

146. | Wan_2006b why 147 OAST . |

147. | Wan_Chiou_2007 148 OAST . |

148. | Wang_2014 149 GAS . |

149. | Wang_2015 150 GAS . |

150. | Wittek_2016 151 GAS . |

|---------------------------------------------------------------------------------------|

151. | Wood_2007 152 theme: experience of "time loss" . |

152. | Wu_2013 153 adapted YIAT "video games" . |

153. | Yee_2006a_dem 154 single item . |

154. | Yee_2006b_psych 155 4 question and qualitative . |

155. | Yu_2015 156 POGU . |

|---------------------------------------------------------------------------------------|

156. | Zhou_2009 157 OAGI (novel) . |

157. | Cole_2013 158 GPIUS 1 |

158. | Jimenez_2014 159 VDT . |

159. | King_2016 160 IGD checklist . |

160. | Laconi_2017 161 IGDT-10 . |

|---------------------------------------------------------------------------------------|

161. | Na_2017 162 IGD crit survey . |

162. | Panagiot_2017 163 PVGT . |

163. | Hyun_2015 164 YIAS 1 |

164. | Yen_2016 165 IGD crit interview . |

165. | Vadlin_5/2016 166 GAIT (IGD-based) . |

|---------------------------------------------------------------------------------------|

166. | Wang_2018 167 IGD 9 scale . |

167. | Wartbg_2017_IGD 168 IGDS . |

168. | Wei_2012 169 CIAS 1 |

169. | Bae_2016 170 IGD+ADHD . |

170. | Bonnaire_2017 171 GAS . |

|---------------------------------------------------------------------------------------|

171. | Cai_2016 172 IGD crit, IAT 1 |

172. | Chun_2015 173 K-scale 1 |

173. | Colder_carr_2018 174 AICA-S, IGD class . |

174. | Ding_2013 175 mod YDQ, CIAS 1 |

175. | Ding_2014 176 mod YDQ 1 |

|---------------------------------------------------------------------------------------|

176. | Du_2016 177 YDQ+YIAT+time gaming 1 |

177. | Feng_2013 178 adapted YDQ 1 |

178. | Han_2017 179 YIAS+time 1 |

179. | Han_dh_2012 180 YIAS+time 1 |

180. | Hong_2015 181 YIAT 1 |

|---------------------------------------------------------------------------------------|

181. | Hu_2017 182 mod. CIAS 1 |

182. | Jin_2016 183 DSM 5 IGD+YIAT >50 1 |

183. | Kim_N_2016 184 OGAS . |

184. | Kim_SM_2012 185 YIAS 1 |

185. | Park_2015 186 Kscale+game time 1 |

|---------------------------------------------------------------------------------------|

186. | Park_2016 187 DSM5, YIAS 1 |

187. | Park_2017 188 dsm 5 crit . |

188. | Pontes_2016 189 IGDS9-SF . |

189. | Qi_2015 190 YDQ+YIAT+hpd 1 |

190. | Sakuma_2016 191 DSM5 + Griffiths . |

|---------------------------------------------------------------------------------------|

191. | Wang_2015_al 192 "mod" YDQ no ex 1 |

192. | Wang_2015_dec 193 mod YDQ, CIAS 1 |

193. | Wartbg_2017 Assoc 194 IGDS . |

194. | Wong_2016 195 mod YDQ w/ ex . |

195. | Xing 196 IAT 1 |

|---------------------------------------------------------------------------------------|

196. | Yu_2016 197 IGDS . |

+---------------------------------------------------------------------------------------+

. list study id pgscale iascale PGscaleYN if PGscaleYN<3 & wasitPIU==1

+-----------------------------------------------------------------------------+

| study id pgscale iascale PGscal~N |

|-----------------------------------------------------------------------------|

7. | Bayrakt_2007 7 YIAT 0 |

9. | Billieux_2011 9 YIAT 0 |

14. | Caplan_2009 14 GPIUS 0 |

15. | Chan&Rab_2006 15 YIAT 0 |

16. | Chang_2008 16 YIAT 0 |

|-----------------------------------------------------------------------------|

33. | Dong_2011 33 YIAT, CIAT 0 |

47. | Han_Hwang_2010 47 YIAT 0 |

48. | Han_Lee_2007 48 IAS 0 |

49. | Han_Lee_2009 49 YIAT 0 |

50. | Han_Lyoo_2012 50 YIAS 0 |

|-----------------------------------------------------------------------------|

52. | Hawi_2012 52 YIAT 0 |

62. | Khazaal_2008 62 YIAT 0 |

72. | King_2013_Axis 72 PTU, specific to games 2 |

76. | Ko_2005a 76 CIAS 0 |

77. | Ko_2009 77 DCIA-C 0 |

|-----------------------------------------------------------------------------|

78. | Konkoly_2015 78 gaming or "int chat" as BAD 2 |

81. | Lee_2007 82 KIAT (not specific) 0 |

82. | Lee_2015 83 GPIUS + game person played 0 |

90. | Leung_2004 91 YIAT 0 |

96. | Meerkerk_2006 97 CIUS 0 |

|-----------------------------------------------------------------------------|

97. | Meerkerk_2010 98 CIUS 0 |

101. | Montag_2011 102 IAT 0 |

102. | Mottram_2009 103 IAT 0 |

106. | Parker_2008 107 IADQ, PVGPS 2 |

112. | Rasmussen_2015 113 3 Q abt games, 3 Q abt Internet 2 |

|-----------------------------------------------------------------------------|

113. | Rau_2006 114 YIAT 0 |

128. | Stetina_2011 129 ISS-20 0 |

129. | Strittmat_2015 130 YDQ 0 |

134. | Thomas_2010 135 adapYDQ Int, YDQ comp games 2 |

137. | Tsitsika_2011 138 YIAT 0 |

|-----------------------------------------------------------------------------|

140. | VRooij_2010_R 141 CIUS 0 |

141. | VRooij_2011 142 CIUS 0 |

142. | VRooij_2012 143 VAT, GAS, CIUS 2 |

157. | Cole_2013 158 GPIUS 0 |

163. | Hyun_2015 164 YIAS 0 |

|-----------------------------------------------------------------------------|

168. | Wei_2012 169 CIAS 0 |

171. | Cai_2016 172 IGD crit, IAT 2 |

172. | Chun_2015 173 K-scale 0 |

174. | Ding_2013 175 mod YDQ, CIAS 0 |

175. | Ding_2014 176 mod YDQ 0 |

|-----------------------------------------------------------------------------|

176. | Du_2016 177 YDQ+YIAT+time gaming 0 |

177. | Feng_2013 178 adapted YDQ 0 |

178. | Han_2017 179 YIAS+time 0 |

179. | Han_dh_2012 180 YIAS+time 0 |

180. | Hong_2015 181 YIAT 0 |

|-----------------------------------------------------------------------------|

181. | Hu_2017 182 mod. CIAS 0 |

182. | Jin_2016 183 DSM 5 IGD+YIAT >50 2 |

184. | Kim_SM_2012 185 YIAS 0 |

185. | Park_2015 186 Kscale+game time 0 |

186. | Park_2016 187 DSM5, YIAS 2 |

|-----------------------------------------------------------------------------|

189. | Qi_2015 190 YDQ+YIAT+hpd 0 |

191. | Wang_2015_al 192 "mod" YDQ no ex 0 |

192. | Wang_2015_dec 193 mod YDQ, CIAS 0 |

195. | Xing 196 IAT 0 |

+-----------------------------------------------------------------------------+

. list study id pgscale iascale PGscaleYN if PGscaleYN==1 & wasitPIU==1

.

. codebook wasitPIU

------------------------------------------------------------------------------------------------------------------------------------

wasitPIU Did the study meas. PIU?

------------------------------------------------------------------------------------------------------------------------------------

type: numeric (float)

range: [1,1] units: 1

unique values: 1 missing .: 142/196

tabulation: Freq. Value

54 1

142 .

. tab wasitPIU PGscaleYN, mi

Did the |

study | PGscaleYN

meas. PIU? | 0 1 2 . | Total

-----------+--------------------------------------------+----------

1 | 45 0 9 0 | 54

. | 25 116 0 1 | 142

-----------+--------------------------------------------+----------

Total | 70 116 9 1 | 196

.

. list study id pgscale iascale if wasitPIU==. & PGscaleYN==1

+------------------------------------------------------------------------------+

| study id pgscale iascale |

|------------------------------------------------------------------------------|

1. | Achab_2011 1 DAS |

2. | Allison_2006 2 Cinical interview |

3. | Andreass_Sch 3 GAS, BSNAS |

6. | Batthya_2009 6 CSVK-R (new) |

8. | Beranuy_2010 8 Clinical interview |

|------------------------------------------------------------------------------|

10. | Bioulac_2008 10 PVP |

11. | Bouna-P 11 IGD based on CIUS |

12. | Brunborg_2014 12 GAS |

13. | Brunborg_2015 13 GAS |

21. | Chiou_2008 21 OAST |

|------------------------------------------------------------------------------|

22. | Chiu_2004 22 new scale |

23. | Choo_2010 23 Adapted DSM IV TR gaming |

24. | Chou_2003 24 New scale |

27. | Coeffec_2015 27 PUVG |

28. | Collins_2012 28 GAS |

|------------------------------------------------------------------------------|

29. | Coyne_2015 29 Adapted DSM IV TR gaming |

31. | Demetrovics_2012 31 POGquestion |

32. | Desai_2010 32 3 items sp to games |

34. | Dreier_2016 34 AICA-S |

36. | Festl_2012 36 GAS |

|------------------------------------------------------------------------------|

37. | Gentile_2009 37 Adapted DSM IV TR |

38. | Gentile_2011 38 Adapted DSM-IV-TR.latent classes |

39. | Griffiths&H 39 criteria adapt. w/ "playing" |

43. | Grusser_2005 43 CSVK |

45. | Haagsma_2012 prev 45 GAS and 3 items |

|------------------------------------------------------------------------------|

46. | Haagsma_2013 TPB 46 GAS |

51. | Hart_2009 51 PVP |

53. | Henchoz_2016 53 GAS |

55. | Hsue_2009 55 Chou scale |

56. | Hull_2013 56 GAS |

|------------------------------------------------------------------------------|

57. | Hussain_2009a_att 57 adap. from the Exercise Add Inv (EAI) |

58. | Hussain_2009b_exc 58 thematic analysis-Addiction |

59. | Hussain_2012 59 GAS |

60. | Jeong_2011 60 YIAT "gaming" |

61. | Johansson_2004 61 YIAT "playing" |

|------------------------------------------------------------------------------|

63. | Kim_2008 63 Online game Addiction scale |

64. | Kim_2010 64 POGU |

65. | Kim_K_2015 65 Int Game Add scale for adol |

66. | Kim_NH_2016 66 Online game Add scale |

67. | Kim_NR_2016 67 DSM 5 criteria |

|------------------------------------------------------------------------------|

68. | King_2009a_motiv 68 PVG |

71. | King_2011 71 PVGT (also adapted |

73. | King_2013 traj 73 PVGP |

74. | Kiraly_2014 74 POGquestion |

75. | Kiraly_2015 75 POGquestion-SF |

|------------------------------------------------------------------------------|

79. | Kwon_2011 80 IAS (adapted to games) |

80. | Lafreniere_2009 81 PVP |

84. | Lemmens_2006 85 dsm iv adapted |

85. | Lemmens_2009 86 DSM based criteria "playing" |

86. | Lemmens_2011_agg 87 DSM based criteria "playing" |

|------------------------------------------------------------------------------|

87. | Lemmens_2011_psyc 88 DSM based criteria "playing" |

88. | Lemmens_2015 89 IGD scale |

89. | Lemos_2016 90 VAT, GAS |

91. | Li_2011 92 Adapted criteria from Choo |

92. | Liu_2009 93 new scale |

|------------------------------------------------------------------------------|

93. | Lopez-F_2013 94 PVP |

94. | Lu_2008 95 new scale |

95. | Manniko_2015 96 GAS |

98. | Mehroof_2010 99 GAS |

99. | Mentzoni_2011 100 GAS |

|------------------------------------------------------------------------------|

100. | Metcalf_2011 101 Adap Charlton's for MMORPG |

103. | Muller_2015 104 AICAS gaming |

105. | Papay_2013 106 POGquestion |

108. | Peng_2010 109 Adapted from previous, included "gaming" |

110. | Pontes_2014 111 IGD-20 |

|------------------------------------------------------------------------------|

111. | Porter_2010 112 PVG |

114. | Ream_2011 115 PVP |

115. | Rehbein_2010 116 KFN-CSAS-II |

116. | Rehbein_2013 117 CSAS |

117. | Rehbein_2015 118 CSAS |

|------------------------------------------------------------------------------|

119. | Salguero_2002 120 PVP |

120. | Scharkow_2014 121 GAS |

121. | Schmitt_2015 122 Revised Choo/Gentile scale |

122. | Seah_2007 123 Adap Charlton's for gaming |

123. | Seok_2012 124 Adap Charlton's for online gaming |

|------------------------------------------------------------------------------|

124. | Skoric_2009 125 novel scale about VG Addiction |

125. | Smahel_2008 126 mult.games |

126. | Son_2013 127 scale from Wan w/ ex |

127. | Starcevic_2011 128 VGU questionnaire |

131. | Tejeiro_2012 132 PVGP |

|------------------------------------------------------------------------------|

132. | Thalemann_2004 133 CSVK comp games |

135. | Tolchinsky_2011 136 PVGP |

136. | Topor_2011 137 novel scale based on DSMIVTR |

138. | Turner_2012 139 PVP |

139. | Vadlin_2015 140 GAIT (IGD-based) |

|------------------------------------------------------------------------------|

143. | Vukosc-G_2015 144 GAS |

144. | Walther_2012 145 KFN-CSAS-II |

145. | Wan_2006a psych 146 OAST |

146. | Wan_2006b why 147 OAST |

147. | Wan_Chiou_2007 148 OAST |

|------------------------------------------------------------------------------|

148. | Wang_2014 149 GAS |

149. | Wang_2015 150 GAS |

150. | Wittek_2016 151 GAS |

152. | Wu_2013 153 adapted YIAT "video games" |

153. | Yee_2006a_dem 154 single item |

|------------------------------------------------------------------------------|

154. | Yee_2006b_psych 155 4 question and qualitative |

155. | Yu_2015 156 POGU |

156. | Zhou_2009 157 OAGI (novel) |

158. | Jimenez_2014 159 VDT |

159. | King_2016 160 IGD checklist |

|------------------------------------------------------------------------------|

160. | Laconi_2017 161 IGDT-10 |

161. | Na_2017 162 IGD crit survey |

162. | Panagiot_2017 163 PVGT |

164. | Yen_2016 165 IGD crit interview |

165. | Vadlin_5/2016 166 GAIT (IGD-based) |

|------------------------------------------------------------------------------|

166. | Wang_2018 167 IGD 9 scale |

167. | Wartbg_2017_IGD 168 IGDS |

170. | Bonnaire_2017 171 GAS |

173. | Colder_carr_2018 174 AICA-S, IGD class |

183. | Kim_N_2016 184 OGAS |

|------------------------------------------------------------------------------|

187. | Park_2017 188 dsm 5 crit |

188. | Pontes_2016 189 IGDS9-SF |

190. | Sakuma_2016 191 DSM5 + Griffiths |

193. | Wartbg_2017 Assoc 194 IGDS |

194. | Wong_2016 195 mod YDQ w/ ex |

|------------------------------------------------------------------------------|

196. | Yu_2016 197 IGDS |

+------------------------------------------------------------------------------+

.

. ***There are many thoughts about how to characterize "games" for the purposes of

. ****PG. In this study, we have not characterized scales that measure "addiction"

. ****to a single game (WoW, Asheron's Call) as PG or PIU.

.

. replace wasitPIU=0 if strpos(pgscale, "WoW")

(3 real changes made)

. replace wasitPIU=0 if id==4 | id==5 | id==18

(3 real changes made)

.

. ***Now that that is partitioned out, change values of PGscaleYN "both" to just "yes"

. codebook PGscaleYN

------------------------------------------------------------------------------------------------------------------------------------

PGscaleYN PGscaleYN

------------------------------------------------------------------------------------------------------------------------------------

type: numeric (byte)

range: [0,2] units: 1

unique values: 3 missing .: 1/196

tabulation: Freq. Value

70 0

116 1

9 2

1 .

. list study id pgscale iascale wasitPIU PGscaleYN if PGscaleYN==2

+----------------------------------------------------------------------------------------+

| study id pgscale iascale wasitPIU PGscal~N |

|----------------------------------------------------------------------------------------|

72. | King_2013_Axis 72 PTU, specific to games 1 2 |

78. | Konkoly_2015 78 gaming or "int chat" as BAD 1 2 |

106. | Parker_2008 107 IADQ, PVGPS 1 2 |

112. | Rasmussen_2015 113 3 Q abt games, 3 Q abt Internet 1 2 |

134. | Thomas_2010 135 adapYDQ Int, YDQ comp games 1 2 |

|----------------------------------------------------------------------------------------|

142. | VRooij_2012 143 VAT, GAS, CIUS 1 2 |

171. | Cai_2016 172 IGD crit, IAT 1 2 |

182. | Jin_2016 183 DSM 5 IGD+YIAT >50 1 2 |

186. | Park_2016 187 DSM5, YIAS 1 2 |

+----------------------------------------------------------------------------------------+

.

. recode PGscaleYN 2=1

(PGscaleYN: 9 changes made)

. codebook PGscaleYN

------------------------------------------------------------------------------------------------------------------------------------

PGscaleYN PGscaleYN

------------------------------------------------------------------------------------------------------------------------------------

type: numeric (byte)

range: [0,1] units: 1

unique values: 2 missing .: 1/196

tabulation: Freq. Value

70 0

125 1

1 .

.

. ***we don't need wasit_PG anymore

. tab wasit_PG PGscaleYN, mi

| PGscaleYN

wasit_PG | 0 1 . | Total

-----------+---------------------------------+----------

0 | 70 0 0 | 70

1 | 0 116 0 | 116

2 | 0 8 0 | 8

. | 0 1 1 | 2

-----------+---------------------------------+----------

Total | 70 125 1 | 196

. list study id pgscale iascale wasitPIU PGscaleYN if PGscaleYN==0 & wasit_PG==1

. list study id pgscale iascale wasitPIU PGscaleYN if PGscaleYN==1 & wasit_PG==2

+----------------------------------------------------------------------------------------+

| study id pgscale iascale wasitPIU PGscal~N |

|----------------------------------------------------------------------------------------|

72. | King_2013_Axis 72 PTU, specific to games 1 1 |

78. | Konkoly_2015 78 gaming or "int chat" as BAD 1 1 |

106. | Parker_2008 107 IADQ, PVGPS 1 1 |

112. | Rasmussen_2015 113 3 Q abt games, 3 Q abt Internet 1 1 |

134. | Thomas_2010 135 adapYDQ Int, YDQ comp games 1 1 |

|----------------------------------------------------------------------------------------|

171. | Cai_2016 172 IGD crit, IAT 1 1 |

182. | Jin_2016 183 DSM 5 IGD+YIAT >50 1 1 |

186. | Park_2016 187 DSM5, YIAS 1 1 |

+----------------------------------------------------------------------------------------+

.

. drop wasit_PG

.

.

. codebook PGdepyesno

------------------------------------------------------------------------------------------------------------------------------------

PGdepyesno New PGdep without 3 or2

------------------------------------------------------------------------------------------------------------------------------------

type: numeric (byte)

label: yesnoPG

range: [0,1] units: 1

unique values: 2 missing .: 1/196

tabulation: Freq. Numeric Label

151 0 0_No

44 1 1_Yes

1 .

.

. list study id PGdepyesno PGanxyesno pgscale wasitPIU if id==1 | id==3 | id==11 | id==72 ///

> | id==74 | id==78 | id==106 | id==107 | id==113 | id==135 | id==143 | id==149 | id==150 ///

> | id==172 | id==183 | id==187

+-----------------------------------------------------------------------------------------+

| study id PGdepy~o PGanxy~o pgscale wasitPIU |

|-----------------------------------------------------------------------------------------|

1. | Achab_2011 1 0_No 1_Yes DAS . |

3. | Andreass_Sch 3 1_Yes 1_Yes GAS, BSNAS . |

11. | Bouna-P 11 1_Yes 1_Yes IGD based on CIUS . |

72. | King_2013_Axis 72 1_Yes 1_Yes PTU, specific to games 1 |

74. | Kiraly_2014 74 1_Yes 0_No POGquestion . |

|-----------------------------------------------------------------------------------------|

78. | Konkoly_2015 78 0_No 0_No gaming or "int chat" as BAD 1 |

105. | Papay_2013 106 1_Yes 0_No POGquestion . |

106. | Parker_2008 107 0_No 0_No IADQ, PVGPS 1 |

112. | Rasmussen_2015 113 0_No 0_No 3 Q abt games, 3 Q abt Internet 1 |

134. | Thomas_2010 135 0_No 0_No adapYDQ Int, YDQ comp games 1 |

|-----------------------------------------------------------------------------------------|

142. | VRooij_2012 143 1_Yes 1_Yes VAT, GAS, CIUS 1 |

148. | Wang_2014 149 0_No 0_No GAS . |

149. | Wang_2015 150 0_No 0_No GAS . |

171. | Cai_2016 172 0_No 0_No IGD crit, IAT 1 |

182. | Jin_2016 183 0_No 0_No DSM 5 IGD+YIAT >50 1 |

|-----------------------------------------------------------------------------------------|

186. | Park_2016 187 0_No 0_No DSM5, YIAS 1 |

+-----------------------------------------------------------------------------------------+

.

.

. *****Only for the studies meas. anx/dep and PG/PIU, input the info re: anx/dep results

.

. ***The following measured both PIU and PG compared to anx/dep: Andreasson (id=3), Bouna-Pyrrou (11), King(72),

. *****Kiraly 2014-depression only-(74),

. *****van Rooij 2012 (143)

. **Andreasson meas.s both and compare them head to head

. replace iascale="BSNAS" if id==3

iascale was str1 now str5

(1 real change made)

. replace wasitPIU=1 if id==3

(1 real change made)

.

. replace IAdepyesno=1 if id==3

(1 real change made)

. replace IAanxyesno=1 if id==3

(1 real change made)

.

.

. **Bouna Pyrrou meas.s both and compare head to head

. replace iascale="SND: adapted criteria from IGD" if id==11

iascale was str5 now str30

(1 real change made)

. replace wasitPIU=1 if id==11

(1 real change made)

. replace IAdepyesno=1 if id==11

(1 real change made)

. replace IAanxyesno=1 if id==11

(1 real change made)

.

. **King meas.s both and compare head to head

. replace wasitPIU=1 if id==72

(0 real changes made)

. replace iascale="PTU: Internet" if id==72

(1 real change made)

. replace IAdepyesno=1 if id==72

(1 real change made)

. replace IAanxyesno=1 if id==72

(1 real change made)

.

. **van Rooij 2012-id==143

. replace wasitPIU=1 if id==143

(0 real changes made)

. replace iascale="CIUS" if id==143

(1 real change made)

. replace IAdepyesno=1 if id==143

(1 real change made)

. replace IAanxyesno=1 if id==143

(1 real change made)

.

. ** Kiraly 2014

. replace wasitPIU=1 if id==74

(1 real change made)

. replace iascale="PIUQ-6" if id==74

(1 real change made)

. replace IAdepyesno=1 if id==74

(1 real change made)

.

. ****These measured PIU and PG but didn't link to dep/anx

. *****Achab (nonspecific measure), Konkoly-Thege, Parker, Rasmussen, Thomas,

. *****Wang 2014, Wang 2015, Cai 2016, Jin 2016, Park 2016

. *Achab

. replace iascale="GIAD, ISS" if id==1

(1 real change made)

. replace wasitPIU=1 if id==1

(1 real change made)

. replace IAdepyesno=0 if id==1

(1 real change made)

. replace IAanxyesno=0 if id==1

(1 real change made)

. replace depanxnote="SingleQ 'more sad'=not dep" if id==1

(1 real change made)

.

. ***Konkoly-Thege

. replace iascale="single Q" if id==78

(1 real change made)

. replace wasitPIU=1 if id==78

(0 real changes made)

. replace IAdepyesno=0 if id==78

(1 real change made)

. replace IAanxyesno=0 if id==78

(1 real change made)

.

. ***Parker

. replace iascale="YIADQ" if id==147

(1 real change made)

. replace wasitPIU=1 if id==147

(1 real change made)

. replace IAdepyesno=0 if id==147

(1 real change made)

. replace IAanxyesno=0 if id==147

(1 real change made)

.

. ***Rasumussen,

. replace iascale="YDQ" if id==113

(1 real change made)

. replace wasitPIU=1 if id==113

(0 real changes made)

. replace IAdepyesno=0 if id==113

(1 real change made)

. replace IAanxyesno=0 if id==113

(1 real change made)

.

. ***Thomas

. replace iascale="YIADQ" if id==135

(1 real change made)

. replace wasitPIU=1 if id==135

(0 real changes made)

. replace IAdepyesno=0 if id==135

(1 real change made)

. replace IAanxyesno=0 if id==135

(1 real change made)

.

. ***Wang 2014

. replace iascale="IAT" if id==149

(1 real change made)

. replace wasitPIU=1 if id==149

(1 real change made)

. replace IAdepyesno=0 if id==149

(1 real change made)

. replace IAanxyesno=0 if id==149

(1 real change made)

.

. ***Wang 2015

. replace iascale="IAT" if id==150

(1 real change made)

. replace wasitPIU=1 if id==150

(1 real change made)

. replace IAdepyesno=0 if id==150

(1 real change made)

. replace IAanxyesno=0 if id==150

(1 real change made)

.

. ***Cai 2016

. replace iascale="IAT" if id==172

(1 real change made)

. replace wasitPIU=1 if id==172

(0 real changes made)

. replace IAdepyesno=0 if id==172

(1 real change made)

. replace IAanxyesno=0 if id==172

(1 real change made)

.

. ***Jin 2016

. replace iascale="YIAT" if id==183

(1 real change made)

. replace wasitPIU=1 if id==183

(0 real changes made)

. replace IAdepyesno=0 if id==183

(1 real change made)

. replace IAanxyesno=0 if id==183

(1 real change made)

.

. ***Park 2016

. replace iascale="YIAS" if id==187

(1 real change made)

. replace wasitPIU=1 if id==187

(0 real changes made)

. replace IAdepyesno=0 if id==187

(1 real change made)

. replace IAanxyesno=0 if id==187

(1 real change made)

.

. *****some results from PG anx/dep actually are IA b/c scale measures IA

. list study id PGdepyesno PGanxyesno PGscaleYN wasitPIU if PGscaleYN==0 & wasitPIU==1 ///

> & (PGdepyesno==1 | PGanxyesno==1)

+------------------------------------------------------------------+

| study id PGdepy~o PGanxy~o PGscal~N wasitPIU |

|------------------------------------------------------------------|

7. | Bayrakt_2007 7 1_Yes 0_No 0 1 |

14. | Caplan_2009 14 1_Yes 1_Yes 0 1 |

47. | Han_Hwang_2010 47 1_Yes 0_No 0 1 |

50. | Han_Lyoo_2012 50 1_Yes 0_No 0 1 |

82. | Lee_2015 83 0_No 1_Yes 0 1 |

|------------------------------------------------------------------|

97. | Meerkerk_2010 98 1_Yes 0_No 0 1 |

101. | Montag_2011 102 1_Yes 0_No 0 1 |

141. | VRooij_2011 142 1_Yes 1_Yes 0 1 |

163. | Hyun_2015 164 1_Yes 1_Yes 0 1 |

172. | Chun_2015 173 1_Yes 0_No 0 1 |

|------------------------------------------------------------------|

174. | Ding_2013 175 1_Yes 1_Yes 0 1 |

175. | Ding_2014 176 1_Yes 1_Yes 0 1 |

184. | Kim_SM_2012 185 1_Yes 0_No 0 1 |

192. | Wang_2015_dec 193 1_Yes 1_Yes 0 1 |

+------------------------------------------------------------------+

.

. ***** Bayraktar, Caplan, Han 2010, Han Lyoo, Meerkerk 2010, Montag, van Rooij

. ********8/27 added new results from Gonzalez review i.e.

. *********Cole, Wei, Hyun,

. *********2020 added new ones from Sugaya, i.e. Chun 2015 Ding 2013 Ding 2014

. *********Kin DM 2012 Wang 2015 dec

. ****Kim SM_2012

. replace iascale="YIAS" if id==185

(1 real change made)

. replace wasitPIU=1 if id==185

(0 real changes made)

. replace IAdepyesno=1 if id==185

(1 real change made)

. replace IAanxyesno=0 if id==185

(1 real change made)

.

. ****Wang 2015 dec

. replace iascale="mod YDQ, CIAS" if id==193

(1 real change made)

. replace wasitPIU=1 if id==193

(0 real changes made)

. replace IAdepyesno=1 if id==193

(1 real change made)

. replace IAanxyesno=1 if id==193

(1 real change made)

.

. ****Chun 2015

. replace iascale="Kscale" if id==173

(1 real change made)

. replace wasitPIU=1 if id==173

(0 real changes made)

. replace IAdepyesno=1 if id==173

(1 real change made)

. replace IAanxyesno=0 if id==173

(1 real change made)

.

. ****Ding 2013

. replace iascale="mod YDQ, CIAS" if id==175

(1 real change made)

. replace wasitPIU=1 if id==175

(0 real changes made)

. replace IAdepyesno=1 if id==175

(1 real change made)

. replace IAanxyesno=1 if id==175

(1 real change made)

.

. ****Ding 2014

. replace iascale="mod YDQ" if id==176

(1 real change made)

. replace wasitPIU=1 if id==176

(0 real changes made)

. replace IAdepyesno=1 if id==176

(1 real change made)

. replace IAanxyesno=1 if id==176

(1 real change made)

.

. ***Cole

. replace iascale="GPIUS" if id==158

(1 real change made)

. replace wasitPIU=1 if id==158

(0 real changes made)

. replace IAdepyesno=0 if id==158

(1 real change made)

. replace IAanxyesno=1 if id==158

(1 real change made)

.

. ***Wei

. replace iascale="CIAS" if id==169

(1 real change made)

. replace wasitPIU=1 if id==169

(0 real changes made)

. replace IAdepyesno=0 if id==169

(1 real change made)

. replace depanxnote= "reports the DSSS but only the combined (not depr subscale)" if id==169

(1 real change made)

. replace IAanxyesno=1 if id==169

(1 real change made)

.

. ***Hyun

. replace iascale="YIAS" if id==164

(1 real change made)

. replace wasitPIU=1 if id==164

(0 real changes made)

. replace IAdepyesno=1 if id==164

(1 real change made)

. replace IAanxyesno=1 if id==164

(1 real change made)

.

. ***Bayraktar

. replace iascale="YIAT" if id==7

(1 real change made)

. replace wasitPIU=1 if id==7

(0 real changes made)

. replace IAdepyesno=1 if id==7

(1 real change made)

. replace IAanxyesno=0 if id==7

(1 real change made)

.

. ***Caplan

. replace iascale="GPIUS" if id==14

(1 real change made)

. replace wasitPIU=1 if id==14

(0 real changes made)

. replace IAdepyesno=1 if id==14

(1 real change made)

. replace IAanxyesno=1 if id==14

(1 real change made)

.

. ***Han 2010

. replace iascale="YIAT" if id==47

(1 real change made)

. replace wasitPIU=1 if id==47

(0 real changes made)

. replace IAdepyesno=1 if id==47

(1 real change made)

. replace IAanxyesno=0 if id==47

(1 real change made)

.

. ***Han Lyoo 2012

. replace iascale="YIAS" if id==50

(1 real change made)

. replace wasitPIU=1 if id==50

(0 real changes made)

. replace IAdepyesno=1 if id==50

(1 real change made)

. replace IAanxyesno=0 if id==50

(1 real change made)

.

. ***Meerkerk

. replace iascale="CIUS" if id==98

(1 real change made)

. replace wasitPIU=1 if id==98

(0 real changes made)

. replace IAdepyesno=1 if id==98

(1 real change made)

. replace IAanxyesno=0 if id==98

(1 real change made)

.

. ***Montag

. replace iascale="IAT" if id==102

(1 real change made)

. replace wasitPIU=1 if id==102

(0 real changes made)

. replace IAdepyesno=1 if id==102

(1 real change made)

. replace IAanxyesno=0 if id==102

(1 real change made)

.

. ***van Rooij 2011

. replace iascale="CIUS" if id==142

(1 real change made)

. replace wasitPIU=1 if id==142

(0 real changes made)

. replace IAdepyesno=1 if id==142

(1 real change made)

. replace IAanxyesno=1 if id==142

(1 real change made)

.

. ***

. replace IAanxyesno=0 if wasitPIU==0

(6 real changes made)

. *6 changes

. replace IAdepyesno=0 if wasitPIU==0

(6 real changes made)

. *6 changes

. bys wasitPIU: list study id pgscale PGdepyesno PGanxyesno PGscaleYN if ///

> (PGanxyesno==1) & (PGscaleYN==0)

------------------------------------------------------------------------------------------------------------------------------------

-> wasitPIU = 0

------------------------------------------------------------------------------------------------------------------------------------

-> wasitPIU = 1

+------------------------------------------------------------------------------------+

| study id pgscale PGdepy~o PGanxy~o PGscal~N |

|------------------------------------------------------------------------------------|

9. | Lee_2015 83 GPIUS + game person played 0_No 1_Yes 0 |

12. | Wang_2015_dec 193 mod YDQ, CIAS 1_Yes 1_Yes 0 |

35. | Hyun_2015 164 YIAS 1_Yes 1_Yes 0 |

38. | Caplan_2009 14 GPIUS 1_Yes 1_Yes 0 |

51. | Ding_2014 176 mod YDQ 1_Yes 1_Yes 0 |

|------------------------------------------------------------------------------------|

52. | VRooij_2011 142 CIUS 1_Yes 1_Yes 0 |

58. | Ding_2013 175 mod YDQ, CIAS 1_Yes 1_Yes 0 |

+------------------------------------------------------------------------------------+

------------------------------------------------------------------------------------------------------------------------------------

-> wasitPIU = .

. bys wasitPIU: list study id pgscale PGdepyesno PGanxyesno PGscaleYN if ///

> (PGdepyesno==1 | PGdepyesno==2) & (PGscaleYN==0)

------------------------------------------------------------------------------------------------------------------------------------

-> wasitPIU = 0

------------------------------------------------------------------------------------------------------------------------------------

-> wasitPIU = 1

+-----------------------------------------------------------------------+

| study id pgscale PGdepy~o PGanxy~o PGscal~N |

|-----------------------------------------------------------------------|

12. | Wang_2015_dec 193 mod YDQ, CIAS 1_Yes 1_Yes 0 |

22. | Bayrakt_2007 7 YIAT 1_Yes 0_No 0 |

28. | Han_Lyoo_2012 50 YIAS 1_Yes 0_No 0 |

31. | Chun_2015 173 K-scale 1_Yes 0_No 0 |

32. | Han_Hwang_2010 47 YIAT 1_Yes 0_No 0 |

|-----------------------------------------------------------------------|

35. | Hyun_2015 164 YIAS 1_Yes 1_Yes 0 |

38. | Caplan_2009 14 GPIUS 1_Yes 1_Yes 0 |

42. | Kim_SM_2012 185 YIAS 1_Yes 0_No 0 |

43. | Meerkerk_2010 98 CIUS 1_Yes 0_No 0 |

47. | Montag_2011 102 IAT 1_Yes 0_No 0 |

|-----------------------------------------------------------------------|

51. | Ding_2014 176 mod YDQ 1_Yes 1_Yes 0 |

52. | VRooij_2011 142 CIUS 1_Yes 1_Yes 0 |

58. | Ding_2013 175 mod YDQ, CIAS 1_Yes 1_Yes 0 |

+-----------------------------------------------------------------------+

------------------------------------------------------------------------------------------------------------------------------------

-> wasitPIU = .

. list study id IAanx* PGscaleYN wasitPIU if IAanxyesno !=0 & wasitPIU==0

. list study id IAdep* PGscaleYN wasitPIU if IAdepyesno !=0 & wasitPIU==0

.

. ***cleaning up

. ***Command decisions.***If the scale is unclear, make sure there is no value for PGscaleYN or wasitPIU

. list id study pgscale PGscaleYN if strpos(pgscale, "Unclear") | strpos(pgscale, "unclear") | strpos(pgscale, "None") | strpos(pgsc

> ale, "none")

+---------------------------------------------------------------+

| id study pgscale PGscal~N |

|---------------------------------------------------------------|

103. | 70 King_2010 none 0 |

119. | 25 Chuang_2006 None 0 |

124. | 54 Hoeft_2008 None 0 |

130. | 131 Sun_2008 none-wrong article? 0 |

162. | 44 Grusser_2007 unclear 0 |

|---------------------------------------------------------------|

170. | 41 Griffiths_2010a_OG_add none-wrong article? . |

173. | 134 Thalemann_2007 Unclear 0 |

184. | 26 Chumbley_2006 None 0 |

+---------------------------------------------------------------+

.

. replace PGscaleYN=. if strpos(pgscale, "Unclear") | strpos(pgscale, "unclear") | strpos(pgscale, "None") | strpos(pgscale, "none")

(7 real changes made, 7 to missing)

. *****7 changes to missing

. list study id iascale pgscale wasitPIU PGscaleYN if wasitPIU==1 & PGscaleYN==0

+------------------------------------------------------------------------------------------+

| study id iascale pgscale wasitPIU PGscal~N |

|------------------------------------------------------------------------------------------|

8. | Mottram_2009 103 IAT 1 0 |

9. | Chang_2008 16 YIAT 1 0 |

10. | Strittmat_2015 130 YDQ 1 0 |

11. | Stetina_2011 129 ISS-20 1 0 |

12. | Khazaal_2008 62 YIAT 1 0 |

|------------------------------------------------------------------------------------------|

13. | Han_Lee_2007 48 IAS 1 0 |

15. | Lee_2015 83 GPIUS + game person played 1 0 |

18. | Wang_2015_dec 193 mod YDQ, CIAS mod YDQ, CIAS 1 0 |

20. | Lee_2007 82 KIAT (not specific) 1 0 |

21. | Dong_2011 33 YIAT, CIAT 1 0 |

|------------------------------------------------------------------------------------------|

22. | Ko_2005a 76 CIAS 1 0 |

24. | Chan&Rab_2006 15 YIAT 1 0 |

25. | Hu_2017 182 mod. CIAS 1 0 |

26. | Meerkerk_2006 97 CIUS 1 0 |

27. | Qi_2015 190 YDQ+YIAT+hpd 1 0 |

|------------------------------------------------------------------------------------------|

28. | Bayrakt_2007 7 YIAT YIAT 1 0 |

30. | Rau_2006 114 YIAT 1 0 |

31. | Wei_2012 169 CIAS CIAS 1 0 |

32. | Han_2017 179 YIAS+time 1 0 |

33. | Billieux_2011 9 YIAT 1 0 |

|------------------------------------------------------------------------------------------|

34. | Han_Lyoo_2012 50 YIAS YIAS 1 0 |

35. | Leung_2004 91 YIAT 1 0 |

37. | Chun_2015 173 Kscale K-scale 1 0 |

38. | Han_Hwang_2010 47 YIAT YIAT 1 0 |

40. | Feng_2013 178 adapted YDQ 1 0 |

|------------------------------------------------------------------------------------------|

41. | Hyun_2015 164 YIAS YIAS 1 0 |

43. | Wang_2015_al 192 "mod" YDQ no ex 1 0 |

44. | Caplan_2009 14 GPIUS GPIUS 1 0 |

45. | VRooij_2010_R 141 CIUS 1 0 |

48. | Kim_SM_2012 185 YIAS YIAS 1 0 |

|------------------------------------------------------------------------------------------|

49. | Meerkerk_2010 98 CIUS CIUS 1 0 |

50. | Han_dh_2012 180 YIAS+time 1 0 |

51. | Hong_2015 181 YIAT 1 0 |

52. | Han_Lee_2009 49 YIAT 1 0 |

53. | Montag_2011 102 IAT IAT 1 0 |

|------------------------------------------------------------------------------------------|

55. | Cole_2013 158 GPIUS GPIUS 1 0 |

56. | Tsitsika_2011 138 YIAT 1 0 |

57. | Ding_2014 176 mod YDQ mod YDQ 1 0 |

58. | VRooij_2011 142 CIUS CIUS 1 0 |

59. | Park_2015 186 Kscale+game time 1 0 |

|------------------------------------------------------------------------------------------|

63. | Ko_2009 77 DCIA-C 1 0 |

64. | Ding_2013 175 mod YDQ, CIAS mod YDQ, CIAS 1 0 |

65. | Hawi_2012 52 YIAT 1 0 |

66. | Xing 196 IAT 1 0 |

67. | Du_2016 177 YDQ+YIAT+time gaming 1 0 |

+------------------------------------------------------------------------------------------+

. ***all missing iascales have IA scales in pgscale var

. replace iascale=pgscale if wasitPIU==1 & PGscaleYN==0

(31 real changes made)

. ***26 studies

.

. list study id pgscale iascale wasitPIU PGscaleYN if wasitPIU==1 & PGscaleYN==0

+--------------------------------------------------------------------------------------------------------+

| study id pgscale iascale wasitPIU PGscal~N |

|--------------------------------------------------------------------------------------------------------|

8. | Mottram_2009 103 IAT IAT 1 0 |

9. | Chang_2008 16 YIAT YIAT 1 0 |

10. | Strittmat_2015 130 YDQ YDQ 1 0 |

11. | Stetina_2011 129 ISS-20 ISS-20 1 0 |

12. | Khazaal_2008 62 YIAT YIAT 1 0 |

|--------------------------------------------------------------------------------------------------------|

13. | Han_Lee_2007 48 IAS IAS 1 0 |

15. | Lee_2015 83 GPIUS + game person played GPIUS + game person played 1 0 |

18. | Wang_2015_dec 193 mod YDQ, CIAS mod YDQ, CIAS 1 0 |

20. | Lee_2007 82 KIAT (not specific) KIAT (not specific) 1 0 |

21. | Dong_2011 33 YIAT, CIAT YIAT, CIAT 1 0 |

|--------------------------------------------------------------------------------------------------------|

22. | Ko_2005a 76 CIAS CIAS 1 0 |

24. | Chan&Rab_2006 15 YIAT YIAT 1 0 |

25. | Hu_2017 182 mod. CIAS mod. CIAS 1 0 |

26. | Meerkerk_2006 97 CIUS CIUS 1 0 |

27. | Qi_2015 190 YDQ+YIAT+hpd YDQ+YIAT+hpd 1 0 |

|--------------------------------------------------------------------------------------------------------|

28. | Bayrakt_2007 7 YIAT YIAT 1 0 |

30. | Rau_2006 114 YIAT YIAT 1 0 |

31. | Wei_2012 169 CIAS CIAS 1 0 |

32. | Han_2017 179 YIAS+time YIAS+time 1 0 |

33. | Billieux_2011 9 YIAT YIAT 1 0 |

|--------------------------------------------------------------------------------------------------------|

34. | Han_Lyoo_2012 50 YIAS YIAS 1 0 |

35. | Leung_2004 91 YIAT YIAT 1 0 |

37. | Chun_2015 173 K-scale K-scale 1 0 |

38. | Han_Hwang_2010 47 YIAT YIAT 1 0 |

40. | Feng_2013 178 adapted YDQ adapted YDQ 1 0 |

|--------------------------------------------------------------------------------------------------------|

41. | Hyun_2015 164 YIAS YIAS 1 0 |

43. | Wang_2015_al 192 "mod" YDQ no ex "mod" YDQ no ex 1 0 |

44. | Caplan_2009 14 GPIUS GPIUS 1 0 |

45. | VRooij_2010_R 141 CIUS CIUS 1 0 |

48. | Kim_SM_2012 185 YIAS YIAS 1 0 |

|--------------------------------------------------------------------------------------------------------|

49. | Meerkerk_2010 98 CIUS CIUS 1 0 |

50. | Han_dh_2012 180 YIAS+time YIAS+time 1 0 |

51. | Hong_2015 181 YIAT YIAT 1 0 |

52. | Han_Lee_2009 49 YIAT YIAT 1 0 |

53. | Montag_2011 102 IAT IAT 1 0 |

|--------------------------------------------------------------------------------------------------------|

55. | Cole_2013 158 GPIUS GPIUS 1 0 |

56. | Tsitsika_2011 138 YIAT YIAT 1 0 |

57. | Ding_2014 176 mod YDQ mod YDQ 1 0 |

58. | VRooij_2011 142 CIUS CIUS 1 0 |

59. | Park_2015 186 Kscale+game time Kscale+game time 1 0 |

|--------------------------------------------------------------------------------------------------------|

63. | Ko_2009 77 DCIA-C DCIA-C 1 0 |

64. | Ding_2013 175 mod YDQ, CIAS mod YDQ, CIAS 1 0 |

65. | Hawi_2012 52 YIAT YIAT 1 0 |

66. | Xing 196 IAT IAT 1 0 |

67. | Du_2016 177 YDQ+YIAT+time gaming YDQ+YIAT+time gaming 1 0 |

+--------------------------------------------------------------------------------------------------------+

. list study id pgscale iascale wasitPIU PGscaleYN if wasitPIU==1 & PGscaleYN==1

+---------------------------------------------------------------------------------------------------------------+

| study id pgscale iascale wasitPIU PGscal~N |

|---------------------------------------------------------------------------------------------------------------|

7. | Park_2016 187 DSM5, YIAS YIAS 1 1 |

14. | Andreass_Sch 3 GAS, BSNAS BSNAS 1 1 |

16. | Konkoly_2015 78 gaming or "int chat" as BAD single Q 1 1 |

17. | King_2013_Axis 72 PTU, specific to games PTU: Internet 1 1 |

19. | Wang_2014 149 GAS IAT 1 1 |

|---------------------------------------------------------------------------------------------------------------|

23. | Kiraly_2014 74 POGquestion PIUQ-6 1 1 |

29. | Thomas_2010 135 adapYDQ Int, YDQ comp games YIADQ 1 1 |

36. | Cai_2016 172 IGD crit, IAT IAT 1 1 |

39. | Rasmussen_2015 113 3 Q abt games, 3 Q abt Internet YDQ 1 1 |

42. | VRooij_2012 143 VAT, GAS, CIUS CIUS 1 1 |

|---------------------------------------------------------------------------------------------------------------|

46. | Bouna-P 11 IGD based on CIUS SND: adapted criteria from IGD 1 1 |

47. | Parker_2008 107 IADQ, PVGPS 1 1 |

54. | Jin_2016 183 DSM 5 IGD+YIAT >50 YIAT 1 1 |

60. | Wan_2006b why 147 OAST YIADQ 1 1 |

61. | Achab_2011 1 DAS GIAD, ISS 1 1 |

|---------------------------------------------------------------------------------------------------------------|

62. | Wang_2015 150 GAS IAT 1 1 |

+---------------------------------------------------------------------------------------------------------------+

. list study id pgscale iascale wasitPIU PGscaleYN if wasitPIU==1 & PGscaleYN==.

.

. bys PGscaleYN: list study id iascale wasitPIU pgscale PGscaleYN if wasitPIU==0 | wasitPIU==.

------------------------------------------------------------------------------------------------------------------------------------

-> PGscaleYN = 0

+----------------------------------------------------------------------------------------------------------+

| study id iascale wasitPIU pgscale PGscal~N |

|----------------------------------------------------------------------------------------------------------|

2. | Griffiths_2004 40 . "sacrificing other activities to play [EQ]" 0 |

6. | Ng_2005 105 . quest. that "suggested heavy overuse" 0 |

7. | Chappell_2006 17 . thematic 0 |

8. | Cultura_2002 30 . parent report 0 |

27. | Dworak_2007 35 . single excessive game use 0 |

|----------------------------------------------------------------------------------------------------------|

28. | Charlt_2002 18 0 Computer Comfort anxiety scale 0 |

39. | Peters_2008 110 0 WoW-specific, based on Charlton 0 |

42. | Charlt_2010 20 . game-specific Add–Eng questionnaire 0 |

43. | Bae_2016 170 . IGD+ADHD 0 |

47. | Wood_2007 152 . theme: experience of "time loss" 0 |

|----------------------------------------------------------------------------------------------------------|

49. | Rikkers_2016 119 . combined games/internet 0 |

51. | Lehenbau_2015 84 0 WoW-specific scale based on Charlton 0 |

52. | Pawlikowski_2011 108 0 IATWoW 0 |

53. | Baer _2012 4 0 Comp/Gaming station Add scale 0 |

56. | King_2009b_under 69 . 1 theme excessive playing w/ consequences 0 |

|----------------------------------------------------------------------------------------------------------|

58. | Charlt_2007 19 . game-specific Add–Eng questionnaire 0 |

60. | Baer_2011 5 0 Comp/Gaming station Add scale 0 |

61. | Griffiths_2010b_role 42 . self-rep Addiction v not 0 |

+----------------------------------------------------------------------------------------------------------+

------------------------------------------------------------------------------------------------------------------------------------

-> PGscaleYN = 1

+----------------------------------------------------------------------------------------------------+

| study id iascale wasitPIU pgscale PGscal~N |

|----------------------------------------------------------------------------------------------------|

1. | Walther_2012 145 . KFN-CSAS-II 1 |

3. | Yu_2016 197 . IGDS 1 |

4. | Wittek_2016 151 . GAS 1 |

5. | Pontes_2016 189 . IGDS9-SF 1 |

6. | Kim_NH_2016 66 . Online game Add scale 1 |

|----------------------------------------------------------------------------------------------------|

7. | Lemmens_2011_psyc 88 . DSM based criteria "playing" 1 |

8. | Lopez-F_2013 94 . PVP 1 |

9. | Son_2013 127 . scale from Wan w/ ex 1 |

10. | Yu_2015 156 . POGU 1 |

11. | Beranuy_2010 8 . Clinical interview 1 |

|----------------------------------------------------------------------------------------------------|

12. | Hussain_2009a_att 57 . adap. from the Exercise Add Inv (EAI) 1 |

13. | Lafreniere_2009 81 . PVP 1 |

14. | Ream_2011 115 . PVP 1 |

15. | Papay_2013 106 . POGquestion 1 |

16. | King_2013 traj 73 . PVGP 1 |

|----------------------------------------------------------------------------------------------------|

17. | Smahel_2008 126 . mult.games 1 |

18. | Wu_2013 153 . adapted YIAT "video games" 1 |

19. | Hull_2013 56 . GAS 1 |

20. | Bioulac_2008 10 . PVP 1 |

21. | Lemos_2016 90 . VAT, GAS 1 |

|----------------------------------------------------------------------------------------------------|

22. | Wang_2018 167 . IGD 9 scale 1 |

25. | Tejeiro_2012 132 . PVGP 1 |

28. | Griffiths&H 39 . criteria adapt. w/ "playing" 1 |

30. | Hussain_2009b_exc 58 . thematic analysis-Addiction 1 |

31. | Li_2011 92 . Adapted criteria from Choo 1 |

|----------------------------------------------------------------------------------------------------|

32. | Peng_2010 109 . Adapted from previous, included "gaming" 1 |

33. | Kiraly_2015 75 . POGquestion-SF 1 |

34. | Rehbein_2015 118 . CSAS 1 |

35. | Desai_2010 32 . 3 items sp to games 1 |

36. | Johansson_2004 61 . YIAT "playing" 1 |

|----------------------------------------------------------------------------------------------------|

38. | Sakuma_2016 191 . DSM5 + Griffiths 1 |

39. | Wartbg_2017 Assoc 194 . IGDS 1 |

40. | Demetrovics_2012 31 . POGquestion 1 |

41. | Scharkow_2014 121 . GAS 1 |

42. | Mentzoni_2011 100 . GAS 1 |

|----------------------------------------------------------------------------------------------------|

43. | Brunborg_2015 13 . GAS 1 |

44. | Grusser_2005 43 . CSVK 1 |

45. | Muller_2015 104 . AICAS gaming 1 |

46. | Collins_2012 28 . GAS 1 |

47. | Bonnaire_2017 171 . GAS 1 |

|----------------------------------------------------------------------------------------------------|

48. | Dreier_2016 34 . AICA-S 1 |

49. | Thalemann_2004 133 . CSVK comp games 1 |

50. | Coyne_2015 29 . Adapted DSM IV TR gaming 1 |

52. | Pontes_2014 111 . IGD-20 1 |

53. | Lemmens_2009 86 . DSM based criteria "playing" 1 |

|----------------------------------------------------------------------------------------------------|

54. | Kwon_2011 80 . IAS (adapted to games) 1 |

55. | Hart_2009 51 . PVP 1 |

56. | Panagiot_2017 163 . PVGT 1 |

57. | Seok_2012 124 . Adap Charlton's for online gaming 1 |

58. | Chiu_2004 22 . new scale 1 |

|----------------------------------------------------------------------------------------------------|

59. | Festl_2012 36 . GAS 1 |

60. | Kim_NR_2016 67 . DSM 5 criteria 1 |

62. | Henchoz_2016 53 . GAS 1 |

63. | Haagsma_2012 prev 45 . GAS and 3 items 1 |

64. | Kim_K_2015 65 . Int Game Add scale for adol 1 |

|----------------------------------------------------------------------------------------------------|

65. | Porter_2010 112 . PVG 1 |

69. | Rehbein_2013 117 . CSAS 1 |

70. | Salguero_2002 120 . PVP 1 |

71. | Wong_2016 195 . mod YDQ w/ ex 1 |

72. | Seah_2007 123 . Adap Charlton's for gaming 1 |

|----------------------------------------------------------------------------------------------------|

73. | Yee_2006a_dem 154 . single item 1 |

74. | Yee_2006b_psych 155 . 4 question and qualitative 1 |

75. | Hussain_2012 59 . GAS 1 |

77. | Colder_carr_2018 174 . AICA-S, IGD class 1 |

78. | Brunborg_2014 12 . GAS 1 |

|----------------------------------------------------------------------------------------------------|

79. | Chiou_2008 21 . OAST 1 |

80. | Batthya_2009 6 . CSVK-R (new) 1 |

81. | Vukosc-G_2015 144 . GAS 1 |

83. | Choo_2010 23 . Adapted DSM IV TR gaming 1 |

84. | Starcevic_2011 128 . VGU questionnaire 1 |

|----------------------------------------------------------------------------------------------------|

85. | Wan_2006a psych 146 . OAST 1 |

86. | Na_2017 162 . IGD crit survey 1 |

87. | Skoric_2009 125 . novel scale about VG Addiction 1 |

88. | Turner_2012 139 . PVP 1 |

89. | Coeffec_2015 27 . PUVG 1 |

|----------------------------------------------------------------------------------------------------|

90. | Mehroof_2010 99 . GAS 1 |

93. | King_2016 160 . IGD checklist 1 |

94. | Hsue_2009 55 . Chou scale 1 |

95. | Gentile_2009 37 . Adapted DSM IV TR 1 |

96. | Vadlin_5/2016 166 . GAIT (IGD-based) 1 |

|----------------------------------------------------------------------------------------------------|

97. | Gentile_2011 38 . Adapted DSM-IV-TR.latent classes 1 |

98. | Wan_Chiou_2007 148 . OAST 1 |

99. | Topor_2011 137 . novel scale based on DSMIVTR 1 |

100. | Schmitt_2015 122 . Revised Choo/Gentile scale 1 |

101. | Lemmens_2011_agg 87 . DSM based criteria "playing" 1 |

|----------------------------------------------------------------------------------------------------|

102. | Jimenez_2014 159 . VDT 1 |

103. | Yen_2016 165 . IGD crit interview 1 |

104. | Liu_2009 93 . new scale 1 |

105. | Lemmens_2015 89 . IGD scale 1 |

106. | Manniko_2015 96 . GAS 1 |

|----------------------------------------------------------------------------------------------------|

107. | Chou_2003 24 . New scale 1 |

108. | Park_2017 188 . dsm 5 crit 1 |

109. | Kim_2010 64 . POGU 1 |

110. | Lemmens_2006 85 . dsm iv adapted 1 |

111. | Kim_N_2016 184 . OGAS 1 |

|----------------------------------------------------------------------------------------------------|

112. | Jeong_2011 60 . YIAT "gaming" 1 |

113. | Kim_2008 63 . Online game Addiction scale 1 |

114. | Tolchinsky_2011 136 . PVGP 1 |

115. | King_2009a_motiv 68 . PVG 1 |

116. | Haagsma_2013 TPB 46 . GAS 1 |

|----------------------------------------------------------------------------------------------------|

117. | Lu_2008 95 . new scale 1 |

118. | Rehbein_2010 116 . KFN-CSAS-II 1 |

119. | Wartbg_2017_IGD 168 . IGDS 1 |

120. | Metcalf_2011 101 . Adap Charlton's for MMORPG 1 |

121. | Zhou_2009 157 . OAGI (novel) 1 |

|----------------------------------------------------------------------------------------------------|

122. | Allison_2006 2 . Cinical interview 1 |

123. | Laconi_2017 161 . IGDT-10 1 |

124. | King_2011 71 . PVGT (also adapted 1 |

125. | Vadlin_2015 140 . GAIT (IGD-based) 1 |

+----------------------------------------------------------------------------------------------------+

------------------------------------------------------------------------------------------------------------------------------------

-> PGscaleYN = .

+------------------------------------------------------------------------------------+

| study id iascale wasitPIU pgscale PGscal~N |

|------------------------------------------------------------------------------------|

1. | Thalemann_2007 134 . Unclear . |

2. | Chuang_2006 25 . None . |

3. | Hoeft_2008 54 . None . |

4. | Chumbley_2006 26 . None . |

5. | Griffiths_2010a_OG_add 41 . none-wrong article? . |

|------------------------------------------------------------------------------------|

6. | Grusser_2007 44 . unclear . |

7. | King_2010 70 . none . |

8. | Sun_2008 131 . none-wrong article? . |

+------------------------------------------------------------------------------------+

.

. *** Otherwise, there are no IAscales when PIU==0 or missing

. list pgscale iascale PGscaleYN wasitPIU if strpos(pgscale, "Unclear") | strpos(pgscale, "unclear") | strpos(pgscale, "None") | str

> pos(pgscale, "none")

+-----------------------------------------------------+

| pgscale iascale PGscal~N wasitPIU |

|-----------------------------------------------------|

189. | Unclear . . |

190. | None . . |

191. | None . . |

192. | None . . |

193. | none-wrong article? . . |

|-----------------------------------------------------|

194. | unclear . . |

195. | none . . |

196. | none-wrong article? . . |

+-----------------------------------------------------+

. bys PGscaleYN: list study id iascale wasitPIU pgscale PGscaleYN if wasitPIU==0 | wasitPIU==.

------------------------------------------------------------------------------------------------------------------------------------

-> PGscaleYN = 0

+----------------------------------------------------------------------------------------------------------+

| study id iascale wasitPIU pgscale PGscal~N |

|----------------------------------------------------------------------------------------------------------|

2. | Griffiths_2004 40 . "sacrificing other activities to play [EQ]" 0 |

6. | Ng_2005 105 . quest. that "suggested heavy overuse" 0 |

7. | Chappell_2006 17 . thematic 0 |

8. | Cultura_2002 30 . parent report 0 |

27. | Dworak_2007 35 . single excessive game use 0 |

|----------------------------------------------------------------------------------------------------------|

28. | Charlt_2002 18 0 Computer Comfort anxiety scale 0 |

39. | Peters_2008 110 0 WoW-specific, based on Charlton 0 |

42. | Charlt_2010 20 . game-specific Add–Eng questionnaire 0 |

43. | Bae_2016 170 . IGD+ADHD 0 |

47. | Wood_2007 152 . theme: experience of "time loss" 0 |

|----------------------------------------------------------------------------------------------------------|

49. | Rikkers_2016 119 . combined games/internet 0 |

51. | Lehenbau_2015 84 0 WoW-specific scale based on Charlton 0 |

52. | Pawlikowski_2011 108 0 IATWoW 0 |

53. | Baer _2012 4 0 Comp/Gaming station Add scale 0 |

56. | King_2009b_under 69 . 1 theme excessive playing w/ consequences 0 |

|----------------------------------------------------------------------------------------------------------|

58. | Charlt_2007 19 . game-specific Add–Eng questionnaire 0 |

60. | Baer_2011 5 0 Comp/Gaming station Add scale 0 |

61. | Griffiths_2010b_role 42 . self-rep Addiction v not 0 |

+----------------------------------------------------------------------------------------------------------+

------------------------------------------------------------------------------------------------------------------------------------

-> PGscaleYN = 1

+----------------------------------------------------------------------------------------------------+

| study id iascale wasitPIU pgscale PGscal~N |

|----------------------------------------------------------------------------------------------------|

1. | Walther_2012 145 . KFN-CSAS-II 1 |

3. | Yu_2016 197 . IGDS 1 |

4. | Wittek_2016 151 . GAS 1 |

5. | Pontes_2016 189 . IGDS9-SF 1 |

6. | Kim_NH_2016 66 . Online game Add scale 1 |

|----------------------------------------------------------------------------------------------------|

7. | Lemmens_2011_psyc 88 . DSM based criteria "playing" 1 |

8. | Lopez-F_2013 94 . PVP 1 |

9. | Son_2013 127 . scale from Wan w/ ex 1 |

10. | Yu_2015 156 . POGU 1 |

11. | Beranuy_2010 8 . Clinical interview 1 |

|----------------------------------------------------------------------------------------------------|

12. | Hussain_2009a_att 57 . adap. from the Exercise Add Inv (EAI) 1 |

13. | Lafreniere_2009 81 . PVP 1 |

14. | Ream_2011 115 . PVP 1 |

15. | Papay_2013 106 . POGquestion 1 |

16. | King_2013 traj 73 . PVGP 1 |

|----------------------------------------------------------------------------------------------------|

17. | Smahel_2008 126 . mult.games 1 |

18. | Wu_2013 153 . adapted YIAT "video games" 1 |

19. | Hull_2013 56 . GAS 1 |

20. | Bioulac_2008 10 . PVP 1 |

21. | Lemos_2016 90 . VAT, GAS 1 |

|----------------------------------------------------------------------------------------------------|

22. | Wang_2018 167 . IGD 9 scale 1 |

25. | Tejeiro_2012 132 . PVGP 1 |

28. | Griffiths&H 39 . criteria adapt. w/ "playing" 1 |

30. | Hussain_2009b_exc 58 . thematic analysis-Addiction 1 |

31. | Li_2011 92 . Adapted criteria from Choo 1 |

|----------------------------------------------------------------------------------------------------|

32. | Peng_2010 109 . Adapted from previous, included "gaming" 1 |

33. | Kiraly_2015 75 . POGquestion-SF 1 |

34. | Rehbein_2015 118 . CSAS 1 |

35. | Desai_2010 32 . 3 items sp to games 1 |

36. | Johansson_2004 61 . YIAT "playing" 1 |

|----------------------------------------------------------------------------------------------------|

38. | Sakuma_2016 191 . DSM5 + Griffiths 1 |

39. | Wartbg_2017 Assoc 194 . IGDS 1 |

40. | Demetrovics_2012 31 . POGquestion 1 |

41. | Scharkow_2014 121 . GAS 1 |

42. | Mentzoni_2011 100 . GAS 1 |

|----------------------------------------------------------------------------------------------------|

43. | Brunborg_2015 13 . GAS 1 |

44. | Grusser_2005 43 . CSVK 1 |

45. | Muller_2015 104 . AICAS gaming 1 |

46. | Collins_2012 28 . GAS 1 |

47. | Bonnaire_2017 171 . GAS 1 |

|----------------------------------------------------------------------------------------------------|

48. | Dreier_2016 34 . AICA-S 1 |

49. | Thalemann_2004 133 . CSVK comp games 1 |

50. | Coyne_2015 29 . Adapted DSM IV TR gaming 1 |

52. | Pontes_2014 111 . IGD-20 1 |

53. | Lemmens_2009 86 . DSM based criteria "playing" 1 |

|----------------------------------------------------------------------------------------------------|

54. | Kwon_2011 80 . IAS (adapted to games) 1 |

55. | Hart_2009 51 . PVP 1 |

56. | Panagiot_2017 163 . PVGT 1 |

57. | Seok_2012 124 . Adap Charlton's for online gaming 1 |

58. | Chiu_2004 22 . new scale 1 |

|----------------------------------------------------------------------------------------------------|

59. | Festl_2012 36 . GAS 1 |

60. | Kim_NR_2016 67 . DSM 5 criteria 1 |

62. | Henchoz_2016 53 . GAS 1 |

63. | Haagsma_2012 prev 45 . GAS and 3 items 1 |

64. | Kim_K_2015 65 . Int Game Add scale for adol 1 |

|----------------------------------------------------------------------------------------------------|

65. | Porter_2010 112 . PVG 1 |

69. | Rehbein_2013 117 . CSAS 1 |

70. | Salguero_2002 120 . PVP 1 |

71. | Wong_2016 195 . mod YDQ w/ ex 1 |

72. | Seah_2007 123 . Adap Charlton's for gaming 1 |

|----------------------------------------------------------------------------------------------------|

73. | Yee_2006a_dem 154 . single item 1 |

74. | Yee_2006b_psych 155 . 4 question and qualitative 1 |

75. | Hussain_2012 59 . GAS 1 |

77. | Colder_carr_2018 174 . AICA-S, IGD class 1 |

78. | Brunborg_2014 12 . GAS 1 |

|----------------------------------------------------------------------------------------------------|

79. | Chiou_2008 21 . OAST 1 |

80. | Batthya_2009 6 . CSVK-R (new) 1 |

81. | Vukosc-G_2015 144 . GAS 1 |

83. | Choo_2010 23 . Adapted DSM IV TR gaming 1 |

84. | Starcevic_2011 128 . VGU questionnaire 1 |

|----------------------------------------------------------------------------------------------------|

85. | Wan_2006a psych 146 . OAST 1 |

86. | Na_2017 162 . IGD crit survey 1 |

87. | Skoric_2009 125 . novel scale about VG Addiction 1 |

88. | Turner_2012 139 . PVP 1 |

89. | Coeffec_2015 27 . PUVG 1 |

|----------------------------------------------------------------------------------------------------|

90. | Mehroof_2010 99 . GAS 1 |

93. | King_2016 160 . IGD checklist 1 |

94. | Hsue_2009 55 . Chou scale 1 |

95. | Gentile_2009 37 . Adapted DSM IV TR 1 |

96. | Vadlin_5/2016 166 . GAIT (IGD-based) 1 |

|----------------------------------------------------------------------------------------------------|

97. | Gentile_2011 38 . Adapted DSM-IV-TR.latent classes 1 |

98. | Wan_Chiou_2007 148 . OAST 1 |

99. | Topor_2011 137 . novel scale based on DSMIVTR 1 |

100. | Schmitt_2015 122 . Revised Choo/Gentile scale 1 |

101. | Lemmens_2011_agg 87 . DSM based criteria "playing" 1 |

|----------------------------------------------------------------------------------------------------|

102. | Jimenez_2014 159 . VDT 1 |

103. | Yen_2016 165 . IGD crit interview 1 |

104. | Liu_2009 93 . new scale 1 |

105. | Lemmens_2015 89 . IGD scale 1 |

106. | Manniko_2015 96 . GAS 1 |

|----------------------------------------------------------------------------------------------------|

107. | Chou_2003 24 . New scale 1 |

108. | Park_2017 188 . dsm 5 crit 1 |

109. | Kim_2010 64 . POGU 1 |

110. | Lemmens_2006 85 . dsm iv adapted 1 |

111. | Kim_N_2016 184 . OGAS 1 |

|----------------------------------------------------------------------------------------------------|

112. | Jeong_2011 60 . YIAT "gaming" 1 |

113. | Kim_2008 63 . Online game Addiction scale 1 |

114. | Tolchinsky_2011 136 . PVGP 1 |

115. | King_2009a_motiv 68 . PVG 1 |

116. | Haagsma_2013 TPB 46 . GAS 1 |

|----------------------------------------------------------------------------------------------------|

117. | Lu_2008 95 . new scale 1 |

118. | Rehbein_2010 116 . KFN-CSAS-II 1 |

119. | Wartbg_2017_IGD 168 . IGDS 1 |

120. | Metcalf_2011 101 . Adap Charlton's for MMORPG 1 |

121. | Zhou_2009 157 . OAGI (novel) 1 |

|----------------------------------------------------------------------------------------------------|

122. | Allison_2006 2 . Cinical interview 1 |

123. | Laconi_2017 161 . IGDT-10 1 |

124. | King_2011 71 . PVGT (also adapted 1 |

125. | Vadlin_2015 140 . GAIT (IGD-based) 1 |

+----------------------------------------------------------------------------------------------------+

------------------------------------------------------------------------------------------------------------------------------------

-> PGscaleYN = .

+------------------------------------------------------------------------------------+

| study id iascale wasitPIU pgscale PGscal~N |

|------------------------------------------------------------------------------------|

1. | Thalemann_2007 134 . Unclear . |

2. | Chuang_2006 25 . None . |

3. | Hoeft_2008 54 . None . |

4. | Chumbley_2006 26 . None . |

5. | Griffiths_2010a_OG_add 41 . none-wrong article? . |

|------------------------------------------------------------------------------------|

6. | Grusser_2007 44 . unclear . |

7. | King_2010 70 . none . |

8. | Sun_2008 131 . none-wrong article? . |

+------------------------------------------------------------------------------------+

.

. ***By this point (and given the above), if there is no value for wasitPIU and no value for iascale,

. **** and there is a value for PGscaleYN, it is definitely not PIU. This is confirmed by

. ****looking at the list following.

. list study id iascale pgscale wasitPIU PGscaleYN if wasitPIU==. & iascale=="" & PGscaleYN==1

+----------------------------------------------------------------------------------------------------+

| study id iascale pgscale wasitPIU PGscal~N |

|----------------------------------------------------------------------------------------------------|

64. | Walther_2012 145 KFN-CSAS-II . 1 |

66. | Yu_2016 197 IGDS . 1 |

67. | Wittek_2016 151 GAS . 1 |

68. | Pontes_2016 189 IGDS9-SF . 1 |

69. | Kim_NH_2016 66 Online game Add scale . 1 |

|----------------------------------------------------------------------------------------------------|

70. | Lemmens_2011_psyc 88 DSM based criteria "playing" . 1 |

71. | Lopez-F_2013 94 PVP . 1 |

72. | Son_2013 127 scale from Wan w/ ex . 1 |

73. | Yu_2015 156 POGU . 1 |

74. | Beranuy_2010 8 Clinical interview . 1 |

|----------------------------------------------------------------------------------------------------|

75. | Hussain_2009a_att 57 adap. from the Exercise Add Inv (EAI) . 1 |

76. | Lafreniere_2009 81 PVP . 1 |

77. | Ream_2011 115 PVP . 1 |

78. | Papay_2013 106 POGquestion . 1 |

79. | King_2013 traj 73 PVGP . 1 |

|----------------------------------------------------------------------------------------------------|

80. | Smahel_2008 126 mult.games . 1 |

81. | Wu_2013 153 adapted YIAT "video games" . 1 |

82. | Hull_2013 56 GAS . 1 |

83. | Bioulac_2008 10 PVP . 1 |

84. | Lemos_2016 90 VAT, GAS . 1 |

|----------------------------------------------------------------------------------------------------|

85. | Wang_2018 167 IGD 9 scale . 1 |

88. | Tejeiro_2012 132 PVGP . 1 |

91. | Griffiths&H 39 criteria adapt. w/ "playing" . 1 |

93. | Hussain_2009b_exc 58 thematic analysis-Addiction . 1 |

94. | Li_2011 92 Adapted criteria from Choo . 1 |

|----------------------------------------------------------------------------------------------------|

95. | Peng_2010 109 Adapted from previous, included "gaming" . 1 |

96. | Kiraly_2015 75 POGquestion-SF . 1 |

97. | Rehbein_2015 118 CSAS . 1 |

98. | Desai_2010 32 3 items sp to games . 1 |

99. | Johansson_2004 61 YIAT "playing" . 1 |

|----------------------------------------------------------------------------------------------------|

101. | Sakuma_2016 191 DSM5 + Griffiths . 1 |

102. | Wartbg_2017 Assoc 194 IGDS . 1 |

103. | Demetrovics_2012 31 POGquestion . 1 |

104. | Scharkow_2014 121 GAS . 1 |

105. | Mentzoni_2011 100 GAS . 1 |

|----------------------------------------------------------------------------------------------------|

106. | Brunborg_2015 13 GAS . 1 |

107. | Grusser_2005 43 CSVK . 1 |

108. | Muller_2015 104 AICAS gaming . 1 |

109. | Collins_2012 28 GAS . 1 |

110. | Bonnaire_2017 171 GAS . 1 |

|----------------------------------------------------------------------------------------------------|

111. | Dreier_2016 34 AICA-S . 1 |

112. | Thalemann_2004 133 CSVK comp games . 1 |

113. | Coyne_2015 29 Adapted DSM IV TR gaming . 1 |

115. | Pontes_2014 111 IGD-20 . 1 |

116. | Lemmens_2009 86 DSM based criteria "playing" . 1 |

|----------------------------------------------------------------------------------------------------|

117. | Kwon_2011 80 IAS (adapted to games) . 1 |

118. | Hart_2009 51 PVP . 1 |

119. | Panagiot_2017 163 PVGT . 1 |

120. | Seok_2012 124 Adap Charlton's for online gaming . 1 |

121. | Chiu_2004 22 new scale . 1 |

|----------------------------------------------------------------------------------------------------|

122. | Festl_2012 36 GAS . 1 |

123. | Kim_NR_2016 67 DSM 5 criteria . 1 |

125. | Henchoz_2016 53 GAS . 1 |

126. | Haagsma_2012 prev 45 GAS and 3 items . 1 |

127. | Kim_K_2015 65 Int Game Add scale for adol . 1 |

|----------------------------------------------------------------------------------------------------|

128. | Porter_2010 112 PVG . 1 |

132. | Rehbein_2013 117 CSAS . 1 |

133. | Salguero_2002 120 PVP . 1 |

134. | Wong_2016 195 mod YDQ w/ ex . 1 |

135. | Seah_2007 123 Adap Charlton's for gaming . 1 |

|----------------------------------------------------------------------------------------------------|

136. | Yee_2006a_dem 154 single item . 1 |

137. | Yee_2006b_psych 155 4 question and qualitative . 1 |

138. | Hussain_2012 59 GAS . 1 |

140. | Colder_carr_2018 174 AICA-S, IGD class . 1 |

141. | Brunborg_2014 12 GAS . 1 |

|----------------------------------------------------------------------------------------------------|

142. | Chiou_2008 21 OAST . 1 |

143. | Batthya_2009 6 CSVK-R (new) . 1 |

144. | Vukosc-G_2015 144 GAS . 1 |

146. | Choo_2010 23 Adapted DSM IV TR gaming . 1 |

147. | Starcevic_2011 128 VGU questionnaire . 1 |

|----------------------------------------------------------------------------------------------------|

148. | Wan_2006a psych 146 OAST . 1 |

149. | Na_2017 162 IGD crit survey . 1 |

150. | Skoric_2009 125 novel scale about VG Addiction . 1 |

151. | Turner_2012 139 PVP . 1 |

152. | Coeffec_2015 27 PUVG . 1 |

|----------------------------------------------------------------------------------------------------|

153. | Mehroof_2010 99 GAS . 1 |

156. | King_2016 160 IGD checklist . 1 |

157. | Hsue_2009 55 Chou scale . 1 |

158. | Gentile_2009 37 Adapted DSM IV TR . 1 |

159. | Vadlin_5/2016 166 GAIT (IGD-based) . 1 |

|----------------------------------------------------------------------------------------------------|

160. | Gentile_2011 38 Adapted DSM-IV-TR.latent classes . 1 |

161. | Wan_Chiou_2007 148 OAST . 1 |

162. | Topor_2011 137 novel scale based on DSMIVTR . 1 |

163. | Schmitt_2015 122 Revised Choo/Gentile scale . 1 |

164. | Lemmens_2011_agg 87 DSM based criteria "playing" . 1 |

|----------------------------------------------------------------------------------------------------|

165. | Jimenez_2014 159 VDT . 1 |

166. | Yen_2016 165 IGD crit interview . 1 |

167. | Liu_2009 93 new scale . 1 |

168. | Lemmens_2015 89 IGD scale . 1 |

169. | Manniko_2015 96 GAS . 1 |

|----------------------------------------------------------------------------------------------------|

170. | Chou_2003 24 New scale . 1 |

171. | Park_2017 188 dsm 5 crit . 1 |

172. | Kim_2010 64 POGU . 1 |

173. | Lemmens_2006 85 dsm iv adapted . 1 |

174. | Kim_N_2016 184 OGAS . 1 |

|----------------------------------------------------------------------------------------------------|

175. | Jeong_2011 60 YIAT "gaming" . 1 |

176. | Kim_2008 63 Online game Addiction scale . 1 |

177. | Tolchinsky_2011 136 PVGP . 1 |

178. | King_2009a_motiv 68 PVG . 1 |

179. | Haagsma_2013 TPB 46 GAS . 1 |

|----------------------------------------------------------------------------------------------------|

180. | Lu_2008 95 new scale . 1 |

181. | Rehbein_2010 116 KFN-CSAS-II . 1 |

182. | Wartbg_2017_IGD 168 IGDS . 1 |

183. | Metcalf_2011 101 Adap Charlton's for MMORPG . 1 |

184. | Zhou_2009 157 OAGI (novel) . 1 |

|----------------------------------------------------------------------------------------------------|

185. | Allison_2006 2 Cinical interview . 1 |

186. | Laconi_2017 161 IGDT-10 . 1 |

187. | King_2011 71 PVGT (also adapted . 1 |

188. | Vadlin_2015 140 GAIT (IGD-based) . 1 |

+----------------------------------------------------------------------------------------------------+

. ****we replace the known "not PIU" values with 0

. replace wasitPIU=0 if wasitPIU==. & iascale=="" & PGscaleYN==1

(109 real changes made)

.

. codebook wasitPIU

------------------------------------------------------------------------------------------------------------------------------------

wasitPIU Did the study meas. PIU?

------------------------------------------------------------------------------------------------------------------------------------

type: numeric (float)

range: [0,1] units: 1

unique values: 2 missing .: 20/196

tabulation: Freq. Value

115 0

61 1

20 .

. list id study pgscale PGscaleYN if wasitPIU==.

+---------------------------------------------------------------------------------------+

| id study pgscale PGscal~N |

|---------------------------------------------------------------------------------------|

2. | 40 Griffiths_2004 "sacrificing other activities to play [EQ]" 0 |

6. | 105 Ng_2005 quest. that "suggested heavy overuse" 0 |

7. | 17 Chappell_2006 thematic 0 |

8. | 30 Cultura_2002 parent report 0 |

27. | 35 Dworak_2007 single excessive game use 0 |

|---------------------------------------------------------------------------------------|

42. | 20 Charlt_2010 game-specific Add–Eng questionnaire 0 |

43. | 170 Bae_2016 IGD+ADHD 0 |

47. | 152 Wood_2007 theme: experience of "time loss" 0 |

49. | 119 Rikkers_2016 combined games/internet 0 |

56. | 69 King_2009b_under 1 theme excessive playing w/ consequences 0 |

|---------------------------------------------------------------------------------------|

58. | 19 Charlt_2007 game-specific Add–Eng questionnaire 0 |

61. | 42 Griffiths_2010b_role self-rep Addiction v not 0 |

189. | 134 Thalemann_2007 Unclear . |

190. | 25 Chuang_2006 None . |

191. | 54 Hoeft_2008 None . |

|---------------------------------------------------------------------------------------|

192. | 26 Chumbley_2006 None . |

193. | 41 Griffiths_2010a_OG_add none-wrong article? . |

194. | 44 Grusser_2007 unclear . |

195. | 70 King_2010 none . |

196. | 131 Sun_2008 none-wrong article? . |

+---------------------------------------------------------------------------------------+

. ***the remaining missing have either unclear scales or specific scales/measures that are neither games nor internet alone

. replace wasitPIU=0 if wasitPIU==. & PGscaleYN==0

(12 real changes made)

. list id study pgscale PGscaleYN if wasitPIU==.

+---------------------------------------------------------------+

| id study pgscale PGscal~N |

|---------------------------------------------------------------|

189. | 134 Thalemann_2007 Unclear . |

190. | 25 Chuang_2006 None . |

191. | 54 Hoeft_2008 None . |

192. | 26 Chumbley_2006 None . |

193. | 41 Griffiths_2010a_OG_add none-wrong article? . |

|---------------------------------------------------------------|

194. | 44 Grusser_2007 unclear . |

195. | 70 King_2010 none . |

196. | 131 Sun_2008 none-wrong article? . |

+---------------------------------------------------------------+

.

. ***now only 8 cases where there is no value b/c scale is unclear

.

. ***Just a quick var to identify whether a study is neither PG nor PIU

. cap drop neither

. gen neither=.

(196 missing values generated)

. codebook neither

------------------------------------------------------------------------------------------------------------------------------------

neither (unlabeled)

------------------------------------------------------------------------------------------------------------------------------------

type: numeric (float)

range: [.,.] units: .

unique values: 0 missing .: 196/196

tabulation: Freq. Value

196 .

. replace neither=0 if wasitPIU==1 | PGscaleYN==1

(170 real changes made)

. replace neither=1 if wasitPIU==0 & PGscaleYN==0

(18 real changes made)

. lab var neither "Neither PIU nor PG"

. bys neither: tab PGscaleYN wasitPIU, mi

------------------------------------------------------------------------------------------------------------------------------------

-> neither = 0

| Did the study meas.

| PIU?

PGscaleYN | 0 1 | Total

-----------+----------------------+----------

0 | 0 45 | 45

1 | 109 16 | 125

-----------+----------------------+----------

Total | 109 61 | 170

------------------------------------------------------------------------------------------------------------------------------------

-> neither = 1

| Did the

| study

| meas. PIU?

PGscaleYN | 0 | Total

-----------+-----------+----------

0 | 18 | 18

-----------+-----------+----------

Total | 18 | 18

------------------------------------------------------------------------------------------------------------------------------------

-> neither = .

| Did the

| study

| meas. PIU?

PGscaleYN | . | Total

-----------+-----------+----------

. | 8 | 8

-----------+-----------+----------

Total | 8 | 8

. list study id pgscale if neither==1

+--------------------------------------------------------------------------+

| study id pgscale |

|--------------------------------------------------------------------------|

171. | Peters_2008 110 WoW-specific, based on Charlton |

172. | Charlt_2010 20 game-specific Add–Eng questionnaire |

173. | Griffiths_2004 40 "sacrificing other activities to play [EQ]" |

174. | Lehenbau_2015 84 WoW-specific scale based on Charlton |

175. | Chappell_2006 17 thematic |

|--------------------------------------------------------------------------|

176. | Ng_2005 105 quest. that "suggested heavy overuse" |

177. | Baer _2012 4 Comp/Gaming station Add scale |

178. | Griffiths_2010b_role 42 self-rep Addiction v not |

179. | Baer_2011 5 Comp/Gaming station Add scale |

180. | King_2009b_under 69 1 theme excessive playing w/ consequences |

|--------------------------------------------------------------------------|

181. | Rikkers_2016 119 combined games/internet |

182. | Bae_2016 170 IGD+ADHD |

183. | Dworak_2007 35 single excessive game use |

184. | Cultura_2002 30 parent report |

185. | Wood_2007 152 theme: experience of "time loss" |

|--------------------------------------------------------------------------|

186. | Pawlikowski_2011 108 IATWoW |

187. | Charlt_2007 19 game-specific Add–Eng questionnaire |

188. | Charlt_2002 18 Computer Comfort anxiety scale |

+--------------------------------------------------------------------------+

.

. save "/Users/michellecarras/Dropbox/Sys rev of sys rev Revisions/PLOS ONE submission/New submission July 2020/OSF additions/Databa

> se.dta", replace

file /Users/michellecarras/Dropbox/Sys rev of sys rev Revisions/PLOS ONE submission/New submission July 2020/OSF additions/Database.

> dta saved

.

. replace IAdepyesno=. if wasitPIU==0 | wasitPIU==.

(6 real changes made, 6 to missing)

. replace IAanxyesno=. if wasitPIU==0 | wasitPIU==.

(6 real changes made, 6 to missing)

.

. ****EDA & validation in codebook report

. codebook PGscaleYN, compact

Variable Obs Unique Mean Min Max Label

------------------------------------------------------------------------------------------------------------------------------------

PGscaleYN 188 2 .6648936 0 1 PGscaleYN

------------------------------------------------------------------------------------------------------------------------------------

. codebook PGscaleYN

------------------------------------------------------------------------------------------------------------------------------------

PGscaleYN PGscaleYN

------------------------------------------------------------------------------------------------------------------------------------

type: numeric (byte)

range: [0,1] units: 1

unique values: 2 missing .: 8/196

tabulation: Freq. Value

63 0

125 1

8 .

. tab PGscaleYN, mi

PGscaleYN | Freq. Percent Cum.

------------+-----------------------------------

0 | 63 32.14 32.14

1 | 125 63.78 95.92

. | 8 4.08 100.00

------------+-----------------------------------

Total | 196 100.00

. list study id PGscaleYN pgscale iascale if missing(PGscaleYN)

+-------------------------------------------------------------------------+

| study id PGscal~N pgscale iascale |

|-------------------------------------------------------------------------|

189. | Grusser_2007 44 . unclear |

190. | Chuang_2006 25 . None |

191. | Chumbley_2006 26 . None |

192. | Sun_2008 131 . none-wrong article? |

193. | Thalemann_2007 134 . Unclear |

|-------------------------------------------------------------------------|

194. | Griffiths_2010a_OG_add 41 . none-wrong article? |

195. | King_2010 70 . none |

196. | Hoeft_2008 54 . None |

+-------------------------------------------------------------------------+

. tab PGscaleYN wasitPIU, mi

| Did the study meas. PIU?

PGscaleYN | 0 1 . | Total

-----------+---------------------------------+----------

0 | 18 45 0 | 63

1 | 109 16 0 | 125

. | 0 0 8 | 8

-----------+---------------------------------+----------

Total | 127 61 8 | 196

.

. codebook wasitPIU, compact

Variable Obs Unique Mean Min Max Label

------------------------------------------------------------------------------------------------------------------------------------

wasitPIU 188 2 .3244681 0 1 Did the study meas. PIU?

------------------------------------------------------------------------------------------------------------------------------------

. codebook wasitPIU

------------------------------------------------------------------------------------------------------------------------------------

wasitPIU Did the study meas. PIU?

------------------------------------------------------------------------------------------------------------------------------------

type: numeric (float)

range: [0,1] units: 1

unique values: 2 missing .: 8/196

tabulation: Freq. Value

127 0

61 1

8 .

. tab wasitPIU, mi

Did the |

study meas. |

PIU? | Freq. Percent Cum.

------------+-----------------------------------

0 | 127 64.80 64.80

1 | 61 31.12 95.92

. | 8 4.08 100.00

------------+-----------------------------------

Total | 196 100.00

. tab wasitPIU PGanxyesno, mi

Did the |

study | New PGanx without 3 or 2

meas. PIU? | 0_No 1_Yes . | Total

-----------+---------------------------------+----------

0 | 104 23 0 | 127

1 | 49 12 0 | 61

. | 7 0 1 | 8

-----------+---------------------------------+----------

Total | 160 35 1 | 196

. tab wasitPIU IAanxyesno, mi

Did the |

study | The study meas.d IA & anx

meas. PIU? | 0 1 . | Total

-----------+---------------------------------+----------

0 | 0 0 127 | 127

1 | 17 12 32 | 61

. | 0 0 8 | 8

-----------+---------------------------------+----------

Total | 17 12 167 | 196

. tab wasitPIU PGdepyesno, mi

Did the |

study | New PGdep without 3 or2

meas. PIU? | 0_No 1_Yes . | Total

-----------+---------------------------------+----------

0 | 101 26 0 | 127

1 | 43 18 0 | 61

. | 7 0 1 | 8

-----------+---------------------------------+----------

Total | 151 44 1 | 196

. tab wasitPIU IAdepyesno, mi

Did the |

study | The study meas.d IA & dep

meas. PIU? | 0 1 . | Total

-----------+---------------------------------+----------

0 | 0 0 127 | 127

1 | 12 18 31 | 61

. | 0 0 8 | 8

-----------+---------------------------------+----------

Total | 12 18 166 | 196

.

. tab PGscaleYN

PGscaleYN | Freq. Percent Cum.

------------+-----------------------------------

0 | 63 33.51 33.51

1 | 125 66.49 100.00

------------+-----------------------------------

Total | 188 100.00

. codebook PGanxyesno, compact

Variable Obs Unique Mean Min Max Label

------------------------------------------------------------------------------------------------------------------------------------

PGanxyesno 195 2 .1794872 0 1 New PGanx without 3 or 2

------------------------------------------------------------------------------------------------------------------------------------

. codebook PGanxyesno

------------------------------------------------------------------------------------------------------------------------------------

PGanxyesno New PGanx without 3 or 2

------------------------------------------------------------------------------------------------------------------------------------

type: numeric (byte)

label: yesnoPG

range: [0,1] units: 1

unique values: 2 missing .: 1/196

tabulation: Freq. Numeric Label

160 0 0_No

35 1 1_Yes

1 .

. tab PGanxyesno, mi

New PGanx without |

3 or 2 | Freq. Percent Cum.

-------------------+-----------------------------------

0_No | 160 81.63 81.63

1_Yes | 35 17.86 99.49

. | 1 0.51 100.00

-------------------+-----------------------------------

Total | 196 100.00

. tab PGanxyesno PGscaleYN, mi

New PGanx without | PGscaleYN

3 or 2 | 0 1 . | Total

-------------------+---------------------------------+----------

0_No | 56 97 7 | 160

1_Yes | 7 28 0 | 35

. | 0 0 1 | 1

-------------------+---------------------------------+----------

Total | 63 125 8 | 196

. tab PGanxyesno wasitPIU, mi

New PGanx without | Did the study meas. PIU?

3 or 2 | 0 1 . | Total

-------------------+---------------------------------+----------

0_No | 104 49 7 | 160

1_Yes | 23 12 0 | 35

. | 0 0 1 | 1

-------------------+---------------------------------+----------

Total | 127 61 8 | 196

. bys PGanxyesno: tab PGscaleYN wasitPIU, mi

------------------------------------------------------------------------------------------------------------------------------------

-> PGanxyesno = 0_No

| Did the study meas. PIU?

PGscaleYN | 0 1 . | Total

-----------+---------------------------------+----------

0 | 18 38 0 | 56

1 | 86 11 0 | 97

. | 0 0 7 | 7

-----------+---------------------------------+----------

Total | 104 49 7 | 160

------------------------------------------------------------------------------------------------------------------------------------

-> PGanxyesno = 1_Yes

| Did the study meas.

| PIU?

PGscaleYN | 0 1 | Total

-----------+----------------------+----------

0 | 0 7 | 7

1 | 23 5 | 28

-----------+----------------------+----------

Total | 23 12 | 35

------------------------------------------------------------------------------------------------------------------------------------

-> PGanxyesno = .

| Did the

| study

| meas. PIU?

PGscaleYN | . | Total

-----------+-----------+----------

. | 1 | 1

-----------+-----------+----------

Total | 1 | 1

. bys IAanxyesno: tab PGscaleYN wasitPIU, mi

------------------------------------------------------------------------------------------------------------------------------------

-> IAanxyesno = 0

| Did the

| study

| meas. PIU?

PGscaleYN | 1 | Total

-----------+-----------+----------

0 | 7 | 7

1 | 10 | 10

-----------+-----------+----------

Total | 17 | 17

------------------------------------------------------------------------------------------------------------------------------------

-> IAanxyesno = 1

| Did the

| study

| meas. PIU?

PGscaleYN | 1 | Total

-----------+-----------+----------

0 | 8 | 8

1 | 4 | 4

-----------+-----------+----------

Total | 12 | 12

------------------------------------------------------------------------------------------------------------------------------------

-> IAanxyesno = .

| Did the study meas. PIU?

PGscaleYN | 0 1 . | Total

-----------+---------------------------------+----------

0 | 18 30 0 | 48

1 | 109 2 0 | 111

. | 0 0 8 | 8

-----------+---------------------------------+----------

Total | 127 32 8 | 167

.

. codebook PGdepyesno, compact

Variable Obs Unique Mean Min Max Label

------------------------------------------------------------------------------------------------------------------------------------

PGdepyesno 195 2 .225641 0 1 New PGdep without 3 or2

------------------------------------------------------------------------------------------------------------------------------------

. codebook PGdepyesno

------------------------------------------------------------------------------------------------------------------------------------

PGdepyesno New PGdep without 3 or2

------------------------------------------------------------------------------------------------------------------------------------

type: numeric (byte)

label: yesnoPG

range: [0,1] units: 1

unique values: 2 missing .: 1/196

tabulation: Freq. Numeric Label

151 0 0_No

44 1 1_Yes

1 .

. tab PGdepyesno, mi

New PGdep without |

3 or2 | Freq. Percent Cum.

-------------------+-----------------------------------

0_No | 151 77.04 77.04

1_Yes | 44 22.45 99.49

. | 1 0.51 100.00

-------------------+-----------------------------------

Total | 196 100.00

. tab PGdepyesno PGscaleYN, mi

New PGdep without | PGscaleYN

3 or2 | 0 1 . | Total

-------------------+---------------------------------+----------

0_No | 50 94 7 | 151

1_Yes | 13 31 0 | 44

. | 0 0 1 | 1

-------------------+---------------------------------+----------

Total | 63 125 8 | 196

. tab PGdepyesno wasitPIU, mi

New PGdep without | Did the study meas. PIU?

3 or2 | 0 1 . | Total

-------------------+---------------------------------+----------

0_No | 101 43 7 | 151

1_Yes | 26 18 0 | 44

. | 0 0 1 | 1

-------------------+---------------------------------+----------

Total | 127 61 8 | 196

. bys PGdepyesno: tab PGscaleYN wasitPIU, mi

------------------------------------------------------------------------------------------------------------------------------------

-> PGdepyesno = 0_No

| Did the study meas. PIU?

PGscaleYN | 0 1 . | Total

-----------+---------------------------------+----------

0 | 18 32 0 | 50

1 | 83 11 0 | 94

. | 0 0 7 | 7

-----------+---------------------------------+----------

Total | 101 43 7 | 151

------------------------------------------------------------------------------------------------------------------------------------

-> PGdepyesno = 1_Yes

| Did the study meas.

| PIU?

PGscaleYN | 0 1 | Total

-----------+----------------------+----------

0 | 0 13 | 13

1 | 26 5 | 31

-----------+----------------------+----------

Total | 26 18 | 44

------------------------------------------------------------------------------------------------------------------------------------

-> PGdepyesno = .

| Did the

| study

| meas. PIU?

PGscaleYN | . | Total

-----------+-----------+----------

. | 1 | 1

-----------+-----------+----------

Total | 1 | 1

. bys IAdepyesno: tab PGscaleYN wasitPIU, mi

------------------------------------------------------------------------------------------------------------------------------------

-> IAdepyesno = 0

| Did the

| study

| meas. PIU?

PGscaleYN | 1 | Total

-----------+-----------+----------

0 | 2 | 2

1 | 10 | 10

-----------+-----------+----------

Total | 12 | 12

------------------------------------------------------------------------------------------------------------------------------------

-> IAdepyesno = 1

| Did the

| study

| meas. PIU?

PGscaleYN | 1 | Total

-----------+-----------+----------

0 | 13 | 13

1 | 5 | 5

-----------+-----------+----------

Total | 18 | 18

------------------------------------------------------------------------------------------------------------------------------------

-> IAdepyesno = .

| Did the study meas. PIU?

PGscaleYN | 0 1 . | Total

-----------+---------------------------------+----------

0 | 18 30 0 | 48

1 | 109 1 0 | 110

. | 0 0 8 | 8

-----------+---------------------------------+----------

Total | 127 31 8 | 166

.

.

. codebook neither, compact

Variable Obs Unique Mean Min Max Label

------------------------------------------------------------------------------------------------------------------------------------

neither 188 2 .0957447 0 1 Neither PIU nor PG

------------------------------------------------------------------------------------------------------------------------------------

. codebook neither

------------------------------------------------------------------------------------------------------------------------------------

neither Neither PIU nor PG

------------------------------------------------------------------------------------------------------------------------------------

type: numeric (float)

range: [0,1] units: 1

unique values: 2 missing .: 8/196

tabulation: Freq. Value

170 0

18 1

8 .

. tab neither, mi

Neither PIU |

nor PG | Freq. Percent Cum.

------------+-----------------------------------

0 | 170 86.73 86.73

1 | 18 9.18 95.92

. | 8 4.08 100.00

------------+-----------------------------------

Total | 196 100.00

. bys neither: tab PGscaleYN wasitPIU, mi

------------------------------------------------------------------------------------------------------------------------------------

-> neither = 0

| Did the study meas.

| PIU?

PGscaleYN | 0 1 | Total

-----------+----------------------+----------

0 | 0 45 | 45

1 | 109 16 | 125

-----------+----------------------+----------

Total | 109 61 | 170

------------------------------------------------------------------------------------------------------------------------------------

-> neither = 1

| Did the

| study

| meas. PIU?

PGscaleYN | 0 | Total

-----------+-----------+----------

0 | 18 | 18

-----------+-----------+----------

Total | 18 | 18

------------------------------------------------------------------------------------------------------------------------------------

-> neither = .

| Did the

| study

| meas. PIU?

PGscaleYN | . | Total

-----------+-----------+----------

. | 8 | 8

-----------+-----------+----------

Total | 8 | 8

. ***19 have both PIU and PG? But only some compare groups

.

.

. *****More notes about nonspecific depression/anxiety measures added

.

. replace depanxnote="examples given of adaptation; total scale score used for mood so nonspecific" if id==80

depanxnote was str67 now str76

(1 real change made)

. replace depanxnote="combined psychosomatic health score" if id==151

(1 real change made)

. replace depanxnote="Global Severity Index (GSI) general distress (combined)" if id==75

(1 real change made)

. replace depanxnote="CBCL anxious/depression, internalizing probs not counted as anx or dep" if id==10

(1 real change made)

. replace depanxnote="DASS21 used, not individual Qs" if id==101

(1 real change made)

. replace depanxnote="perceived stress, coping only" if id==34

(1 real change made)

. replace depanxnote="aggression, self-control, narcissism" if id==63

(1 real change made)

. replace depanxnote="Vietnamese Self-Reporting Questionnaire full scale" if id==127

(1 real change made)

. replace depanxnote="compared IGD to comorbid IGD+depression" if id==167

(1 real change made)

. replace depanxnote="parent anxiety, nonspecific distress" if id==168

(1 real change made)

. replace depanxnote="parent anxiety, nonspecific distress" if id==190

(1 real change made)

. replace depanxnote="depression compared between IGD tx arms, vs controls" if id==185

(1 real change made)

.

. save "/Users/michellecarras/Dropbox/Sys rev of sys rev Revisions/PLOS ONE submission/New submission July 2020/OSF additions/Databa

> se.dta", replace

file /Users/michellecarras/Dropbox/Sys rev of sys rev Revisions/PLOS ONE submission/New submission July 2020/OSF additions/Database.

> dta saved

.

. *******************************************************************************

. *

> *

. * SECTION 3

> *

. * Enter outcome data abstracted from systematic reviews *

. *

> *

. *

> *

. *******************************************************************************

.

. ***USE DATA ABSTRACTED FROM REVIEWS TO SHOW HOW REVIEWS REPORTED STUDY FINDINGS*

. ****i.e., did they report pos, neg and null results? *

. ***no entry means they did not report a result *

. ***sometimes they report results in a way that doesn't match outcomes

.

. ***Sugaya (added 5_2020)

. cap drop sug_dep1

. gen sug_dep1=.

(196 missing values generated)

. cap drop sug_dep2

. gen sug_dep2=.

(196 missing values generated)

. cap drop sug_dep3

. gen sug_dep3=.

(196 missing values generated)

.

. cap drop sug_anx1

. gen sug_anx1=.

(196 missing values generated)

. cap drop sug_anx2

. gen sug_anx2=.

(196 missing values generated)

. cap drop sug_anx3

. gen sug_anx3=.

(196 missing values generated)

.

. cap drop sug_Ldep1

. gen sug_Ldep1=.

(196 missing values generated)

. cap drop sug_Ldep2

. gen sug_Ldep2=.

(196 missing values generated)

.

. cap drop sug_Lanx1

. gen sug_Lanx1=.

(196 missing values generated)

. cap drop sug_Lanx2

. gen sug_Lanx2=.

(196 missing values generated)

.

. **Compare how dep/anx were actually reported in studies vs. what reviews said

. ***Sugaya only reported a few MH associations

. *Sugaya associations with dep

. *Strittmatter p 4 text

. replace sug_dep1=1 if id==130

(1 real change made)

.

. *King cognitive

. replace sug_dep1=1 if id==160

(1 real change made)

.

. **Sugaya assoc with anx

. replace sug_anx1=1 if id==160

(1 real change made)

.

.

. ***Gonzalez

. cap drop gonz_dep1

. gen gonz_dep1=.

(196 missing values generated)

. cap drop gonz_dep2

. gen gonz_dep2=.

(196 missing values generated)

. cap drop gonz_dep3

. gen gonz_dep3=.

(196 missing values generated)

. cap drop gonz_dep4

. gen gonz_dep4=.

(196 missing values generated)

.

.

. cap drop gonz_anx1

. gen gonz_anx1=.

(196 missing values generated)

. cap drop gonz_anx2

. gen gonz_anx2=.

(196 missing values generated)

. cap drop gonz_anx3

. gen gonz_anx3=.

(196 missing values generated)

. cap drop gonz_anx4

. gen gonz_anx4=.

(196 missing values generated)

.

. cap drop gonz_Ldep1

. gen gonz_Ldep1=.

(196 missing values generated)

. cap drop gonz_Ldep2

. gen gonz_Ldep2=.

(196 missing values generated)

. cap drop gonz_Lanx1

. gen gonz_Lanx1=.

(196 missing values generated)

. cap drop gonz_Lanx2

. gen gonz_Lanx2=.

(196 missing values generated)

.

. *Gonzalez associations with dep

. ***Jimenez-Murcia #159

. replace gonz_dep1=1 if id==159

(1 real change made)

.

. ***Kim NR #67

. replace gonz_dep1=1 if id==67

(1 real change made)

.

. ***King 2013 axis #72

. replace gonz_dep1=0 if id==72

(1 real change made)

.

. ***King 2016 #160

. replace gonz_dep1=1 if id==160

(1 real change made)

.

. ***Lanconi #161

. replace gonz_dep1=1 if id==161

(1 real change made)

.

. ***Mannikko #96

. replace gonz_dep1=1 if id==96

(1 real change made)

.

. ***Mentzoni #100

. replace gonz_dep1=1 if id==100

(1 real change made)

.

. ***Muller #104 - counting only withdrawn dep as dep

. replace gonz_dep1=1 if id==104

(1 real change made)

.

. ***Na #162

. replace gonz_dep1=1 if id==162

(1 real change made)

.

. ***Starcevic #128

. ******NOTE that Gonzalez defines correlations as partial if they are not found in all subsamples of the analysis.

. *****To be conservative, we will translate that into one positive and one null report

. replace gonz_dep1=1 if id==128

(1 real change made)

. replace gonz_dep2=0 if id==128

(1 real change made)

.

. ***Stetina #129

. replace gonz_dep1=1 if id==129

(1 real change made)

.

. ***Strittmatter #130

. replace gonz_dep1=1 if id==130

(1 real change made)

.

. ***Vadlin #166

. replace gonz_dep1=1 if id==166

(1 real change made)

.

. ***Wang #167

. replace gonz_dep1=1 if id==167

(1 real change made)

. ***Wartberg #168

. replace gonz_dep1=1 if id==168

(1 real change made)

. ***Wei #169

. replace gonz_dep1=1 if id==169

(1 real change made)

.

. ***Gentile #38

. replace gonz_Ldep1=1 if id==38

(1 real change made)

.

. ***Hyun #164

. replace gonz_dep1=1 if id==164

(1 real change made)

.

. ***Brunborg #12

. ***T1: full

. replace gonz_dep1=1 if id==12

(1 real change made)

. ***Other time: None

. replace gonz_dep2=0 if id==12

(1 real change made)

.

.

. ***Van Rooij#142

.

. replace gonz_Ldep1=0 if id==142

(1 real change made)

. replace gonz_Ldep2=1 if id==142

(1 real change made)

.

. *Gonzalez associations with anx

.

. **Cole&Hooley #158

. *anxiety trait, anxiety state, and social phobia

. replace gonz_anx1=1 if id==158

(1 real change made)

. replace gonz_anx2=1 if id==158

(1 real change made)

. replace gonz_anx3=1 if id==158

(1 real change made)

.

. ***Jimenez-Murcia #159

. ***anxiety, phobic anxiety

. replace gonz_anx1=1 if id==159

(1 real change made)

. replace gonz_anx2=1 if id==159

(1 real change made)

.

. ***Kim NR #67

. ***anxiety, phobic anxiety

. replace gonz_anx1=1 if id==67

(1 real change made)

. replace gonz_anx2=1 if id==67

(1 real change made)

.

. ***King 2013 axis #72

. replace gonz_anx1=0 if id==72

(1 real change made)

.

. ***King 2016 #160

. replace gonz_anx1=1 if id==160

(1 real change made)

.

. ***Mannikko #96

. replace gonz_anx1=1 if id==96

(1 real change made)

.

. ***Mentzoni #100

. replace gonz_anx1=1 if id==100

(1 real change made)

.

. ***Na #162

. replace gonz_anx1=1 if id==162

(1 real change made)

.

. ***Starcevic #128

. ******NOTE that Gonzalez defines correlations as partial if they are not found in all subsamples of the analysis.

. *****To be conservative, we will translate that into one positive and one null report

. ********Anxiety reporting

. replace gonz_anx1=1 if id==128

(1 real change made)

. replace gonz_anx2=0 if id==128

(1 real change made)

. ********Phobic anxiety

. replace gonz_anx3=1 if id==128

(1 real change made)

. replace gonz_anx4=0 if id==128

(1 real change made)

.

.

. ***Vadlin #166

. replace gonz_anx1=1 if id==166

(1 real change made)

.

. ***Wang #167--says anx is n/a, anx scale in study but didn't compare IGD/no IGD

.

. ***Wartberg #168

. replace gonz_anx1=1 if id==168

(1 real change made)

.

. ***Wei #169

. replace gonz_anx1=1 if id==169

(1 real change made)

.

. ***Gentile #38

. replace gonz_Lanx1=1 if id==38

(1 real change made)

. replace gonz_Lanx2=1 if id==38

(1 real change made)

.

.

. ***Van Rooij#142

. ***T1=none

. replace gonz_Lanx1=0 if id==142

(1 real change made)

. ***T2=none

. replace gonz_Lanx2=0 if id==142

(1 real change made)

.

.

. ***Hyun #164

. replace gonz_anx1=1 if id==164

(1 real change made)

.

.

. ***Mihara

. cap drop miha_dep1

. gen miha_dep1=.

(196 missing values generated)

. cap drop miha_dep2

. gen miha_dep2=.

(196 missing values generated)

. cap drop miha_dep3

. gen miha_dep3=.

(196 missing values generated)

. cap drop miha_dep4

. gen miha_dep4=.

(196 missing values generated)

.

.

. cap drop miha_anx1

. gen miha_anx1=.

(196 missing values generated)

. cap drop miha_anx2

. gen miha_anx2=.

(196 missing values generated)

. cap drop miha_anx3

. gen miha_anx3=.

(196 missing values generated)

. cap drop miha_anx4

. gen miha_anx4=.

(196 missing values generated)

.

. cap drop miha_Ldep1

. gen miha_Ldep1=.

(196 missing values generated)

. cap drop miha_Ldep2

. gen miha_Ldep2=.

(196 missing values generated)

. cap drop miha_Lanx1

. gen miha_Lanx1=.

(196 missing values generated)

. cap drop miha_Lanx2

. gen miha_Lanx2=.

(196 missing values generated)

.

. *Which studies from Mihara used a PG scale to meas. associations b/w PG and dep?

. list study id pgscale PGdepyesno inmihar if inmihar

+----------------------------------------------------------------------------------+

| study id pgscale PGdepy~o inmihar |

|----------------------------------------------------------------------------------|

1. | Manniko_2015 96 GAS 1_Yes 1 |

8. | Johansson_2004 61 YIAT "playing" 0_No 1 |

12. | Mentzoni_2011 100 GAS 1_Yes 1 |

23. | Rehbein_2010 116 KFN-CSAS-II 1_Yes 1 |

27. | Lemmens_2011_agg 87 DSM based criteria "playing" 0_No 1 |

|----------------------------------------------------------------------------------|

29. | Pontes_2014 111 IGD-20 0_No 1 |

32. | Wang_2014 149 GAS 0_No 1 |

33. | Konkoly_2015 78 gaming or "int chat" as BAD 0_No 1 |

38. | Rehbein_2015 118 CSAS 0_No 1 |

39. | Kim_NR_2016 67 DSM 5 criteria 1_Yes 1 |

|----------------------------------------------------------------------------------|

47. | Wittek_2016 151 GAS 0_No 1 |

48. | Hussain_2012 59 GAS 0_No 1 |

57. | Kiraly_2014 74 POGquestion 1_Yes 1 |

58. | Desai_2010 32 3 items sp to games 1_Yes 1 |

59. | Choo_2010 23 Adapted DSM IV TR gaming 0_No 1 |

|----------------------------------------------------------------------------------|

60. | VRooij_2011 142 CIUS 1_Yes 1 |

71. | Lemmens_2015 89 IGD scale 0_No 1 |

73. | Lemmens_2009 86 DSM based criteria "playing" 0_No 1 |

77. | Lopez-F_2013 94 PVP 0_No 1 |

78. | Rasmussen_2015 113 3 Q abt games, 3 Q abt Internet 0_No 1 |

|----------------------------------------------------------------------------------|

80. | Seok_2012 124 Adap Charlton's for online gaming 0_No 1 |

89. | King_2013 traj 73 PVGP 0_No 1 |

90. | Haagsma_2012 prev 45 GAS and 3 items 0_No 1 |

95. | Festl_2012 36 GAS 0_No 1 |

97. | Turner_2012 139 PVP 0_No 1 |

|----------------------------------------------------------------------------------|

104. | Achab_2011 1 DAS 0_No 1 |

108. | King_2013_Axis 72 PTU, specific to games 1_Yes 1 |

111. | Coeffec_2015 27 PUVG 0_No 1 |

112. | Griffiths&H 39 criteria adapt. w/ "playing" 0_No 1 |

118. | Thomas_2010 135 adapYDQ Int, YDQ comp games 0_No 1 |

|----------------------------------------------------------------------------------|

119. | Lemmens_2011_psyc 88 DSM based criteria "playing" 0_No 1 |

120. | Muller_2015 104 AICAS gaming 1_Yes 1 |

126. | Vadlin_2015 140 GAIT (IGD-based) 0_No 1 |

130. | Gentile_2009 37 Adapted DSM IV TR 0_No 1 |

137. | Dreier_2016 34 AICA-S 0_No 1 |

|----------------------------------------------------------------------------------|

140. | Papay_2013 106 POGquestion 1_Yes 1 |

147. | Yu_2015 156 POGU 0_No 1 |

148. | Henchoz_2016 53 GAS 0_No 1 |

151. | Gentile_2011 38 Adapted DSM-IV-TR.latent classes 1_Yes 1 |

154. | Strittmat_2015 130 YDQ 0_No 1 |

|----------------------------------------------------------------------------------|

158. | Brunborg_2014 12 GAS 1_Yes 1 |

160. | Brunborg_2015 13 GAS 0_No 1 |

163. | Schmitt_2015 122 Revised Choo/Gentile scale 0_No 1 |

166. | Scharkow_2014 121 GAS 0_No 1 |

168. | Rehbein_2013 117 CSAS 0_No 1 |

|----------------------------------------------------------------------------------|

169. | Haagsma_2013 TPB 46 GAS 0_No 1 |

193. | Grusser_2007 44 unclear 0_No 1 |

+----------------------------------------------------------------------------------+

.

.

. *Mihara associations with dep

.

. *Desai=#61

. replace miha_dep1=1 if id==32

(1 real change made)

. *Gentile=60

. replace miha_Ldep1=1 if id==38

(1 real change made)

. *Kim NR=70

. replace miha_dep1=1 if id==67

(1 real change made)

. *King 2013 anxis=72

. replace miha_dep1=1 if id==72

(1 real change made)

. *Kiraly 2014=45

. replace miha_dep1=1 if id==74

(1 real change made)

. *Manniko=51

. replace miha_dep1=1 if id==96

(1 real change made)

. *Mentzoni=40

. replace miha_dep1=1 if id==100

(1 real change made)

.

. *Strittmatter=56

. replace miha_dep1=1 if id==130

(1 real change made)

. replace miha_dep2=1 if id==130

(1 real change made)

.

. *Muller=52

. replace miha_dep1=1 if id==104

(1 real change made)

.

. **Other ways of reporting dep/anx outcomes in studies-do not affect results of this study

. **Achab=39="psych health" but also "more sad" (not depression), "more anxious" (yes anxiety)

. **Dreier=59="emo probs"

. **Wittek=58="psychometric health"

.

. list study id pgscale PGdepyesno miha_dep1 miha_dep2 PGscaleYN longit inmihar if inmihar==1 & PGdepyesno>0

+------------------------------------------------------------------------------------------------------------------------+

| study id pgscale PGdepy~o miha_d~1 miha_d~2 PGscal~N longit inmihar |

|------------------------------------------------------------------------------------------------------------------------|

1. | Manniko_2015 96 GAS 1_Yes 1 . 1 0 1 |

12. | Mentzoni_2011 100 GAS 1_Yes 1 . 1 0 1 |

23. | Rehbein_2010 116 KFN-CSAS-II 1_Yes . . 1 0 1 |

39. | Kim_NR_2016 67 DSM 5 criteria 1_Yes 1 . 1 0 1 |

57. | Kiraly_2014 74 POGquestion 1_Yes 1 . 1 0 1 |

|------------------------------------------------------------------------------------------------------------------------|

58. | Desai_2010 32 3 items sp to games 1_Yes 1 . 1 0 1 |

60. | VRooij_2011 142 CIUS 1_Yes . . 0 1 1 |

108. | King_2013_Axis 72 PTU, specific to games 1_Yes 1 . 1 0 1 |

120. | Muller_2015 104 AICAS gaming 1_Yes 1 . 1 0 1 |

140. | Papay_2013 106 POGquestion 1_Yes . . 1 0 1 |

|------------------------------------------------------------------------------------------------------------------------|

151. | Gentile_2011 38 Adapted DSM-IV-TR.latent classes 1_Yes . . 1 1 1 |

158. | Brunborg_2014 12 GAS 1_Yes . . 1 1 1 |

+------------------------------------------------------------------------------------------------------------------------+

.

. list study id pgscale PGanxyesno inmihar if inmihar==1 & PGanxyesno>0

+------------------------------------------------------------------------------+

| study id pgscale PGanxy~o inmihar |

|------------------------------------------------------------------------------|

1. | Manniko_2015 96 GAS 1_Yes 1 |

12. | Mentzoni_2011 100 GAS 1_Yes 1 |

23. | Rehbein_2010 116 KFN-CSAS-II 1_Yes 1 |

39. | Kim_NR_2016 67 DSM 5 criteria 1_Yes 1 |

60. | VRooij_2011 142 CIUS 1_Yes 1 |

|------------------------------------------------------------------------------|

104. | Achab_2011 1 DAS 1_Yes 1 |

108. | King_2013_Axis 72 PTU, specific to games 1_Yes 1 |

151. | Gentile_2011 38 Adapted DSM-IV-TR.latent classes 1_Yes 1 |

+------------------------------------------------------------------------------+

.

.

. *Dreier=59-nonspecific anx/dep and didn't report anx results

. *Gentile=60

. replace miha_Lanx1=1 if id==38

(1 real change made)

. replace miha_Lanx2=1 if id==38

(1 real change made)

. replace miha_Ldep1=1 if id==38

(0 real changes made)

. *King 2013 anxis=72

. replace miha_anx1=1 if id==72

(1 real change made)

. replace miha_anx2=1 if id==72

(1 real change made)

.

. *Manniko=51

. replace miha_anx1=1 if id==96

(1 real change made)

. *Mentzoni=40

. replace miha_anx1=1 if id==100

(1 real change made)

.

. *Rehbein=35

. replace miha_anx1=1 if id==116

(1 real change made)

.

. *Brunborg 2014

. replace miha_Ldep1=1 if id==12

(1 real change made)

.

.

.

. *Strittmatter

. *Muller=52 *we will say "withdrawn/depressed' is depression but not "anx/depr"

. **Achab=39="psych health"

. **Dreier=59="emo probs"

. **Wittek=58="psychometric health"

.

.

. **King

. cap drop king_dep1

. gen king_dep1=.

(196 missing values generated)

. cap drop king_dep2

. gen king_dep2=.

(196 missing values generated)

.

.

. cap drop king_anx1

. gen king_anx1=.

(196 missing values generated)

. cap drop king_anx2

. gen king_anx2=.

(196 missing values generated)

. cap drop king_anx3

.

.

. cap drop king_Ldep1

. gen king_Ldep1=.

(196 missing values generated)

. cap drop king_Ldep2

. gen king_Ldep2=.

(196 missing values generated)

. cap drop king_Lanx1

. gen king_Lanx1=.

(196 missing values generated)

. cap drop king_Lanx2

. gen king_Lanx2=.

(196 missing values generated)

.

. **King stated associations with dep (up arrow in table)

. *Gentile 2011

. replace king_dep1=1 if id==38

(1 real change made)

.

. *Li 2011

. replace king_dep1=1 if id==92

(1 real change made)

.

. *Porter 2010

. replace king_dep1=1 if id==112

(1 real change made)

. ***this actually seems to be completely a mistake; no mention of dep in Porter

. replace depanxnote= "King says assoc w/ dep but this is a mistake-wrong study?" if id==112

(1 real change made)

. *Metcalfe 2011

. replace king_dep1=1 if id==101

(1 real change made)

.

. *van Rooij 2010 identification

. replace king_dep1=1 if id==142

(1 real change made)

.

. *Meerkerk 2010

. replace king_dep1=1 if id==98

(1 real change made)

.

. * van Rooij 2010 role

. replace king_dep1=1 if id==141

(1 real change made)

.

. *Mentzoni 2011

. replace king_dep1=1 if id==100

(1 real change made)

.

. *Stetina 2011

. replace king_dep1=1 if id==129

(1 real change made)

.

. *van Rooij 2012 VAT

. replace king_dep1=1 if id==143

(1 real change made)

.

. *Bayraktar 2007

. replace king_dep1=1 if id==7

(1 real change made)

.

. *Kwon 2009

. replace king_dep1=1 if id==80

(1 real change made)

.

. *Rehbein 2010

. replace king_dep1=1 if id==116

(1 real change made)

.

. **King stated associations with anx

. **Gentile 2011

. replace king_anx1=1 if id==38

(1 real change made)

. replace king_anx2=1 if id==38

(1 real change made)

.

. **Mehroof 2010

. replace king_anx1=1 if id==99

(1 real change made)

.

. *Mentzoni 2011

. replace king_anx1=1 if id==100

(1 real change made)

.

. *Kim & Kim 2010

. replace king_anx1=1 if id==64

(1 real change made)

.

. *van Rooij 2012

. replace king_anx1=1 if id==143

(1 real change made)

.

. **Manniko

.

. cap drop man_dep1

. gen man_dep1=.

(196 missing values generated)

. cap drop man_dep2

. gen man_dep2=.

(196 missing values generated)

.

. cap drop man_anx1

. gen man_anx1=.

(196 missing values generated)

. cap drop man_anx2

. gen man_anx2=.

(196 missing values generated)

.

. cap drop man_Ldep1

. gen man_Ldep1=.

(196 missing values generated)

. cap drop man_Ldep2

. gen man_Ldep2=.

(196 missing values generated)

. cap drop man_Lanx1

. gen man_Lanx1=.

(196 missing values generated)

. cap drop man_Lanx2

. gen man_Lanx2=.

(196 missing values generated)

.

.

. **Mannikko stated associations with dep

.

. replace man_dep1=1 if ///

> id==3 | ///

> id==11 | ///

> id==12 | ///

> id==32 | ///

> id==67 | ///

> id==74 | ///

> id==84 | ///

> id==90 | ///

> id==92 | ///

> id==100 | ///

> id==104 | ///

> id==106 | ///

> id==109 | ///

> id==127 | ///

> id==128 | ///

> id==130 | ///

> id==143 | ///

> id==144 | ///

> id==116

(19 real changes made)

.

.

. replace man_dep2=1 if id==130

(1 real change made)

.

. **Mannikko stated associations with anx

. replace man_anx1=1 if ///

> id==3 | ///

> id==11 | ///

> id==66 | ///

> id==67 | ///

> id==83 | ///

> id==84 | ///

> id==90 | ///

> id==99 | ///

> id==100 | ///

> id==104 | ///

> id==127 | ///

> id==128 | ///

> id==143 | ///

> id==144 | ///

> id==145

(15 real changes made)

.

. replace man_anx2=1 if id==144 | id==128

(2 real changes made)

.

. **one "longit" dep result for VR 2011--reported as longit but not

. replace man_Ldep1=1 if id==142

(1 real change made)

.

. **Kuss Online

.

. cap drop kuon_dep1

. gen kuon_dep1=.

(196 missing values generated)

. cap drop kuon_dep2

. gen kuon_dep2=.

(196 missing values generated)

.

. cap drop kuon_anx1

. gen kuon_anx1=.

(196 missing values generated)

. cap drop kuon_anx2

. gen kuon_anx2=.

(196 missing values generated)

.

. cap drop kuon_Ldep1

. gen kuon_Ldep1=.

(196 missing values generated)

. cap drop kuon_Ldep2

. gen kuon_Ldep2=.

(196 missing values generated)

. cap drop kuon_Lanx1

. gen kuon_Lanx1=.

(196 missing values generated)

. cap drop kuon_Lanx2

. gen kuon_Lanx2=.

(196 missing values generated)

.

.

. replace kuon_dep1=1 if ///

> id==116

(1 real change made)

.

.

. replace kuon_anx1=1 if ///

> id==6 | ///

> id==64

(2 real changes made)

.

.

. replace kuon_Lanx1=1 if ///

> id==38 | id==142

(2 real changes made)

.

.

. replace kuon_Ldep1=1 if ///

> id==38 | ///

> id==92 | ///

> id==142

(3 real changes made)

.

.

.

. **Kuss Internet

. cap drop kuint_dep1

. gen kuint_dep1=.

(196 missing values generated)

. cap drop kuint_dep2

. gen kuint_dep2=.

(196 missing values generated)

.

. cap drop kuint_anx1

. gen kuint_anx1=.

(196 missing values generated)

. cap drop kuint_anx2

. gen kuint_anx2=.

(196 missing values generated)

. cap drop kuint_anx3

. gen kuint_anx3=.

(196 missing values generated)

.

. cap drop kuint_Ldep1

. gen kuint_Ldep1=.

(196 missing values generated)

. cap drop kuint_Ldep2

. gen kuint_Ldep2=.

(196 missing values generated)

. cap drop kuint_Lanx1

. gen kuint_Lanx1=.

(196 missing values generated)

. cap drop kuint_Lanx2

. gen kuint_Lanx2=.

(196 missing values generated)

.

. replace kuint_dep1=1 if ///

> id==2

(1 real change made)

.

. replace kuint_anx1=1 if ///

> id==2 | ///

> id==6 | ///

> id==99

(3 real changes made)

.

. replace kuint_anx2=1 if id==2

(1 real change made)

.

. replace kuint_anx3=1 if id==2

(1 real change made)

.

.

. foreach var in gonz_Lanx1 gonz_Lanx2 king_Lanx1 man_Lanx1 king_Lanx2 man_Lanx2 kuint_Lanx1 miha_Lanx1 kuint_Lanx2 miha_Lanx2 k

> uon_Lanx1 kuon_Lanx2 {

2. lab var `var' "Longit anx result in review"

3. }

. ds anxbi*vote

anxbi1vote anxbi2vote anxbi3vote anxbi4vote anxbi5vote anxbi6vote anxbi7vote anxbi8vote anxbi9vote

. foreach var in anxbi1vote anxbi3vote anxbi5vote anxbi7vote anxbi9vote anxbi2vote anxbi4vote anxbi6vote anxbi8vote {

2. lab var `var' "C-S bivar anx result in study"

3. }

. /*ds IAdepbi*

> foreach var in IAdepbi1vote IAdepbi3vote IAdepbi1txt IAdepbi3txt IAdepbi2vote IAdepbi4vote IAdepbi2txt IAdepb

> i4txt {

> lab var `var' "C-S bivar dep result in study if IA scale"

> }

> */

. ds anxmu*vote

anxmu1vote anxmu2vote anxmu3vote anxmu4vote anxmu5vote anxmu6vote anxmu7vote anxmu8vote anxmu9vote

. foreach var in anxmu1vote anxmu2vote anxmu3vote anxmu4vote anxmu5vote anxmu6vote ///

> anxmu7vote anxmu8vote anxmu9vote {

2. lab var `var' "C-S multiv anx result in study"

3. }

. ds depbi*vote

depbi1vote depbi2vote depbi3vote depbi4vote depbi5vote depbi6vote depbi7vote depbi8vote depbi9vote

. foreach var in depbi1vote depbi3vote depbi5vote depbi7vote depbi9vote depbi2vote depbi4vote depbi6vote depbi8vote {

2. lab var `var' "C-S biv dep result in study"

3. }

. ds kuint*dep*

kuint_dep1 kuint_dep2 kuint_Ldep1 kuint_Ldep2

. ds kuon*dep*

kuon_dep1 kuon_dep2 kuon_Ldep1 kuon_Ldep2

. ds king*dep*

king_dep1 king_dep2 king_Ldep1 king_Ldep2

. ds man*dep*

man_dep1 man_dep2 man_Ldep1 man_Ldep2

. ds miha*dep*

miha_dep1 miha_dep2 miha_dep3 miha_dep4 miha_Ldep1 miha_Ldep2

. ds sug*dep*

sug_dep1 sug_dep2 sug_dep3 sug_Ldep1 sug_Ldep2

.

. foreach var in kuint_Ldep1 kuint_Ldep2 kuon_Ldep1 kuon_Ldep2 king_Ldep1 king_Ldep2 man_Ldep1 man_Ldep2 miha_Ldep1 miha_Ld

> ep2 {

2. lab var `var' "Longit dep result at REVIEW level"

3. }

.

. ds *_anx*

sug_anx1 sug_anx3 gonz_anx2 gonz_anx4 miha_anx2 miha_anx4 king_anx2 man_anx2 kuon_anx2 kuint_anx2

sug_anx2 gonz_anx1 gonz_anx3 miha_anx1 miha_anx3 king_anx1 man_anx1 kuon_anx1 kuint_anx1 kuint_anx3

. foreach var in sug_anx1 king_anx1 king_anx2 kuint_anx1 kuint_anx2 man_anx1 kuint_anx3 man_anx2 k

> uon_anx1 miha_anx1 kuon_anx2 miha_anx2 miha_anx3 miha_anx4 {

2. lab var `var' "C-S anx results at REVIEW level"

3. }

. foreach var in miha_anx2 king_Lanx1 king_Lanx2 kuint_Lanx1 man_Lanx1 kuint_Lanx2 man_Lanx2 kuon_Lanx2 miha_Lanx1

> kuon_Lanx1 {

2. lab var `var' "Longit anx result at REVIEW level"

3. }

. ds Ldepmu*vote

Ldepmu1vote Ldepmu2vote Ldepmu3vote Ldepmu4vote Ldepmu5vote

. foreach var in Ldepmu1vote Ldepmu2vote Ldepmu3vote Ldepmu4vote Ldepmu5vote {

2. lab var `var' "Longit multiv dep results STUDY lvl"

3. }

. ds Ldepbi*vote

Ldepbi1vote Ldepbi2vote Ldepbi3vote Ldepbi4vote Ldepbi5vote Ldepbi6vote

. foreach var in Ldepbi1vote Ldepbi3vote Ldepbi5vote Ldepbi2vote Ldepbi4vote Ldepbi6vote {

2. lab var `var' "Longit bivar dep results STUDY lvl"

3. }

. ds Lanxbi*vote

Lanxbi1vote Lanxbi2vote Lanxbi3vote Lanxbi4vote Lanxbi5vote Lanxbi6vote

. foreach var in Lanxbi1vote Lanxbi3vote Lanxbi5vote Lanxbi2vote Lanxbi4vote Lanxbi6vote {

2. lab var `var' "Longit bivar anx results STUDY lvl"

3. }

. foreach var in sug_dep1 kuon_dep1 kuon_dep2 kuon_dep1 kuon_dep2 king_dep1 man_dep1 man_dep2 miha_dep1 miha_dep3 miha_d

> ep2 miha_dep4 kuint_dep1 kuint_dep2 {

2. lab var `var' "C-S dep result at REVIEW level"

3. }

. foreach var in kuon_dep1 kuon_dep2 kuon_dep1 kuon_dep2 king_dep1 man_dep1 man_dep2 miha_dep1 miha_dep3 miha_dep2 mi

> ha_dep4 kuint_dep1 kuint_dep2 {

2. lab var `var' "C-S dep result at REVIEW level"

3. }

. ds Lanxmu*vote

Lanxmu1vote Lanxmu3vote Lanxmu5vote Lanxmu7vote Lanxmu9vote Lanxmu11vote

Lanxmu2vote Lanxmu4vote Lanxmu6vote Lanxmu8vote Lanxmu10vote

. foreach var in Lanxmu1vote Lanxmu3vote Lanxmu7vote Lanxmu10vote Lanxmu4vote Lanxmu8vote Lanxmu11vote Lanxmu5vote Lanxmu9

> vote Lanxmu2vote Lanxmu6vote {

2. lab var `var' "Longit multiv anx result STUDY lvl"

3. }

. ds *txt

depmu1txt anxmu3txt anxmu9txt depbi6txt anxbi3txt anxbi9txt Ldepbi6txt Lanxbi6txt Lanxmu1txt Lanxmu7txt

depmu2txt anxmu4txt depbi1txt depbi7txt anxbi4txt Ldepbi1txt Lanxbi1txt Ldepmu1txt Lanxmu2txt Lanxmu8txt

depmu3txt anxmu5txt depbi2txt depbi8txt anxbi5txt Ldepbi2txt Lanxbi2txt Ldepmu2txt Lanxmu3txt Lanxmu9txt

depmu4txt anxmu6txt depbi3txt depbi9txt anxbi6txt Ldepbi3txt Lanxbi3txt Ldepmu3txt Lanxmu4txt Lanxmu10txt

anxmu1txt anxmu7txt depbi4txt anxbi1txt anxbi7txt Ldepbi4txt Lanxbi4txt Ldepmu4txt Lanxmu5txt Lanxmu11txt

anxmu2txt anxmu8txt depbi5txt anxbi2txt anxbi8txt Ldepbi5txt Lanxbi5txt Ldepmu5txt Lanxmu6txt

. foreach var in anxbi7txt Lanxbi6txt anxbi8txt ///

> Lanxmu1txt anxbi9txt Lanxmu10txt anxmu1txt ///

> Lanxmu11txt anxmu2txt Lanxmu2txt anxmu3txt ///

> Lanxmu3txt anxmu4txt Lanxmu4txt anxmu5txt anxmu6txt ///

> Lanxmu5txt depbi1txt Lanxmu6txt depbi2txt ///

> Lanxmu7txt depbi3txt Lanxmu8txt depbi4txt ///

> Lanxmu9txt depbi5txt Ldepbi1txt depbi6txt ///

> Ldepbi2txt depbi7txt Ldepbi3txt depbi8txt ///

> Ldepbi4txt depbi9txt Ldepbi5txt depmu1txt ///

> Ldepbi6txt depmu2txt Ldepmu1txt anxbi1txt depmu3txt depmu4txt ///

> Ldepmu2txt anxbi2txt Lanxbi1txt Ldepmu3txt anxbi3txt Lanxbi2txt ///

> Ldepmu4txt anxbi4txt Lanxbi3txt Ldepmu5txt anxbi5txt Lanxbi4txt ///

> anxbi6txt Lanxbi5txt anxmu7txt anxmu8txt anxmu9txt {

2. lab var `var' "Free text: specific assoc. b/w dep/anx and PG/IA"

3. }

.

.

. save "/Users/michellecarras/Dropbox/Sys rev of sys rev Revisions/PLOS ONE submission/New submission July 2020/OSF additions/Databa

> se.dta", replace

file /Users/michellecarras/Dropbox/Sys rev of sys rev Revisions/PLOS ONE submission/New submission July 2020/OSF additions/Database.

> dta saved

.

. *****************************************************************************

. *

> *

. * SECTION 4

> *

. * ANALYSIS VARIABLE CREATION

> *

. *

> *

. *****************************************************************************

.

.

. codebook anxbi*txt, compact

Variable Obs Unique Mean Min Max Label

------------------------------------------------------------------------------------------------------------------------------------

anxbi1txt 35 31 . . . Free text: specific assoc. b/w dep/anx and PG/IA

anxbi2txt 19 19 . . . Free text: specific assoc. b/w dep/anx and PG/IA

anxbi3txt 9 9 . . . Free text: specific assoc. b/w dep/anx and PG/IA

anxbi4txt 5 5 . . . Free text: specific assoc. b/w dep/anx and PG/IA

anxbi5txt 3 3 . . . Free text: specific assoc. b/w dep/anx and PG/IA

anxbi6txt 2 2 . . . Free text: specific assoc. b/w dep/anx and PG/IA

anxbi7txt 1 1 . . . Free text: specific assoc. b/w dep/anx and PG/IA

anxbi8txt 1 1 . . . Free text: specific assoc. b/w dep/anx and PG/IA

anxbi9txt 1 1 . . . Free text: specific assoc. b/w dep/anx and PG/IA

------------------------------------------------------------------------------------------------------------------------------------

. codebook anxbi*vote, compact

Variable Obs Unique Mean Min Max Label

------------------------------------------------------------------------------------------------------------------------------------

anxbi1vote 34 2 .7647059 0 1 C-S bivar anx result in study

anxbi2vote 19 2 .6842105 0 1 C-S bivar anx result in study

anxbi3vote 9 2 .6666667 0 1 C-S bivar anx result in study

anxbi4vote 5 2 .4 0 1 C-S bivar anx result in study

anxbi5vote 3 2 .3333333 0 1 C-S bivar anx result in study

anxbi6vote 2 2 .5 0 1 C-S bivar anx result in study

anxbi7vote 1 1 0 0 0 C-S bivar anx result in study

anxbi8vote 1 1 0 0 0 C-S bivar anx result in study

anxbi9vote 1 1 0 0 0 C-S bivar anx result in study

------------------------------------------------------------------------------------------------------------------------------------

. codebook anxmu*vote, compact

Variable Obs Unique Mean Min Max Label

------------------------------------------------------------------------------------------------------------------------------------

anxmu1vote 12 3 .3333333 -1 1 C-S multiv anx result in study

anxmu2vote 7 2 .4285714 0 1 C-S multiv anx result in study

anxmu3vote 2 2 .5 0 1 C-S multiv anx result in study

anxmu4vote 2 2 .5 0 1 C-S multiv anx result in study

anxmu5vote 1 1 1 1 1 C-S multiv anx result in study

anxmu6vote 1 1 1 1 1 C-S multiv anx result in study

anxmu7vote 1 1 1 1 1 C-S multiv anx result in study

anxmu8vote 1 1 1 1 1 C-S multiv anx result in study

anxmu9vote 1 1 1 1 1 C-S multiv anx result in study

------------------------------------------------------------------------------------------------------------------------------------

. codebook Lanxbi*vote, compact

Variable Obs Unique Mean Min Max Label

------------------------------------------------------------------------------------------------------------------------------------

Lanxbi1vote 0 0 . . . Longit bivar anx results STUDY lvl

Lanxbi2vote 0 0 . . . Longit bivar anx results STUDY lvl

Lanxbi3vote 0 0 . . . Longit bivar anx results STUDY lvl

Lanxbi4vote 0 0 . . . Longit bivar anx results STUDY lvl

Lanxbi5vote 0 0 . . . Longit bivar anx results STUDY lvl

Lanxbi6vote 0 0 . . . Longit bivar anx results STUDY lvl

------------------------------------------------------------------------------------------------------------------------------------

. codebook Lanxmu*vote, compact

Variable Obs Unique Mean Min Max Label

------------------------------------------------------------------------------------------------------------------------------------

Lanxmu1vote 2 1 1 1 1 Longit multiv anx result STUDY lvl

Lanxmu2vote 2 2 0 -1 1 Longit multiv anx result STUDY lvl

Lanxmu3vote 2 1 1 1 1 Longit multiv anx result STUDY lvl

Lanxmu4vote 2 1 1 1 1 Longit multiv anx result STUDY lvl

Lanxmu5vote 1 1 1 1 1 Longit multiv anx result STUDY lvl

Lanxmu6vote 1 1 1 1 1 Longit multiv anx result STUDY lvl

Lanxmu7vote 1 1 1 1 1 Longit multiv anx result STUDY lvl

Lanxmu8vote 1 1 1 1 1 Longit multiv anx result STUDY lvl

Lanxmu9vote 1 1 1 1 1 Longit multiv anx result STUDY lvl

Lanxmu10vote 1 1 1 1 1 Longit multiv anx result STUDY lvl

Lanxmu11vote 1 1 1 1 1 Longit multiv anx result STUDY lvl

------------------------------------------------------------------------------------------------------------------------------------

. codebook depbi*vote, compact

Variable Obs Unique Mean Min Max Label

------------------------------------------------------------------------------------------------------------------------------------

depbi1vote 42 2 .7142857 0 1 C-S biv dep result in study

depbi2vote 16 2 .875 0 1 C-S biv dep result in study

depbi3vote 4 1 1 1 1 C-S biv dep result in study

depbi4vote 1 1 0 0 0 C-S biv dep result in study

depbi5vote 1 1 0 0 0 C-S biv dep result in study

depbi6vote 1 1 0 0 0 C-S biv dep result in study

depbi7vote 1 1 1 1 1 C-S biv dep result in study

depbi8vote 1 1 0 0 0 C-S biv dep result in study

depbi9vote 0 0 . . . C-S biv dep result in study

------------------------------------------------------------------------------------------------------------------------------------

. codebook depmu*vote, compact

Variable Obs Unique Mean Min Max Label

------------------------------------------------------------------------------------------------------------------------------------

depmu1vote 18 2 .6666667 0 1 depmu1vote

depmu2vote 6 2 .8333333 0 1 depmu2vote

depmu3vote 2 1 1 1 1 depmu3vote

depmu4vote 1 1 1 1 1 depmu4vote

------------------------------------------------------------------------------------------------------------------------------------

. codebook Ldepbi*vote, compact

Variable Obs Unique Mean Min Max Label

------------------------------------------------------------------------------------------------------------------------------------

Ldepbi1vote 1 1 0 0 0 Longit bivar dep results STUDY lvl

Ldepbi2vote 1 1 0 0 0 Longit bivar dep results STUDY lvl

Ldepbi3vote 0 0 . . . Longit bivar dep results STUDY lvl

Ldepbi4vote 0 0 . . . Longit bivar dep results STUDY lvl

Ldepbi5vote 0 0 . . . Longit bivar dep results STUDY lvl

Ldepbi6vote 0 0 . . . Longit bivar dep results STUDY lvl

------------------------------------------------------------------------------------------------------------------------------------

. codebook Ldepmu*vote, compact

Variable Obs Unique Mean Min Max Label

------------------------------------------------------------------------------------------------------------------------------------

Ldepmu1vote 2 1 1 1 1 Longit multiv dep results STUDY lvl

Ldepmu2vote 1 1 1 1 1 Longit multiv dep results STUDY lvl

Ldepmu3vote 1 1 1 1 1 Longit multiv dep results STUDY lvl

Ldepmu4vote 1 1 1 1 1 Longit multiv dep results STUDY lvl

Ldepmu5vote 0 0 . . . Longit multiv dep results STUDY lvl

------------------------------------------------------------------------------------------------------------------------------------

.

. foreach var in anxbi1vote anxbi2vote anxbi3vote anxbi4vote anxbi5vote ///

> anxmu1vote anxmu2vote anxmu3vote anxmu4vote anxmu5vote anxmu6vote ///

> anxmu7vote anxmu8vote anxmu9vote Lanxbi1vote Lanxbi2vote Lanxbi3vote Lanxbi4vote Lanxbi5vote ///

> Lanxmu10vote Lanxmu11vote Lanxmu1vote Lanxmu2vote Lanxmu3vote ///

> depbi1vote depbi2vote depbi3vote depbi4vote depbi5vote ///

> depmu1vote depmu2vote depmu3vote depmu4vote ///

> Ldepbi1vote Ldepbi2vote Ldepbi3vote Ldepbi4vote Ldepbi5vote ///

> Ldepmu1vote Ldepmu2vote Ldepmu3vote Ldepmu4vote Ldepmu5vote {

2. list study id `var' if `var'==-1

3. lab values `var' vtct

4. }

+------------------------------+

| study id anxmu1~e |

|------------------------------|

41. | Andreass_Sch 3 -1 |

+------------------------------+

+----------------------------+

| study id Lanxmu.. |

|----------------------------|

22. | Coyne_2015 29 -1 |

+----------------------------+

. ***only 3 cases of inverse relationships so most vars will be 0/1

.

.

. *******create a new var that renames the abstracted results (B for both) but leave the source variables as is

. ******Strip the PG cases from the (combo var) orig abstracted results so they are only Internet

. *******Strip the PIU cases from the orig abstracted results so they are only gaming

.

.

. foreach var in anxbi1vote anxbi2vote anxbi3vote anxbi4vote anxbi5vote ///

> anxbi6vote anxbi7vote anxbi8vote anxbi9vote ///

> anxmu1vote anxmu2vote anxmu3vote anxmu4vote anxmu5vote anxmu6vote ///

> anxmu7vote anxmu8vote anxmu9vote ///

> Lanxbi1vote Lanxbi2vote Lanxbi3vote Lanxbi4vote Lanxbi5vote Lanxbi6vote ///

> Lanxmu1vote Lanxmu2vote Lanxmu3vote Lanxmu4vote Lanxmu5vote Lanxmu6vote ///

> Lanxmu7vote Lanxmu8vote Lanxmu9vote Lanxmu10vote Lanxmu11vote ///

> depbi1vote depbi2vote depbi3vote depbi4vote depbi5vote ///

> depbi6vote depbi7vote depbi8vote depbi9vote ///

> depmu1vote depmu2vote depmu3vote depmu4vote ///

> Ldepbi1vote Ldepbi2vote Ldepbi3vote Ldepbi4vote Ldepbi5vote Ldepbi6vote ///

> Ldepmu1vote Ldepmu2vote Ldepmu3vote Ldepmu4vote Ldepmu5vote {

2. lab values `var' vtct

3. cap drop B`var'

4. clonevar B`var'=`var'

5. lab var B`var' "Combo PG/IA `var'"

6. lab values B`var' vtct

7. cap drop I`var'

8. clonevar I`var'=`var'

9. lab var I`var' "Internet only`var'"

10. replace I`var'=. if wasitPIU==0 | wasitPIU==.

11. lab values I`var' vtct

12. cap drop G`var'

13. clonevar G`var'=`var'

14. lab var G`var' "Gaming only `var'"

15. replace G`var'=. if PGscaleYN==0 | PGscaleYN==.

16. lab values G`var' vtct

17. tab G`var' PGscaleYN, mi

18. tab I`var' wasitPIU, mi

19. tab G`var' I`var', mi

20. *table wasitPIU PGscaleYN B`var'

. }

(162 missing values generated)

(162 missing values generated)

(22 real changes made, 22 to missing)

(162 missing values generated)

(9 real changes made, 9 to missing)

Gaming |

only | PGscaleYN

anxbi1vote | 0 1 . | Total

-----------+---------------------------------+----------

0 | 0 3 0 | 3

1 | 0 22 0 | 22

. | 63 100 8 | 171

-----------+---------------------------------+----------

Total | 63 125 8 | 196

Internet |

onlyanxbi1 | Did the study meas. PIU?

vote | 0 1 . | Total

-----------+---------------------------------+----------

0 | 0 6 0 | 6

1 | 0 6 0 | 6

. | 127 49 8 | 184

-----------+---------------------------------+----------

Total | 127 61 8 | 196

Gaming |

only | Internet onlyanxbi1vote

anxbi1vote | 0 1 . | Total

-----------+---------------------------------+----------

0 | 1 0 2 | 3

1 | 0 3 19 | 22

. | 5 3 163 | 171

-----------+---------------------------------+----------

Total | 6 6 184 | 196

(177 missing values generated)

(177 missing values generated)

(14 real changes made, 14 to missing)

(177 missing values generated)

(3 real changes made, 3 to missing)

Gaming |

only | PGscaleYN

anxbi2vote | 0 1 . | Total

-----------+---------------------------------+----------

0 | 0 5 0 | 5

1 | 0 11 0 | 11

. | 63 109 8 | 180

-----------+---------------------------------+----------

Total | 63 125 8 | 196

Internet |

onlyanxbi2 | Did the study meas. PIU?

vote | 0 1 . | Total

-----------+---------------------------------+----------

0 | 0 3 0 | 3

1 | 0 2 0 | 2

. | 127 56 8 | 191

-----------+---------------------------------+----------

Total | 127 61 8 | 196

Gaming |

only | Internet onlyanxbi2vote

anxbi2vote | 0 1 . | Total

-----------+---------------------------------+----------

0 | 2 0 3 | 5

1 | 0 1 10 | 11

. | 1 1 178 | 180

-----------+---------------------------------+----------

Total | 3 2 191 | 196

(187 missing values generated)

(187 missing values generated)

(6 real changes made, 6 to missing)

(187 missing values generated)

(2 real changes made, 2 to missing)

Gaming |

only | PGscaleYN

anxbi3vote | 0 1 . | Total

-----------+---------------------------------+----------

0 | 0 2 0 | 2

1 | 0 5 0 | 5

. | 63 118 8 | 189

-----------+---------------------------------+----------

Total | 63 125 8 | 196

Internet |

onlyanxbi3 | Did the study meas. PIU?

vote | 0 1 . | Total

-----------+---------------------------------+----------

0 | 0 2 0 | 2

1 | 0 1 0 | 1

. | 127 58 8 | 193

-----------+---------------------------------+----------

Total | 127 61 8 | 196

Gaming |

only | Internet onlyanxbi3vote

anxbi3vote | 0 1 . | Total

-----------+---------------------------------+----------

0 | 2 0 0 | 2

1 | 0 0 5 | 5

. | 0 1 188 | 189

-----------+---------------------------------+----------

Total | 2 1 193 | 196

(191 missing values generated)

(191 missing values generated)

(4 real changes made, 4 to missing)

(191 missing values generated)

(1 real change made, 1 to missing)

Gaming |

only | PGscaleYN

anxbi4vote | 0 1 . | Total

-----------+---------------------------------+----------

0 | 0 2 0 | 2

1 | 0 2 0 | 2

. | 63 121 8 | 192

-----------+---------------------------------+----------

Total | 63 125 8 | 196

Internet |

onlyanxbi4 | Did the study meas. PIU?

vote | 0 1 . | Total

-----------+---------------------------------+----------

0 | 0 1 0 | 1

. | 127 60 8 | 195

-----------+---------------------------------+----------

Total | 127 61 8 | 196

Gaming | Internet

only | onlyanxbi4vote

anxbi4vote | 0 . | Total

-----------+----------------------+----------

0 | 1 1 | 2

1 | 0 2 | 2

. | 0 192 | 192

-----------+----------------------+----------

Total | 1 195 | 196

(193 missing values generated)

(193 missing values generated)

(2 real changes made, 2 to missing)

(193 missing values generated)

(0 real changes made)

Gaming |

only | PGscaleYN

anxbi5vote | 0 1 . | Total

-----------+---------------------------------+----------

0 | 0 2 0 | 2

1 | 0 1 0 | 1

. | 63 122 8 | 193

-----------+---------------------------------+----------

Total | 63 125 8 | 196

Internet |

onlyanxbi5 | Did the study meas. PIU?

vote | 0 1 . | Total

-----------+---------------------------------+----------

0 | 0 1 0 | 1

. | 127 60 8 | 195

-----------+---------------------------------+----------

Total | 127 61 8 | 196

Gaming | Internet

only | onlyanxbi5vote

anxbi5vote | 0 . | Total

-----------+----------------------+----------

0 | 1 1 | 2

1 | 0 1 | 1

. | 0 193 | 193

-----------+----------------------+----------

Total | 1 195 | 196

(194 missing values generated)

(194 missing values generated)

(1 real change made, 1 to missing)

(194 missing values generated)

(0 real changes made)

Gaming |

only | PGscaleYN

anxbi6vote | 0 1 . | Total

-----------+---------------------------------+----------

0 | 0 1 0 | 1

1 | 0 1 0 | 1

. | 63 123 8 | 194

-----------+---------------------------------+----------

Total | 63 125 8 | 196

Internet |

onlyanxbi6 | Did the study meas. PIU?

vote | 0 1 . | Total

-----------+---------------------------------+----------

0 | 0 1 0 | 1

. | 127 60 8 | 195

-----------+---------------------------------+----------

Total | 127 61 8 | 196

Gaming | Internet

only | onlyanxbi6vote

anxbi6vote | 0 . | Total

-----------+----------------------+----------

0 | 1 0 | 1

1 | 0 1 | 1

. | 0 194 | 194

-----------+----------------------+----------

Total | 1 195 | 196

(195 missing values generated)

(195 missing values generated)

(0 real changes made)

(195 missing values generated)

(0 real changes made)

Gaming |

only | PGscaleYN

anxbi7vote | 0 1 . | Total

-----------+---------------------------------+----------

0 | 0 1 0 | 1

. | 63 124 8 | 195

-----------+---------------------------------+----------

Total | 63 125 8 | 196

Internet |

onlyanxbi7 | Did the study meas. PIU?

vote | 0 1 . | Total

-----------+---------------------------------+----------

0 | 0 1 0 | 1

. | 127 60 8 | 195

-----------+---------------------------------+----------

Total | 127 61 8 | 196

Gaming | Internet

only | onlyanxbi7vote

anxbi7vote | 0 . | Total

-----------+----------------------+----------

0 | 1 0 | 1

. | 0 195 | 195

-----------+----------------------+----------

Total | 1 195 | 196

(195 missing values generated)

(195 missing values generated)

(0 real changes made)

(195 missing values generated)

(0 real changes made)

Gaming |

only | PGscaleYN

anxbi8vote | 0 1 . | Total

-----------+---------------------------------+----------

0 | 0 1 0 | 1

. | 63 124 8 | 195

-----------+---------------------------------+----------

Total | 63 125 8 | 196

Internet |

onlyanxbi8 | Did the study meas. PIU?

vote | 0 1 . | Total

-----------+---------------------------------+----------

0 | 0 1 0 | 1

. | 127 60 8 | 195

-----------+---------------------------------+----------

Total | 127 61 8 | 196

Gaming | Internet

only | onlyanxbi8vote

anxbi8vote | 0 . | Total

-----------+----------------------+----------

0 | 1 0 | 1

. | 0 195 | 195

-----------+----------------------+----------

Total | 1 195 | 196

(195 missing values generated)

(195 missing values generated)

(0 real changes made)

(195 missing values generated)

(0 real changes made)

Gaming |

only | PGscaleYN

anxbi9vote | 0 1 . | Total

-----------+---------------------------------+----------

0 | 0 1 0 | 1

. | 63 124 8 | 195

-----------+---------------------------------+----------

Total | 63 125 8 | 196

Internet |

onlyanxbi9 | Did the study meas. PIU?

vote | 0 1 . | Total

-----------+---------------------------------+----------

0 | 0 1 0 | 1

. | 127 60 8 | 195

-----------+---------------------------------+----------

Total | 127 61 8 | 196

Gaming | Internet

only | onlyanxbi9vote

anxbi9vote | 0 . | Total

-----------+----------------------+----------

0 | 1 0 | 1

. | 0 195 | 195

-----------+----------------------+----------

Total | 1 195 | 196

(184 missing values generated)

(184 missing values generated)

(5 real changes made, 5 to missing)

(184 missing values generated)

(5 real changes made, 5 to missing)

Gaming |

only | PGscaleYN

anxmu1vote | 0 1 . | Total

-----------+---------------------------------+----------

-1 | 0 1 0 | 1

0 | 0 3 0 | 3

1 | 0 3 0 | 3

. | 63 118 8 | 189

-----------+---------------------------------+----------

Total | 63 125 8 | 196

Internet |

onlyanxmu1 | Did the study meas. PIU?

vote | 0 1 . | Total

-----------+---------------------------------+----------

-1 | 0 1 0 | 1

0 | 0 4 0 | 4

1 | 0 2 0 | 2

. | 127 54 8 | 189

-----------+---------------------------------+----------

Total | 127 61 8 | 196

Gaming |

only | Internet onlyanxmu1vote

anxmu1vote | -1 0 1 . | Total

-----------+--------------------------------------------+----------

-1 | 1 0 0 0 | 1

0 | 0 1 0 2 | 3

1 | 0 0 0 3 | 3

. | 0 3 2 184 | 189

-----------+--------------------------------------------+----------

Total | 1 4 2 189 | 196

(189 missing values generated)

(189 missing values generated)

(3 real changes made, 3 to missing)

(189 missing values generated)

(4 real changes made, 4 to missing)

Gaming |

only | PGscaleYN

anxmu2vote | 0 1 . | Total

-----------+---------------------------------+----------

0 | 0 2 0 | 2

1 | 0 1 0 | 1

. | 63 122 8 | 193

-----------+---------------------------------+----------

Total | 63 125 8 | 196

Internet |

onlyanxmu2 | Did the study meas. PIU?

vote | 0 1 . | Total

-----------+---------------------------------+----------

0 | 0 2 0 | 2

1 | 0 2 0 | 2

. | 127 57 8 | 192

-----------+---------------------------------+----------

Total | 127 61 8 | 196

Gaming |

only | Internet onlyanxmu2vote

anxmu2vote | 0 1 . | Total

-----------+---------------------------------+----------

0 | 0 0 2 | 2

1 | 0 0 1 | 1

. | 2 2 189 | 193

-----------+---------------------------------+----------

Total | 2 2 192 | 196

(194 missing values generated)

(194 missing values generated)

(0 real changes made)

(194 missing values generated)

(2 real changes made, 2 to missing)

Gaming |

only | PGscaleYN

anxmu3vote | 0 1 . | Total

-----------+---------------------------------+----------

. | 63 125 8 | 196

-----------+---------------------------------+----------

Total | 63 125 8 | 196

Internet |

onlyanxmu3 | Did the study meas. PIU?

vote | 0 1 . | Total

-----------+---------------------------------+----------

0 | 0 1 0 | 1

1 | 0 1 0 | 1

. | 127 59 8 | 194

-----------+---------------------------------+----------

Total | 127 61 8 | 196

Gaming |

only | Internet onlyanxmu3vote

anxmu3vote | 0 1 . | Total

-----------+---------------------------------+----------

. | 1 1 194 | 196

-----------+---------------------------------+----------

Total | 1 1 194 | 196

(194 missing values generated)

(194 missing values generated)

(0 real changes made)

(194 missing values generated)

(2 real changes made, 2 to missing)

Gaming |

only | PGscaleYN

anxmu4vote | 0 1 . | Total

-----------+---------------------------------+----------

. | 63 125 8 | 196

-----------+---------------------------------+----------

Total | 63 125 8 | 196

Internet |

onlyanxmu4 | Did the study meas. PIU?

vote | 0 1 . | Total

-----------+---------------------------------+----------

0 | 0 1 0 | 1

1 | 0 1 0 | 1

. | 127 59 8 | 194

-----------+---------------------------------+----------

Total | 127 61 8 | 196

Gaming |

only | Internet onlyanxmu4vote

anxmu4vote | 0 1 . | Total

-----------+---------------------------------+----------

. | 1 1 194 | 196

-----------+---------------------------------+----------

Total | 1 1 194 | 196

(195 missing values generated)

(195 missing values generated)

(0 real changes made)

(195 missing values generated)

(1 real change made, 1 to missing)

Gaming |

only | PGscaleYN

anxmu5vote | 0 1 . | Total

-----------+---------------------------------+----------

. | 63 125 8 | 196

-----------+---------------------------------+----------

Total | 63 125 8 | 196

Internet |

onlyanxmu5 | Did the study meas. PIU?

vote | 0 1 . | Total

-----------+---------------------------------+----------

1 | 0 1 0 | 1

. | 127 60 8 | 195

-----------+---------------------------------+----------

Total | 127 61 8 | 196

Gaming | Internet

only | onlyanxmu5vote

anxmu5vote | 1 . | Total

-----------+----------------------+----------

. | 1 195 | 196

-----------+----------------------+----------

Total | 1 195 | 196

(195 missing values generated)

(195 missing values generated)

(0 real changes made)

(195 missing values generated)

(1 real change made, 1 to missing)

Gaming |

only | PGscaleYN

anxmu6vote | 0 1 . | Total

-----------+---------------------------------+----------

. | 63 125 8 | 196

-----------+---------------------------------+----------

Total | 63 125 8 | 196

Internet |

onlyanxmu6 | Did the study meas. PIU?

vote | 0 1 . | Total

-----------+---------------------------------+----------

1 | 0 1 0 | 1

. | 127 60 8 | 195

-----------+---------------------------------+----------

Total | 127 61 8 | 196

Gaming | Internet

only | onlyanxmu6vote

anxmu6vote | 1 . | Total

-----------+----------------------+----------

. | 1 195 | 196

-----------+----------------------+----------

Total | 1 195 | 196

(195 missing values generated)

(195 missing values generated)

(0 real changes made)

(195 missing values generated)

(1 real change made, 1 to missing)

Gaming |

only | PGscaleYN

anxmu7vote | 0 1 . | Total

-----------+---------------------------------+----------

. | 63 125 8 | 196

-----------+---------------------------------+----------

Total | 63 125 8 | 196

Internet |

onlyanxmu7 | Did the study meas. PIU?

vote | 0 1 . | Total

-----------+---------------------------------+----------

1 | 0 1 0 | 1

. | 127 60 8 | 195

-----------+---------------------------------+----------

Total | 127 61 8 | 196

Gaming | Internet

only | onlyanxmu7vote

anxmu7vote | 1 . | Total

-----------+----------------------+----------

. | 1 195 | 196

-----------+----------------------+----------

Total | 1 195 | 196

(195 missing values generated)

(195 missing values generated)

(0 real changes made)

(195 missing values generated)

(1 real change made, 1 to missing)

Gaming |

only | PGscaleYN

anxmu8vote | 0 1 . | Total

-----------+---------------------------------+----------

. | 63 125 8 | 196

-----------+---------------------------------+----------

Total | 63 125 8 | 196

Internet |

onlyanxmu8 | Did the study meas. PIU?

vote | 0 1 . | Total

-----------+---------------------------------+----------

1 | 0 1 0 | 1

. | 127 60 8 | 195

-----------+---------------------------------+----------

Total | 127 61 8 | 196

Gaming | Internet

only | onlyanxmu8vote

anxmu8vote | 1 . | Total

-----------+----------------------+----------

. | 1 195 | 196

-----------+----------------------+----------

Total | 1 195 | 196

(195 missing values generated)

(195 missing values generated)

(0 real changes made)

(195 missing values generated)

(1 real change made, 1 to missing)

Gaming |

only | PGscaleYN

anxmu9vote | 0 1 . | Total

-----------+---------------------------------+----------

. | 63 125 8 | 196

-----------+---------------------------------+----------

Total | 63 125 8 | 196

Internet |

onlyanxmu9 | Did the study meas. PIU?

vote | 0 1 . | Total

-----------+---------------------------------+----------

1 | 0 1 0 | 1

. | 127 60 8 | 195

-----------+---------------------------------+----------

Total | 127 61 8 | 196

Gaming | Internet

only | onlyanxmu9vote

anxmu9vote | 1 . | Total

-----------+----------------------+----------

. | 1 195 | 196

-----------+----------------------+----------

Total | 1 195 | 196

(196 missing values generated)

(196 missing values generated)

(0 real changes made)

(196 missing values generated)

(0 real changes made)

Gaming |

only |

Lanxbi1vot | PGscaleYN

e | 0 1 . | Total

-----------+---------------------------------+----------

. | 63 125 8 | 196

-----------+---------------------------------+----------

Total | 63 125 8 | 196

Internet |

onlyLanxbi | Did the study meas. PIU?

1vote | 0 1 . | Total

-----------+---------------------------------+----------

. | 127 61 8 | 196

-----------+---------------------------------+----------

Total | 127 61 8 | 196

Gaming | Internet

only | onlyLanxbi

Lanxbi1vot | 1vote

e | . | Total

-----------+-----------+----------

. | 196 | 196

-----------+-----------+----------

Total | 196 | 196

(196 missing values generated)

(196 missing values generated)

(0 real changes made)

(196 missing values generated)

(0 real changes made)

Gaming |

only |

Lanxbi2vot | PGscaleYN

e | 0 1 . | Total

-----------+---------------------------------+----------

. | 63 125 8 | 196

-----------+---------------------------------+----------

Total | 63 125 8 | 196

Internet |

onlyLanxbi | Did the study meas. PIU?

2vote | 0 1 . | Total

-----------+---------------------------------+----------

. | 127 61 8 | 196

-----------+---------------------------------+----------

Total | 127 61 8 | 196

Gaming | Internet

only | onlyLanxbi

Lanxbi2vot | 2vote

e | . | Total

-----------+-----------+----------

. | 196 | 196

-----------+-----------+----------

Total | 196 | 196

(196 missing values generated)

(196 missing values generated)

(0 real changes made)

(196 missing values generated)

(0 real changes made)

Gaming |

only |

Lanxbi3vot | PGscaleYN

e | 0 1 . | Total

-----------+---------------------------------+----------

. | 63 125 8 | 196

-----------+---------------------------------+----------

Total | 63 125 8 | 196

Internet |

onlyLanxbi | Did the study meas. PIU?

3vote | 0 1 . | Total

-----------+---------------------------------+----------

. | 127 61 8 | 196

-----------+---------------------------------+----------

Total | 127 61 8 | 196

Gaming | Internet

only | onlyLanxbi

Lanxbi3vot | 3vote

e | . | Total

-----------+-----------+----------

. | 196 | 196

-----------+-----------+----------

Total | 196 | 196

(196 missing values generated)

(196 missing values generated)

(0 real changes made)

(196 missing values generated)

(0 real changes made)

Gaming |

only |

Lanxbi4vot | PGscaleYN

e | 0 1 . | Total

-----------+---------------------------------+----------

. | 63 125 8 | 196

-----------+---------------------------------+----------

Total | 63 125 8 | 196

Internet |

onlyLanxbi | Did the study meas. PIU?

4vote | 0 1 . | Total

-----------+---------------------------------+----------

. | 127 61 8 | 196

-----------+---------------------------------+----------

Total | 127 61 8 | 196

Gaming | Internet

only | onlyLanxbi

Lanxbi4vot | 4vote

e | . | Total

-----------+-----------+----------

. | 196 | 196

-----------+-----------+----------

Total | 196 | 196

(196 missing values generated)

(196 missing values generated)

(0 real changes made)

(196 missing values generated)

(0 real changes made)

Gaming |

only |

Lanxbi5vot | PGscaleYN

e | 0 1 . | Total

-----------+---------------------------------+----------

. | 63 125 8 | 196

-----------+---------------------------------+----------

Total | 63 125 8 | 196

Internet |

onlyLanxbi | Did the study meas. PIU?

5vote | 0 1 . | Total

-----------+---------------------------------+----------

. | 127 61 8 | 196

-----------+---------------------------------+----------

Total | 127 61 8 | 196

Gaming | Internet

only | onlyLanxbi

Lanxbi5vot | 5vote

e | . | Total

-----------+-----------+----------

. | 196 | 196

-----------+-----------+----------

Total | 196 | 196

(196 missing values generated)

(196 missing values generated)

(0 real changes made)

(196 missing values generated)

(0 real changes made)

Gaming |

only |

Lanxbi6vot | PGscaleYN

e | 0 1 . | Total

-----------+---------------------------------+----------

. | 63 125 8 | 196

-----------+---------------------------------+----------

Total | 63 125 8 | 196

Internet |

onlyLanxbi | Did the study meas. PIU?

6vote | 0 1 . | Total

-----------+---------------------------------+----------

. | 127 61 8 | 196

-----------+---------------------------------+----------

Total | 127 61 8 | 196

Gaming | Internet

only | onlyLanxbi

Lanxbi6vot | 6vote

e | . | Total

-----------+-----------+----------

. | 196 | 196

-----------+-----------+----------

Total | 196 | 196

(194 missing values generated)

(194 missing values generated)

(2 real changes made, 2 to missing)

(194 missing values generated)

(0 real changes made)

Gaming |

only |

Lanxmu1vot | PGscaleYN

e | 0 1 . | Total

-----------+---------------------------------+----------

1 | 0 2 0 | 2

. | 63 123 8 | 194

-----------+---------------------------------+----------

Total | 63 125 8 | 196

Internet |

onlyLanxmu | Did the study meas. PIU?

1vote | 0 1 . | Total

-----------+---------------------------------+----------

. | 127 61 8 | 196

-----------+---------------------------------+----------

Total | 127 61 8 | 196

Gaming | Internet

only | onlyLanxmu

Lanxmu1vot | 1vote

e | . | Total

-----------+-----------+----------

1 | 2 | 2

. | 194 | 194

-----------+-----------+----------

Total | 196 | 196

(194 missing values generated)

(194 missing values generated)

(2 real changes made, 2 to missing)

(194 missing values generated)

(0 real changes made)

Gaming |

only |

Lanxmu2vot | PGscaleYN

e | 0 1 . | Total

-----------+---------------------------------+----------

-1 | 0 1 0 | 1

1 | 0 1 0 | 1

. | 63 123 8 | 194

-----------+---------------------------------+----------

Total | 63 125 8 | 196

Internet |

onlyLanxmu | Did the study meas. PIU?

2vote | 0 1 . | Total

-----------+---------------------------------+----------

. | 127 61 8 | 196

-----------+---------------------------------+----------

Total | 127 61 8 | 196

Gaming | Internet

only | onlyLanxmu

Lanxmu2vot | 2vote

e | . | Total

-----------+-----------+----------

-1 | 1 | 1

1 | 1 | 1

. | 194 | 194

-----------+-----------+----------

Total | 196 | 196

(194 missing values generated)

(194 missing values generated)

(2 real changes made, 2 to missing)

(194 missing values generated)

(0 real changes made)

Gaming |

only |

Lanxmu3vot | PGscaleYN

e | 0 1 . | Total

-----------+---------------------------------+----------

1 | 0 2 0 | 2

. | 63 123 8 | 194

-----------+---------------------------------+----------

Total | 63 125 8 | 196

Internet |

onlyLanxmu | Did the study meas. PIU?

3vote | 0 1 . | Total

-----------+---------------------------------+----------

. | 127 61 8 | 196

-----------+---------------------------------+----------

Total | 127 61 8 | 196

Gaming | Internet

only | onlyLanxmu

Lanxmu3vot | 3vote

e | . | Total

-----------+-----------+----------

1 | 2 | 2

. | 194 | 194

-----------+-----------+----------

Total | 196 | 196

(194 missing values generated)

(194 missing values generated)

(2 real changes made, 2 to missing)

(194 missing values generated)

(0 real changes made)

Gaming |

only |

Lanxmu4vot | PGscaleYN

e | 0 1 . | Total

-----------+---------------------------------+----------

1 | 0 2 0 | 2

. | 63 123 8 | 194

-----------+---------------------------------+----------

Total | 63 125 8 | 196

Internet |

onlyLanxmu | Did the study meas. PIU?

4vote | 0 1 . | Total

-----------+---------------------------------+----------

. | 127 61 8 | 196

-----------+---------------------------------+----------

Total | 127 61 8 | 196

Gaming | Internet

only | onlyLanxmu

Lanxmu4vot | 4vote

e | . | Total

-----------+-----------+----------

1 | 2 | 2

. | 194 | 194

-----------+-----------+----------

Total | 196 | 196

(195 missing values generated)

(195 missing values generated)

(1 real change made, 1 to missing)

(195 missing values generated)

(0 real changes made)

Gaming |

only |

Lanxmu5vot | PGscaleYN

e | 0 1 . | Total

-----------+---------------------------------+----------

1 | 0 1 0 | 1

. | 63 124 8 | 195

-----------+---------------------------------+----------

Total | 63 125 8 | 196

Internet |

onlyLanxmu | Did the study meas. PIU?

5vote | 0 1 . | Total

-----------+---------------------------------+----------

. | 127 61 8 | 196

-----------+---------------------------------+----------

Total | 127 61 8 | 196

Gaming | Internet

only | onlyLanxmu

Lanxmu5vot | 5vote

e | . | Total

-----------+-----------+----------

1 | 1 | 1

. | 195 | 195

-----------+-----------+----------

Total | 196 | 196

(195 missing values generated)

(195 missing values generated)

(1 real change made, 1 to missing)

(195 missing values generated)

(0 real changes made)

Gaming |

only |

Lanxmu6vot | PGscaleYN

e | 0 1 . | Total

-----------+---------------------------------+----------

1 | 0 1 0 | 1

. | 63 124 8 | 195

-----------+---------------------------------+----------

Total | 63 125 8 | 196

Internet |

onlyLanxmu | Did the study meas. PIU?

6vote | 0 1 . | Total

-----------+---------------------------------+----------

. | 127 61 8 | 196

-----------+---------------------------------+----------

Total | 127 61 8 | 196

Gaming | Internet

only | onlyLanxmu

Lanxmu6vot | 6vote

e | . | Total

-----------+-----------+----------

1 | 1 | 1

. | 195 | 195

-----------+-----------+----------

Total | 196 | 196

(195 missing values generated)

(195 missing values generated)

(1 real change made, 1 to missing)

(195 missing values generated)

(0 real changes made)

Gaming |

only |

Lanxmu7vot | PGscaleYN

e | 0 1 . | Total

-----------+---------------------------------+----------

1 | 0 1 0 | 1

. | 63 124 8 | 195

-----------+---------------------------------+----------

Total | 63 125 8 | 196

Internet |

onlyLanxmu | Did the study meas. PIU?

7vote | 0 1 . | Total

-----------+---------------------------------+----------

. | 127 61 8 | 196

-----------+---------------------------------+----------

Total | 127 61 8 | 196

Gaming | Internet

only | onlyLanxmu

Lanxmu7vot | 7vote

e | . | Total

-----------+-----------+----------

1 | 1 | 1

. | 195 | 195

-----------+-----------+----------

Total | 196 | 196

(195 missing values generated)

(195 missing values generated)

(1 real change made, 1 to missing)

(195 missing values generated)

(0 real changes made)

Gaming |

only |

Lanxmu8vot | PGscaleYN

e | 0 1 . | Total

-----------+---------------------------------+----------

1 | 0 1 0 | 1

. | 63 124 8 | 195

-----------+---------------------------------+----------

Total | 63 125 8 | 196

Internet |

onlyLanxmu | Did the study meas. PIU?

8vote | 0 1 . | Total

-----------+---------------------------------+----------

. | 127 61 8 | 196

-----------+---------------------------------+----------

Total | 127 61 8 | 196

Gaming | Internet

only | onlyLanxmu

Lanxmu8vot | 8vote

e | . | Total

-----------+-----------+----------

1 | 1 | 1

. | 195 | 195

-----------+-----------+----------

Total | 196 | 196

(195 missing values generated)

(195 missing values generated)

(1 real change made, 1 to missing)

(195 missing values generated)

(0 real changes made)

Gaming |

only |

Lanxmu9vot | PGscaleYN

e | 0 1 . | Total

-----------+---------------------------------+----------

1 | 0 1 0 | 1

. | 63 124 8 | 195

-----------+---------------------------------+----------

Total | 63 125 8 | 196

Internet |

onlyLanxmu | Did the study meas. PIU?

9vote | 0 1 . | Total

-----------+---------------------------------+----------

. | 127 61 8 | 196

-----------+---------------------------------+----------

Total | 127 61 8 | 196

Gaming | Internet

only | onlyLanxmu

Lanxmu9vot | 9vote

e | . | Total

-----------+-----------+----------

1 | 1 | 1

. | 195 | 195

-----------+-----------+----------

Total | 196 | 196

(195 missing values generated)

(195 missing values generated)

(1 real change made, 1 to missing)

(195 missing values generated)

(0 real changes made)

Gaming |

only |

Lanxmu10vo | PGscaleYN

te | 0 1 . | Total

-----------+---------------------------------+----------

1 | 0 1 0 | 1

. | 63 124 8 | 195

-----------+---------------------------------+----------

Total | 63 125 8 | 196

Internet |

onlyLanxmu | Did the study meas. PIU?

10vote | 0 1 . | Total

-----------+---------------------------------+----------

. | 127 61 8 | 196

-----------+---------------------------------+----------

Total | 127 61 8 | 196

Gaming | Internet

only | onlyLanxmu

Lanxmu10vo | 10vote

te | . | Total

-----------+-----------+----------

1 | 1 | 1

. | 195 | 195

-----------+-----------+----------

Total | 196 | 196

(195 missing values generated)

(195 missing values generated)

(1 real change made, 1 to missing)

(195 missing values generated)

(0 real changes made)

Gaming |

only |

Lanxmu11vo | PGscaleYN

te | 0 1 . | Total

-----------+---------------------------------+----------

1 | 0 1 0 | 1

. | 63 124 8 | 195

-----------+---------------------------------+----------

Total | 63 125 8 | 196

Internet |

onlyLanxmu | Did the study meas. PIU?

11vote | 0 1 . | Total

-----------+---------------------------------+----------

. | 127 61 8 | 196

-----------+---------------------------------+----------

Total | 127 61 8 | 196

Gaming | Internet

only | onlyLanxmu

Lanxmu11vo | 11vote

te | . | Total

-----------+-----------+----------

1 | 1 | 1

. | 195 | 195

-----------+-----------+----------

Total | 196 | 196

(154 missing values generated)

(154 missing values generated)

(23 real changes made, 23 to missing)

(154 missing values generated)

(15 real changes made, 15 to missing)

Gaming |

only | PGscaleYN

depbi1vote | 0 1 . | Total

-----------+---------------------------------+----------

0 | 0 3 0 | 3

1 | 0 24 0 | 24

. | 63 98 8 | 169

-----------+---------------------------------+----------

Total | 63 125 8 | 196

Internet |

onlydepbi1 | Did the study meas. PIU?

vote | 0 1 . | Total

-----------+---------------------------------+----------

0 | 0 9 0 | 9

1 | 0 10 0 | 10

. | 127 42 8 | 177

-----------+---------------------------------+----------

Total | 127 61 8 | 196

Gaming |

only | Internet onlydepbi1vote

depbi1vote | 0 1 . | Total

-----------+---------------------------------+----------

0 | 1 0 2 | 3

1 | 0 4 20 | 24

. | 8 6 155 | 169

-----------+---------------------------------+----------

Total | 9 10 177 | 196

(180 missing values generated)

(180 missing values generated)

(11 real changes made, 11 to missing)

(180 missing values generated)

(4 real changes made, 4 to missing)

Gaming |

only | PGscaleYN

depbi2vote | 0 1 . | Total

-----------+---------------------------------+----------

0 | 0 1 0 | 1

1 | 0 11 0 | 11

. | 63 113 8 | 184

-----------+---------------------------------+----------

Total | 63 125 8 | 196

Internet |

onlydepbi2 | Did the study meas. PIU?

vote | 0 1 . | Total

-----------+---------------------------------+----------

1 | 0 5 0 | 5

. | 127 56 8 | 191

-----------+---------------------------------+----------

Total | 127 61 8 | 196

Gaming | Internet

only | onlydepbi2vote

depbi2vote | 1 . | Total

-----------+----------------------+----------

0 | 0 1 | 1

1 | 2 9 | 11

. | 3 181 | 184

-----------+----------------------+----------

Total | 5 191 | 196

(192 missing values generated)

(192 missing values generated)

(3 real changes made, 3 to missing)

(192 missing values generated)

(0 real changes made)

Gaming |

only | PGscaleYN

depbi3vote | 0 1 . | Total

-----------+---------------------------------+----------

1 | 0 4 0 | 4

. | 63 121 8 | 192

-----------+---------------------------------+----------

Total | 63 125 8 | 196

Internet |

onlydepbi3 | Did the study meas. PIU?

vote | 0 1 . | Total

-----------+---------------------------------+----------

1 | 0 1 0 | 1

. | 127 60 8 | 195

-----------+---------------------------------+----------

Total | 127 61 8 | 196

Gaming | Internet

only | onlydepbi3vote

depbi3vote | 1 . | Total

-----------+----------------------+----------

1 | 1 3 | 4

. | 0 192 | 192

-----------+----------------------+----------

Total | 1 195 | 196

(195 missing values generated)

(195 missing values generated)

(1 real change made, 1 to missing)

(195 missing values generated)

(0 real changes made)

Gaming |

only | PGscaleYN

depbi4vote | 0 1 . | Total

-----------+---------------------------------+----------

0 | 0 1 0 | 1

. | 63 124 8 | 195

-----------+---------------------------------+----------

Total | 63 125 8 | 196

Internet |

onlydepbi4 | Did the study meas. PIU?

vote | 0 1 . | Total

-----------+---------------------------------+----------

. | 127 61 8 | 196

-----------+---------------------------------+----------

Total | 127 61 8 | 196

| Internet

Gaming | onlydepbi4

only | vote

depbi4vote | . | Total

-----------+-----------+----------

0 | 1 | 1

. | 195 | 195

-----------+-----------+----------

Total | 196 | 196

(195 missing values generated)

(195 missing values generated)

(1 real change made, 1 to missing)

(195 missing values generated)

(0 real changes made)

Gaming |

only | PGscaleYN

depbi5vote | 0 1 . | Total

-----------+---------------------------------+----------

0 | 0 1 0 | 1

. | 63 124 8 | 195

-----------+---------------------------------+----------

Total | 63 125 8 | 196

Internet |

onlydepbi5 | Did the study meas. PIU?

vote | 0 1 . | Total

-----------+---------------------------------+----------

. | 127 61 8 | 196

-----------+---------------------------------+----------

Total | 127 61 8 | 196

| Internet

Gaming | onlydepbi5

only | vote

depbi5vote | . | Total

-----------+-----------+----------

0 | 1 | 1

. | 195 | 195

-----------+-----------+----------

Total | 196 | 196

(195 missing values generated)

(195 missing values generated)

(1 real change made, 1 to missing)

(195 missing values generated)

(0 real changes made)

Gaming |

only | PGscaleYN

depbi6vote | 0 1 . | Total

-----------+---------------------------------+----------

0 | 0 1 0 | 1

. | 63 124 8 | 195

-----------+---------------------------------+----------

Total | 63 125 8 | 196

Internet |

onlydepbi6 | Did the study meas. PIU?

vote | 0 1 . | Total

-----------+---------------------------------+----------

. | 127 61 8 | 196

-----------+---------------------------------+----------

Total | 127 61 8 | 196

| Internet

Gaming | onlydepbi6

only | vote

depbi6vote | . | Total

-----------+-----------+----------

0 | 1 | 1

. | 195 | 195

-----------+-----------+----------

Total | 196 | 196

(195 missing values generated)

(195 missing values generated)

(1 real change made, 1 to missing)

(195 missing values generated)

(0 real changes made)

Gaming |

only | PGscaleYN

depbi7vote | 0 1 . | Total

-----------+---------------------------------+----------

1 | 0 1 0 | 1

. | 63 124 8 | 195

-----------+---------------------------------+----------

Total | 63 125 8 | 196

Internet |

onlydepbi7 | Did the study meas. PIU?

vote | 0 1 . | Total

-----------+---------------------------------+----------

. | 127 61 8 | 196

-----------+---------------------------------+----------

Total | 127 61 8 | 196

| Internet

Gaming | onlydepbi7

only | vote

depbi7vote | . | Total

-----------+-----------+----------

1 | 1 | 1

. | 195 | 195

-----------+-----------+----------

Total | 196 | 196

(195 missing values generated)

(195 missing values generated)

(1 real change made, 1 to missing)

(195 missing values generated)

(0 real changes made)

Gaming |

only | PGscaleYN

depbi8vote | 0 1 . | Total

-----------+---------------------------------+----------

0 | 0 1 0 | 1

. | 63 124 8 | 195

-----------+---------------------------------+----------

Total | 63 125 8 | 196

Internet |

onlydepbi8 | Did the study meas. PIU?

vote | 0 1 . | Total

-----------+---------------------------------+----------

. | 127 61 8 | 196

-----------+---------------------------------+----------

Total | 127 61 8 | 196

| Internet

Gaming | onlydepbi8

only | vote

depbi8vote | . | Total

-----------+-----------+----------

0 | 1 | 1

. | 195 | 195

-----------+-----------+----------

Total | 196 | 196

(196 missing values generated)

(196 missing values generated)

(0 real changes made)

(196 missing values generated)

(0 real changes made)

Gaming |

only | PGscaleYN

depbi9vote | 0 1 . | Total

-----------+---------------------------------+----------

. | 63 125 8 | 196

-----------+---------------------------------+----------

Total | 63 125 8 | 196

Internet |

onlydepbi9 | Did the study meas. PIU?

vote | 0 1 . | Total

-----------+---------------------------------+----------

. | 127 61 8 | 196

-----------+---------------------------------+----------

Total | 127 61 8 | 196

| Internet

Gaming | onlydepbi9

only | vote

depbi9vote | . | Total

-----------+-----------+----------

. | 196 | 196

-----------+-----------+----------

Total | 196 | 196

(178 missing values generated)

(178 missing values generated)

(12 real changes made, 12 to missing)

(178 missing values generated)

(5 real changes made, 5 to missing)

Gaming |

only | PGscaleYN

depmu1vote | 0 1 . | Total

-----------+---------------------------------+----------

0 | 0 5 0 | 5

1 | 0 8 0 | 8

. | 63 112 8 | 183

-----------+---------------------------------+----------

Total | 63 125 8 | 196

Internet |

onlydepmu1 | Did the study meas. PIU?

vote | 0 1 . | Total

-----------+---------------------------------+----------

0 | 0 1 0 | 1

1 | 0 5 0 | 5

. | 127 55 8 | 190

-----------+---------------------------------+----------

Total | 127 61 8 | 196

Gaming |

only | Internet onlydepmu1vote

depmu1vote | 0 1 . | Total

-----------+---------------------------------+----------

0 | 0 0 5 | 5

1 | 0 1 7 | 8

. | 1 4 178 | 183

-----------+---------------------------------+----------

Total | 1 5 190 | 196

(190 missing values generated)

(190 missing values generated)

(3 real changes made, 3 to missing)

(190 missing values generated)

(3 real changes made, 3 to missing)

Gaming |

only | PGscaleYN

depmu2vote | 0 1 . | Total

-----------+---------------------------------+----------

0 | 0 1 0 | 1

1 | 0 2 0 | 2

. | 63 122 8 | 193

-----------+---------------------------------+----------

Total | 63 125 8 | 196

Internet |

onlydepmu2 | Did the study meas. PIU?

vote | 0 1 . | Total

-----------+---------------------------------+----------

1 | 0 3 0 | 3

. | 127 58 8 | 193

-----------+---------------------------------+----------

Total | 127 61 8 | 196

Gaming | Internet

only | onlydepmu2vote

depmu2vote | 1 . | Total

-----------+----------------------+----------

0 | 0 1 | 1

1 | 0 2 | 2

. | 3 190 | 193

-----------+----------------------+----------

Total | 3 193 | 196

(194 missing values generated)

(194 missing values generated)

(1 real change made, 1 to missing)

(194 missing values generated)

(1 real change made, 1 to missing)

Gaming |

only | PGscaleYN

depmu3vote | 0 1 . | Total

-----------+---------------------------------+----------

1 | 0 1 0 | 1

. | 63 124 8 | 195

-----------+---------------------------------+----------

Total | 63 125 8 | 196

Internet |

onlydepmu3 | Did the study meas. PIU?

vote | 0 1 . | Total

-----------+---------------------------------+----------

1 | 0 1 0 | 1

. | 127 60 8 | 195

-----------+---------------------------------+----------

Total | 127 61 8 | 196

Gaming | Internet

only | onlydepmu3vote

depmu3vote | 1 . | Total

-----------+----------------------+----------

1 | 0 1 | 1

. | 1 194 | 195

-----------+----------------------+----------

Total | 1 195 | 196

(195 missing values generated)

(195 missing values generated)

(0 real changes made)

(195 missing values generated)

(1 real change made, 1 to missing)

Gaming |

only | PGscaleYN

depmu4vote | 0 1 . | Total

-----------+---------------------------------+----------

. | 63 125 8 | 196

-----------+---------------------------------+----------

Total | 63 125 8 | 196

Internet |

onlydepmu4 | Did the study meas. PIU?

vote | 0 1 . | Total

-----------+---------------------------------+----------

1 | 0 1 0 | 1

. | 127 60 8 | 195

-----------+---------------------------------+----------

Total | 127 61 8 | 196

Gaming | Internet

only | onlydepmu4vote

depmu4vote | 1 . | Total

-----------+----------------------+----------

. | 1 195 | 196

-----------+----------------------+----------

Total | 1 195 | 196

(195 missing values generated)

(195 missing values generated)

(1 real change made, 1 to missing)

(195 missing values generated)

(0 real changes made)

Gaming |

only |

Ldepbi1vot | PGscaleYN

e | 0 1 . | Total

-----------+---------------------------------+----------

0 | 0 1 0 | 1

. | 63 124 8 | 195

-----------+---------------------------------+----------

Total | 63 125 8 | 196

Internet |

onlyLdepbi | Did the study meas. PIU?

1vote | 0 1 . | Total

-----------+---------------------------------+----------

. | 127 61 8 | 196

-----------+---------------------------------+----------

Total | 127 61 8 | 196

Gaming | Internet

only | onlyLdepbi

Ldepbi1vot | 1vote

e | . | Total

-----------+-----------+----------

0 | 1 | 1

. | 195 | 195

-----------+-----------+----------

Total | 196 | 196

(195 missing values generated)

(195 missing values generated)

(1 real change made, 1 to missing)

(195 missing values generated)

(0 real changes made)

Gaming |

only |

Ldepbi2vot | PGscaleYN

e | 0 1 . | Total

-----------+---------------------------------+----------

0 | 0 1 0 | 1

. | 63 124 8 | 195

-----------+---------------------------------+----------

Total | 63 125 8 | 196

Internet |

onlyLdepbi | Did the study meas. PIU?

2vote | 0 1 . | Total

-----------+---------------------------------+----------

. | 127 61 8 | 196

-----------+---------------------------------+----------

Total | 127 61 8 | 196

Gaming | Internet

only | onlyLdepbi

Ldepbi2vot | 2vote

e | . | Total

-----------+-----------+----------

0 | 1 | 1

. | 195 | 195

-----------+-----------+----------

Total | 196 | 196

(196 missing values generated)

(196 missing values generated)

(0 real changes made)

(196 missing values generated)

(0 real changes made)

Gaming |

only |

Ldepbi3vot | PGscaleYN

e | 0 1 . | Total

-----------+---------------------------------+----------

. | 63 125 8 | 196

-----------+---------------------------------+----------

Total | 63 125 8 | 196

Internet |

onlyLdepbi | Did the study meas. PIU?

3vote | 0 1 . | Total

-----------+---------------------------------+----------

. | 127 61 8 | 196

-----------+---------------------------------+----------

Total | 127 61 8 | 196

Gaming | Internet

only | onlyLdepbi

Ldepbi3vot | 3vote

e | . | Total

-----------+-----------+----------

. | 196 | 196

-----------+-----------+----------

Total | 196 | 196

(196 missing values generated)

(196 missing values generated)

(0 real changes made)

(196 missing values generated)

(0 real changes made)

Gaming |

only |

Ldepbi4vot | PGscaleYN

e | 0 1 . | Total

-----------+---------------------------------+----------

. | 63 125 8 | 196

-----------+---------------------------------+----------

Total | 63 125 8 | 196

Internet |

onlyLdepbi | Did the study meas. PIU?

4vote | 0 1 . | Total

-----------+---------------------------------+----------

. | 127 61 8 | 196

-----------+---------------------------------+----------

Total | 127 61 8 | 196

Gaming | Internet

only | onlyLdepbi

Ldepbi4vot | 4vote

e | . | Total

-----------+-----------+----------

. | 196 | 196

-----------+-----------+----------

Total | 196 | 196

(196 missing values generated)

(196 missing values generated)

(0 real changes made)

(196 missing values generated)

(0 real changes made)

Gaming |

only |

Ldepbi5vot | PGscaleYN

e | 0 1 . | Total

-----------+---------------------------------+----------

. | 63 125 8 | 196

-----------+---------------------------------+----------

Total | 63 125 8 | 196

Internet |

onlyLdepbi | Did the study meas. PIU?

5vote | 0 1 . | Total

-----------+---------------------------------+----------

. | 127 61 8 | 196

-----------+---------------------------------+----------

Total | 127 61 8 | 196

Gaming | Internet

only | onlyLdepbi

Ldepbi5vot | 5vote

e | . | Total

-----------+-----------+----------

. | 196 | 196

-----------+-----------+----------

Total | 196 | 196

(196 missing values generated)

(196 missing values generated)

(0 real changes made)

(196 missing values generated)

(0 real changes made)

Gaming |

only |

Ldepbi6vot | PGscaleYN

e | 0 1 . | Total

-----------+---------------------------------+----------

. | 63 125 8 | 196

-----------+---------------------------------+----------

Total | 63 125 8 | 196

Internet |

onlyLdepbi | Did the study meas. PIU?

6vote | 0 1 . | Total

-----------+---------------------------------+----------

. | 127 61 8 | 196

-----------+---------------------------------+----------

Total | 127 61 8 | 196

Gaming | Internet

only | onlyLdepbi

Ldepbi6vot | 6vote

e | . | Total

-----------+-----------+----------

. | 196 | 196

-----------+-----------+----------

Total | 196 | 196

(194 missing values generated)

(194 missing values generated)

(2 real changes made, 2 to missing)

(194 missing values generated)

(0 real changes made)

Gaming |

only |

Ldepmu1vot | PGscaleYN

e | 0 1 . | Total

-----------+---------------------------------+----------

1 | 0 2 0 | 2

. | 63 123 8 | 194

-----------+---------------------------------+----------

Total | 63 125 8 | 196

Internet |

onlyLdepmu | Did the study meas. PIU?

1vote | 0 1 . | Total

-----------+---------------------------------+----------

. | 127 61 8 | 196

-----------+---------------------------------+----------

Total | 127 61 8 | 196

Gaming | Internet

only | onlyLdepmu

Ldepmu1vot | 1vote

e | . | Total

-----------+-----------+----------

1 | 2 | 2

. | 194 | 194

-----------+-----------+----------

Total | 196 | 196

(195 missing values generated)

(195 missing values generated)

(1 real change made, 1 to missing)

(195 missing values generated)

(0 real changes made)

Gaming |

only |

Ldepmu2vot | PGscaleYN

e | 0 1 . | Total

-----------+---------------------------------+----------

1 | 0 1 0 | 1

. | 63 124 8 | 195

-----------+---------------------------------+----------

Total | 63 125 8 | 196

Internet |

onlyLdepmu | Did the study meas. PIU?

2vote | 0 1 . | Total

-----------+---------------------------------+----------

. | 127 61 8 | 196

-----------+---------------------------------+----------

Total | 127 61 8 | 196

Gaming | Internet

only | onlyLdepmu

Ldepmu2vot | 2vote

e | . | Total

-----------+-----------+----------

1 | 1 | 1

. | 195 | 195

-----------+-----------+----------

Total | 196 | 196

(195 missing values generated)

(195 missing values generated)

(1 real change made, 1 to missing)

(195 missing values generated)

(0 real changes made)

Gaming |

only |

Ldepmu3vot | PGscaleYN

e | 0 1 . | Total

-----------+---------------------------------+----------

1 | 0 1 0 | 1

. | 63 124 8 | 195

-----------+---------------------------------+----------

Total | 63 125 8 | 196

Internet |

onlyLdepmu | Did the study meas. PIU?

3vote | 0 1 . | Total

-----------+---------------------------------+----------

. | 127 61 8 | 196

-----------+---------------------------------+----------

Total | 127 61 8 | 196

Gaming | Internet

only | onlyLdepmu

Ldepmu3vot | 3vote

e | . | Total

-----------+-----------+----------

1 | 1 | 1

. | 195 | 195

-----------+-----------+----------

Total | 196 | 196

(195 missing values generated)

(195 missing values generated)

(1 real change made, 1 to missing)

(195 missing values generated)

(0 real changes made)

Gaming |

only |

Ldepmu4vot | PGscaleYN

e | 0 1 . | Total

-----------+---------------------------------+----------

1 | 0 1 0 | 1

. | 63 124 8 | 195

-----------+---------------------------------+----------

Total | 63 125 8 | 196

Internet |

onlyLdepmu | Did the study meas. PIU?

4vote | 0 1 . | Total

-----------+---------------------------------+----------

. | 127 61 8 | 196

-----------+---------------------------------+----------

Total | 127 61 8 | 196

Gaming | Internet

only | onlyLdepmu

Ldepmu4vot | 4vote

e | . | Total

-----------+-----------+----------

1 | 1 | 1

. | 195 | 195

-----------+-----------+----------

Total | 196 | 196

(196 missing values generated)

(196 missing values generated)

(0 real changes made)

(196 missing values generated)

(0 real changes made)

Gaming |

only |

Ldepmu5vot | PGscaleYN

e | 0 1 . | Total

-----------+---------------------------------+----------

. | 63 125 8 | 196

-----------+---------------------------------+----------

Total | 63 125 8 | 196

Internet |

onlyLdepmu | Did the study meas. PIU?

5vote | 0 1 . | Total

-----------+---------------------------------+----------

. | 127 61 8 | 196

-----------+---------------------------------+----------

Total | 127 61 8 | 196

Gaming | Internet

only | onlyLdepmu

Ldepmu5vot | 5vote

e | . | Total

-----------+-----------+----------

. | 196 | 196

-----------+-----------+----------

Total | 196 | 196

.

.

.

.

. tab anxbi1vote PGscaleYN, mi

C-S bivar |

anx result | PGscaleYN

in study | 0 1 . | Total

-----------+---------------------------------+----------

0 | 5 3 0 | 8

1 | 4 22 0 | 26

. | 54 100 8 | 162

-----------+---------------------------------+----------

Total | 63 125 8 | 196

. tab Banxbi1vote PGscaleYN, mi

Combo |

PG/IA | PGscaleYN

anxbi1vote | 0 1 . | Total

-----------+---------------------------------+----------

0 | 5 3 0 | 8

1 | 4 22 0 | 26

. | 54 100 8 | 162

-----------+---------------------------------+----------

Total | 63 125 8 | 196

.

.

.

. foreach var in ///

> anxbi1txt anxbi2txt anxbi3txt anxbi4txt anxbi5txt ///

> anxbi6txt anxbi7txt anxbi8txt anxbi9txt ///

> anxmu1txt anxmu2txt anxmu3txt anxmu4txt anxmu5txt anxmu6txt ///

> anxmu7txt anxmu8txt anxmu9txt ///

> Lanxbi1txt Lanxbi2txt Lanxbi3txt Lanxbi4txt Lanxbi5txt Lanxbi6txt ///

> Lanxmu1txt Lanxmu2txt Lanxmu3txt Lanxmu4txt Lanxmu5txt Lanxmu6txt ///

> Lanxmu7txt Lanxmu8txt Lanxmu9txt Lanxmu10txt Lanxmu11txt ///

> depbi1txt depbi2txt depbi3txt depbi4txt depbi5txt ///

> depbi6txt depbi7txt depbi8txt depbi9txt ///

> depmu1txt depmu2txt depmu3txt depmu4txt ///

> Ldepbi1txt Ldepbi2txt Ldepbi3txt Ldepbi4txt Ldepbi5txt Ldepbi6txt ///

> Ldepmu1txt Ldepmu2txt Ldepmu3txt Ldepmu4txt Ldepmu5txt {

2. cap drop B`var'

3. clonevar B`var'=`var'

4. lab var B`var' "Combo PG/IA `var'"

5. cap drop I`var'

6. clonevar I`var'=`var'

7. lab var I`var' "Internet only`var'"

8. replace I`var'="" if wasitPIU==0 | wasitPIU==.

9. cap drop G`var'

10. clonevar G`var'=`var'

11. lab var G`var' "Gaming only `var'"

12. replace G`var'="" if PGscaleYN==0 | PGscaleYN==.

13. tab G`var' PGscaleYN, mi

14. tab I`var' wasitPIU, mi

15. tab G`var' I`var', mi

16. }

(161 missing values generated)

(161 missing values generated)

(22 real changes made)

(161 missing values generated)

(10 real changes made)

| PGscaleYN

Gaming only anxbi1txt | 0 1 . | Total

----------------------+---------------------------------+----------

| 63 100 8 | 171

anxiety correlation | 0 1 0 | 1

anxiety items from.. | 0 1 0 | 1

anxiety subscale o.. | 0 1 0 | 1

BAS3 social anxiety | 0 1 0 | 1

DASS anxiety ANOVA | 0 1 0 | 1

DASS anxiety corre.. | 0 1 0 | 1

GAS-SCL90 anx | 0 1 0 | 1

HADS-1A anxiety AN.. | 0 1 0 | 1

M-W school anxiety | 0 1 0 | 1

SAS sub1 | 0 1 0 | 1

SCAS | 0 1 0 | 1

SCL-90 anxiety ANOVA | 0 1 0 | 1

STAI state | 0 1 0 | 1

School-1related an.. | 0 1 0 | 1

Svanx IGD vs non-ga.. | 0 1 0 | 1

adult males SCL an.. | 0 1 0 | 1

chi-square anxiety | 0 2 0 | 2

comorbid GAD | 0 1 0 | 1

correlation anxiety | 0 1 0 | 1

correlation with RC.. | 0 1 0 | 1

multipledim. anxiet.. | 0 1 0 | 1

panic/ anxiety males | 0 1 0 | 1

social anxiety VAT | 0 1 0 | 1

social anxiety over.. | 0 1 0 | 1

----------------------+---------------------------------+----------

Total | 63 125 8 | 196

Internet | Did the study meas. PIU?

onlyanxbi1txt | 0 1 . | Total

----------------------+---------------------------------+----------

| 127 48 8 | 183

BDA IGD vs HC | 0 1 0 | 1

Beck anxiety mean .. | 0 1 0 | 1

SAS IGA v contol | 0 4 0 | 4

SAS sub1 | 0 1 0 | 1

STAI-S mean iff | 0 1 0 | 1

correlation anxiety | 0 1 0 | 1

panic/ anxiety males | 0 1 0 | 1

social anxiety Tim.. | 0 1 0 | 1

social anxiety VAT | 0 1 0 | 1

social anxiety corr.. | 0 1 0 | 1

----------------------+---------------------------------+----------

Total | 127 61 8 | 196

| Internet onlyanxbi1txt

Gaming only anxbi1txt | BDA IGD.. Beck a.. SAS IGA.. SAS sub1 STAI-S .. correla.. panic/ .. | Total

----------------------+----------------------------------------------------------------------------------------+----------

| 162 1 1 4 0 1 0 0 | 171

anxiety correlation | 1 0 0 0 0 0 0 0 | 1

anxiety items from.. | 1 0 0 0 0 0 0 0 | 1

anxiety subscale o.. | 1 0 0 0 0 0 0 0 | 1

BAS3 social anxiety | 1 0 0 0 0 0 0 0 | 1

DASS anxiety ANOVA | 1 0 0 0 0 0 0 0 | 1

DASS anxiety corre.. | 1 0 0 0 0 0 0 0 | 1

GAS-SCL90 anx | 1 0 0 0 0 0 0 0 | 1

HADS-1A anxiety AN.. | 1 0 0 0 0 0 0 0 | 1

M-W school anxiety | 1 0 0 0 0 0 0 0 | 1

SAS sub1 | 0 0 0 0 1 0 0 0 | 1

SCAS | 1 0 0 0 0 0 0 0 | 1

SCL-90 anxiety ANOVA | 1 0 0 0 0 0 0 0 | 1

STAI state | 1 0 0 0 0 0 0 0 | 1

School-1related an.. | 1 0 0 0 0 0 0 0 | 1

Svanx IGD vs non-ga.. | 1 0 0 0 0 0 0 0 | 1

adult males SCL an.. | 1 0 0 0 0 0 0 0 | 1

chi-square anxiety | 2 0 0 0 0 0 0 0 | 2

comorbid GAD | 1 0 0 0 0 0 0 0 | 1

correlation anxiety | 0 0 0 0 0 0 1 0 | 1

correlation with RC.. | 1 0 0 0 0 0 0 0 | 1

multipledim. anxiet.. | 1 0 0 0 0 0 0 0 | 1

panic/ anxiety males | 0 0 0 0 0 0 0 1 | 1

social anxiety VAT | 0 0 0 0 0 0 0 0 | 1

social anxiety over.. | 1 0 0 0 0 0 0 0 | 1

----------------------+----------------------------------------------------------------------------------------+----------

Total | 183 1 1 4 1 1 1 1 | 196

| Internet onlyanxbi1txt

Gaming only anxbi1txt | social .. social .. social .. | Total

----------------------+---------------------------------+----------

| 1 0 1 | 171

anxiety correlation | 0 0 0 | 1

anxiety items from.. | 0 0 0 | 1

anxiety subscale o.. | 0 0 0 | 1

BAS3 social anxiety | 0 0 0 | 1

DASS anxiety ANOVA | 0 0 0 | 1

DASS anxiety corre.. | 0 0 0 | 1

GAS-SCL90 anx | 0 0 0 | 1

HADS-1A anxiety AN.. | 0 0 0 | 1

M-W school anxiety | 0 0 0 | 1

SAS sub1 | 0 0 0 | 1

SCAS | 0 0 0 | 1

SCL-90 anxiety ANOVA | 0 0 0 | 1

STAI state | 0 0 0 | 1

School-1related an.. | 0 0 0 | 1

Svanx IGD vs non-ga.. | 0 0 0 | 1

adult males SCL an.. | 0 0 0 | 1

chi-square anxiety | 0 0 0 | 2

comorbid GAD | 0 0 0 | 1

correlation anxiety | 0 0 0 | 1

correlation with RC.. | 0 0 0 | 1

multipledim. anxiet.. | 0 0 0 | 1

panic/ anxiety males | 0 0 0 | 1

social anxiety VAT | 0 1 0 | 1

social anxiety over.. | 0 0 0 | 1

----------------------+---------------------------------+----------

Total | 1 1 1 | 196

(177 missing values generated)

(177 missing values generated)

(14 real changes made)

(177 missing values generated)

(3 real changes made)

| PGscaleYN

Gaming only anxbi2txt | 0 1 . | Total

----------------------+---------------------------------+----------

| 63 109 8 | 180

anxiety chi-square | 0 1 0 | 1

anxiety disorder | 0 1 0 | 1

DASS anxiety corre.. | 0 1 0 | 1

GAS SCL90 phob | 0 1 0 | 1

Phobic anxiety sub.. | 0 1 0 | 1

SAS sub2 | 0 1 0 | 1

SCL-90 phobicANOVA | 0 1 0 | 1

STAI trait | 0 1 0 | 1

Svanx IGD vs reg ga.. | 0 1 0 | 1

adol males SCL anx.. | 0 1 0 | 1

comorbid social pho.. | 0 1 0 | 1

multipledem anxiet.. | 0 1 0 | 1

panic/ anxiety over.. | 0 1 0 | 1

social anxiety over.. | 0 1 0 | 1

social anxiety~GAS | 0 1 0 | 1

t-test vs. non-IGA .. | 0 1 0 | 1

----------------------+---------------------------------+----------

Total | 63 125 8 | 196

Internet | Did the study meas. PIU?

onlyanxbi2txt | 0 1 . | Total

----------------------+---------------------------------+----------

| 127 56 8 | 191

SAS sub2 | 0 1 0 | 1

STAI-T mean differe.. | 0 1 0 | 1

panic/ anxiety over.. | 0 1 0 | 1

social anxiety Tim.. | 0 1 0 | 1

social anxiety~GAS | 0 1 0 | 1

----------------------+---------------------------------+----------

Total | 127 61 8 | 196

| Internet onlyanxbi2txt

Gaming only anxbi2txt | SAS sub2 STAI-T .. panic/ .. social .. social .. | Total

----------------------+------------------------------------------------------------------+----------

| 178 0 1 0 1 0 | 180

anxiety chi-square | 1 0 0 0 0 0 | 1

anxiety disorder | 1 0 0 0 0 0 | 1

DASS anxiety corre.. | 1 0 0 0 0 0 | 1

GAS SCL90 phob | 1 0 0 0 0 0 | 1

Phobic anxiety sub.. | 1 0 0 0 0 0 | 1

SAS sub2 | 0 1 0 0 0 0 | 1

SCL-90 phobicANOVA | 1 0 0 0 0 0 | 1

STAI trait | 1 0 0 0 0 0 | 1

Svanx IGD vs reg ga.. | 1 0 0 0 0 0 | 1

adol males SCL anx.. | 1 0 0 0 0 0 | 1

comorbid social pho.. | 1 0 0 0 0 0 | 1

multipledem anxiet.. | 1 0 0 0 0 0 | 1

panic/ anxiety over.. | 0 0 0 1 0 0 | 1

social anxiety over.. | 1 0 0 0 0 0 | 1

social anxiety~GAS | 0 0 0 0 0 1 | 1

t-test vs. non-IGA .. | 1 0 0 0 0 0 | 1

----------------------+------------------------------------------------------------------+----------

Total | 191 1 1 1 1 1 | 196

(187 missing values generated)

(187 missing values generated)

(6 real changes made)

(187 missing values generated)

(2 real changes made)

| PGscaleYN

Gaming only anxbi3txt | 0 1 . | Total

----------------------+---------------------------------+----------

| 63 118 8 | 189

SAS sub3 | 0 1 0 | 1

Svanx IGD vs PIGD | 0 1 0 | 1

TSC anx impair | 0 1 0 | 1

comorbid panic | 0 1 0 | 1

overall SLC anxiety | 0 1 0 | 1

panic/ anxiety fema.. | 0 1 0 | 1

social anxiety fear.. | 0 1 0 | 1

----------------------+---------------------------------+----------

Total | 63 125 8 | 196

Internet | Did the study meas. PIU?

onlyanxbi3txt | 0 1 . | Total

----------------------+---------------------------------+----------

| 127 58 8 | 193

SAS sub3 | 0 1 0 | 1

SPS mean difference | 0 1 0 | 1

panic/ anxiety fema.. | 0 1 0 | 1

----------------------+---------------------------------+----------

Total | 127 61 8 | 196

| Internet onlyanxbi3txt

Gaming only anxbi3txt | SAS sub3 SPS mea.. panic/ .. | Total

----------------------+--------------------------------------------+----------

| 188 0 1 0 | 189

SAS sub3 | 0 1 0 0 | 1

Svanx IGD vs PIGD | 1 0 0 0 | 1

TSC anx impair | 1 0 0 0 | 1

comorbid panic | 1 0 0 0 | 1

overall SLC anxiety | 1 0 0 0 | 1

panic/ anxiety fema.. | 0 0 0 1 | 1

social anxiety fear.. | 1 0 0 0 | 1

----------------------+--------------------------------------------+----------

Total | 193 1 1 1 | 196

(191 missing values generated)

(191 missing values generated)

(4 real changes made)

(191 missing values generated)

(1 real change made)

| PGscaleYN

Gaming only anxbi4txt | 0 1 . | Total

----------------------+---------------------------------+----------

| 63 121 8 | 192

RCADS anxiety | 0 1 0 | 1

TSC anx sx | 0 1 0 | 1

adult males phobic .. | 0 1 0 | 1

social anxiety fear.. | 0 1 0 | 1

----------------------+---------------------------------+----------

Total | 63 125 8 | 196

Internet | Did the study meas. PIU?

onlyanxbi4txt | 0 1 . | Total

----------------------+---------------------------------+----------

| 127 60 8 | 195

RCADS anxiety | 0 1 0 | 1

----------------------+---------------------------------+----------

Total | 127 61 8 | 196

| Internet

| onlyanxbi4txt

Gaming only anxbi4txt | RCADS .. | Total

----------------------+----------------------+----------

| 192 0 | 192

RCADS anxiety | 0 1 | 1

TSC anx sx | 1 0 | 1

adult males phobic .. | 1 0 | 1

social anxiety fear.. | 1 0 | 1

----------------------+----------------------+----------

Total | 195 1 | 196

(193 missing values generated)

(193 missing values generated)

(2 real changes made)

(193 missing values generated)

(0 real changes made)

| PGscaleYN

Gaming only anxbi5txt | 0 1 . | Total

----------------------+---------------------------------+----------

| 63 122 8 | 193

RCADS panic | 0 1 0 | 1

adol males phobic .. | 0 1 0 | 1

social anxiety avoi.. | 0 1 0 | 1

----------------------+---------------------------------+----------

Total | 63 125 8 | 196

Internet | Did the study meas. PIU?

onlyanxbi5txt | 0 1 . | Total

----------------------+---------------------------------+----------

| 127 60 8 | 195

RCADS panic | 0 1 0 | 1

----------------------+---------------------------------+----------

Total | 127 61 8 | 196

| Internet

| onlyanxbi5txt

Gaming only anxbi5txt | RCADS p.. | Total

----------------------+----------------------+----------

| 193 0 | 193

RCADS panic | 0 1 | 1

adol males phobic .. | 1 0 | 1

social anxiety avoi.. | 1 0 | 1

----------------------+----------------------+----------

Total | 195 1 | 196

(194 missing values generated)

(194 missing values generated)

(1 real change made)

(194 missing values generated)

(0 real changes made)

| PGscaleYN

Gaming only anxbi6txt | 0 1 . | Total

----------------------+---------------------------------+----------

| 63 123 8 | 194

RCADS separation an.. | 0 1 0 | 1

social anxiety avoi.. | 0 1 0 | 1

----------------------+---------------------------------+----------

Total | 63 125 8 | 196

Internet | Did the study meas. PIU?

onlyanxbi6txt | 0 1 . | Total

----------------------+---------------------------------+----------

| 127 60 8 | 195

RCADS separation an.. | 0 1 0 | 1

----------------------+---------------------------------+----------

Total | 127 61 8 | 196

| Internet

| onlyanxbi6txt

Gaming only anxbi6txt | RCADS s.. | Total

----------------------+----------------------+----------

| 194 0 | 194

RCADS separation an.. | 0 1 | 1

social anxiety avoi.. | 1 0 | 1

----------------------+----------------------+----------

Total | 195 1 | 196

(195 missing values generated)

(195 missing values generated)

(0 real changes made)

(195 missing values generated)

(0 real changes made)

Gaming |

only | PGscaleYN

anxbi7txt | 0 1 . | Total

-----------+---------------------------------+----------

| 63 124 8 | 195

RCADS soc | 0 1 0 | 1

-----------+---------------------------------+----------

Total | 63 125 8 | 196

Internet |

onlyanxbi7 | Did the study meas. PIU?

txt | 0 1 . | Total

-----------+---------------------------------+----------

| 127 60 8 | 195

RCADS soc | 0 1 0 | 1

-----------+---------------------------------+----------

Total | 127 61 8 | 196

Gaming | Internet

only | onlyanxbi7txt

anxbi7txt | RCADS soc | Total

-----------+----------------------+----------

| 195 0 | 195

RCADS soc | 0 1 | 1

-----------+----------------------+----------

Total | 195 1 | 196

(195 missing values generated)

(195 missing values generated)

(0 real changes made)

(195 missing values generated)

(0 real changes made)

Gaming |

only | PGscaleYN

anxbi8txt | 0 1 . | Total

-----------+---------------------------------+----------

| 63 124 8 | 195

RCADS tot | 0 1 0 | 1

-----------+---------------------------------+----------

Total | 63 125 8 | 196

Internet |

onlyanxbi8 | Did the study meas. PIU?

txt | 0 1 . | Total

-----------+---------------------------------+----------

| 127 60 8 | 195

RCADS tot | 0 1 0 | 1

-----------+---------------------------------+----------

Total | 127 61 8 | 196

Gaming | Internet

only | onlyanxbi8txt

anxbi8txt | RCADS tot | Total

-----------+----------------------+----------

| 195 0 | 195

RCADS tot | 0 1 | 1

-----------+----------------------+----------

Total | 195 1 | 196

(195 missing values generated)

(195 missing values generated)

(0 real changes made)

(195 missing values generated)

(0 real changes made)

Gaming |

only | PGscaleYN

anxbi9txt | 0 1 . | Total

-----------+---------------------------------+----------

| 63 124 8 | 195

SAS tot | 0 1 0 | 1

-----------+---------------------------------+----------

Total | 63 125 8 | 196

Internet |

onlyanxbi9 | Did the study meas. PIU?

txt | 0 1 . | Total

-----------+---------------------------------+----------

| 127 60 8 | 195

SAS tot | 0 1 0 | 1

-----------+---------------------------------+----------

Total | 127 61 8 | 196

Gaming | Internet

only | onlyanxbi9txt

anxbi9txt | SAS tot | Total

-----------+----------------------+----------

| 195 0 | 195

SAS tot | 0 1 | 1

-----------+----------------------+----------

Total | 195 1 | 196

(184 missing values generated)

(184 missing values generated)

(5 real changes made)

(184 missing values generated)

(5 real changes made)

| PGscaleYN

Gaming only anxmu1txt | 0 1 . | Total

----------------------+---------------------------------+----------

| 63 118 8 | 189

"more anxietyious" | 0 1 0 | 1

DASS anxiety R1 | 0 1 0 | 1

HADS anxiety | 0 1 0 | 1

SCAS | 0 1 0 | 1

SCL-90 anxiety par.. | 0 1 0 | 1

SCL90 anx mediator .. | 0 1 0 | 1

multilevel regressi.. | 0 1 0 | 1

----------------------+---------------------------------+----------

Total | 63 125 8 | 196

Internet | Did the study meas. PIU?

onlyanxmu1txt | 0 1 . | Total

----------------------+---------------------------------+----------

| 127 54 8 | 189

"more anxietyious" | 0 1 0 | 1

HADS anxiety | 0 1 0 | 1

Model 2 anxiety | 0 1 0 | 1

SPIN | 0 1 0 | 1

Table4 BDA all | 0 1 0 | 1

Table4R1:STAI-State | 0 1 0 | 1

social anxiety pat.. | 0 1 0 | 1

----------------------+---------------------------------+----------

Total | 127 61 8 | 196

| Internet onlyanxmu1txt

Gaming only anxmu1txt | "more .. HADS a.. Model 2.. SPIN Table4 .. Table4R.. social .. | Total

----------------------+----------------------------------------------------------------------------------------+----------

| 184 0 0 1 1 1 1 1 | 189

"more anxietyious" | 0 1 0 0 0 0 0 0 | 1

DASS anxiety R1 | 1 0 0 0 0 0 0 0 | 1

HADS anxiety | 0 0 1 0 0 0 0 0 | 1

SCAS | 1 0 0 0 0 0 0 0 | 1

SCL-90 anxiety par.. | 1 0 0 0 0 0 0 0 | 1

SCL90 anx mediator .. | 1 0 0 0 0 0 0 0 | 1

multilevel regressi.. | 1 0 0 0 0 0 0 0 | 1

----------------------+----------------------------------------------------------------------------------------+----------

Total | 189 1 1 1 1 1 1 1 | 196

(189 missing values generated)

(189 missing values generated)

(3 real changes made)

(189 missing values generated)

(4 real changes made)

| PGscaleYN

Gaming only anxmu2txt | 0 1 . | Total

----------------------+---------------------------------+----------

| 63 122 8 | 193

DASS anxiety R2 | 0 1 0 | 1

SCL phob mediator t.. | 0 1 0 | 1

SCL-90 phobicpart c.. | 0 1 0 | 1

----------------------+---------------------------------+----------

Total | 63 125 8 | 196

Internet | Did the study meas. PIU?

onlyanxmu2txt | 0 1 . | Total

----------------------+---------------------------------+----------

| 127 57 8 | 192

Model 3 anxiety | 0 1 0 | 1

Table4 BDA OG | 0 1 0 | 1

Table4R1: STAI-Trait | 0 1 0 | 1

social anxiety path.. | 0 1 0 | 1

----------------------+---------------------------------+----------

Total | 127 61 8 | 196

| Internet onlyanxmu2txt

Gaming only anxmu2txt | Model 3.. Table4 .. Table4R.. social .. | Total

----------------------+-------------------------------------------------------+----------

| 189 1 1 1 1 | 193

DASS anxiety R2 | 1 0 0 0 0 | 1

SCL phob mediator t.. | 1 0 0 0 0 | 1

SCL-90 phobicpart c.. | 1 0 0 0 0 | 1

----------------------+-------------------------------------------------------+----------

Total | 192 1 1 1 1 | 196

(194 missing values generated)

(194 missing values generated)

(0 real changes made)

(194 missing values generated)

(2 real changes made)

| PGscaleYN

Gaming only anxmu3txt | 0 1 . | Total

----------------------+---------------------------------+----------

| 63 125 8 | 196

----------------------+---------------------------------+----------

Total | 63 125 8 | 196

Internet | Did the study meas. PIU?

onlyanxmu3txt | 0 1 . | Total

----------------------+---------------------------------+----------

| 127 59 8 | 194

Table4R1: SPS | 0 1 0 | 1

Table5 BDA all | 0 1 0 | 1

----------------------+---------------------------------+----------

Total | 127 61 8 | 196

| Internet onlyanxmu3txt

Gaming only anxmu3txt | Table4R.. Table5 .. | Total

----------------------+---------------------------------+----------

| 194 1 1 | 196

----------------------+---------------------------------+----------

Total | 194 1 1 | 196

(194 missing values generated)

(194 missing values generated)

(0 real changes made)

(194 missing values generated)

(2 real changes made)

Gaming only | PGscaleYN

anxmu4txt | 0 1 . | Total

----------------+---------------------------------+----------

| 63 125 8 | 196

----------------+---------------------------------+----------

Total | 63 125 8 | 196

Internet | Did the study meas. PIU?

onlyanxmu4txt | 0 1 . | Total

----------------+---------------------------------+----------

| 127 59 8 | 194

Table4R2:STAI-T | 0 1 0 | 1

Table5R4:BAI OG | 0 1 0 | 1

----------------+---------------------------------+----------

Total | 127 61 8 | 196

Gaming only | Internet onlyanxmu4txt

anxmu4txt | Table4R.. Table5R.. | Total

----------------+---------------------------------+----------

| 194 1 1 | 196

----------------+---------------------------------+----------

Total | 194 1 1 | 196

(195 missing values generated)

(195 missing values generated)

(0 real changes made)

(195 missing values generated)

(1 real change made)

Gaming only | PGscaleYN

anxmu5txt | 0 1 . | Total

-------------+---------------------------------+----------

| 63 125 8 | 196

-------------+---------------------------------+----------

Total | 63 125 8 | 196

Internet |

onlyanxmu5tx | Did the study meas. PIU?

t | 0 1 . | Total

-------------+---------------------------------+----------

| 127 60 8 | 195

Table4R2:SPS | 0 1 0 | 1

-------------+---------------------------------+----------

Total | 127 61 8 | 196

| Internet

Gaming only | onlyanxmu5txt

anxmu5txt | Table4R.. | Total

-------------+----------------------+----------

| 195 1 | 196

-------------+----------------------+----------

Total | 195 1 | 196

(195 missing values generated)

(195 missing values generated)

(0 real changes made)

(195 missing values generated)

(1 real change made)

Gaming only | PGscaleYN

anxmu6txt | 0 1 . | Total

----------------+---------------------------------+----------

| 63 125 8 | 196

----------------+---------------------------------+----------

Total | 63 125 8 | 196

Internet | Did the study meas. PIU?

onlyanxmu6txt | 0 1 . | Total

----------------+---------------------------------+----------

| 127 60 8 | 195

Table4R3:STAI-T | 0 1 0 | 1

----------------+---------------------------------+----------

Total | 127 61 8 | 196

| Internet

Gaming only | onlyanxmu6txt

anxmu6txt | Table4R.. | Total

----------------+----------------------+----------

| 195 1 | 196

----------------+----------------------+----------

Total | 195 1 | 196

(195 missing values generated)

(195 missing values generated)

(0 real changes made)

(195 missing values generated)

(1 real change made)

Gaming only | PGscaleYN

anxmu7txt | 0 1 . | Total

-------------+---------------------------------+----------

| 63 125 8 | 196

-------------+---------------------------------+----------

Total | 63 125 8 | 196

Internet |

onlyanxmu7tx | Did the study meas. PIU?

t | 0 1 . | Total

-------------+---------------------------------+----------

| 127 60 8 | 195

Table4R3:SPS | 0 1 0 | 1

-------------+---------------------------------+----------

Total | 127 61 8 | 196

| Internet

Gaming only | onlyanxmu7txt

anxmu7txt | Table4R.. | Total

-------------+----------------------+----------

| 195 1 | 196

-------------+----------------------+----------

Total | 195 1 | 196

(195 missing values generated)

(195 missing values generated)

(0 real changes made)

(195 missing values generated)

(1 real change made)

Gaming only | PGscaleYN

anxmu8txt | 0 1 . | Total

----------------+---------------------------------+----------

| 63 125 8 | 196

----------------+---------------------------------+----------

Total | 63 125 8 | 196

Internet | Did the study meas. PIU?

onlyanxmu8txt | 0 1 . | Total

----------------+---------------------------------+----------

| 127 60 8 | 195

Table5R2:STAI-T | 0 1 0 | 1

----------------+---------------------------------+----------

Total | 127 61 8 | 196

| Internet

Gaming only | onlyanxmu8txt

anxmu8txt | Table5R.. | Total

----------------+----------------------+----------

| 195 1 | 196

----------------+----------------------+----------

Total | 195 1 | 196

(195 missing values generated)

(195 missing values generated)

(0 real changes made)

(195 missing values generated)

(1 real change made)

Gaming only | PGscaleYN

anxmu9txt | 0 1 . | Total

-------------+---------------------------------+----------

| 63 125 8 | 196

-------------+---------------------------------+----------

Total | 63 125 8 | 196

Internet |

onlyanxmu9tx | Did the study meas. PIU?

t | 0 1 . | Total

-------------+---------------------------------+----------

| 127 60 8 | 195

Table5R2:SPS | 0 1 0 | 1

-------------+---------------------------------+----------

Total | 127 61 8 | 196

| Internet

Gaming only | onlyanxmu9txt

anxmu9txt | Table5R.. | Total

-------------+----------------------+----------

| 195 1 | 196

-------------+----------------------+----------

Total | 195 1 | 196

(135 real changes made)

(71 real changes made)

Gaming |

only | PGscaleYN

Lanxbi1txt | 0 1 . | Total

-----------+---------------------------------+----------

| 63 0 8 | 71

. | 0 125 0 | 125

-----------+---------------------------------+----------

Total | 63 125 8 | 196

Internet |

onlyLanxbi | Did the study meas. PIU?

1txt | 0 1 . | Total

-----------+---------------------------------+----------

| 127 0 8 | 135

. | 0 61 0 | 61

-----------+---------------------------------+----------

Total | 127 61 8 | 196

Gaming | Internet

only | onlyLanxbi1txt

Lanxbi1txt | . | Total

-----------+----------------------+----------

| 26 45 | 71

. | 109 16 | 125

-----------+----------------------+----------

Total | 135 61 | 196

(135 real changes made)

(71 real changes made)

Gaming |

only | PGscaleYN

Lanxbi2txt | 0 1 . | Total

-----------+---------------------------------+----------

| 63 0 8 | 71

. | 0 125 0 | 125

-----------+---------------------------------+----------

Total | 63 125 8 | 196

Internet |

onlyLanxbi | Did the study meas. PIU?

2txt | 0 1 . | Total

-----------+---------------------------------+----------

| 127 0 8 | 135

. | 0 61 0 | 61

-----------+---------------------------------+----------

Total | 127 61 8 | 196

Gaming | Internet

only | onlyLanxbi2txt

Lanxbi2txt | . | Total

-----------+----------------------+----------

| 26 45 | 71

. | 109 16 | 125

-----------+----------------------+----------

Total | 135 61 | 196

(135 real changes made)

(71 real changes made)

Gaming |

only | PGscaleYN

Lanxbi3txt | 0 1 . | Total

-----------+---------------------------------+----------

| 63 0 8 | 71

. | 0 125 0 | 125

-----------+---------------------------------+----------

Total | 63 125 8 | 196

Internet |

onlyLanxbi | Did the study meas. PIU?

3txt | 0 1 . | Total

-----------+---------------------------------+----------

| 127 0 8 | 135

. | 0 61 0 | 61

-----------+---------------------------------+----------

Total | 127 61 8 | 196

Gaming | Internet

only | onlyLanxbi3txt

Lanxbi3txt | . | Total

-----------+----------------------+----------

| 26 45 | 71

. | 109 16 | 125

-----------+----------------------+----------

Total | 135 61 | 196

(135 real changes made)

(71 real changes made)

Gaming |

only | PGscaleYN

Lanxbi4txt | 0 1 . | Total

-----------+---------------------------------+----------

| 63 0 8 | 71

. | 0 125 0 | 125

-----------+---------------------------------+----------

Total | 63 125 8 | 196

Internet |

onlyLanxbi | Did the study meas. PIU?

4txt | 0 1 . | Total

-----------+---------------------------------+----------

| 127 0 8 | 135

. | 0 61 0 | 61

-----------+---------------------------------+----------

Total | 127 61 8 | 196

Gaming | Internet

only | onlyLanxbi4txt

Lanxbi4txt | . | Total

-----------+----------------------+----------

| 26 45 | 71

. | 109 16 | 125

-----------+----------------------+----------

Total | 135 61 | 196

(135 real changes made)

(71 real changes made)

Gaming |

only | PGscaleYN

Lanxbi5txt | 0 1 . | Total

-----------+---------------------------------+----------

| 63 0 8 | 71

. | 0 125 0 | 125

-----------+---------------------------------+----------

Total | 63 125 8 | 196

Internet |

onlyLanxbi | Did the study meas. PIU?

5txt | 0 1 . | Total

-----------+---------------------------------+----------

| 127 0 8 | 135

. | 0 61 0 | 61

-----------+---------------------------------+----------

Total | 127 61 8 | 196

Gaming | Internet

only | onlyLanxbi5txt

Lanxbi5txt | . | Total

-----------+----------------------+----------

| 26 45 | 71

. | 109 16 | 125

-----------+----------------------+----------

Total | 135 61 | 196

(135 real changes made)

(71 real changes made)

Gaming |

only | PGscaleYN

Lanxbi6txt | 0 1 . | Total

-----------+---------------------------------+----------

| 63 0 8 | 71

. | 0 125 0 | 125

-----------+---------------------------------+----------

Total | 63 125 8 | 196

Internet |

onlyLanxbi | Did the study meas. PIU?

6txt | 0 1 . | Total

-----------+---------------------------------+----------

| 127 0 8 | 135

. | 0 61 0 | 61

-----------+---------------------------------+----------

Total | 127 61 8 | 196

Gaming | Internet

only | onlyLanxbi6txt

Lanxbi6txt | . | Total

-----------+----------------------+----------

| 26 45 | 71

. | 109 16 | 125

-----------+----------------------+----------

Total | 135 61 | 196

(194 missing values generated)

(194 missing values generated)

(2 real changes made)

(194 missing values generated)

(0 real changes made)

Gaming only | PGscaleYN

Lanxmu1txt | 0 1 . | Total

----------------------+---------------------------------+----------

| 63 123 8 | 194

W5 anx on W6 # of P.. | 0 1 0 | 1

anx Starts v Never T2 | 0 1 0 | 1

----------------------+---------------------------------+----------

Total | 63 125 8 | 196

Internet | Did the study meas. PIU?

onlyLanxmu1txt | 0 1 . | Total

----------------------+---------------------------------+----------

| 127 61 8 | 196

----------------------+---------------------------------+----------

Total | 127 61 8 | 196

| Internet

| onlyLanxmu

Gaming only | 1txt

Lanxmu1txt | | Total

----------------------+-----------+----------

| 194 | 194

W5 anx on W6 # of P.. | 1 | 1

anx Starts v Never T2 | 1 | 1

----------------------+-----------+----------

Total | 196 | 196

(194 missing values generated)

(194 missing values generated)

(2 real changes made)

(194 missing values generated)

(0 real changes made)

Gaming only | PGscaleYN

Lanxmu2txt | 0 1 . | Total

----------------------+---------------------------------+----------

| 63 123 8 | 194

W5 anx on W6presenc.. | 0 1 0 | 1

anx Starts vs Never.. | 0 1 0 | 1

----------------------+---------------------------------+----------

Total | 63 125 8 | 196

Internet | Did the study meas. PIU?

onlyLanxmu2txt | 0 1 . | Total

----------------------+---------------------------------+----------

| 127 61 8 | 196

----------------------+---------------------------------+----------

Total | 127 61 8 | 196

| Internet

| onlyLanxmu

Gaming only | 2txt

Lanxmu2txt | | Total

----------------------+-----------+----------

| 194 | 194

W5 anx on W6presenc.. | 1 | 1

anx Starts vs Never.. | 1 | 1

----------------------+-----------+----------

Total | 196 | 196

(194 missing values generated)

(194 missing values generated)

(2 real changes made)

(194 missing values generated)

(0 real changes made)

Gaming only | PGscaleYN

Lanxmu3txt | 0 1 . | Total

----------------------+---------------------------------+----------

| 63 123 8 | 194

SocPhob Starts v Ne.. | 0 1 0 | 1

W5 anx on W6 # of P.. | 0 1 0 | 1

----------------------+---------------------------------+----------

Total | 63 125 8 | 196

Internet | Did the study meas. PIU?

onlyLanxmu3txt | 0 1 . | Total

----------------------+---------------------------------+----------

| 127 61 8 | 196

----------------------+---------------------------------+----------

Total | 127 61 8 | 196

| Internet

| onlyLanxmu

Gaming only | 3txt

Lanxmu3txt | | Total

----------------------+-----------+----------

| 194 | 194

SocPhob Starts v Ne.. | 1 | 1

W5 anx on W6 # of P.. | 1 | 1

----------------------+-----------+----------

Total | 196 | 196

(194 missing values generated)

(194 missing values generated)

(2 real changes made)

(194 missing values generated)

(0 real changes made)

Gaming only | PGscaleYN

Lanxmu4txt | 0 1 . | Total

----------------------+---------------------------------+----------

| 63 123 8 | 194

SocPhob Starts vs N.. | 0 1 0 | 1

W5 anx on W6 presen.. | 0 1 0 | 1

----------------------+---------------------------------+----------

Total | 63 125 8 | 196

Internet | Did the study meas. PIU?

onlyLanxmu4txt | 0 1 . | Total

----------------------+---------------------------------+----------

| 127 61 8 | 196

----------------------+---------------------------------+----------

Total | 127 61 8 | 196

| Internet

| onlyLanxmu

Gaming only | 4txt

Lanxmu4txt | | Total

----------------------+-----------+----------

| 194 | 194

SocPhob Starts vs N.. | 1 | 1

W5 anx on W6 presen.. | 1 | 1

----------------------+-----------+----------

Total | 196 | 196

(195 missing values generated)

(195 missing values generated)

(1 real change made)

(195 missing values generated)

(0 real changes made)

Gaming only | PGscaleYN

Lanxmu5txt | 0 1 . | Total

-------------------+---------------------------------+----------

| 63 124 8 | 195

anx StopsvStays T3 | 0 1 0 | 1

-------------------+---------------------------------+----------

Total | 63 125 8 | 196

Internet | Did the study meas. PIU?

onlyLanxmu5txt | 0 1 . | Total

-------------------+---------------------------------+----------

| 127 61 8 | 196

-------------------+---------------------------------+----------

Total | 127 61 8 | 196

| Internet

| onlyLanxmu

Gaming only | 5txt

Lanxmu5txt | | Total

-------------------+-----------+----------

| 195 | 195

anx StopsvStays T3 | 1 | 1

-------------------+-----------+----------

Total | 196 | 196

(195 missing values generated)

(195 missing values generated)

(1 real change made)

(195 missing values generated)

(0 real changes made)

Gaming only | PGscaleYN

Lanxmu6txt | 0 1 . | Total

----------------------+---------------------------------+----------

| 63 124 8 | 195

SocPhob stopsvStays.. | 0 1 0 | 1

----------------------+---------------------------------+----------

Total | 63 125 8 | 196

Internet | Did the study meas. PIU?

onlyLanxmu6txt | 0 1 . | Total

----------------------+---------------------------------+----------

| 127 61 8 | 196

----------------------+---------------------------------+----------

Total | 127 61 8 | 196

| Internet

| onlyLanxmu

Gaming only | 6txt

Lanxmu6txt | | Total

----------------------+-----------+----------

| 195 | 195

SocPhob stopsvStays.. | 1 | 1

----------------------+-----------+----------

Total | 196 | 196

(195 missing values generated)

(195 missing values generated)

(1 real change made)

(195 missing values generated)

(0 real changes made)

Gaming only | PGscaleYN

Lanxmu7txt | 0 1 . | Total

----------------------+---------------------------------+----------

| 63 124 8 | 195

SocPhob Stopsvs.Sta.. | 0 1 0 | 1

----------------------+---------------------------------+----------

Total | 63 125 8 | 196

Internet | Did the study meas. PIU?

onlyLanxmu7txt | 0 1 . | Total

----------------------+---------------------------------+----------

| 127 61 8 | 196

----------------------+---------------------------------+----------

Total | 127 61 8 | 196

| Internet

| onlyLanxmu

Gaming only | 7txt

Lanxmu7txt | | Total

----------------------+-----------+----------

| 195 | 195

SocPhob Stopsvs.Sta.. | 1 | 1

----------------------+-----------+----------

Total | 196 | 196

(195 missing values generated)

(195 missing values generated)

(1 real change made)

(195 missing values generated)

(0 real changes made)

Gaming only | PGscaleYN

Lanxmu8txt | 0 1 . | Total

----------------------+---------------------------------+----------

| 63 124 8 | 195

Change in PG to T3 .. | 0 1 0 | 1

----------------------+---------------------------------+----------

Total | 63 125 8 | 196

Internet | Did the study meas. PIU?

onlyLanxmu8txt | 0 1 . | Total

----------------------+---------------------------------+----------

| 127 61 8 | 196

----------------------+---------------------------------+----------

Total | 127 61 8 | 196

| Internet

| onlyLanxmu

Gaming only | 8txt

Lanxmu8txt | | Total

----------------------+-----------+----------

| 195 | 195

Change in PG to T3 .. | 1 | 1

----------------------+-----------+----------

Total | 196 | 196

(195 missing values generated)

(195 missing values generated)

(1 real change made)

(195 missing values generated)

(0 real changes made)

Gaming only | PGscaleYN

Lanxmu9txt | 0 1 . | Total

----------------------+---------------------------------+----------

| 63 124 8 | 195

Change in PG to T3 .. | 0 1 0 | 1

----------------------+---------------------------------+----------

Total | 63 125 8 | 196

Internet | Did the study meas. PIU?

onlyLanxmu9txt | 0 1 . | Total

----------------------+---------------------------------+----------

| 127 61 8 | 196

----------------------+---------------------------------+----------

Total | 127 61 8 | 196

| Internet

| onlyLanxmu

Gaming only | 9txt

Lanxmu9txt | | Total

----------------------+-----------+----------

| 195 | 195

Change in PG to T3 .. | 1 | 1

----------------------+-----------+----------

Total | 196 | 196

(195 missing values generated)

(195 missing values generated)

(1 real change made)

(195 missing values generated)

(0 real changes made)

Gaming only | PGscaleYN

Lanxmu10txt | 0 1 . | Total

----------------------+---------------------------------+----------

| 63 124 8 | 195

PG intercept to T3anx | 0 1 0 | 1

----------------------+---------------------------------+----------

Total | 63 125 8 | 196

Internet | Did the study meas. PIU?

onlyLanxmu10txt | 0 1 . | Total

----------------------+---------------------------------+----------

| 127 61 8 | 196

----------------------+---------------------------------+----------

Total | 127 61 8 | 196

| Internet

| onlyLanxmu

Gaming only | 10txt

Lanxmu10txt | | Total

----------------------+-----------+----------

| 195 | 195

PG intercept to T3anx | 1 | 1

----------------------+-----------+----------

Total | 196 | 196

(195 missing values generated)

(195 missing values generated)

(1 real change made)

(195 missing values generated)

(0 real changes made)

Gaming only | PGscaleYN

Lanxmu11txt | 0 1 . | Total

----------------------+---------------------------------+----------

| 63 124 8 | 195

PG intercept to T3 .. | 0 1 0 | 1

----------------------+---------------------------------+----------

Total | 63 125 8 | 196

Internet | Did the study meas. PIU?

onlyLanxmu11txt | 0 1 . | Total

----------------------+---------------------------------+----------

| 127 61 8 | 196

----------------------+---------------------------------+----------

Total | 127 61 8 | 196

| Internet

| onlyLanxmu

Gaming only | 11txt

Lanxmu11txt | | Total

----------------------+-----------+----------

| 195 | 195

PG intercept to T3 .. | 1 | 1

----------------------+-----------+----------

Total | 196 | 196

(153 missing values generated)

(153 missing values generated)

(23 real changes made)

(153 missing values generated)

(16 real changes made)

| PGscaleYN

Gaming only depbi1txt | 0 1 . | Total

----------------------+---------------------------------+----------

| 63 98 8 | 169

"depression" vs no .. | 0 1 0 | 1

Asian Adol depressi.. | 0 1 0 | 1

BDI VAT | 0 1 0 | 1

DASS depression ANO.. | 0 1 0 | 1

DASS depression cor.. | 0 1 0 | 1

DRS-A | 0 1 0 | 1

GAS-SCL90 dep | 0 1 0 | 1

HADS-1D depression .. | 0 1 0 | 1

RADS & impairment fac | 0 1 0 | 1

SCL-90 depression A.. | 0 1 0 | 1

Svdep IGD vs non-ga.. | 0 1 0 | 1

Table 6 boys chi-sq.. | 0 1 0 | 1

Time1 depression Ti.. | 0 1 0 | 1

YSR depression sub | 0 1 0 | 1

adult males SCL dep.. | 0 1 0 | 1

chi-square depression | 0 3 0 | 3

comorbid depression | 0 1 0 | 1

correlation depress.. | 0 1 0 | 1

depression & VAT co.. | 0 1 0 | 1

depression RCADS sub | 0 1 0 | 1

depression correlat.. | 0 1 0 | 1

depression sub BSI | 0 1 0 | 1

depression sx | 0 1 0 | 1

latent class PG chi.. | 0 1 0 | 1

suicidal thoughts | 0 1 0 | 1

----------------------+---------------------------------+----------

Total | 63 125 8 | 196

Internet | Did the study meas. PIU?

onlydepbi1txt | 0 1 . | Total

----------------------+---------------------------------+----------

| 127 41 8 | 176

"depression" vs no .. | 0 1 0 | 1

BDI ANOVA | 0 1 0 | 1

BDI IGD vs HC | 0 2 0 | 2

BDI IGD vs. HC | 0 1 0 | 1

BDI vs. controls | 0 1 0 | 1

Beck mean difference | 0 1 0 | 1

SDS IGA vs control | 0 4 0 | 4

correlation depress.. | 0 1 0 | 1

depression & VAT co.. | 0 1 0 | 1

depression ANOVA | 0 2 0 | 2

depression RCADS sub | 0 1 0 | 1

depression sx | 0 1 0 | 1

depressionr Time1~P.. | 0 1 0 | 1

single question dep.. | 0 1 0 | 1

t-test with CIUS | 0 1 0 | 1

----------------------+---------------------------------+----------

Total | 127 61 8 | 196

| Internet onlydepbi1txt

Gaming only depbi1txt | "depres.. BDI ANOVA BDI IGD.. BDI IGD.. BDI vs... Beck me.. SDS IGA.. | Total

----------------------+----------------------------------------------------------------------------------------+----------

| 154 0 1 2 1 1 1 4 | 169

"depression" vs no .. | 0 1 0 0 0 0 0 0 | 1

Asian Adol depressi.. | 1 0 0 0 0 0 0 0 | 1

BDI VAT | 1 0 0 0 0 0 0 0 | 1

DASS depression ANO.. | 1 0 0 0 0 0 0 0 | 1

DASS depression cor.. | 1 0 0 0 0 0 0 0 | 1

DRS-A | 1 0 0 0 0 0 0 0 | 1

GAS-SCL90 dep | 1 0 0 0 0 0 0 0 | 1

HADS-1D depression .. | 1 0 0 0 0 0 0 0 | 1

RADS & impairment fac | 1 0 0 0 0 0 0 0 | 1

SCL-90 depression A.. | 1 0 0 0 0 0 0 0 | 1

Svdep IGD vs non-ga.. | 1 0 0 0 0 0 0 0 | 1

Table 6 boys chi-sq.. | 1 0 0 0 0 0 0 0 | 1

Time1 depression Ti.. | 1 0 0 0 0 0 0 0 | 1

YSR depression sub | 1 0 0 0 0 0 0 0 | 1

adult males SCL dep.. | 1 0 0 0 0 0 0 0 | 1

chi-square depression | 3 0 0 0 0 0 0 0 | 3

comorbid depression | 1 0 0 0 0 0 0 0 | 1

correlation depress.. | 0 0 0 0 0 0 0 0 | 1

depression & VAT co.. | 0 0 0 0 0 0 0 0 | 1

depression RCADS sub | 0 0 0 0 0 0 0 0 | 1

depression correlat.. | 1 0 0 0 0 0 0 0 | 1

depression sub BSI | 1 0 0 0 0 0 0 0 | 1

depression sx | 0 0 0 0 0 0 0 0 | 1

latent class PG chi.. | 1 0 0 0 0 0 0 0 | 1

suicidal thoughts | 1 0 0 0 0 0 0 0 | 1

----------------------+----------------------------------------------------------------------------------------+----------

Total | 176 1 1 2 1 1 1 4 | 196

| Internet onlydepbi1txt

Gaming only depbi1txt | correla.. depress.. depress.. depress.. depress.. depress.. single .. t-test .. | Total

----------------------+----------------------------------------------------------------------------------------+----------

| 0 0 2 0 0 1 1 1 | 169

"depression" vs no .. | 0 0 0 0 0 0 0 0 | 1

Asian Adol depressi.. | 0 0 0 0 0 0 0 0 | 1

BDI VAT | 0 0 0 0 0 0 0 0 | 1

DASS depression ANO.. | 0 0 0 0 0 0 0 0 | 1

DASS depression cor.. | 0 0 0 0 0 0 0 0 | 1

DRS-A | 0 0 0 0 0 0 0 0 | 1

GAS-SCL90 dep | 0 0 0 0 0 0 0 0 | 1

HADS-1D depression .. | 0 0 0 0 0 0 0 0 | 1

RADS & impairment fac | 0 0 0 0 0 0 0 0 | 1

SCL-90 depression A.. | 0 0 0 0 0 0 0 0 | 1

Svdep IGD vs non-ga.. | 0 0 0 0 0 0 0 0 | 1

Table 6 boys chi-sq.. | 0 0 0 0 0 0 0 0 | 1

Time1 depression Ti.. | 0 0 0 0 0 0 0 0 | 1

YSR depression sub | 0 0 0 0 0 0 0 0 | 1

adult males SCL dep.. | 0 0 0 0 0 0 0 0 | 1

chi-square depression | 0 0 0 0 0 0 0 0 | 3

comorbid depression | 0 0 0 0 0 0 0 0 | 1

correlation depress.. | 1 0 0 0 0 0 0 0 | 1

depression & VAT co.. | 0 1 0 0 0 0 0 0 | 1

depression RCADS sub | 0 0 0 1 0 0 0 0 | 1

depression correlat.. | 0 0 0 0 0 0 0 0 | 1

depression sub BSI | 0 0 0 0 0 0 0 0 | 1

depression sx | 0 0 0 0 1 0 0 0 | 1

latent class PG chi.. | 0 0 0 0 0 0 0 0 | 1

suicidal thoughts | 0 0 0 0 0 0 0 0 | 1

----------------------+----------------------------------------------------------------------------------------+----------

Total | 1 1 2 1 1 1 1 1 | 196

(180 missing values generated)

(180 missing values generated)

(11 real changes made)

(180 missing values generated)

(4 real changes made)

| PGscaleYN

Gaming only depbi2txt | 0 1 . | Total

----------------------+---------------------------------+----------

| 63 113 8 | 184

BDI GAS | 0 1 0 | 1

DASS depression cor.. | 0 1 0 | 1

Hopeless & imp fac | 0 1 0 | 1

Svdep IGD vs reg ga.. | 0 1 0 | 1

Table 6 girls chi-s.. | 0 1 0 | 1

Time2 depression Ti.. | 0 1 0 | 1

adol males SCL depr.. | 0 1 0 | 1

chi-square depression | 0 1 0 | 1

correlation depress.. | 0 1 0 | 1

depression and GAS .. | 0 1 0 | 1

depression females | 0 1 0 | 1

depressive dis | 0 1 0 | 1

----------------------+---------------------------------+----------

Total | 63 125 8 | 196

Internet | Did the study meas. PIU?

onlydepbi2txt | 0 1 . | Total

----------------------+---------------------------------+----------

| 127 56 8 | 191

correlation with CIUS | 0 1 0 | 1

depression and GAS .. | 0 1 0 | 1

depression females | 0 1 0 | 1

depressionr Time2~P.. | 0 1 0 | 1

suicide PSS ANOVA | 0 1 0 | 1

----------------------+---------------------------------+----------

Total | 127 61 8 | 196

| Internet onlydepbi2txt

Gaming only depbi2txt | correla.. depress.. depress.. depress.. suicide.. | Total

----------------------+------------------------------------------------------------------+----------

| 181 1 0 0 1 1 | 184

BDI GAS | 1 0 0 0 0 0 | 1

DASS depression cor.. | 1 0 0 0 0 0 | 1

Hopeless & imp fac | 1 0 0 0 0 0 | 1

Svdep IGD vs reg ga.. | 1 0 0 0 0 0 | 1

Table 6 girls chi-s.. | 1 0 0 0 0 0 | 1

Time2 depression Ti.. | 1 0 0 0 0 0 | 1

adol males SCL depr.. | 1 0 0 0 0 0 | 1

chi-square depression | 1 0 0 0 0 0 | 1

correlation depress.. | 1 0 0 0 0 0 | 1

depression and GAS .. | 0 0 1 0 0 0 | 1

depression females | 0 0 0 1 0 0 | 1

depressive dis | 1 0 0 0 0 0 | 1

----------------------+------------------------------------------------------------------+----------

Total | 191 1 1 1 1 1 | 196

(192 missing values generated)

(192 missing values generated)

(3 real changes made)

(192 missing values generated)

(0 real changes made)

| PGscaleYN

Gaming only depbi3txt | 0 1 . | Total

----------------------+---------------------------------+----------

| 63 121 8 | 192

Svdep IGD vs PIGD | 0 1 0 | 1

depression males | 0 1 0 | 1

overall SCL depress.. | 0 1 0 | 1

suicide & impairmen.. | 0 1 0 | 1

----------------------+---------------------------------+----------

Total | 63 125 8 | 196

Internet | Did the study meas. PIU?

onlydepbi3txt | 0 1 . | Total

----------------------+---------------------------------+----------

| 127 60 8 | 195

depression males | 0 1 0 | 1

----------------------+---------------------------------+----------

Total | 127 61 8 | 196

| Internet

| onlydepbi3txt

Gaming only depbi3txt | depress.. | Total

----------------------+----------------------+----------

| 192 0 | 192

Svdep IGD vs PIGD | 1 0 | 1

depression males | 0 1 | 1

overall SCL depress.. | 1 0 | 1

suicide & impairmen.. | 1 0 | 1

----------------------+----------------------+----------

Total | 195 1 | 196

(195 missing values generated)

(195 missing values generated)

(1 real change made)

(195 missing values generated)

(0 real changes made)

Gaming only | PGscaleYN

depbi4txt | 0 1 . | Total

----------------+---------------------------------+----------

| 63 124 8 | 195

RADS & symp fac | 0 1 0 | 1

----------------+---------------------------------+----------

Total | 63 125 8 | 196

Internet | Did the study meas. PIU?

onlydepbi4txt | 0 1 . | Total

----------------+---------------------------------+----------

| 127 61 8 | 196

----------------+---------------------------------+----------

Total | 127 61 8 | 196

| Internet

| onlydepbi4

Gaming only | txt

depbi4txt | | Total

----------------+-----------+----------

| 195 | 195

RADS & symp fac | 1 | 1

----------------+-----------+----------

Total | 196 | 196

(195 missing values generated)

(195 missing values generated)

(1 real change made)

(195 missing values generated)

(0 real changes made)

Gaming only | PGscaleYN

depbi5txt | 0 1 . | Total

--------------------+---------------------------------+----------

| 63 124 8 | 195

Hopeless & symp fac | 0 1 0 | 1

--------------------+---------------------------------+----------

Total | 63 125 8 | 196

Internet | Did the study meas. PIU?

onlydepbi5txt | 0 1 . | Total

--------------------+---------------------------------+----------

| 127 61 8 | 196

--------------------+---------------------------------+----------

Total | 127 61 8 | 196

| Internet

| onlydepbi5

Gaming only | txt

depbi5txt | | Total

--------------------+-----------+----------

| 195 | 195

Hopeless & symp fac | 1 | 1

--------------------+-----------+----------

Total | 196 | 196

(195 missing values generated)

(195 missing values generated)

(1 real change made)

(195 missing values generated)

(0 real changes made)

Gaming only | PGscaleYN

depbi6txt | 0 1 . | Total

-------------------+---------------------------------+----------

| 63 124 8 | 195

suicide & symp fac | 0 1 0 | 1

-------------------+---------------------------------+----------

Total | 63 125 8 | 196

Internet | Did the study meas. PIU?

onlydepbi6txt | 0 1 . | Total

-------------------+---------------------------------+----------

| 127 61 8 | 196

-------------------+---------------------------------+----------

Total | 127 61 8 | 196

| Internet

| onlydepbi6

Gaming only | txt

depbi6txt | | Total

-------------------+-----------+----------

| 195 | 195

suicide & symp fac | 1 | 1

-------------------+-----------+----------

Total | 196 | 196

(195 missing values generated)

(195 missing values generated)

(1 real change made)

(195 missing values generated)

(0 real changes made)

Gaming only | PGscaleYN

depbi7txt | 0 1 . | Total

------------+---------------------------------+----------

| 63 124 8 | 195

TSC dep imp | 0 1 0 | 1

------------+---------------------------------+----------

Total | 63 125 8 | 196

Internet |

onlydepbi7t | Did the study meas. PIU?

xt | 0 1 . | Total

------------+---------------------------------+----------

| 127 61 8 | 196

------------+---------------------------------+----------

Total | 127 61 8 | 196

| Internet

| onlydepbi7

Gaming only | txt

depbi7txt | | Total

------------+-----------+----------

| 195 | 195

TSC dep imp | 1 | 1

------------+-----------+----------

Total | 196 | 196

(195 missing values generated)

(195 missing values generated)

(1 real change made)

(195 missing values generated)

(0 real changes made)

Gaming |

only | PGscaleYN

depbi8txt | 0 1 . | Total

-----------+---------------------------------+----------

| 63 124 8 | 195

TSC dep sx | 0 1 0 | 1

-----------+---------------------------------+----------

Total | 63 125 8 | 196

Internet |

onlydepbi8 | Did the study meas. PIU?

txt | 0 1 . | Total

-----------+---------------------------------+----------

| 127 61 8 | 196

-----------+---------------------------------+----------

Total | 127 61 8 | 196

| Internet

Gaming | onlydepbi8

only | txt

depbi8txt | | Total

-----------+-----------+----------

| 195 | 195

TSC dep sx | 1 | 1

-----------+-----------+----------

Total | 196 | 196

(135 real changes made)

(71 real changes made)

Gaming |

only | PGscaleYN

depbi9txt | 0 1 . | Total

-----------+---------------------------------+----------

| 63 0 8 | 71

. | 0 125 0 | 125

-----------+---------------------------------+----------

Total | 63 125 8 | 196

Internet |

onlydepbi9 | Did the study meas. PIU?

txt | 0 1 . | Total

-----------+---------------------------------+----------

| 127 0 8 | 135

. | 0 61 0 | 61

-----------+---------------------------------+----------

Total | 127 61 8 | 196

Gaming | Internet

only | onlydepbi9txt

depbi9txt | . | Total

-----------+----------------------+----------

| 26 45 | 71

. | 109 16 | 125

-----------+----------------------+----------

Total | 135 61 | 196

(178 missing values generated)

(178 missing values generated)

(12 real changes made)

(178 missing values generated)

(5 real changes made)

| PGscaleYN

Gaming only depmu1txt | 0 1 . | Total

----------------------+---------------------------------+----------

| 63 112 8 | 183

CESD10 R1 | 0 1 0 | 1

DASS depression R1 | 0 1 0 | 1

DRS-A | 0 1 0 | 1

HADS depression | 0 1 0 | 1

SCL-90 depression p.. | 0 1 0 | 1

SCL90 dep mediator .. | 0 1 0 | 1

Table 7 multivariat.. | 0 1 0 | 1

direct path | 0 1 0 | 1

multilevel regressi.. | 0 1 0 | 1

multiple linear reg.. | 0 1 0 | 1

multiple regression.. | 0 1 0 | 1

path model CESD | 0 1 0 | 1

w/d/dep scale of YS.. | 0 1 0 | 1

----------------------+---------------------------------+----------

Total | 63 125 8 | 196

Internet | Did the study meas. PIU?

onlydepmu1txt | 0 1 . | Total

----------------------+---------------------------------+----------

| 127 55 8 | 190

depression regress.. | 0 1 0 | 1

HADS depression | 0 1 0 | 1

Model 2 depression | 0 1 0 | 1

Table 4 BDI all | 0 1 0 | 1

depression multiple.. | 0 1 0 | 1

multiple regression | 0 1 0 | 1

----------------------+---------------------------------+----------

Total | 127 61 8 | 196

| Internet onlydepmu1txt

Gaming only depmu1txt | depres.. HADS de.. Model 2.. Table 4.. depress.. multipl.. | Total

----------------------+-----------------------------------------------------------------------------+----------

| 178 1 0 1 1 1 1 | 183

CESD10 R1 | 1 0 0 0 0 0 0 | 1

DASS depression R1 | 1 0 0 0 0 0 0 | 1

DRS-A | 1 0 0 0 0 0 0 | 1

HADS depression | 0 0 1 0 0 0 0 | 1

SCL-90 depression p.. | 1 0 0 0 0 0 0 | 1

SCL90 dep mediator .. | 1 0 0 0 0 0 0 | 1

Table 7 multivariat.. | 1 0 0 0 0 0 0 | 1

direct path | 1 0 0 0 0 0 0 | 1

multilevel regressi.. | 1 0 0 0 0 0 0 | 1

multiple linear reg.. | 1 0 0 0 0 0 0 | 1

multiple regression.. | 1 0 0 0 0 0 0 | 1

path model CESD | 1 0 0 0 0 0 0 | 1

w/d/dep scale of YS.. | 1 0 0 0 0 0 0 | 1

----------------------+-----------------------------------------------------------------------------+----------

Total | 190 1 1 1 1 1 1 | 196

(190 missing values generated)

(190 missing values generated)

(3 real changes made)

(190 missing values generated)

(3 real changes made)

| PGscaleYN

Gaming only depmu2txt | 0 1 . | Total

----------------------+---------------------------------+----------

| 63 122 8 | 193

CESD10 males R2 | 0 1 0 | 1

DASS depression R2 | 0 1 0 | 1

indirect path | 0 1 0 | 1

----------------------+---------------------------------+----------

Total | 63 125 8 | 196

Internet | Did the study meas. PIU?

onlydepmu2txt | 0 1 . | Total

----------------------+---------------------------------+----------

| 127 58 8 | 193

Model 3 depression | 0 1 0 | 1

Table4 BDI OG | 0 1 0 | 1

suicide multiple re.. | 0 1 0 | 1

----------------------+---------------------------------+----------

Total | 127 61 8 | 196

| Internet onlydepmu2txt

Gaming only depmu2txt | Model 3.. Table4 .. suicide.. | Total

----------------------+--------------------------------------------+----------

| 190 1 1 1 | 193

CESD10 males R2 | 1 0 0 0 | 1

DASS depression R2 | 1 0 0 0 | 1

indirect path | 1 0 0 0 | 1

----------------------+--------------------------------------------+----------

Total | 193 1 1 1 | 196

(194 missing values generated)

(194 missing values generated)

(1 real change made)

(194 missing values generated)

(1 real change made)

Gaming only | PGscaleYN

depmu3txt | 0 1 . | Total

---------------+---------------------------------+----------

| 63 124 8 | 195

CESD10 FR3 | 0 1 0 | 1

---------------+---------------------------------+----------

Total | 63 125 8 | 196

Internet | Did the study meas. PIU?

onlydepmu3txt | 0 1 . | Total

---------------+---------------------------------+----------

| 127 60 8 | 195

Table5 BDI all | 0 1 0 | 1

---------------+---------------------------------+----------

Total | 127 61 8 | 196

| Internet

Gaming only | onlydepmu3txt

depmu3txt | Table5 .. | Total

---------------+----------------------+----------

| 194 1 | 195

CESD10 FR3 | 1 0 | 1

---------------+----------------------+----------

Total | 195 1 | 196

(195 missing values generated)

(195 missing values generated)

(0 real changes made)

(195 missing values generated)

(1 real change made)

Gaming only | PGscaleYN

depmu4txt | 0 1 . | Total

----------------+---------------------------------+----------

| 63 125 8 | 196

----------------+---------------------------------+----------

Total | 63 125 8 | 196

Internet | Did the study meas. PIU?

onlydepmu4txt | 0 1 . | Total

----------------+---------------------------------+----------

| 127 60 8 | 195

Table5R4:BAI OG | 0 1 0 | 1

----------------+---------------------------------+----------

Total | 127 61 8 | 196

| Internet

Gaming only | onlydepmu4txt

depmu4txt | Table5R.. | Total

----------------+----------------------+----------

| 195 1 | 196

----------------+----------------------+----------

Total | 195 1 | 196

(195 missing values generated)

(195 missing values generated)

(1 real change made)

(195 missing values generated)

(0 real changes made)

Gaming only | PGscaleYN

Ldepbi1txt | 0 1 . | Total

----------------------+---------------------------------+----------

| 63 124 8 | 195

Time1 depression~Ti.. | 0 1 0 | 1

----------------------+---------------------------------+----------

Total | 63 125 8 | 196

Internet | Did the study meas. PIU?

onlyLdepbi1txt | 0 1 . | Total

----------------------+---------------------------------+----------

| 127 61 8 | 196

----------------------+---------------------------------+----------

Total | 127 61 8 | 196

| Internet

| onlyLdepbi

Gaming only | 1txt

Ldepbi1txt | | Total

----------------------+-----------+----------

| 195 | 195

Time1 depression~Ti.. | 1 | 1

----------------------+-----------+----------

Total | 196 | 196

(195 missing values generated)

(195 missing values generated)

(1 real change made)

(195 missing values generated)

(0 real changes made)

Gaming only | PGscaleYN

Ldepbi2txt | 0 1 . | Total

----------------------+---------------------------------+----------

| 63 124 8 | 195

Time1PG~Time2 depre.. | 0 1 0 | 1

----------------------+---------------------------------+----------

Total | 63 125 8 | 196

Internet | Did the study meas. PIU?

onlyLdepbi2txt | 0 1 . | Total

----------------------+---------------------------------+----------

| 127 61 8 | 196

----------------------+---------------------------------+----------

Total | 127 61 8 | 196

| Internet

| onlyLdepbi

Gaming only | 2txt

Ldepbi2txt | | Total

----------------------+-----------+----------

| 195 | 195

Time1PG~Time2 depre.. | 1 | 1

----------------------+-----------+----------

Total | 196 | 196

(135 real changes made)

(71 real changes made)

Gaming |

only | PGscaleYN

Ldepbi3txt | 0 1 . | Total

-----------+---------------------------------+----------

| 63 0 8 | 71

. | 0 125 0 | 125

-----------+---------------------------------+----------

Total | 63 125 8 | 196

Internet |

onlyLdepbi | Did the study meas. PIU?

3txt | 0 1 . | Total

-----------+---------------------------------+----------

| 127 0 8 | 135

. | 0 61 0 | 61

-----------+---------------------------------+----------

Total | 127 61 8 | 196

Gaming | Internet

only | onlyLdepbi3txt

Ldepbi3txt | . | Total

-----------+----------------------+----------

| 26 45 | 71

. | 109 16 | 125

-----------+----------------------+----------

Total | 135 61 | 196

(135 real changes made)

(71 real changes made)

Gaming |

only | PGscaleYN

Ldepbi4txt | 0 1 . | Total

-----------+---------------------------------+----------

| 63 0 8 | 71

. | 0 125 0 | 125

-----------+---------------------------------+----------

Total | 63 125 8 | 196

Internet |

onlyLdepbi | Did the study meas. PIU?

4txt | 0 1 . | Total

-----------+---------------------------------+----------

| 127 0 8 | 135

. | 0 61 0 | 61

-----------+---------------------------------+----------

Total | 127 61 8 | 196

Gaming | Internet

only | onlyLdepbi4txt

Ldepbi4txt | . | Total

-----------+----------------------+----------

| 26 45 | 71

. | 109 16 | 125

-----------+----------------------+----------

Total | 135 61 | 196

(135 real changes made)

(71 real changes made)

Gaming |

only | PGscaleYN

Ldepbi5txt | 0 1 . | Total

-----------+---------------------------------+----------

| 63 0 8 | 71

. | 0 125 0 | 125

-----------+---------------------------------+----------

Total | 63 125 8 | 196

Internet |

onlyLdepbi | Did the study meas. PIU?

5txt | 0 1 . | Total

-----------+---------------------------------+----------

| 127 0 8 | 135

. | 0 61 0 | 61

-----------+---------------------------------+----------

Total | 127 61 8 | 196

Gaming | Internet

only | onlyLdepbi5txt

Ldepbi5txt | . | Total

-----------+----------------------+----------

| 26 45 | 71

. | 109 16 | 125

-----------+----------------------+----------

Total | 135 61 | 196

(135 real changes made)

(71 real changes made)

Gaming |

only | PGscaleYN

Ldepbi6txt | 0 1 . | Total

-----------+---------------------------------+----------

| 63 0 8 | 71

. | 0 125 0 | 125

-----------+---------------------------------+----------

Total | 63 125 8 | 196

Internet |

onlyLdepbi | Did the study meas. PIU?

6txt | 0 1 . | Total

-----------+---------------------------------+----------

| 127 0 8 | 135

. | 0 61 0 | 61

-----------+---------------------------------+----------

Total | 127 61 8 | 196

Gaming | Internet

only | onlyLdepbi6txt

Ldepbi6txt | . | Total

-----------+----------------------+----------

| 26 45 | 71

. | 109 16 | 125

-----------+----------------------+----------

Total | 135 61 | 196

(194 missing values generated)

(194 missing values generated)

(2 real changes made)

(194 missing values generated)

(0 real changes made)

Gaming only | PGscaleYN

Ldepmu1txt | 0 1 . | Total

----------------------+---------------------------------+----------

| 63 123 8 | 194

FD regression, weig.. | 0 1 0 | 1

StartsvNever T2 | 0 1 0 | 1

----------------------+---------------------------------+----------

Total | 63 125 8 | 196

Internet | Did the study meas. PIU?

onlyLdepmu1txt | 0 1 . | Total

----------------------+---------------------------------+----------

| 127 61 8 | 196

----------------------+---------------------------------+----------

Total | 127 61 8 | 196

| Internet

| onlyLdepmu

Gaming only | 1txt

Ldepmu1txt | | Total

----------------------+-----------+----------

| 194 | 194

FD regression, weig.. | 1 | 1

StartsvNever T2 | 1 | 1

----------------------+-----------+----------

Total | 196 | 196

(195 missing values generated)

(195 missing values generated)

(1 real change made)

(195 missing values generated)

(0 real changes made)

Gaming only | PGscaleYN

Ldepmu2txt | 0 1 . | Total

-------------------+---------------------------------+----------

| 63 124 8 | 195

Stops vs. Stays T3 | 0 1 0 | 1

-------------------+---------------------------------+----------

Total | 63 125 8 | 196

Internet | Did the study meas. PIU?

onlyLdepmu2txt | 0 1 . | Total

-------------------+---------------------------------+----------

| 127 61 8 | 196

-------------------+---------------------------------+----------

Total | 127 61 8 | 196

| Internet

| onlyLdepmu

Gaming only | 2txt

Ldepmu2txt | | Total

-------------------+-----------+----------

| 195 | 195

Stops vs. Stays T3 | 1 | 1

-------------------+-----------+----------

Total | 196 | 196

(195 missing values generated)

(195 missing values generated)

(1 real change made)

(195 missing values generated)

(0 real changes made)

Gaming only | PGscaleYN

Ldepmu3txt | 0 1 . | Total

-------------------+---------------------------------+----------

| 63 124 8 | 195

PG intercept to T3 | 0 1 0 | 1

-------------------+---------------------------------+----------

Total | 63 125 8 | 196

Internet | Did the study meas. PIU?

onlyLdepmu3txt | 0 1 . | Total

-------------------+---------------------------------+----------

| 127 61 8 | 196

-------------------+---------------------------------+----------

Total | 127 61 8 | 196

| Internet

| onlyLdepmu

Gaming only | 3txt

Ldepmu3txt | | Total

-------------------+-----------+----------

| 195 | 195

PG intercept to T3 | 1 | 1

-------------------+-----------+----------

Total | 196 | 196

(195 missing values generated)

(195 missing values generated)

(1 real change made)

(195 missing values generated)

(0 real changes made)

Gaming only | PGscaleYN

Ldepmu4txt | 0 1 . | Total

-------------------+---------------------------------+----------

| 63 124 8 | 195

Change in PG to T3 | 0 1 0 | 1

-------------------+---------------------------------+----------

Total | 63 125 8 | 196

Internet | Did the study meas. PIU?

onlyLdepmu4txt | 0 1 . | Total

-------------------+---------------------------------+----------

| 127 61 8 | 196

-------------------+---------------------------------+----------

Total | 127 61 8 | 196

| Internet

| onlyLdepmu

Gaming only | 4txt

Ldepmu4txt | | Total

-------------------+-----------+----------

| 195 | 195

Change in PG to T3 | 1 | 1

-------------------+-----------+----------

Total | 196 | 196

(135 real changes made)

(71 real changes made)

Gaming |

only | PGscaleYN

Ldepmu5txt | 0 1 . | Total

-----------+---------------------------------+----------

| 63 0 8 | 71

. | 0 125 0 | 125

-----------+---------------------------------+----------

Total | 63 125 8 | 196

Internet |

onlyLdepmu | Did the study meas. PIU?

5txt | 0 1 . | Total

-----------+---------------------------------+----------

| 127 0 8 | 135

. | 0 61 0 | 61

-----------+---------------------------------+----------

Total | 127 61 8 | 196

Gaming | Internet

only | onlyLdepmu5txt

Ldepmu5txt | . | Total

-----------+----------------------+----------

| 26 45 | 71

. | 109 16 | 125

-----------+----------------------+----------

Total | 135 61 | 196

.

.

. ***missing values in strings ended up as stata missing (.), replace these with ""

. foreach var in BLanxbi1txt BLanxmu1txt BLdepmu5txt GLanxbi1txt GLanxmu1txt ///

> ILanxbi1txt ILanxbi2txt ILanxmu1txt ILanxmu2txt Lanxbi1txt Lanxbi2txt Lanxmu1txt Lanxmu1txt {

2. replace `var'="" if `var'=="."

3. codebook `var'

4. }

(196 real changes made)

------------------------------------------------------------------------------------------------------------------------------------

BLanxbi1txt Combo PG/IA Lanxbi1txt

------------------------------------------------------------------------------------------------------------------------------------

type: string (str1), but longest is str0

unique values: 0 missing "": 196/196

tabulation: Freq. Value

196 ""

(0 real changes made)

------------------------------------------------------------------------------------------------------------------------------------

BLanxmu1txt Combo PG/IA Lanxmu1txt

------------------------------------------------------------------------------------------------------------------------------------

type: string (str34)

unique values: 2 missing "": 194/196

tabulation: Freq. Value

194 ""

1 "W5 anx on W6 # of PG sx, RSA model"

1 "anx Starts v Never T2"

warning: variable has embedded blanks

(196 real changes made)

------------------------------------------------------------------------------------------------------------------------------------

BLdepmu5txt Combo PG/IA Ldepmu5txt

------------------------------------------------------------------------------------------------------------------------------------

type: string (str1), but longest is str0

unique values: 0 missing "": 196/196

tabulation: Freq. Value

196 ""

(125 real changes made)

------------------------------------------------------------------------------------------------------------------------------------

GLanxbi1txt Gaming only Lanxbi1txt

------------------------------------------------------------------------------------------------------------------------------------

type: string (str1), but longest is str0

unique values: 0 missing "": 196/196

tabulation: Freq. Value

196 ""

(0 real changes made)

------------------------------------------------------------------------------------------------------------------------------------

GLanxmu1txt Gaming only Lanxmu1txt

------------------------------------------------------------------------------------------------------------------------------------

type: string (str34)

unique values: 2 missing "": 194/196

tabulation: Freq. Value

194 ""

1 "W5 anx on W6 # of PG sx, RSA model"

1 "anx Starts v Never T2"

warning: variable has embedded blanks

(61 real changes made)

------------------------------------------------------------------------------------------------------------------------------------

ILanxbi1txt Internet onlyLanxbi1txt

------------------------------------------------------------------------------------------------------------------------------------

type: string (str1), but longest is str0

unique values: 0 missing "": 196/196

tabulation: Freq. Value

196 ""

(61 real changes made)

------------------------------------------------------------------------------------------------------------------------------------

ILanxbi2txt Internet onlyLanxbi2txt

------------------------------------------------------------------------------------------------------------------------------------

type: string (str1), but longest is str0

unique values: 0 missing "": 196/196

tabulation: Freq. Value

196 ""

(0 real changes made)

------------------------------------------------------------------------------------------------------------------------------------

ILanxmu1txt Internet onlyLanxmu1txt

------------------------------------------------------------------------------------------------------------------------------------

type: string (str34), but longest is str0

unique values: 0 missing "": 196/196

tabulation: Freq. Value

196 ""

(0 real changes made)

------------------------------------------------------------------------------------------------------------------------------------

ILanxmu2txt Internet onlyLanxmu2txt

------------------------------------------------------------------------------------------------------------------------------------

type: string (str39), but longest is str0

unique values: 0 missing "": 196/196

tabulation: Freq. Value

196 ""

(196 real changes made)

------------------------------------------------------------------------------------------------------------------------------------

Lanxbi1txt Free text: specific assoc. b/w dep/anx and PG/IA

------------------------------------------------------------------------------------------------------------------------------------

type: string (str1), but longest is str0

unique values: 0 missing "": 196/196

tabulation: Freq. Value

196 ""

(196 real changes made)

------------------------------------------------------------------------------------------------------------------------------------

Lanxbi2txt Free text: specific assoc. b/w dep/anx and PG/IA

------------------------------------------------------------------------------------------------------------------------------------

type: string (str1), but longest is str0

unique values: 0 missing "": 196/196

tabulation: Freq. Value

196 ""

(0 real changes made)

------------------------------------------------------------------------------------------------------------------------------------

Lanxmu1txt Free text: specific assoc. b/w dep/anx and PG/IA

------------------------------------------------------------------------------------------------------------------------------------

type: string (str34)

unique values: 2 missing "": 194/196

tabulation: Freq. Value

194 ""

1 "W5 anx on W6 # of PG sx, RSA model"

1 "anx Starts v Never T2"

warning: variable has embedded blanks

(0 real changes made)

------------------------------------------------------------------------------------------------------------------------------------

Lanxmu1txt Free text: specific assoc. b/w dep/anx and PG/IA

------------------------------------------------------------------------------------------------------------------------------------

type: string (str34)

unique values: 2 missing "": 194/196

tabulation: Freq. Value

194 ""

1 "W5 anx on W6 # of PG sx, RSA model"

1 "anx Starts v Never T2"

warning: variable has embedded blanks

.

.

. *. GAMING VOTE COUNT (NOT PIU)

.

. codebook Ganxbi*vote, compact

Variable Obs Unique Mean Min Max Label

------------------------------------------------------------------------------------------------------------------------------------

Ganxbi1vote 25 2 .88 0 1 Gaming only anxbi1vote

Ganxbi2vote 16 2 .6875 0 1 Gaming only anxbi2vote

Ganxbi3vote 7 2 .7142857 0 1 Gaming only anxbi3vote

Ganxbi4vote 4 2 .5 0 1 Gaming only anxbi4vote

Ganxbi5vote 3 2 .3333333 0 1 Gaming only anxbi5vote

Ganxbi6vote 2 2 .5 0 1 Gaming only anxbi6vote

Ganxbi7vote 1 1 0 0 0 Gaming only anxbi7vote

Ganxbi8vote 1 1 0 0 0 Gaming only anxbi8vote

Ganxbi9vote 1 1 0 0 0 Gaming only anxbi9vote

------------------------------------------------------------------------------------------------------------------------------------

. codebook Ganxmu*vote, compact

Variable Obs Unique Mean Min Max Label

------------------------------------------------------------------------------------------------------------------------------------

Ganxmu1vote 7 3 .2857143 -1 1 Gaming only anxmu1vote

Ganxmu2vote 3 2 .3333333 0 1 Gaming only anxmu2vote

Ganxmu3vote 0 0 . . . Gaming only anxmu3vote

Ganxmu4vote 0 0 . . . Gaming only anxmu4vote

Ganxmu5vote 0 0 . . . Gaming only anxmu5vote

Ganxmu6vote 0 0 . . . Gaming only anxmu6vote

Ganxmu7vote 0 0 . . . Gaming only anxmu7vote

Ganxmu8vote 0 0 . . . Gaming only anxmu8vote

Ganxmu9vote 0 0 . . . Gaming only anxmu9vote

------------------------------------------------------------------------------------------------------------------------------------

. codebook GLanxbi*vote, compact

Variable Obs Unique Mean Min Max Label

------------------------------------------------------------------------------------------------------------------------------------

GLanxbi1vote 0 0 . . . Gaming only Lanxbi1vote

GLanxbi2vote 0 0 . . . Gaming only Lanxbi2vote

GLanxbi3vote 0 0 . . . Gaming only Lanxbi3vote

GLanxbi4vote 0 0 . . . Gaming only Lanxbi4vote

GLanxbi5vote 0 0 . . . Gaming only Lanxbi5vote

GLanxbi6vote 0 0 . . . Gaming only Lanxbi6vote

------------------------------------------------------------------------------------------------------------------------------------

. codebook GLanxmu*vote, compact

Variable Obs Unique Mean Min Max Label

------------------------------------------------------------------------------------------------------------------------------------

GLanxmu1vote 2 1 1 1 1 Gaming only Lanxmu1vote

GLanxmu2vote 2 2 0 -1 1 Gaming only Lanxmu2vote

GLanxmu3vote 2 1 1 1 1 Gaming only Lanxmu3vote

GLanxmu4vote 2 1 1 1 1 Gaming only Lanxmu4vote

GLanxmu5vote 1 1 1 1 1 Gaming only Lanxmu5vote

GLanxmu6vote 1 1 1 1 1 Gaming only Lanxmu6vote

GLanxmu7vote 1 1 1 1 1 Gaming only Lanxmu7vote

GLanxmu8vote 1 1 1 1 1 Gaming only Lanxmu8vote

GLanxmu9vote 1 1 1 1 1 Gaming only Lanxmu9vote

GLanxmu10v~e 1 1 1 1 1 Gaming only Lanxmu10vote

GLanxmu11v~e 1 1 1 1 1 Gaming only Lanxmu11vote

------------------------------------------------------------------------------------------------------------------------------------

. codebook Gdepbi*vote, compact

Variable Obs Unique Mean Min Max Label

------------------------------------------------------------------------------------------------------------------------------------

Gdepbi1vote 27 2 .8888889 0 1 Gaming only depbi1vote

Gdepbi2vote 12 2 .9166667 0 1 Gaming only depbi2vote

Gdepbi3vote 4 1 1 1 1 Gaming only depbi3vote

Gdepbi4vote 1 1 0 0 0 Gaming only depbi4vote

Gdepbi5vote 1 1 0 0 0 Gaming only depbi5vote

Gdepbi6vote 1 1 0 0 0 Gaming only depbi6vote

Gdepbi7vote 1 1 1 1 1 Gaming only depbi7vote

Gdepbi8vote 1 1 0 0 0 Gaming only depbi8vote

Gdepbi9vote 0 0 . . . Gaming only depbi9vote

------------------------------------------------------------------------------------------------------------------------------------

. codebook Gdepmu*vote, compact

Variable Obs Unique Mean Min Max Label

------------------------------------------------------------------------------------------------------------------------------------

Gdepmu1vote 13 2 .6153846 0 1 Gaming only depmu1vote

Gdepmu2vote 3 2 .6666667 0 1 Gaming only depmu2vote

Gdepmu3vote 1 1 1 1 1 Gaming only depmu3vote

Gdepmu4vote 0 0 . . . Gaming only depmu4vote

------------------------------------------------------------------------------------------------------------------------------------

. codebook GLdepbi*vote, compact

Variable Obs Unique Mean Min Max Label

------------------------------------------------------------------------------------------------------------------------------------

GLdepbi1vote 1 1 0 0 0 Gaming only Ldepbi1vote

GLdepbi2vote 1 1 0 0 0 Gaming only Ldepbi2vote

GLdepbi3vote 0 0 . . . Gaming only Ldepbi3vote

GLdepbi4vote 0 0 . . . Gaming only Ldepbi4vote

GLdepbi5vote 0 0 . . . Gaming only Ldepbi5vote

GLdepbi6vote 0 0 . . . Gaming only Ldepbi6vote

------------------------------------------------------------------------------------------------------------------------------------

. codebook GLdepmu*vote, compact

Variable Obs Unique Mean Min Max Label

------------------------------------------------------------------------------------------------------------------------------------

GLdepmu1vote 2 1 1 1 1 Gaming only Ldepmu1vote

GLdepmu2vote 1 1 1 1 1 Gaming only Ldepmu2vote

GLdepmu3vote 1 1 1 1 1 Gaming only Ldepmu3vote

GLdepmu4vote 1 1 1 1 1 Gaming only Ldepmu4vote

GLdepmu5vote 0 0 . . . Gaming only Ldepmu5vote

------------------------------------------------------------------------------------------------------------------------------------

.

.

. ******************************************************************************

.

. **generate vars that show how many PG results a study reports for dep/anx ///

> *****report is at the level of the empirical study

. *****these don't have to be missing

. ds Gdepbi*vote

Gdepbi1vote Gdepbi2vote Gdepbi3vote Gdepbi4vote Gdepbi5vote Gdepbi6vote Gdepbi7vote Gdepbi8vote Gdepbi9vote

. cap drop tot_dep_bi

. egen tot_dep_bi=rownonmiss(Gdepbi1vote Gdepbi2vote Gdepbi3vote Gdepbi4vote Gdepbi5vote Gdepbi6vote Gdepbi7vote Gdepbi8vote Gdepbi9

> vote)

. lab var tot_dep_bi "Total # bivar PG-dep results/indiv study"

. codebook tot_dep_bi

------------------------------------------------------------------------------------------------------------------------------------

tot_dep_bi Total # bivar PG-dep results/indiv study

------------------------------------------------------------------------------------------------------------------------------------

type: numeric (float)

range: [0,8] units: 1

unique values: 5 missing .: 0/196

tabulation: Freq. Value

169 0

15 1

8 2

3 3

1 8

.

. ds Ganxbi*vote

Ganxbi1vote Ganxbi2vote Ganxbi3vote Ganxbi4vote Ganxbi5vote Ganxbi6vote Ganxbi7vote Ganxbi8vote Ganxbi9vote

. cap drop tot_anx_bi

. egen tot_anx_bi=rownonmiss(Ganxbi1vote Ganxbi2vote Ganxbi3vote Ganxbi4vote Ganxbi5vote Ganxbi6vote Ganxbi7vote Ganxbi8vote

> Ganxbi9vote)

. lab var tot_anx_bi "Total # bivar PG-anx results/indiv study"

. codebook tot_anx_bi

------------------------------------------------------------------------------------------------------------------------------------

tot_anx_bi Total # bivar PG-anx results/indiv study

------------------------------------------------------------------------------------------------------------------------------------

type: numeric (float)

range: [0,9] units: 1

unique values: 8 missing .: 0/196

tabulation: Freq. Value

171 0

9 1

9 2

3 3

1 4

1 5

1 6

1 9

.

. ds Ganxmu*vote

Ganxmu1vote Ganxmu2vote Ganxmu3vote Ganxmu4vote Ganxmu5vote Ganxmu6vote Ganxmu7vote Ganxmu8vote Ganxmu9vote

. cap drop tot_anx_mu

. egen tot_anx_mu=rownonmiss(Ganxmu1vote Ganxmu2vote Ganxmu3vote Ganxmu4vote Ganxmu5vote Ganxmu6vote Ganxmu7vote Ganxmu8vote Gan

> xmu9vote)

. lab var tot_anx_mu "Total # multivar PG-anx results/indiv study"

. codebook tot_anx_mu

------------------------------------------------------------------------------------------------------------------------------------

tot_anx_mu Total # multivar PG-anx results/indiv study

------------------------------------------------------------------------------------------------------------------------------------

type: numeric (float)

range: [0,2] units: 1

unique values: 3 missing .: 0/196

tabulation: Freq. Value

189 0

4 1

3 2

.

. ds Gdepmu*vote

Gdepmu1vote Gdepmu2vote Gdepmu3vote Gdepmu4vote

. cap drop tot_dep_mu

. egen tot_dep_mu=rownonmiss(Gdepmu1vote Gdepmu2vote Gdepmu3vote Gdepmu4vote)

. lab var tot_dep_mu "Total # multivar PG-dep results/indiv study"

. codebook tot_dep_mu

------------------------------------------------------------------------------------------------------------------------------------

tot_dep_mu Total # multivar PG-dep results/indiv study

------------------------------------------------------------------------------------------------------------------------------------

type: numeric (float)

range: [0,3] units: 1

unique values: 4 missing .: 0/196

tabulation: Freq. Value

183 0

10 1

2 2

1 3

.

. ds GLanxbi*vote

GLanxbi1vote GLanxbi2vote GLanxbi3vote GLanxbi4vote GLanxbi5vote GLanxbi6vote

. cap drop tot_Lanx_bi

. egen tot_Lanx_bi=rownonmiss(GLanxbi1vote GLanxbi2vote GLanxbi3vote GLanxbi4vote GLanxbi5vote GLanxbi6vote)

. lab var tot_Lanx_bi "Total # longit bivar PG-anx results/indiv study"

. codebook tot_Lanx_bi

------------------------------------------------------------------------------------------------------------------------------------

tot_Lanx_bi Total # longit bivar PG-anx results/indiv study

------------------------------------------------------------------------------------------------------------------------------------

type: numeric (float)

range: [0,0] units: 1

unique values: 1 missing .: 0/196

tabulation: Freq. Value

196 0

.

. ds GLanxmu*vote

GLanxmu1vote GLanxmu3vote GLanxmu5vote GLanxmu7vote GLanxmu9vote GLanxmu11v~e

GLanxmu2vote GLanxmu4vote GLanxmu6vote GLanxmu8vote GLanxmu10v~e

. cap drop tot_Lanxmu

. egen tot_Lanxmu=rownonmiss(GLanxmu1vote GLanxmu3vote GLanxmu5vote GLanxmu7vote GLanxmu9vote GLanxmu2vote GLanxmu4vote

> GLanxmu6vote GLanxmu8vote GLanxmu10vote GLanxmu11vote)

. lab var tot_Lanxmu "Total # longit multivar PG-anx results/indiv study"

. codebook tot_Lanxmu

------------------------------------------------------------------------------------------------------------------------------------

tot_Lanxmu Total # longit multivar PG-anx results/indiv study

------------------------------------------------------------------------------------------------------------------------------------

type: numeric (float)

range: [0,11] units: 1

unique values: 3 missing .: 0/196

tabulation: Freq. Value

194 0

1 4

1 11

.

. ds GLdepbi*vote

GLdepbi1vote GLdepbi2vote GLdepbi3vote GLdepbi4vote GLdepbi5vote GLdepbi6vote

. cap drop tot_Ldep_bi

. egen tot_Ldep_bi=rownonmiss(GLdepbi1vote GLdepbi2vote GLdepbi3vote GLdepbi4vote GLdepbi5vote GLdepbi6vote)

. lab var tot_Ldep_bi "Total # longit bivar PG-dep results/indiv study"

. codebook tot_Ldep_bi

------------------------------------------------------------------------------------------------------------------------------------

tot_Ldep_bi Total # longit bivar PG-dep results/indiv study

------------------------------------------------------------------------------------------------------------------------------------

type: numeric (float)

range: [0,2] units: 1

unique values: 2 missing .: 0/196

tabulation: Freq. Value

195 0

1 2

.

. ds GLdepmu*vote

GLdepmu1vote GLdepmu2vote GLdepmu3vote GLdepmu4vote GLdepmu5vote

. cap drop tot_Ldepmu

. egen tot_Ldepmu=rownonmiss(GLdepmu1vote GLdepmu2vote GLdepmu3vote GLdepmu4vote GLdepmu5vote)

. lab var tot_Ldepmu "Total # longit multiv PG-dep results/indiv study"

. codebook tot_Ldepmu

------------------------------------------------------------------------------------------------------------------------------------

tot_Ldepmu Total # longit multiv PG-dep results/indiv study

------------------------------------------------------------------------------------------------------------------------------------

type: numeric (float)

range: [0,4] units: 1

unique values: 3 missing .: 0/196

tabulation: Freq. Value

194 0

1 1

1 4

.

.

. ****GENERATE TOTAL number of resultS BY COMBINING BIVARIATE AND MULTIVARIATE AT STUDY LEVEL

. *******both longit and cross-sect

. ********These are specificleano gaming b/c previous variables are created from G`var'

. cap drop alldep

. bys id: gen alldep=tot_dep_bi+tot_dep_mu+tot_Ldep_bi+tot_Ldepmu

. lab var alldep "Total NUMBER of depression results in study"

. codebook alldep

------------------------------------------------------------------------------------------------------------------------------------

alldep Total NUMBER of depression results in study

------------------------------------------------------------------------------------------------------------------------------------

type: numeric (float)

range: [0,8] units: 1

unique values: 7 missing .: 0/196

tabulation: Freq. Value

165 0

12 1

8 2

6 3

2 4

2 5

1 8

.

. cap drop allanx

. bys id: gen allanx=tot_anx_bi+tot_anx_mu+tot_Lanx_bi+tot_Lanxmu

. lab var allanx "Total NUMBER of anxiety results in study"

. codebook allanx

------------------------------------------------------------------------------------------------------------------------------------

allanx Total NUMBER of anxiety results in study

------------------------------------------------------------------------------------------------------------------------------------

type: numeric (float)

range: [0,11] units: 1

unique values: 9 missing .: 0/196

tabulation: Freq. Value

168 0

7 1

9 2

3 3

5 4

1 5

1 6

1 9

1 11

.

. **test to make sure the indicator for "was there a depression result" /

. ***"Was there an anxiety result" (alldep/allanx) is not missing if first G`var' is not missing

. foreach var in GLdepbi1vote GLdepmu1vote Gdepmu1vote Gdepbi1vote {

2. assert alldep>0 if `var' !=.

3. }

.

. foreach var in GLanxbi1vote GLanxmu1vote Ganxmu1vote Ganxbi1vote {

2. assert allanx>0 if `var' !=.

3. }

(null assertion)

.

. ********Make binary variables to condition on anx and dep

. ***********in some cases, depression

. ***********results could be present if PGscaleYN==0

. cap drop Gbindep

. clonevar Gbindep=alldep

. codebook Gbindep

------------------------------------------------------------------------------------------------------------------------------------

Gbindep Total NUMBER of depression results in study

------------------------------------------------------------------------------------------------------------------------------------

type: numeric (float)

range: [0,8] units: 1

unique values: 7 missing .: 0/196

tabulation: Freq. Value

165 0

12 1

8 2

6 3

2 4

2 5

1 8

. recode Gbindep 2/8=1

(Gbindep: 19 changes made)

. lab var Gbindep "Binary indicator that study measures PG-Dep"

. codebook Gbindep

------------------------------------------------------------------------------------------------------------------------------------

Gbindep Binary indicator that study measures PG-Dep

------------------------------------------------------------------------------------------------------------------------------------

type: numeric (float)

range: [0,1] units: 1

unique values: 2 missing .: 0/196

tabulation: Freq. Value

165 0

31 1

. tab alldep Gbindep, mi

Total |

NUMBER of |

depression | Binary indicator that

results in | study measures PG-Dep

study | 0 1 | Total

-----------+----------------------+----------

0 | 165 0 | 165

1 | 0 12 | 12

2 | 0 8 | 8

3 | 0 6 | 6

4 | 0 2 | 2

5 | 0 2 | 2

8 | 0 1 | 1

-----------+----------------------+----------

Total | 165 31 | 196

. tab Gbindep, mi

Binary |

indicator |

that study |

measures |

PG-Dep | Freq. Percent Cum.

------------+-----------------------------------

0 | 165 84.18 84.18

1 | 31 15.82 100.00

------------+-----------------------------------

Total | 196 100.00

. tab Gbindep PGscaleYN, mi

Binary |

indicator |

that study |

measures | PGscaleYN

PG-Dep | 0 1 . | Total

-----------+---------------------------------+----------

0 | 63 94 8 | 165

1 | 0 31 0 | 31

-----------+---------------------------------+----------

Total | 63 125 8 | 196

. tab Gbindep wasitPIU, mi

Binary |

indicator |

that study |

measures | Did the study meas. PIU?

PG-Dep | 0 1 . | Total

-----------+---------------------------------+----------

0 | 101 56 8 | 165

1 | 26 5 0 | 31

-----------+---------------------------------+----------

Total | 127 61 8 | 196

.

. cap drop Gbinanx

. clonevar Gbinanx=allanx

. codebook Gbinanx

------------------------------------------------------------------------------------------------------------------------------------

Gbinanx Total NUMBER of anxiety results in study

------------------------------------------------------------------------------------------------------------------------------------

type: numeric (float)

range: [0,11] units: 1

unique values: 9 missing .: 0/196

tabulation: Freq. Value

168 0

7 1

9 2

3 3

5 4

1 5

1 6

1 9

1 11

. recode Gbinanx 2/11=1

(Gbinanx: 21 changes made)

. lab var Gbinanx "Binary indicator that study measures PG-Anx"

. codebook Gbinanx

------------------------------------------------------------------------------------------------------------------------------------

Gbinanx Binary indicator that study measures PG-Anx

------------------------------------------------------------------------------------------------------------------------------------

type: numeric (float)

range: [0,1] units: 1

unique values: 2 missing .: 0/196

tabulation: Freq. Value

168 0

28 1

. tab allanx Gbinanx, mi

Total |

NUMBER of |

anxiety | Binary indicator that

results in | study measures PG-Anx

study | 0 1 | Total

-----------+----------------------+----------

0 | 168 0 | 168

1 | 0 7 | 7

2 | 0 9 | 9

3 | 0 3 | 3

4 | 0 5 | 5

5 | 0 1 | 1

6 | 0 1 | 1

9 | 0 1 | 1

11 | 0 1 | 1

-----------+----------------------+----------

Total | 168 28 | 196

. tab Gbinanx, mi

Binary |

indicator |

that study |

measures |

PG-Anx | Freq. Percent Cum.

------------+-----------------------------------

0 | 168 85.71 85.71

1 | 28 14.29 100.00

------------+-----------------------------------

Total | 196 100.00

. tab Gbinanx PGscaleYN, mi

Binary |

indicator |

that study |

measures | PGscaleYN

PG-Anx | 0 1 . | Total

-----------+---------------------------------+----------

0 | 63 97 8 | 168

1 | 0 28 0 | 28

-----------+---------------------------------+----------

Total | 63 125 8 | 196

. tab Gbinanx wasitPIU, mi

Binary |

indicator |

that study |

measures | Did the study meas. PIU?

PG-Anx | 0 1 . | Total

-----------+---------------------------------+----------

0 | 104 56 8 | 168

1 | 23 5 0 | 28

-----------+---------------------------------+----------

Total | 127 61 8 | 196

.

. assert (Gbinanx>0 & Gbinanx<.) if Ganxbi1vote <. | Ganxmu1vote <. | GLanxbi1vote<. | GLanxmu1vote<.

. assert (Gbindep>0 & Gbindep<.) if Gdepbi1vote <. | Gdepmu1vote <. | GLdepbi1vote<. | GLdepmu1vote<.

. **First example commented fully

. ****We want to preserve the missing when using egen. There's probably a better way to do it

. ******but this works:

.

. *******************************************************************************

. *Count # positive findings for gaming’s anx bivar relationships

. *******************************************************************************

. *******************************************************************************

.

. *********Make sure the vote vars are missing if the first vote var is missing

. *********then replace the egen vars with missing if there is no value for the first var

. ds Ganxbi*vote

Ganxbi1vote Ganxbi2vote Ganxbi3vote Ganxbi4vote Ganxbi5vote Ganxbi6vote Ganxbi7vote Ganxbi8vote Ganxbi9vote

. foreach var in Ganxbi2vote Ganxbi3vote Ganxbi4vote Ganxbi5vote Ganxbi6vote Ganxbi7vote Ganxbi8vote Ganxbi9vote {

2. assert `var'==. if Ganxbi1vote==.

3. }

.

. capture drop GabP

. ****any time you see a 1 in the vote, count that as positive and add those up for each study

. egen GabP=anycount(Ganxbi1vote Ganxbi2vote Ganxbi3vote Ganxbi4vote Ganxbi5vote Ganxbi6vote Ganxbi7vote Ganxbi8vote Ganxbi9vote), v

> alues(1)

.

. ****preserve the missing values by study

. replace GabP=. if Ganxbi1vote==. | GabP==0

(173 real changes made, 173 to missing)

. lab var GabP "No. (+) results for bivar anx STUDY lvl"

. codebook GabP

------------------------------------------------------------------------------------------------------------------------------------

GabP No. (+) results for bivar anx STUDY lvl

------------------------------------------------------------------------------------------------------------------------------------

type: numeric (byte)

range: [1,6] units: 1

unique values: 4 missing .: 173/196

tabulation: Freq. Value

11 1

8 2

3 3

1 6

173 .

. list study GabP Ganxbi1vote Ganxbi2vote Ganxbi3vote Ganxbi4vote Ganxbi5vote Ganxbi6vote Ganxbi7vote Ganxbi8vote Ganxbi9vote if ///

> Ganxbi1vote !=.

+--------------------------------------------------------------------------------------------------------------------------+

| study GabP Ganxbi.. Ganxbi.. Ganxbi.. Ganxbi.. Ganxbi.. Ganxbi.. Ganxbi.. Ganxbi.. Ganxbi.. |

|--------------------------------------------------------------------------------------------------------------------------|

2. | Allison_2006 3 1 1 1 . . . . . . |

3. | Andreass_Sch 1 1 . . . . . . . . |

6. | Batthya_2009 1 1 . . . . . . . . |

11. | Bouna-P 1 1 0 0 . . . . . . |

64. | Kim_2010 1 1 . . . . . . . . |

|--------------------------------------------------------------------------------------------------------------------------|

66. | Kim_NH_2016 2 1 1 . . . . . . . |

67. | Kim_NR_2016 2 1 1 . . . . . . . |

71. | King_2011 1 1 . . . . . . . . |

72. | King_2013_Axis . 0 0 0 0 0 0 0 0 0 |

89. | Lemos_2016 6 1 1 1 1 1 1 . . . |

|--------------------------------------------------------------------------------------------------------------------------|

95. | Manniko_2015 2 1 1 . . . . . . . |

98. | Mehroof_2010 2 1 1 . . . . . . . |

99. | Mentzoni_2011 1 1 . . . . . . . . |

115. | Rehbein_2010 1 1 0 . . . . . . . |

127. | Starcevic_2011 3 1 0 1 1 0 . . . . |

|--------------------------------------------------------------------------------------------------------------------------|

131. | Tejeiro_2012 . 0 . . . . . . . . |

136. | Topor_2011 1 0 0 1 0 . . . . . |

142. | VRooij_2012 2 1 1 . . . . . . . |

143. | Vukosc-G_2015 2 1 1 . . . . . . . |

144. | Walther_2012 1 1 . . . . . . . . |

|--------------------------------------------------------------------------------------------------------------------------|

158. | Jimenez_2014 2 1 1 . . . . . . . |

159. | King_2016 2 1 1 . . . . . . . |

161. | Na_2017 1 1 . . . . . . . . |

165. | Vadlin_5/2016 1 1 . . . . . . . . |

196. | Yu_2016 3 1 1 1 . . . . . . |

+--------------------------------------------------------------------------------------------------------------------------+

. ********

. *******i.e., some studies have up to 6 anxiety results that show positive associations

.

. *count No. of "reverse" findings--e.g., being less anxious

. capture drop GabR

. ***any time you see a -1 in the vote, count that as negative and add up the number of negative results for each study

. egen GabR= anycount(Ganxbi1vote Ganxbi2vote Ganxbi3vote Ganxbi4vote Ganxbi5vote Ganxbi6vote Ganxbi7vote Ganxbi8vote Ganxbi9vote),

> values(-1)

. replace GabR=. if Ganxbi1vote==. | GabR==0

(196 real changes made, 196 to missing)

. *of the 26 studies that have bivariate anxiety results, none have reverse associations

.

.

. *same for null findings

. capture drop GabN

. ***any time you see a 0 in the vote, count that as null and add up the number of null results for each study

. egen GabN=anycount(Ganxbi1vote Ganxbi2vote Ganxbi3vote Ganxbi4vote Ganxbi5vote Ganxbi6vote Ganxbi7vote Ganxbi8vote Ganxbi9vote), v

> alues(0)

. replace GabN=. if Ganxbi1vote==. | GabN==0

(190 real changes made, 190 to missing)

. lab var GabN "No. of null results (e.g.,NS) for bivar anx STUDY lvl"

. codebook GabN

------------------------------------------------------------------------------------------------------------------------------------

GabN No. of null results (e.g.,NS) for bivar anx STUDY lvl

------------------------------------------------------------------------------------------------------------------------------------

type: numeric (byte)

range: [1,9] units: 1

unique values: 4 missing .: 190/196

tabulation: Freq. Value

2 1

2 2

1 3

1 9

190 .

. ********* range 0-9

. list id study *anx* if GabN==9

+-----------------------------------------------------------------------------------------------------------------------------+

72. | id | study | PGanxy~o | anxmu1~t | anxmu1~e | anxmu2~t | anxmu2~e | anxmu3~t | anxmu3~e | anxmu4~t | anxmu4~e |

| 72 | King_2013_Axis | 1_Yes | | . | | . | | . | | . |

|---------------------+----------+----------+----------+----------+-----------+-----------+-----------+-----------+-----------|

| anxmu5~t | anxmu5~e | anxmu6~t | anxmu6~e | anxmu7~t | anxmu7~e | anxmu8~t | anxmu8~e | anxmu9~t | anxmu9~e | anxbi1~t |

| | . | | . | | . | | . | | . | SAS sub1 |

|-----------------------------------------------------------------------------------------------------------------------------|

| anxbi1~e | anxbi2~t | anxbi2~e | anxbi3~t | anxbi3~e | anxbi4txt | anxbi4~e | anxbi5txt | anxbi5~e |

| 0 | SAS sub2 | 0 | SAS sub3 | 0 | RCADS anxiety | 0 | RCADS panic | 0 |

|----------------------------------------------------------------+------------------------------------------------------------|

| anxbi6txt | anxbi6~e | anxbi7txt | anxbi7~e | anxbi8txt | anxbi8~e | anxbi9~t | anxbi9~e | La~i1txt |

| RCADS separation anxiety | 0 | RCADS soc | 0 | RCADS tot | 0 | SAS tot | 0 | |

|-----------------------------------------------------------------------------+-----------+-----------+-----------+-----------|

| Lanxbi.. | La~i2txt | Lanxbi.. | La~i3txt | Lanxbi.. | La~i4txt | Lanxbi.. | La~i5txt | Lanxbi.. | La~i6txt | Lanxbi.. |

| . | | . | . | . | . | . | . | . | . | . |

|----------+----------+----------+----------+----------+----------+-----------+-----------+-----------+-----------+-----------|

| La~u1txt | Lanxmu.. | La~u2txt | Lanxmu.. | La~u3txt | Lanxmu.. | La~u4txt | Lanxmu.. | La~u5txt | Lanxmu.. | La~u6txt |

| | . | | . | | . | | . | | . | |

|----------+----------+----------+----------+----------+----------+-----------+-----------+-----------+-----------+-----------|

| L~u6vote | Lan~7txt | La~7vote | Lan~8txt | La~8vote | Lan~9txt | La~9vote | Lan~0txt | La~0vote | La~11txt | L~11vote |

| . | | . | | . | | . | | . | | . |

|-----------------------------------------------------------------+-----------+-----------+-----------+-----------+-----------|

| old_PGanxyesno | IAanxy~o | depanx~e | sug_anx1 | sug_anx2 | sug_anx3 | sug_La~1 | sug_La~2 | gonz_a~1 | gonz_a~2 |

| 2_both PG & PIU | 1 | | . | . | . | . | . | 0 | . |

|-----------------------------------------------------------------+-----------+-----------+-----------+-----------+-----------|

| gonz_a~3 | gonz_a~4 | go~Lanx1 | go~Lanx2 | miha_a~1 | miha_a~2 | miha_a~3 | miha_a~4 | mi~Lanx1 | mi~Lanx2 | king_a~1 |

| . | . | . | . | 1 | 1 | . | . | . | . | . |

|----------+----------+----------+----------+----------+----------+-----------+-----------+-----------+-----------+-----------|

| king_a~2 | ki~Lanx1 | ki~Lanx2 | man_anx1 | man_anx2 | man_La~1 | man_La~2 | kuon_a~1 | kuon_a~2 | kuon_L.. | kuon_L.. |

| . | . | . | . | . | . | . | . | . | . | . |

|----------+----------+----------+----------+----------+----------+-----------+-----------+-----------+-----------+-----------|

| k~t_anx1 | k~t_anx2 | kuint_~3 | ~t_Lanx1 | ~t_Lanx2 | Banxbi.. | Ianxbi.. | Ganxbi.. | Banxbi.. | Ianxbi.. | Ganxbi.. |

| . | . | . | . | . | 0 | 0 | 0 | 0 | 0 | 0 |

|----------+----------+----------+----------+----------+----------+-----------+-----------+-----------+-----------+-----------|

| Banxbi.. | Ianxbi.. | Ganxbi.. | Banxbi.. | Ianxbi.. | Ganxbi.. | Banxbi.. | Ianxbi.. | Ganxbi.. | Banxbi.. | Ianxbi.. |

| 0 | 0 | 0 | 0 | 0 | 0 | 0 | 0 | 0 | 0 | 0 |

|----------+----------+----------+----------+----------+----------+-----------+-----------+-----------+-----------+-----------|

| Ganxbi.. | Banxbi.. | Ianxbi.. | Ganxbi.. | Banxbi.. | Ianxbi.. | Ganxbi.. | Banxbi.. | Ianxbi.. | Ganxbi.. | Banxmu.. |

| 0 | 0 | 0 | 0 | 0 | 0 | 0 | 0 | 0 | 0 | . |

|----------+----------+----------+----------+----------+----------+-----------+-----------+-----------+-----------+-----------|

| Ianxmu.. | Ganxmu.. | Banxmu.. | Ianxmu.. | Ganxmu.. | Banxmu.. | Ianxmu.. | Ganxmu.. | Banxmu.. | Ianxmu.. | Ganxmu.. |

| . | . | . | . | . | . | . | . | . | . | . |

|----------+----------+----------+----------+----------+----------+-----------+-----------+-----------+-----------+-----------|

| Banxmu.. | Ianxmu.. | Ganxmu.. | Banxmu.. | Ianxmu.. | Ganxmu.. | Banxmu.. | Ianxmu.. | Ganxmu.. | Banxmu.. | Ianxmu.. |

| . | . | . | . | . | . | . | . | . | . | . |

|----------+----------+----------+----------+----------+----------+-----------+-----------+-----------+-----------+-----------|

| Ganxmu.. | Banxmu.. | Ianxmu.. | Ganxmu.. | BLanxb.. | ILanxb.. | GLanxb.. | BLanxb.. | ILanxb.. | GLanxb.. | BLanxb.. |

| . | . | . | . | . | . | . | . | . | . | . |

|----------+----------+----------+----------+----------+----------+-----------+-----------+-----------+-----------+-----------|

| ILanxb.. | GLanxb.. | BLanxb.. | ILanxb.. | GLanxb.. | BLanxb.. | ILanxb.. | GLanxb.. | BLanxb.. | ILanxb.. | GLanxb.. |

| . | . | . | . | . | . | . | . | . | . | . |

|----------+----------+----------+----------+----------+----------+-----------+-----------+-----------+-----------+-----------|

| BLanxm.. | ILanxm.. | GLanxm.. | BLanxm.. | ILanxm.. | GLanxm.. | BLanxm.. | ILanxm.. | GLanxm.. | BLanxm.. | ILanxm.. |

| . | . | . | . | . | . | . | . | . | . | . |

|----------+----------+----------+----------+----------+----------+-----------+-----------+-----------+-----------+-----------|

| GLanxm.. | BLanxm.. | ILanxm.. | GLanxm.. | BLanxm.. | ILanxm.. | GLanxm.. | BL~7vote | IL~7vote | GL~7vote | BL~8vote |

| . | . | . | . | . | . | . | . | . | . | . |

|----------+----------+----------+----------+----------+----------+-----------+-----------+-----------+-----------+-----------|

| IL~8vote | GL~8vote | BL~9vote | IL~9vote | GL~9vote | BL~0vote | IL~0vote | GL~0vote | B~11vote | I~11vote | G~11vote |

| . | . | . | . | . | . | . | . | . | . | . |

|-----------------------------------------------------------------------------------------------------------------------------|

| Ba~i1txt | Ia~i1txt | Ga~i1txt | Ba~i2txt | Ia~i2txt | Ga~i2txt | Ba~i3txt | Ia~i3txt | Ga~i3txt | Banxbi4txt |

| SAS sub1 | SAS sub1 | SAS sub1 | SAS sub2 | SAS sub2 | SAS sub2 | SAS sub3 | SAS sub3 | SAS sub3 | RCADS anxiety |

|-----------------------------------------------------------------------------------------------------------------------------|

| Ianxbi4txt | Ganxbi4txt | Banxbi5txt | Ianxbi5txt | Ganxbi5txt | Banxbi6txt |

| RCADS anxiety | RCADS anxiety | RCADS panic | RCADS panic | RCADS panic | RCADS separation anxiety |

|-----------------------------------------------------------------------------------------------------------------------------|

| Ianxbi6txt | Ganxbi6txt | Banxbi7~t | Ianxbi7~t | Ganxbi7~t | Banxbi8~t | Ianxbi8~t | Ganxbi8~t |

| RCADS separation anxiety | RCADS separation anxiety | RCADS soc | RCADS soc | RCADS soc | RCADS tot | RCADS tot | RCADS tot |

|-----------------------------------------------------------------+-----------+-----------+-----------+-----------+-----------|

| Ba~i9txt | Ia~i9txt | Ga~i9txt | Ba~u1txt | Ia~u1txt | Ga~u1txt | Ba~u2txt | Ia~u2txt | Ga~u2txt | Ba~u3txt | Ia~u3txt |

| SAS tot | SAS tot | SAS tot | | | | | | | | |

|----------+----------+----------+----------+----------+----------+-----------+-----------+-----------+-----------+-----------|
[truncated: 1,353,928 more chars]
