# Supplementary material for: Evaluating the quality of evidence for gaming disorder: A summary of systematic reviews of associations between gaming disorder and depression or anxiety
Source: PLoS One. 2020 Oct 26;15(10):e0240032. doi: 10.1371/journal.pone.0240032 (PMC7588081; doi:10.1371/journal.pone.0240032)
Supplement: Attachment — Submitted filename: PLOS One Review .docx [file pone.0240032.s007.docx]

Carras et al. conducted an interesting study analyzing the quality of evidence for gaming disorder and found that no reviews were classified as reliable. This topic is of great importance and I commend the authors for taking on this task. I additionally commend the authors for following a pre-registered protocol, for reporting results according to the PRISMA reporting standards, and for making all materials available online through OSF. Overall, this is a robust study and, with minor corrections, should be considered for publication.

- The introduction and discussion is well written and appropriately introduces and discusses the topic at hand and the importance of having high-quality reviews to provide reliable evidence for healthcare and policy decision-making. I am pleased the authors have made all materials and protocols available online. This improves the transparency and reproducibility of their work.
- Line 135 says Open Science Foundation, but the correct name is Open Science Framework.
- Please provide a statement regarding IRB approval.
- While I agree the with the classification criteria chosen in this study, it would benefit from further explanation why these 6 aspects of the criteria were chosen. The authors mention Mayo-Wilson and colleagues as part of their reasoning, yet Mayo-Wilson and colleagues reference including components of the Critical Appraisal Skills Programme (CASP), the Assessment of Multiple Systematic Reviews (AMSTAR), and the Preferred Reporting Items for Systematic Reviews (PRISMA).
- The second line in the conclusion has an extra space. While the authors mention systematic reviews should follow existing standards and should be improved, this review could benefit from providing a few more concrete suggestions on how to do so.
