# Supplementary material for: Evaluating the quality of evidence for gaming disorder: A summary of systematic reviews of associations between gaming disorder and depression or anxiety
Source: PLoS One. 2020 Oct 26;15(10):e0240032. doi: 10.1371/journal.pone.0240032 (PMC7588081; doi:10.1371/journal.pone.0240032)
Supplement: Attachment — Submitted filename: Response to reviewers.docx [file pone.0240032.s009.docx]

6. Review Comments to the Author

Please use the space provided to explain your answers to the questions above. You may also include additional comments for the author, including concerns about dual publication, research ethics, or publication ethics. (Please upload your review as an attachment if it exceeds 20,000 characters)

Reviewer #1: (No Response)

Reviewer #2: This study attempts to present a review of the quality evidence of systematic review for gaming disorder. I also reviewed a previous version of this manuscript. The authors did a good job in revising the manuscript, but I still have some minus remaining comments.
Comments
1. There is a syntax error in the sentence on line 167 page 9.

*Response: Thank you for bringing this to our attention. We have fixed this error.*

2. The authors have chosen to use your own definition using various sources to determine a reliable systematic review. Therefore, I think it would be useful for a better understanding of all, to better define your 6 criteria. For example: how did you judge the criterion 'comprehensive literature search'? Did you give a yes when the authors had consulted at least one database and had not limited their searches in English or other criteria were taken into account? The remark is valid for all items. How did you assign yes and no to the different items for each systematic review?

*Response: We have clarified the definition of reliability. As we stated in the opening sentence of the section “Assessment of reliability of reviews”, this definition was not created de novo, but was adapted from one developed and published by Cochrane Eyes and Vision. Further, we have made changes throughout the manuscript to avoid confusion between “reliability” and “quality”.*

3. Thank you for appending the search strategy used in Medline. Could you add the one used in Psycinfo to be completely transparent?

*Response: We have now also appended the search strategy that we used for searching PsycInfo (in S2 (Search Strategies)).*

4. Finally, the authors used the AMSTAR2 tool to assess the methodological quality of systematic reviews as outlined in the method. However, the results are not presented even briefly in the results section, let alone discussed in the discussion. A commentary on these results would be very interesting and could allow a comparison of the results with the existing literature on this topic.

*Response: As mentioned in our response to your comment #2 above, we have clarified the distinction between ‘reliability’ (assessed using a few items from AMSTAR and other tools) and ‘quality’ (assessed using all AMSTAR items). The focus of this paper is on reliability of reviews, so we describe the AMSTAR items relevant to reliability in the main paper, and we include the results of the full AMSTAR assessment in the appendix table (S3).*
